# The Evolution of Silicon Transport in Eukaryotes

**DOI:** 10.1093/molbev/msw209

**Published:** 2016-10-11

**Authors:** Alan O. Marron, Sarah Ratcliffe, Glen L. Wheeler, Raymond E. Goldstein, Nicole King, Fabrice Not, Colomban de Vargas, Daniel J. Richter

**Affiliations:** ^1^Department of Applied Mathematics and Theoretical Physics, Centre for Mathematical Sciences, University of Cambridge, Cambridge, United Kingdom; ^2^Department of Zoology, University of Cambridge, Cambridge, United Kingdom; ^3^School of Biochemistry, Biomedical Sciences Building, University of Bristol, University Walk, Bristol, United Kingdom; ^4^Marine Biological Association, The Laboratory, Citadel Hill, Plymouth, Devon, United Kingdom; ^5^Howard Hughes Medical Institute and Department of Molecular and Cell Biology, University of California, Berkeley, CA; ^6^CNRS, UMR 7144, Station Biologique de Roscoff, Place Georges Teissier, Roscoff, France; ^7^Sorbonne Universités, Université Pierre et Marie Curie (UPMC) Paris 06, UMR 7144, Station Biologique de Roscoff, Place Georges Teissier, Roscoff, France

**Keywords:** silicon, eukaryotes, SIT, Lsi2, convergent evolution, transporter.

## Abstract

Biosilicification (the formation of biological structures from silica) occurs in diverse eukaryotic lineages, plays a major role in global biogeochemical cycles, and has significant biotechnological applications. Silicon (Si) uptake is crucial for biosilicification, yet the evolutionary history of the transporters involved remains poorly known. Recent evidence suggests that the SIT family of Si transporters, initially identified in diatoms, may be widely distributed, with an extended family of related transporters (SIT-Ls) present in some nonsilicified organisms. Here, we identify SITs and SIT-Ls in a range of eukaryotes, including major silicified lineages (radiolarians and chrysophytes) and also bacterial SIT-Ls. Our evidence suggests that the symmetrical 10-transmembrane-domain SIT structure has independently evolved multiple times via duplication and fusion of 5-transmembrane-domain SIT-Ls. We also identify a second gene family, similar to the active Si transporter Lsi2, that is broadly distributed amongst siliceous and nonsiliceous eukaryotes. Our analyses resolve a distinct group of Lsi2-like genes, including plant and diatom Si-responsive genes, and sequences unique to siliceous sponges and choanoflagellates. The SIT/SIT-L and Lsi2 transporter families likely contribute to biosilicification in diverse lineages, indicating an ancient role for Si transport in eukaryotes. We propose that these Si transporters may have arisen initially to prevent Si toxicity in the high Si Precambrian oceans, with subsequent biologically induced reductions in Si concentrations of Phanerozoic seas leading to widespread losses of SIT, SIT-L, and Lsi2-like genes in diverse lineages. Thus, the origin and diversification of two independent Si transporter families both drove and were driven by ancient ocean Si levels.

## Introduction

**Fig. 1:**
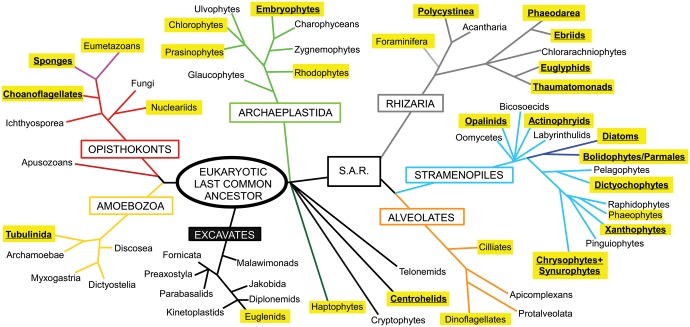
Si biomineralization across the Eukaryotes. The eukaryotic phylogeny is based on Adl et al. (2012). Major eukaryotic supergroups are named in boxes. Those taxa with highlighted names contain one or more biosilicifying species. Taxa with widespread biosilicification and extensively silicified lineages are in bold and underlined.

The involvement of silicon (Si) in biology is a widespread but relatively poorly understood phenomenon. The most prominent role for Si in eukaryotes is in the formation of biomineralized cellular structures. Silica is the most taxonomically diverse biomineral (Knoll and Kotrc 2015), occurring in all main eukaryotic supergroups (fig. 1). Indeed, given that Si is the second most abundant element in the Earth’s crust, it is perhaps surprising that biosilicification is not a more prominent feature of life.

Major silicified lineages are found in the stramenopiles (diatoms, chrysophytes/synurophytes, dictyochophytes, etc.), rhizarians (phaeodarians, polycystinean radiolarians, etc.), opisthokonts (sponges and loricate choanoflagellates), testate amoebae, and land plants (fig. 1). These organisms use silica extensively to form macrostructures such as protective cell coverings although less prominent silicified structures are also present in many other lineages (e.g., copepod mouthparts). Recent evidence suggests that Si may also contribute to other forms of biomineralization, such as coccolithophore calcification (Durak et al. 2016) or vertebrate bone formation (Henstock et al. 2015). In addition, Si may play a metabolic role within the cell although this is less well defined (Ye et al. 2013). Si utilization is not limited to eukaryotes. Baines et al. (2012) recently identified that the massively abundant marine picocyanobacteria, *Synechococcus*, was capable of accumulating significant quantities of Si. Examination of environmental samples demonstrated that *Synechococcus* can exhibit Si:P ratios comparable to diatoms although the intracellular form of this Si and its role within the cell are not known (Baines et al. 2012).

The extensive use of Si for biomineralization by many ecologically important organisms in both marine (e.g., diatoms and radiolarians) and terrestrial environments (e.g., grasses) is a central driver of the global Si cycle. Interactions between silicifying organisms and ocean Si geochemistry have driven variations in the Si cycle and biosilicification over geological time (Van Cappellen 2003; Finkel and Kotrc 2010). As many siliceous organisms are central to ecosystem functioning, the availability of Si for biosilicification can have an important influence on other biogeochemical cycles, such as fixation and cycling of atmospheric carbon by marine diatoms (Van Cappellen 2003; Street-Perrott and Barker 2008). Biosilicification is also important due to its potential biotechnological applications, including the production of complex nanopatterned structures, drug delivery vehicles, and bioactive glass implants (Sumper and Kroger 2004; Henstock et al. 2015).

All biosilicifying organisms require a mechanism to enable uptake of Si (primarily in the form of silicic acid, Si(OH)_4_) from the environment, across the plasma membrane and into the cell. Si is then concentrated within intracellular compartments, termed Silica Deposition Vesicles (Martin-Jézéquel et al. 2000), where controlled amorphous silica formation can occur. This process requires specific Si-interacting transmembrane proteins that can transport Si(OH)_4_ without inducing silica polymerization.

The Silicon Transporters (SITs) were the first such Si transporting proteins identified, initially from diatoms (Hildebrand et al. 1997) and later chrysophytes (Likhoshway et al. 2006). SITs are sodium-coupled active transporters with specific silicic acid uptake activity. Diatoms possess multiple SITs that display different patterns of gene expression, which may relate to different roles in silicification (Thamatrakoln et al. 2006). SITs have 10 transmembrane domains (TMDs), and a transport model was proposed centred around repeated EGXQ and GRQ motif pairs at TMD2 + 3, and TMD7 + 8 (Thamatrakoln et al. 2006). SITs have not been found in siliceous plants or sponges although they were identified in loricate choanoflagellates (Marron et al. 2013). The isolated occurrence of SITs in distantly related eukaryotic groups led to the hypothesis that they evolved by horizontal gene transfer (HGT).

Other proteins, unrelated to SITs, have been proposed to play a role in Si transport in sponges (Schröder et al. 2004), and recent studies have characterized several mammalian aquaporins that can act as Si(OH)_4_ channels (Garneau et al. 2015). Embryophytes (land plants) also possess a system of major intrinsic proteins that act as Si(OH)_4_ influx channels: Lsi1, EaNIP3, etc. (Grégoire et al. 2012; Ma and Yamaji 2015). These protein channels are passive transporters and therefore cannot concentrate Si in the cytosol or intracellular compartments against an Si(OH)_4_ gradient. Plant cells overcome this constraint using the polarized distribution of the active Si effluxer, Lsi2, to enable the flux of Si across the root into the vascular system. Lsi2 proteins are H^+ ^antiporters with significant similarity to the ArsB prokaryotic arsenic transporters (Ma et al. 2007). A gene with similarity to Lsi2 in the diatom *Thalassiosira pseudonana* showed a transcriptional pattern characteristic of silicification-related genes, suggesting that this family of transporters may play a role in Si transport in both plants and diatoms (Shrestha et al. 2012).

The occurrences in taxonomically isolated lineages and lack of homology between Si transporters suggested that they evolved independently, or via HGT (Marron et al. 2013). However, SIT and Lsi2 genes were recently identified in siliceous haptophytes (Durak et al. 2016), indicating that these gene families exhibit a much broader distribution in eukaryotes. Additionally, an expanded family of SIT-like (SIT-L) genes was identified in nonsiliceous haptophytes and other eukaryotes (Durak et al. 2016). Coccolithophore species with SIT-Ls showed sensitivity to the Si analogue germanium, suggesting that SIT-Ls may play a role in Si uptake in these organisms. SIT-Ls resemble “semi-SITs”, with five TMDs and one EGXQ-GRQ motif pair at TMD2 + 3. This suggests that the SIT family of Si transporters is much more diverse and widely distributed than previously thought, raising important questions: Did SITs evolve from SIT-Ls? Why are SITs and SIT-Ls found in such a diverse range of lineages, including nonsiliceous species? Why are SITs and SIT-Ls absent from an equally diverse range of silicifying organisms? Is the same true for other Si transporters, such as Lsi2, pointing toward a wider role for Si in eukaryotes?

We have conducted a detailed phylogenetic analysis of SITs and Lsi2 to examine their distribution and evolutionary history. We report the identification of new groups of SIT and SIT-L sequences and identify that this family of transporters are present in major silicified lineages such as polycystinean radiolarians, phaeodarians, and chrysophytes/synurophytes. The SIT gene family has a deep and ancient origin within eukaryotes, and there have been multiple subsequent losses, duplications, and subfunctionalizations. We also identify a widely distributed class of eukaryotic Lsi2-like genes. These Lsi2-like genes are prevalent in many major silicifying lineages, including the siliceous sponges. We propose an evolutionary model where selective pressure from environmental Si concentrations has led to the parallel evolution of Si transport mechanisms.

## Results

### Identification of SITs and SIT-Ls

Using *Stephanoeca diplocostata* SITa (Marron et al. 2013) as a query sequence, BLAST searches were performed in multiple data sets, encompassing diverse eukaryotes for which little or no sequence information was previously available (see Materials and Methods and supplementary table S1, Supplementary Material online). Diatoms were excluded from our searches due to the abundant information existing on SITs in this group (Thamatrakoln et al. 2006). Matching sequences are listed in supplementary table S2, Supplementary Material online.

The procedures involved in sequencing transcriptomes of novel eukaryotes can introduce contamination. Foreign organisms may be present in the cultures, due to the difficulty in purifying axenic cultures or prey requirements of heterotrophic species. Misreading of indexes on the Illumina Sequencing platform is a further potential source of cross-contamination (Kircher et al. 2012). These problems are a major factor to consider when searching for novel highly expressed genes (such as SITs) in de novo assemblies of diverse, poorly characterized species, as in the Moore Microbial Transcriptome Sequencing Project (MMETSP) data sets (Keeling et al. 2014).

We performed a decontamination step on the MMETSP assemblies to remove potential cross-contamination events (see Materials and Methods). We also conducted phylogenetic analyses of housekeeping genes (Hsp90, psaA, and EF-1) in all MMETSP transcriptomes found to contain SITs or SIT-Ls (see Materials and Methods, supplementary figures S2 and S3, Supplementary Material online, supplementary table S3, Supplementary Material online). If these housekeeping gene sequences branched distinct from the expected species and with another eukaryotic group, then the parent transcriptome was not analysed further due to the likelihood of the SIT or SIT-L sequences being contamination derived. In addition, SIT sequences were removed from further analysis if they branched with very high support and short branches deep among known SIT groups from unrelated organisms (e.g., diatoms and choanoflagellates). It should be noted that these potential contaminant sequences could represent examples of very recent species-specific HGT although we consider this unlikely in the majority of cases and this scenario was not explored further. Sequences likely to be contaminants are noted in supplementary table S2, Supplementary Material online.

### SITs and SIT-Ls Are Found in Diverse Eukaryotic Lineages

**Fig. 2:**
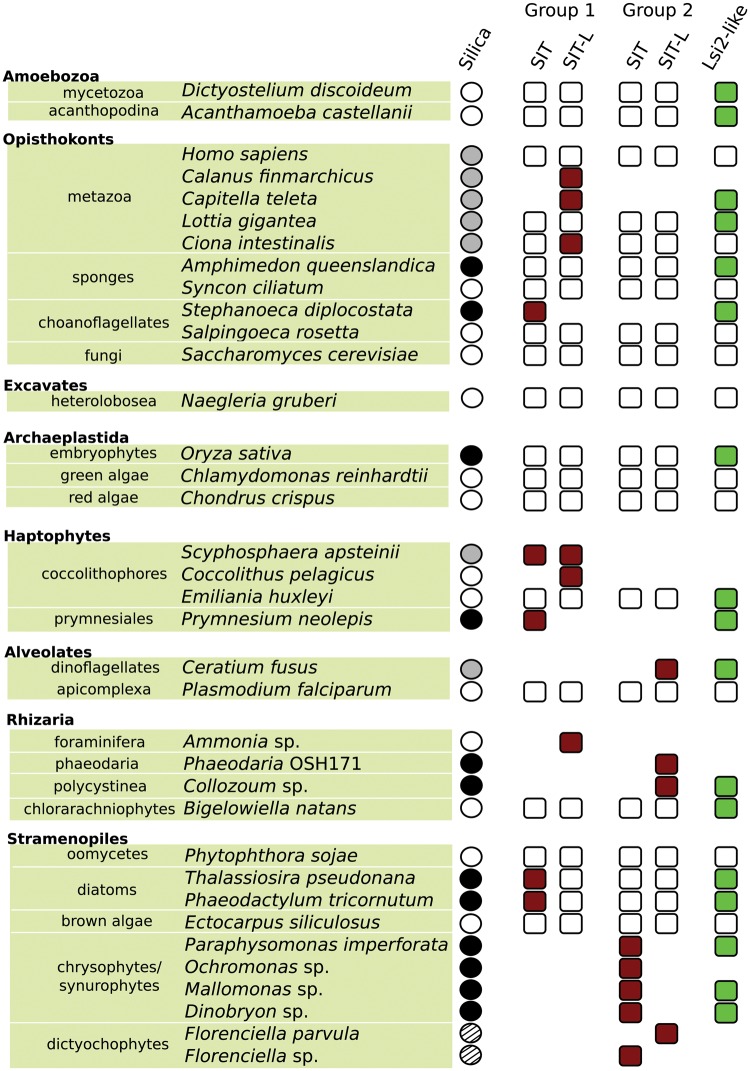
Summary table of SIT, SIT-L, and Lsi2-like genes detected in selected species. Si transporters are widely distributed across the eukaryotic supergroups and are found in species that are extensively silicified (black circle), partially silicified (gray circle), and nonsiliceous (white circle). Hatched circles signify species where no biosilica has been reported but where the wider class is extensively silicified. A filled square indicates that the gene was detected (dark red for SIT family, light green for Lsi2-like). The *Florenciella* sp. SIT and *Collozoum* SIT-L were classified on the basis of alignments and preliminary phylogenies (see Materials and Methods). White squares indicate absence from fully sequence genomes; blank space denotes that the relevant gene was not detectable but only transcriptomic data was available. Taxonomic classifications and degrees of silicification match those from fig. 1.

Full SIT sequences, defined as having 10 TMDs and 2 EGXQ-GRQ motif pairs, were identified in diatoms, choanoflagellates and some haptophytes, as previously noted (Hildebrand et al. 1997; Marron et al. 2013; Durak et al. 2016). Furthermore, we also identified 10-TMD SITs in multiple stramenopiles (fig. 2), including the siliceous chrysophyte/synurophytes *Paraphysomonas*, *Ochromonas*, and *Dinobryon*. These SITs showed little similarity to SITs previously reported from other chrysophytes (Likhoshway et al. 2006). No SITs (nor SIT-Ls) were identified in the transcriptomes of nonsiliceous stramenopiles (e.g., *Mesopedinella arctica*, *Vaucheria litorea*, and *Chattonella subsalsa*). Additional searches of nonsilicified stramenopile species with sequenced genomes (e.g., *Ectocarpus siliculosus, Phytophthora sojae*, and *Aureococcus anophagefferens*) confirmed a lack of both SITs and SIT-Ls (fig. 2). The only exception was an SIT in *Florenciella* sp. (fig. 2). This is an apparently nonsiliceous stramenopile genus (Eikrem et al. 2004) although the biology of the Florenciellales family is poorly characterized and they belong within the extensively silicified dictyochophyte lineage (the “silicoflagellates”).

In contrast, SIT-L sequences, characterized as having five TMDs and one EGXQ-GRQ motif pair (Durak et al. 2016), were found in a wide variety of siliceous and nonsiliceous eukaryotic lineages (fig. 2, also see supplementary table S2, Supplementary Material online). An SIT-L, distinct from the SIT of *Florenciella* sp., is present in the transcriptome of the reportedly nonsiliceous dictyochophyte *Florenciella parvula* (see above). The siliceous stramenopile *Mallomonas* also possesses a one EGXQ-GRQ motif pair sequence; however, alignments and TMD predictions provide very strong evidence that rather than being an SIT-L it in fact represents an incomplete SIT sequence with four TMDs of the N-terminal portion truncated in assembly. Importantly, SIT-Ls are also present in the siliceous rhizarians: *Collozoum* (a polycystine radiolarian) and *Phaeodaria* (a silicified cercozoan). Very short sequences bearing significant similarity to SITs or SIT-L were identified in a further polycystine radiolarian (*Spongosphaera streptacantha*). No SIT-L sequences were identified in transcriptomes generated from nonsiliceous radiolarians, including the strontium sulphate biomineralizing acantharian *Amphilonche elongate*. SIT-Ls were not detected in any diatom or choanoflagellate transcriptomes (fig. 2).

SIT-Ls were also found in organisms that are not extensively silicified. Interestingly, the metazoan groups that possess SIT-Ls all produce minor silicified structures (fig. 2). The tunicates have silicified ovarian granules (Monniot et al. 1992), calanoid copepods (*Calanus finmarchicus*) have silica-hardened mouthparts (Karlson and Bamstedt 1994), and polychaete worms (*Capitella teleta* and *Platynereis dumerilii*) may possess mineralized chaetae (Schroeder 1984). SIT-Ls were also present in several genera (*Ceratium*, *Dinophysis*, and *Noctiluca*) of dinoflagellates, a group that forms silicified cysts (Preisig 1994). We also identified SIT-Ls in calcifying organisms; three calcifying foraminifera and the calcifying coccolithophores, *Calcidiscus leptoporus*, *Coccolithus pelagicus*, and *Scyphosphaera apsteinii* (Durak et al. 2016). Both of these calcified lineages have closely related silicifying lineages, namely silicoforams (Sen Gupta 2003) and the silicifying haptophyte, *Prymnesium neolepis* (Yoshida et al. 2006).

### SIT-Ls Are Found in Bacteria

Outside of the eukaryotes, sequences with similarity to SITs were found in a limited range of bacterial genomes. Two strains of the cyanobacterium *Synechococcus* possessed SIT-L genes, containing one EGXQ-GRQ motif pair and five TMDs. Similar SIT-L genes were found in the genome of two strains of the actinobacterium *Rhodococcus opacus*. The identification of SIT-Ls in *Synechococcus* is noteworthy, as this species can accumulate large quantities of Si. We also examined evidence for their wider existence and utilization in the marine environment using metagenomic data sets. Two contigs exhibiting high similarity to cyanobacterial SIT-Ls were present in the prokaryotic size fractions (0.22–3 µm) of the *Tara* Oceans metaG environmental metagenomic data sets (Sunagawa et al. 2015). Sequences exhibiting strong similarity to eukaryotic SITs or SIT-Ls were also found in *Tara* Oceans metaG data (supplementary table S2, Supplementary Material online).

### Generalized Structure of SITs and SIT-Ls

**Fig. 3:**
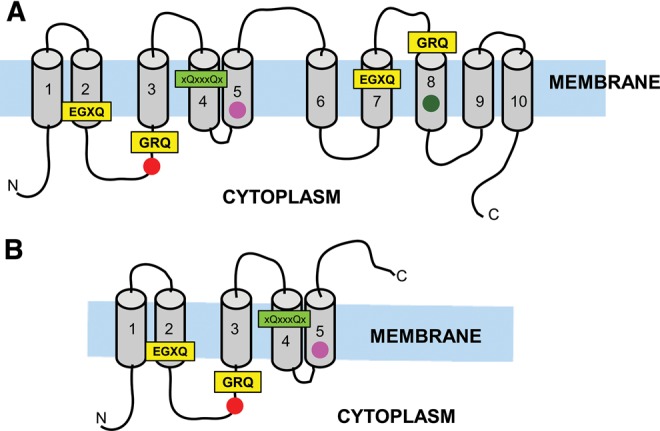
A generalized structure for SITs and SIT-Ls. (*A*) SIT structure schematic. (*B*) SIT-L structure schematic. Transmembrane helixes (gray) are based on predictions from TMPred. Conserved motifs (boxes, proposed binding sites highlighted in yellow, other conserved motif in bright green) were determined from the alignment in supplementary figure S1, Supplementary Material online. Circles are conserved residues: hydroxylated residues = dark green, positive residues= bright red, negative residues = magenta.

Protein sequence alignments indicate that the Silicon Transporter gene family divides between SITs (10 TMDs and 2 EGXQ-GRQ motif pairs) and SIT-Ls (5 TMDs and 1 EGXQ-GRQ motif pair) (fig. 3 and supplementary figure S1, Supplementary Material online). For both SITs and SIT-Ls, the N terminus is predicted to be inward-facing in the plasma membrane. In both SITs and SIT-Ls, the first conserved EGXQ motif is found before the intracellular face of TMD2 and the first conserved GRQ motif is at the intracellular face of TMD3. In SITs, the second pair of conserved EGXQ and GRQ motifs is found at the extracellular face of TMD7 and TMD8, respectively. The organization of the TMDs and the conserved motifs confirm the pseudosymmetry of the SIT protein (Thamatrakoln et al. 2006; Marron et al. 2013), with SIT-Ls resembling half of this pseudosymmetrical structure (fig. 3).

The GRQ motifs are highly conserved, with only two variants identified: GRH in *Diaphanoeca grandis* SITβ, which was confirmed by cloning and re-sequencing, and GQS in *Paraphysomonas* SIT2. EGXQ motifs show more variability, with minor variations (A substituted for G; K/M/H substituted for Q) in some stramenopiles (e.g., *Paraphysomonas* SIT2), tunicates, rhizarians, and *Rhodococcus opacus.*

Inspection of the multiple sequence alignment indicates that a positively charged residue (arginine/lysine) between TMD2 and TMD3 and a negatively charged residue (glutamate) in TMD5 are conserved among eukaryotic SITs and SIT-Ls (Marron et al. 2013). The hydroxylated residue (threonine) in TMD8 is also widely conserved among SITs. In addition, a motif in TMD4 (xQxxxQx) is broadly conserved between SITs and SIT-Ls (fig. 3).

### Choanoflagellate SITs Display Functional Diversification

Transcriptomic analysis identified two SITs in all siliceous choanoflagellate species examined (except *S.diplocostata*, which has three). No SITs or SIT-Ls were found in any nonsiliceous choanoflagellate species (see supplementary table S1, Supplementary Material online). Phylogenetic analysis of choanoflagellate SITs resolves two clades, SITα and SITβ (see supplementary figure S4, Supplementary Material online), having a branching pattern broadly corresponding to the species phylogeny and the nudiform/tectiform division (Nitsche et al. 2011).

Transcriptome read mapping counts indicate that SITα expression is much higher than SITβ expression across all species. A more detailed examination was conducted using reverse transcription quantitative PCR (RT-qPCR) of *D. grandis* and *S. diplocostata* cultures grown in high (>240 µM), low (<5 µM), and control (128 µM) Si seawater media. In *D. grandis*, SITα was significantly upregulated in low-Si compared with control-Si treatments (supplementary figure S5A, Supplementary Material online) and significantly downregulated in high-Si versus control-Si conditions (supplementary figure S5B, Supplementary Material online). DgSITβ expression, although present, was consistently too low across Si treatments to reliably take sufficient measurements for statistical analysis. SITα expression was orders of magnitude higher than SITβ expression in *S. diplocostata* under all Si conditions (supplementary figure S5C, Supplementary Material online). The two SITα genes, SdSITαA and SdSITαBC (see Materials and Methods), displayed different responses to Si treatments. Both SITα genes were downregulated in high-Si compared with control-Si conditions (supplementary figure S5D, Supplementary Material online). However, only SdSITαA was significantly upregulated under low-Si compared with control-Si treatments (supplementary figure S5E, Supplementary Material online). The SdSITβ gene expression measurements showed no significant transcriptional response to Si treatments (supplementary figure S5D–E, Supplementary Material online). SITα gene expression is higher and Si-responsive, whereas SITβ genes are expressed at low levels, unaffected by Si concentration.

### Phylogenetic Analysis of SITs and SIT-Ls

**Fig. 4:**
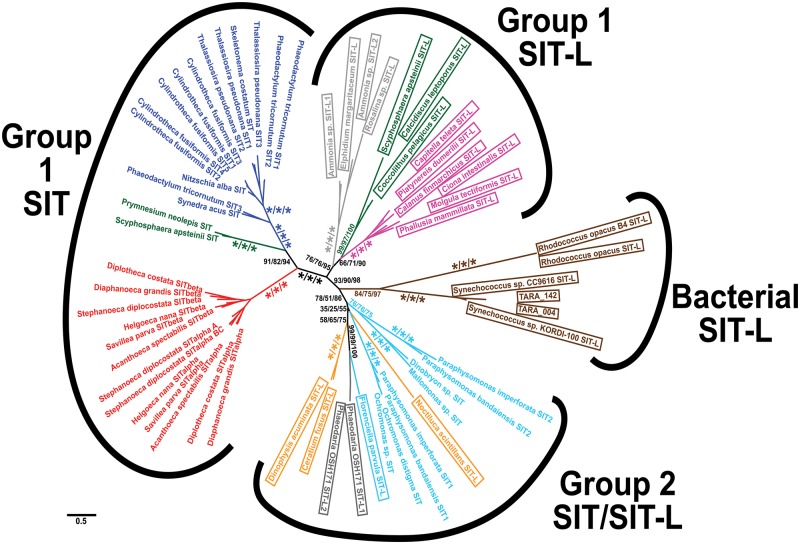
Phylogenetic tree of SIT and SIT-L sequences. Bacterial SIT-Ls form a distinct clade, and eukaryotic SITs can be divided into two main groups. Group 1 SITs and Group 1 SIT-Ls are connected by a branch with good support (93/90/98), whereas the basal branching order of Group 2 has poor statistical support. SITs and SIT-Ls largely follow the species phylogeny, with the exception of the paraphyletic stramenopile, dinoflagellate, and rhizarian sequences in Group 2. Brown = bacteria, dark green = haptophyte, gray = rhizarian (light gray = foraminiferan), Bright red = choanoflagellate, magenta = metazoan, orange = dinoflagellate, dark blue = diatoms, light blue = other stramenopiles. SIT-L sequences are in boxes. This unrooted radial tree is based on the RaxML maximum likelihood analysis with the best-fitting LG + G4 model from an alignment of 485 amino acid residues. Numbers at nodes are a percentage of 100 bootstrap or 1000 ultrafast bootstrap replicates in the format RaxML/PhyML/IQ-TREE support value, with */*/* signifying nodes with 100% support for all methods (for clarity only support values for major nodes are shown, see supplementary figure S6, Supplementary Material online for full trees). Scale bar indicates average number of amino acid substitutions per site.

The evolutionary origin of SITs and SIT-Ls was explored by phylogenetic analysis (fig. 4; supplementary figure S6, Supplementary Material online). Bacterial SIT-Ls, plus several environmental sequences that are likely bacterial in origin, branch together to form a group distinct from the eukaryotic sequences. These sequences may represent a true sister group at the root of the tree, indicating that eukaryotic Silicon Transporters evolved from a prokaryotic SIT-L ancestor. This would imply that some of the diversity of bacterial SIT-Ls has been lost or remains to be discovered. Alternatively, the bacterial SIT-Ls may have originated from a more recent eukaryote-to-prokaryote HGT although presently there is no obvious candidate for the eukaryotic source of this transfer event.

The eukaryotic sequences fall into three main groups: Group 1 SITs, Group 1 SIT-Ls, and Group 2 (which contains both SITs and SIT-Ls). The full SITs (10 TMDs and 2 EGXQ-GRQ motif pairs) found in diatoms, choanoflagellates, and haptophytes form a well-supported monophyletic clade (the Group 1 SITs). The branching order within Group 1 SITs largely reflects the species-level relationships, with choanoflagellates representing the outgroup to diatoms and haptophytes. The Group 1 SIT-L sequences belong to haptophytes, metazoans, and foraminiferans (Rhizaria). There is good statistical support (>90% bootstrap values, posterior probability = 1, see supplementary figure S6, Supplementary Material online) for the node connecting the Group 1 SIT and Group 1 SIT-L branches.

The remaining SIT and SIT-L sequences (i.e., those found in non-diatom stramenopiles, alveolates, and “radiolarian” rhizarians) form a group with a poorly resolved basal branching order. We have termed them Group 2 SITs/SIT-Ls to distinguish them from the Group 1 branches in our subsequent discussion. Neither Group 2 SITs nor Group 2 SIT-Ls are monophyletic with respect to each other, with the dinoflagellate and rhizarian SIT-L sequences being paraphyletic with respect to the stramenopile SITs and the SIT-L from *Florenciella parvula*.

### Phylogenetic Analysis of SIT-Ls and SIT N/C-Terminal Halves

**Fig. 5:**
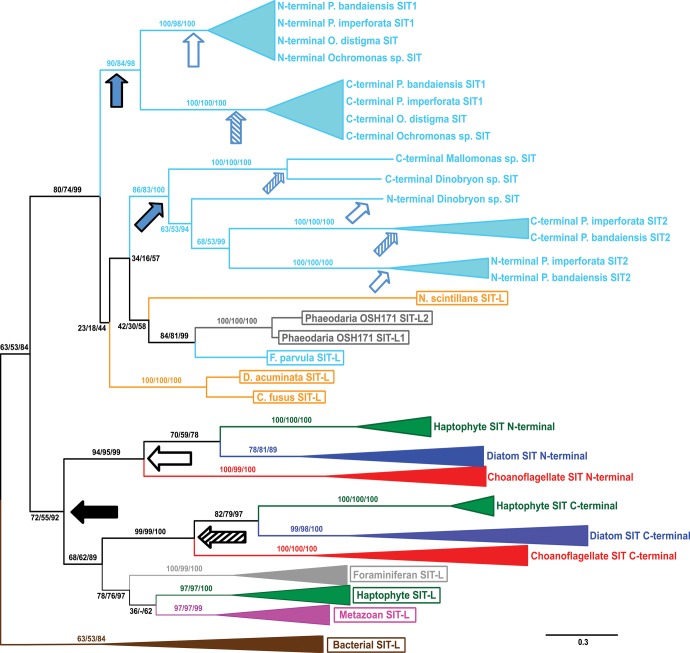
Phylogenetic tree of SIT N-terminal halves, SIT C-terminal halves and SIT-Ls. Phylogenetic analysis of halved SITs and SIT-Ls reflects that of the full-length sequences (fig. 4). The N-terminal and C-terminal halves of the Group 1 SITs (diatom, haptophyte, and choanoflagellate) are, respectively, most related to each other. In contrast, the N- and C-terminal halves of the nondiatom stramenopiles SITs of Group 2 are more closely related to the other half of the same gene. This indicates that these SITs arose by independent duplication, and this may have even happened multiple times in the chrysophytes/synurophytes. Arrows show different inferred duplication-inversion-fusion events, with solid arrows indicating a duplication event, open arrows the resulting N-terminal halves and hatched arrows the resulting C-terminal halves. The various arrow angles denote individual inferred SIT-producing events; arrows of the same angle are inferred to be part of the same event. Brown = bacteria, dark green = haptophyte, gray = rhizarian (light gray = foraminiferan), bright red = choanoflagellate, magenta = metazoan, orange = dinoflagellate, dark blue = diatom, light blue = other stramenopiles. Tree based on RaxML maximum likelihood analysis with the best-fitting LG + G4 model from an alignment of 166 amino acid residues. Note that the tree topology is that of an unrooted tree; the bacterial SIT-L clade was arbitrarily designated as an outgroup for presentation purposes. Numbers at nodes are a percentage of 100 bootstrap or 1000 ultrafast bootstrap replicates in the format RaxML/PhyML/IQ-TREE value. Scale bars indicate average number of amino acid substitutions per site. Full phylogenetic trees are given in supplementary figure S7, Supplementary Material online.

SIT sequence structure resembles that of two SIT-Ls that have fused into a 10-TMD, 2 EGXQ-GRQ motif pair gene (fig. 3). To investigate the origins of this duplication-fusion, we phylogenetically analyzed SIT-Ls together with SIT sequences artificially split into two 5-TMD “halves” (fig. 5 and supplementary figure S7, Supplementary Material online).

This analysis finds that the N- and C-terminal halves of the Group 1 SITs form independent monophyletic groups. Within each N- and C-terminal clade the diatom, choanoflagellate, and haptophyte sequences form monophyletic subgroups and preserve a branching order largely reflecting the species phylogenies and full-SIT tree (fig. 4). The Group 1 SIT-Ls were also recovered together, as in the full-SIT tree (fig. 4).

N- and C-terminal halves of the Group 2 SITs branch paraphyletically among the Group 2 SIT-Ls and are separated from the Group 1 SIT terminal halves. Unlike Group 1 SITs, the N- and C-terminal halves of Group 2 SITs do not resolve into two independent clades. The N- and C-terminal halves of the *Ochromonas* SITs and *Paraphysomonas* SIT1 branch together in a monophyletic group and are more closely related to each other than to any other SIT terminals or SIT-Ls. Further SIT sequences (*Paraphysomonas* SIT2 and *Dinobryon*) within the Group 2 exhibit a similar pattern, with the two halves appearing more closely related to each other than to the respective regions of other Group 2 SITs.

We note that the topology within the eukaryotic SIT-L and full SIT sequences remains unchanged in phylogenetic trees constructed after removing the group of bacterial and putatively bacterial sequences (supplementary figure S8A–E, Supplementary Material online). All tree topologies, including that of the SIT-L and SIT N/C-terminal half phylogeny, are similar when phylogenetically analyzed using software and models of sequence evolution (see Materials and Methods) designed to mitigate possible long-branch attraction effects of fast-evolving sequences (supplementary figure S8E–G, Supplementary Material online).

### Phylogeny of Lsi2-Like Sequences

**Fig. 6:**
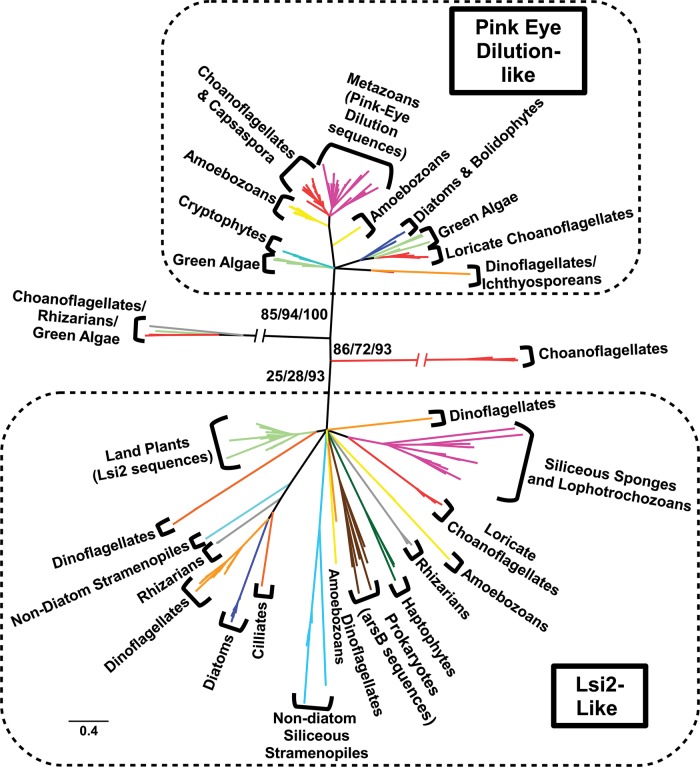
Phylogenetic tree of Lsi2-like sequences from a taxonomically diverse range of eukaryotes and prokaryotes. The sequences divide into two main groups: one containing Pink-Eyed Dilution P-protein (PED) sequences and the other containing the active Si transporter Low Si 2 (Lsi2). Among opisthokonts most metazoans and all choanoflagellates investigated had at least one PED-like sequence; conversely only loricate choanoflagellates, siliceous sponges and three lophotrochozoans (*Lottia gigantea, C. teleta*, and *Lingula anatina*) possessed Lsi2-like sequences. Plant Lsi2 sequences form a strongly supported (86% RaxML, 99% PhyML, 100% IQ-TREE bootstrap) monophyletic clade. Sequences from multiple siliceous and nonsiliceous eukaryotes branch in the Lsi2-like clade, including a *Thalassiosira pseudonana* Si-responsive gene, which falls within a well-supported (100% RaxML, 100% PhyML, 100% IQ-TREE bootstrap) diatom branch. Brown = bacteria, dark green = haptophyte, gray = rhizarian (light gray = foraminiferan), bright red = choanoflagellate, magenta = metazoan, orange = alveolate (light orange = dinoflagellate), dark blue = diatom, light blue = other stramenopiles, bright green = archaeplastids, turquoise = cryptophyte, yellow = amoebozoan (note contamination of MMETSP sequences is accounted for, see supplementary table S4, Supplementary Material online). The tree was produced using RaxML maximum likelihood analysis with the best-fitting LG + G4 model from an alignment of 247 amino acid residues. Nodes with <20% bootstrap support were collapsed to give a topology agreed across all methods (see supplementary text S1–S5, Supplementary Material online for full uncollapsed tree files). Scale bar indicates average number of amino acid substitutions per site; slashes indicate very long branches that were clipped for display purposes.

The Lsi2 antiporters are used for active Si transport in embryophytes (Ma et al. 2007; Ma and Yamaji 2015) and have recently been identified in diatoms and the siliceous haptophyte *P. neolepis*. Therefore, we examined their wider distribution in eukaryotes. Sequence similarity searches using *Oryza sativa* (rice) Lsi2 as a query identified sequences in a wide variety of eukaryotes, with Lsi2 sequences found in all land plant groups examined (fig 2; supplementary table S4, Supplementary Material online). Phylogenetic analysis resolves two main groups (fig. 6, supplementary text, Supplementary Material online), which both contain representatives from all main eukaryotic supergroups except excavates. Two very long branches (a choanoflagellate branch and a mixed-taxon branch) emerge as intermediate between the two main groups; their placement may be due to phylogenetic artefacts (e.g., long branch attraction).

The first group contains multiple vertebrate Pink-Eyed Dilution P-protein (PED) sequences, which encode an integral membrane transporter connected to transport of metalloids, including arsenic (Staleva et al. 2002). This PED-like clade includes both siliceous and non-siliceous species. The second group contains land plant Lsi2 sequences and the prokaryotic ArsB genes. Therefore, we have referred to these genes as “Lsi2-like.” Within this Lsi2-like group are sequences from many silicified organisms (fig. 2 and supplementary table S4, Supplementary Material online) such as sponges, loricate choanoflagellates, haptophytes, chrysophytes/synurophytes, radiolarians, and diatoms (including the *T. pseudonana* Si-responsive gene). Some nonsiliceous species (e.g., the amoebozoan *Acanthamoeba castellanii*) also contain Lsi2-like sequences, suggesting that its cellular role may not be restricted to biosilicification.

However, there are clear trends in the distribution of Lsi2, particularly within the opisthokonts. Within the choanoflagellates, only siliceous species have genes from the Lsi2-like group, whereas both siliceous and nonsiliceous choanoflagellates possess PED-like genes. There are a large number of Lsi2-like sequences from siliceous sponges (hexactinellids and demosponges), with each species showing an expanded repertoire of Lsi2-like genes. Sister to this sponge clade is a subgroup containing eumetazoan (lophotrochozoan) sequences. Other metazoans, including all nonsiliceous sponges examined, only possess genes branching within the PED-like group.

## Discussion

### SITs and SIT-Ls Are Widely Distributed in Siliceous Eukaryotes

Our wide-ranging analyses of genomic and transcriptomic data sets indicate that the SIT family of Si transporters, encompassing SITs and SIT-Ls, are broadly distributed amongst eukaryotes (fig. 2). This contrasts with previous suggestions that Si transporters, like biosilicification, may have arisen independently in isolated silicifying lineages (Knoll 2003). Instead, we find that in addition to the previously reported presence of SITs in diatoms, choanoflagellates, and haptophytes, SITs are also present in the dictyochophytes and synurophytes/chrysophytes. Furthermore, we find that the related SIT-L genes are present in major silicified lineages such as the polycystinean radiolarians and phaeodarians, as well as in the Si-accumulating cyanobacterium *Synechococcus*. Although functional characterization of the SIT-Ls will be required to fully determine their role in cellular Si transport, these SIT-L transporters are prime candidates to support Si uptake in these lineages. It appears likely that the expanded SIT family may therefore contribute to Si uptake in many extensively silicified eukaryote lineages. The embryophytes (land plants) and the siliceous sponges, therefore, appear to be exceptional in that they represent extensively siliceous lineages lacking SIT or SIT-L transporters.

### Eukaryotic SIT-Ls and Convergent Evolution of SITs

SITs and SIT-Ls have now been detected in every major eukaryotic supergroup (Adl et al. 2012; Burki 2014) except for Archaeplastida, Amoebozoa, and Excavata although no transcriptomic or genomic data are available from biosilicifying amoebozoan or excavate species. Based on current knowledge of evolutionary relationships between supergroups, this distribution could be explained by an ancestral origin for Silicon Transporters (both SITs and SIT-Ls) in the eukaryotic last common ancestor (LCA). This hypothesis requires that there have been widespread losses of SITs and SIT-Ls throughout the eukaryotes, as SITs and SIT-Ls are absent from the majority of sequenced eukaryote genomes. An alternative explanation for the distribution of SITs and SIT-Ls in eukaryotes is a more recent origin in a specific lineage, followed by a series of HGT events between lineages. However, there is no strong phylogenetic support for HGT and although the HGT scenario requires fewer gene loss events, it still requires extensive loss of both SITs and SIT-Ls in certain lineages. The evidence for and against these two evolutionary scenarios is outlined below.

**Fig. 7:**
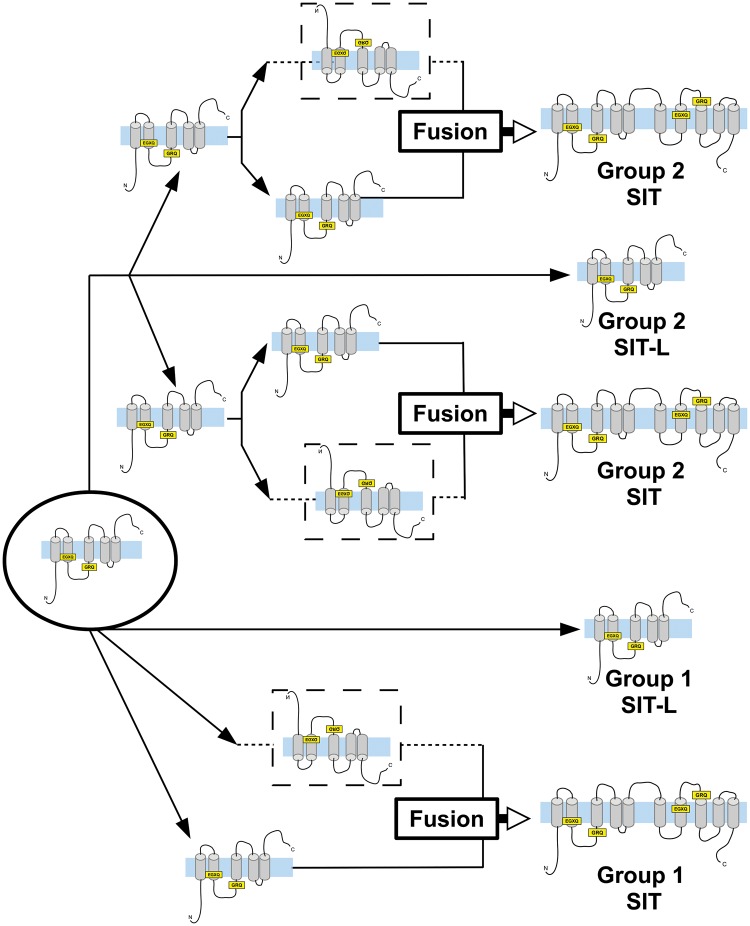
Schematic model of SIT evolution. This interpretation is based on the phylogenetic analyses presented in figs. 4 and 5. SITs likely originated as a gene encoding a 5-TMD protein (circled), and diversified into the Group 1 SITs, Group 1 SIT-Ls and Group 2 SIT/SIT-Ls. Independent duplication (black arrows), inversion (dashed lines), and fusion (white arrows) events gave rise to 10-TMD SITs in both the Group 1 SITs and in Group 2. SIT protein structure diagrams are adapted from fig. 3, with N and C terminals shown, TMDs in gray crossing the membrane (in blue). Conserved GRQ and EGXQ motifs are highlighted in yellow.

The broad distribution of Group 1 SIT-Ls in metazoans, haptophytes and rhizarians suggests an ancient origin for these transporters in the eukaryotic LCA (fig. 1). Our evidence also supports a single ancient origin for Group 1 SITs (fig. 7). In this sense, Group 1 SITs and SIT-Ls are paralogous, with Group 1 SITs likely evolving via duplication, inversion and fusion of Group 1 SIT-Ls. The branching pattern of N- and C-terminal halves of Group 1 SITs provides strong correlation with evolution from a single common ancestor (fig. 5). As Group 1 SITs are found in stramenopiles, haptophytes, and choanoflagellates, this duplication/fusion likely represents an ancient event that occurred before the divergence of these lineages. Thus, the distribution of the Group 1 SITs, like the Group 1 SIT-Ls, is suggestive of an origin in the eukaryotic LCA followed by dramatic and widespread losses of SIT-Ls and/or SITs in many eukaryote lineages. In densely sampled groups with numerous fully sequenced genomes, there is substantial evidence to support extensive gene loss. Metazoan SIT-Ls are found in the three main bilaterian groups and form a strongly supported monophyletic clade (fig. 4). The most reasonable explanation is that Group 1 SIT-Ls were at least present in the bilaterian LCA (Dunn et al. 2008) and have been independently lost in many animal lineages.

An alternative evolutionary scenario to explain the limited distribution of SITs and SIT-Ls in eukaryotes is a series of HGT events between isolated lineages. Our phylogenetic trees do not provide support for recent HGT, as Group 1 SITs and SIT-Ls in each lineage form strongly supported monophyletic clades and would require many, highly specific, intralineage transfer events. However, we cannot rule out a contribution from ancient HGT or endosymbiotic gene transfer (EGT). For example, if we imagine that Group 1 SITs arose in the stramenopile lineage, then only two HGT events would be required to explain their presence in haptophytes and choanoflagellates. There are abundant examples of HGT from algal genomes into choanoflagellates and this was previously suggested to be the mechanism by which the loricate choanoflagellates acquired SITs (Marron et al. 2013). However, if choanoflagellate SITs did originate by HGT, the transfer event must be ancient. The Group 1 SITs are found in both major lineages of the loricate choanoflagellates, Stephanoecidae and Acanthoecidae (Nitsche et al. 2011). Their phylogeny mirrors the known species relationships among these two groups, which likely diverged soon after the choanoflagellates split from Metazoa and is estimated at 900 Ma (Nitsche et al. 2011; Parfrey et al. 2011). Moreover, the branching pattern of Group 1 SITs provides no support for HGT from stramenopiles or haptophytes into choanoflagellates, as choanoflagellate SITs branch independently from SITs present in either of these lineages. As the LCA of haptophytes and stramenopiles is proposed to be close to the eukaryotic LCA (fig. 1), the phylogenetic signal of HGT has either been obscured or the phylogeny reflects an ancestral origin of Group 1 SITs.

Recent evidence suggests that haptophytes may have acquired their plastid from a stramenopile ancestor (Stiller et al. 2014). Although the origin of the haptophyte plastid remains to be fully determined, EGT associated with this proposed event does provide a potential mechanism through which haptophytes may have acquired Group 1 SITs. As the timing of plastid acquisition must predate the divergence of the major haptophyte lineages (>500 Ma), the potential acquisition of Group 1 SITs by haptophytes by EGT is restricted to the Precambrian (Liu et al. 2010). Group 1 SITs are found in the Prymnesiales and the Coccolithales, indicating that even in the absence of EGT, acquisition of SITs by a separate HGT event would need to predate their divergence (*c.*300 Ma, Liu et al. 2010). Alternative HGT scenarios involving an origin of Group 1 SITs in choanoflagellates or haptophytes have similar requirements for the timing of the HGT events, suggesting that if HGT is responsible for their distribution, it is likely to be ancient. Although we cannot definitively rule out further complex evolutionary scenarios involving extensive recent intralineage HGT events, these appear to be less likely.

In summary, based on the current evidence, the taxonomic distribution of the Group 1 SITs and Group 1 SIT-Ls can be reasonably explained either by an origin in the eukaryotic LCA or by a series of ancient HGT/EGT events. Based on the consistent monophyletic branching relationships within the Group 1 SITs and Group 1 SIT-Ls (figs. 4 and 5), we consider it more likely that Group 1 SITs evolved by vertical inheritance from a shared ancestor (most probably the eukaryotic LCA). Although we favor an ancestral origin for Group 1 SITs and SIT-Ls, it should be noted that neither hypothesis can be completely discounted at present and resolving this will require further sampling from key groups, e.g., silicifying amoebozoans (Lahr et al. 2013).

However, it is also important to note that both scenarios require extensive loss of SITs and SIT-Ls: even if some taxa acquired Group 1 SIT-Ls or SITs by HGT/EGT (and not by vertical inheritance), there is still strong evidence for substantial losses in many lineages. In addition to the inferred losses of Group 1 SIT-Ls in the well-sampled bilaterians, the monophyly of haptophyte SIT and SIT-L sequences is evidence for their extensive loss throughout this group, as SITs and SIT-Ls are absent from the genomes of the coccolithophore *Emiliania huxleyi* and the prymnesiale *Chrysochromulina tobin*. The distribution of SIT and SIT-L sequences from both Group 1 and Group 2 across the ochrophyte stramenopiles (figs. 1 and 2) also implies that they were present ancestrally with widespread losses in various stramenopile groups, not only in nonsilicifying lineages (e.g., the pelagophyte *Aureococcus anophagefferens*) but also in silicified taxa such as diatoms, which lack Group 2–type sequences.

In contrast to Group 1 SIT-Ls, Group 2 SIT-Ls are only found in members of the SAR supergroup (stramenopiles, alveolates, and rhizarians). Their distribution suggests that Group 2 SIT-Ls were at least present in the SAR ancestor, which in turn suggests that there has been extensive loss throughout the SAR supergroup (with phylogenetic analysis raising the possibility that Group 2 SIT-Ls may have an even earlier origin if the bacterial SIT-L clade represents the ancestral sister group to the eukaryotic sequences). An alternative scenario involving a later evolution of Group 2 SIT-Ls followed by HGT between SAR lineages is also possible, but there is limited evidence for this from our phylogenetic analyses and improved taxonomic sampling will be required to fully address this hypothesis. We hypothesize that Group 2 SIT-Ls arose following the duplication of an ancestral SIT-L (fig. 7) and that the Group 1 and Group 2 genes represent ancient paralogues. It is interesting that taxa that express Group 1 SIT-Ls are primarily calcareous with minor occurrences of biosilica (Knoll and Kotrc 2015). In contrast, taxa possessing Group 2 SIT-Ls produce heavily silicified structures like scales, skeletons, and cysts (Preisig 1994). *Florenciella* is notable in retaining SIT and SIT-L genes (see Materials and Methods), and although silicification is not reported in this genus (Eikrem 2004), our analyses would suggest that it has some degree of Si-related metabolism.

The Group 2 SITs are all from nondiatom stramenopile classes that display extensive silicification. Phylogenetic analysis indicates that these full SITs have likely originated multiple times, independently from the Group 1 SITs, in a remarkable case of parallel evolution. The sister relationships of the N- and C-terminal halves of the different Group 2 SITs are consistently well supported. This implies that these SITs evolved by independent SIT-L duplication-inversion-fusion events even within the siliceous stramenopiles (figs. 5 and 7). That full SITs have evolved convergently in various siliceous stramenopiles points toward the possibility that SITs are superior to SIT-Ls for certain functions. It is interesting to note that on the available evidence, full SITs apparently did not arise convergently in many other groups, including within highly silicified protist groups like rhizarians (fig. 1).

Possessing both an SIT and an SIT-L is very rare; of the Silicon Transporter-containing species analyzed here, only *S. apsteinii* expresses both gene types; however, both an SIT and an SIT-L are present in closely related species within the Florenciellales family (fig. 2). This suggests that SIT or SIT-L loss could be frequent even in biosilicifying organisms and that there may be a high degree of redundancy in the basic functions of SITs and SIT-Ls. Therefore, it is interesting to find both “ancestor” and “descendant” transporters expressed in the same organism. To address questions regarding the advantages and disadvantages of each transporter and identify the selective pressures underlying their evolutionary distribution will require a full functional characterization of each type of transporter (SITs and SIT-Ls) from the divergent Groups 1 and 2.

### Functional Diversification of Group 1 SITs

A further case of parallel evolution is the independent diversification of Group 1 SITs within different lineages. Transporter diversification indicates sub- or neo-functionalization, often in response to novel environmental conditions. Diatoms show a high degree of SIT diversification (Thamatrakoln et al. 2006; Sapriel et al. 2009). In *T. pseudonana*, different SITs are expressed at different stages of the cell cycle (Thamatrakoln and Hildebrand 2007). Expression levels differ greatly, suggesting that highly expressed SITs (TpSIT1 and 2) are plasma membrane transporters, whereas the lower-expression TpSIT3 has a role in Si sensing, regulation or intracellular transport. This is supported the fact that TpSIT1 and TpSIT2 are localized to the plasma membrane, whereas TpSIT3 is diffused throughout the cell (Shrestha and Hildebrand 2015). We have found that the Group 1 SITs from loricate choanoflagellates show a similar diversity. The gene duplication event that produced the SITα and β classes occurred early in loricate choanoflagellate evolution, and their roles are likely to be conserved throughout the group. We suggest that the highly expressed SITα proteins are involved in Si(OH)_4_ uptake, whereas SITβs have a secondary role in biosilicification.

SITβ gene expression was several orders of magnitude lower than SITα genes in both species examined. The SITβ gene of *S. diplocostata* showed no regulation in response to the Si concentration of the growth medium in a similar manner to *T. pseudonana* SIT3. The choanoflagellate SITβs may therefore play a similar role to TpSIT3, acting as intracellular transporters, Si sensors or having a regulatory role during biosilicification. In choanoflagellates, there are two modes of lorica formation: nudiform (siliceous components formed after cytokinesis) and tectiform (siliceous components formed before cytokinesis) (Leadbeater 2015). Under conditions of Si starvation and replenishment, normally tectiform species can revert to the nudiform mode (Leadbeater 2015). This must require a sensing mechanism, potentially SITβ, for the cell to measure Si availability and to delay or proceed with biosilicification appropriately without disrupting cell division.

Additional evidence for choanoflagellate SIT subfunctionalization comes from SITα gene expression. In both *D. grandis* and *S. diplocostata*, SITα genes are downregulated under high-Si conditions, as expected if higher nutrient availability reduces the need for uptake transporter proteins. The single *D. grandis* SITα gene is upregulated in response to low-Si conditions, again as predicted in order to scavenge the limited Si available. *S. diplocostata* has two SITα genes that show differing expressional responses to the low-Si treatment, with only SdSITαA being upregulated. Similar diversification has also been observed for highly expressed SITs in *T. pseudonana* (Thamatrakoln and Hildebrand 2007) and *Phaeodactylum tricornutum* (Sapriel et al. 2009), further underlining the convergent evolution between independent choanoflagellate and diatom SIT repertoires.

### Silicon Transporter Structure

Protein sequence alignment supports the existing alternate access model for SIT transport (Thamatrakoln et al. 2006; Marron et al. 2013), with the EGXQ and GRQ motifs being coordinated within the central helix bundle to create a membrane-embedded aqueous binding vestibule. The high degree of conservation and consistent TMD positions of these motifs further indicates that the charged residues and glutamine side-chains provide the local polar environment and binding sites necessary for transmembrane transport. The other residues conserved between SITs and SIT-Ls (fig. 3, supplementary figure S1, Supplementary Material online) point toward their involvement in fundamental transport processes, such as in Na^+^ binding or providing a binding site for an intracellular Si-complexing molecule (Thamatrakoln and Hildebrand 2008).

The xQxxxQx motif located in the fourth TMD is also broadly conserved (see supplementary figure S1, Supplementary Material online) and as such could have a structural or oligomerization-related role (Boudker and Verdon 2010; Marron et al. 2013). Oligomeric structures as functional units are common among transporter proteins (Veenhoff et al. 2002; Xuan et al. 2013; Tao et al. 2015). Heterologous expression of SITs in yeast has found that SITs exist as homotetramers (Curnow et al. 2012), and the same may be the case for SIT-Ls. An oligomeric functional SIT-L unit may confer an equivalent Si transport capacity to that of SITs, in a situation akin to SWEET and semi-SWEET oligomers (Xuan et al. 2013; Tao et al. 2015).

### Eukaryotic Lsi2-Like Genes and Si Transport

Embryophytes differ from many other eukaryotes in that secondary active transport across the plasma membrane is energized primarily by H^+ ^rather than by Na^+ ^(Sze et al. 1999), and this fact may underlie the absence of the Na^+^-coupled SIT/SIT-Ls in this lineage. Instead, Si uptake by land plants is performed by a combination of passive channels and active Si effluxers (Ma and Yamaji 2015). The active transporter is Lsi2, an H^+ ^antiporter with sequence similarity to ArsB-class prokaryotic arsenic transporters. Rice Lsi2 also has the capacity to transport arsenic (Ma et al. 2008), indicative of a generalized metalloid transport capacity due to molecular mimicry (Bienert et al. 2007). We found that land plant Lsi2 sequences form a strongly supported monophyletic clade, with horsetail (*Equisetum giganteum*) Lsi2s being distinct and expanded compared with other plant species (see supplementary text, Supplementary Material online). This mirrors the independent evolution of horsetail and angiosperm Si influx channels (Grégoire et al. 2012). The widespread presence of Lsi2 in land plants suggests that Si transport is an ancient feature (Trembath-Reichert et al. 2015) and that active Si transport plays fundamental role in plant metabolism even in lineages where silica is not used extensively in a mechanical role.

Beyond land plants, Lsi2-like genes are found in many siliceous eukaryotes, including sponges, loricate choanoflagellates, polycystinean radiolarians, and diatoms (fig. 6). The Lsi2-like gene in the diatom *T. pseudonana* is linked to a role in silicification (Shrestha et al. 2012). In siliceous sponges, there is evidence for diversification of Lsi2-like genes (e.g., multiple genes found within the *Amphimedon queenslandica* genome). Sister to the siliceous sponge clade are sequences from bilaterian eumetazoans; limpets (which have silicified mouthparts [Hua and Li 2007]), brachiopods (whose larval stages produce siliceous tablets [Williams 1998]) and *C. teleta* (which possesses an SIT-L). It should be noted that several species not known to be siliceous possess Lsi2-like genes (e.g., *Bigelowiella natans* [rhizarian], *Dictyostelium discoideum* [amoebozoan]). However, siliceous species are over-represented versus nonsiliceous species in comparison to the PED-like group, suggesting that although Lsi2-like genes may have other cellular roles, they appear to have a conserved role in biosilicification. As with the SIT and SIT-L genes, their broad phylogenetic distribution supports an ancient origin for both Lsi2-like and PED-like genes, likely in the eukaryotic LCA, and with independent losses in multiple lineages.

The distribution of Lsi2-like sequences in many silicifying lineages makes them a prime candidate for further research into Si transport. Lsi2-like genes could play a role in Si transfer from the cytosol into the acidic silica deposition vesicle (driven by the H^+ ^gradient across the vesicle membrane) or may contribute to a system for Si(OH)_4_ uptake across the plasma membrane independent of SITs, in a manner similar to land plants. The presence of Lsi2-like genes in the siliceous sponges is therefore particularly interesting, as no Si transporter genes have yet been characterized from these organisms (Schröder et al. 2004).

### SITs over Geological Time

**Fig. 8:**
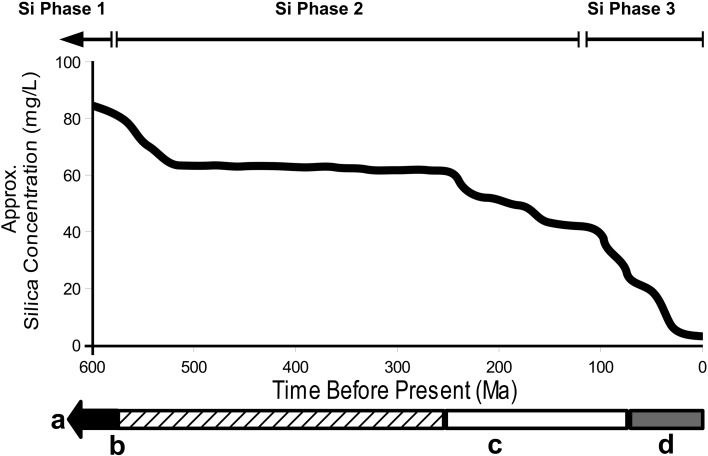
Geological history of Si. The graph plots approximate seawater silica concentration (at 25 °C) over the past 600 My, based on Racki and Cordey (2000). Indicated are the approximate age ranges of the three biosilicification phases, the Precambrian (black), Palaeozoic (hatching), Mesozoic (white), and Cenozoic (gray) eras and major events involving biosilicifying organisms. The first phase extends from at least the Archean (3000 Ma) until the Precambrian/Cambrian boundary. This is characterized by high seawater Si concentrations and witnessed the origin of the eukaryotic supergroups (a, ∼2700 Ma) (Parfrey et al. 2011). The second phase occurred between the Cambrian and the Mesozoic and saw a fall in seawater Si concentrations. Here, we find the first fossil evidence for biosilicifying organisms (b, spicules from ∼540 Ma) and widespread biogenic sedimentary silica deposits from sponges and radiolarians (Maldonado et al. 1999; Knoll 2003; Knoll and Kotrc 2015). The third phase covers the further reduction in seawater Si concentrations to modern levels (∼10 μM in surface waters). This phase is marked out by the appearance of new siliceous groups in the fossil record (c, approximately 200 Ma), the rise to dominance of the diatoms (d, ∼33 Ma) and reduced biosilicification across several taxa.

Si transporters are widely distributed and multiple lineages have undergone parallel gene losses, duplications, and diversifications. Some of these events may have been driven by physiological specialization in different lineages, such as the membrane physiology of land plants. However, the taxonomic distribution of the SIT family and Lsi2-like transporters implies gene loss on a massive scale, suggesting a common evolutionary driver for such loss. We propose a scenario where loss was largely driven by changes in environmental Si concentrations. Evidence from sedimentary records broadly shows three major phases of seawater Si geochemistry: a very high Si Precambrian ocean, an intermediate Si Palaeozoic/Mesozoic ocean, and a Mesozoic/Cenozoic low Si ocean (fig. 8) (Siever 1992; Racki and Cordey 2000; Knoll 2003; Maliva et al. 2005; Knoll and Kotrc 2015). We hypothesize that SIT and SIT-L evolution, and perhaps also the evolution of the Lsi2-like group, has been strongly influenced by the transition between these phases.

The origin and diversification of the eukaryotic supergroups (Parfrey et al. 2011) occurred during the Precambrian (fig. 8, black box) when ocean Si concentrations were extremely high (fig. 8a). Abiotically precipitated oceanic cherts deposited during this period constrain the Si concentration to >100 μM, and potentially up to 2 mM (Siever 1992; Maliva et al. 2005). Although there were multiple Si influx sources (Racki and Cordey 2000), Precambrian oceans lacked one major Si output flux: biosilicifying organisms. There is no fossil evidence for silica biomineralization until the latest Precambrian or early Cambrian (Sperling et al. 2010), thus permitting very high seawater Si concentrations.

These conditions would have presented a major challenge to living organisms. Many metalloids are toxic due to their interference with metabolic processes (Bienert et al. 2007). Si(OH)_4_ presents a special case as it can enter the cell via diffusion (Thamatrakoln and Hildebrand 2008). At concentrations above 2 mM, Si(OH)_4_ autopolymerizes out of solution into silica by condensation reactions (Annenkov et al. 2008). Free silica in the cytoplasm would bind to proteins and catastrophically disrupt cellular metabolism (Martin-Jézéquel et al. 2000). Modern siliceous organisms deliberately sequester Si(OH)_4_ within vesicles for controlled polymerization, but in the Precambrian seas the diffusive influx of Si would require constant Si homeostasis for all marine organisms. Therefore, we propose that the original function of Si transporters was as a detoxification mechanism to remove excess cellular Si(OH)_4_, and from our phylogenetic analysis we infer that this role was possibly present in the original eukaryote. The biphasic transport mechanism proposed for a multimeric SIT-L complex (see above) would allow insertion into the membrane in both orientations, and therefore Si transport either into or out of the cell. Such a role was previously proposed for SITs in localized high Si conditions, and by extension ancient oceans (Thamatrakoln and Hildebrand 2008). A similar hypothesis for biomineralized calcium carbonate structures has also been proposed, with mucins originally used as “anticalcification” mechanisms to prevent toxic external calcification, and only later being co-opted as organic matrix molecules for CaCO_3_ shells and skeletons (Marin et al. 1996).

Ocean geochemistry underwent major upheavals around the Precambrian/Cambrian boundary (Brennan et al. 2004; Sperling et al. 2010) (fig. 8b) and into the Palaeozoic (fig. 8, hatched box). A concomitant biotic upheaval at this time was the appearance of mineralized hard parts of calcium carbonate, calcium phosphate, and silica. The resulting evolutionary arms race led to a proliferation of skeletonized organisms, including siliceous radiolarians and sponges (Kouchinsky et al. 2011; Knoll and Kotrc 2015). The introduction of this output flux resulted in a large drop in oceanic Si concentration to around 60 μM, as evidenced by the lack of Phanerozoic abiotic cherts outside of unusual environments like hydrothermal waters (Maliva et al. 2005).

The fall in Si levels would have eliminated the threat of excessive Si(OH)_4_ uptake, and under our hypothesis, this reduction in selective pressure resulted in widespread losses of SITs and SIT-Ls. The timing of some of these losses can be constrained: the inferred presence of an SIT-L in the bilaterian LCA (fig. 4) restricts widespread bilaterian SIT-L gene losses to after the divergence of the three main lineages 600–700 Ma (Peterson et al. 2008). Loss of Si transporters does not rule out the possibility that Si may play a minor but fundamental role in eukaryotic biology unrelated to biomineralization, e.g., in protein conformation (Eglin et al. 2006).

The exceptions were those taxa that required Si for biomineralization of silica structures or as a minor component of calcareous structures. It should be noted that under this hypothesis, Si *transport* has ancient and deep origins and may be ancestral in eukaryotes; however, *biosilicification* is not. From molecular comparisons of silica polymerization mechanisms, it does appear that biosilicification has arisen independently in multiple different lineages (Sumper and Kroger 2004; Matsunaga et al. 2007; Gong et al. 2010; Shimizu et al. 2015; Durak et al. 2016).

The third phase (fig. 8) came in the Mesozoic (fig. 8, white box) and Cenozoic (fig. 8, gray box), with the transition to the phytoplankton ecosystems of modern oceans (fig. 8c), where stramenopiles, haptophytes, and dinoflagellates dominate (Falkowski et al. 2004). Diatoms, in particular, flourished (fig. 8d), resulting in a drawdown of surface seawater Si (Racki and Cordey 2000). Fossil evidence demonstrates the decline of siliceous sponge reefs (Maldonado et al. 1999) and scarcity of major biotic siliceous deposits in Cenozoic sediments (Racki and Cordey 2000). Silicoflagellate (dictyochophyte), diatom, and radiolarian microfossils (Lazarus et al. 2009; Finkel and Kotrc 2010; Van Tol et al. 2012) show a trend toward lighter silicified structures and more efficient biosilica use through this phase. Sponges and radiolarians, the dominant siliceous groups of the Palaeozoic (fig. 8) are Si limited under modern seawater conditions (Maldonado et al. 1999, 2011) and are restricted to low latitudes or deeper waters with greater Si availability and less competition from diatoms.

In contrast, siliceous groups such as loricate choanoflagellates, chrysophytes, and dictyochophytes are common alongside diatoms in modern oceans. This may be due in part to possession of a more efficient SIT-based Si uptake mechanism. Declining Si levels necessitated the independent duplication and fusion of SIT-Ls in the nondiatom stramenopiles, leading to the convergent evolution of SITs (Group 2 SITs, see figs. 4 and 7). For groups already possessing Group 1 SITs (choanoflagellates and diatoms, fig. 2), the fall in Si levels may have contributed to diversification in SITs into a more efficient suite of specialized transporters and sensors (see above).

The deep origin and widespread distribution (figs. 2 and 6) of Lsi2-like genes resembles the evolutionary history of SIT-L and SIT type Silicon Transporters. We hypothesize that these genes were also involved in detoxification of metalloids, including Si, in Precambrian oceans. As with SITs and SIT-Ls, once the selective pressure for metalloid detoxification was reduced, Lsi2-like genes were lost independently across multiple lineages. In land plants, Lsi2 was presumably retained as an active Si transporter to control Si(OH)_4_ uptake rates from Si-replete soil water. Lsi2 apparently had this role very early in land plant evolution, before the recruitment of NIPII-class transmembrane proteins as passive Si channel pores (Trembath-Reichert et al. 2015).

Our analyses (fig. 6) suggest that Lsi2-like genes may have a Si-related role in siliceous sponges, choanoflagellates, and lophotrochozoans. An Lsi2-like transporter was likely present in the LCA of the metazoans, restricting the loss of Lsi2-like genes until after the bilaterian diversification <600 Ma (Peterson et al. 2008). Under our hypothesis, sponge Lsi2-like transporters were originally involved in detoxification and were co-opted for a role in spicule formation at the Precambrian/Cambrian boundary, with siliceous spicules evolving independently in different sponge groups. This could explain the apparent “spicule gap” in the fossil record (Sperling et al. 2010) and makes the sponge Lsi2-like genes an interesting candidate for further research. The role of Lsi2 in sponges may be particularly important, as SITs and SIT-Ls are absent from this lineage. Group 1 SITs and SIT-Ls are present in choanoflagellates and metazoans, respectively (fig. 2), suggesting that the sponges may actually have lost these transporter types prior to the emergence of biosilicification.

## Conclusions

**Fig. 9:**
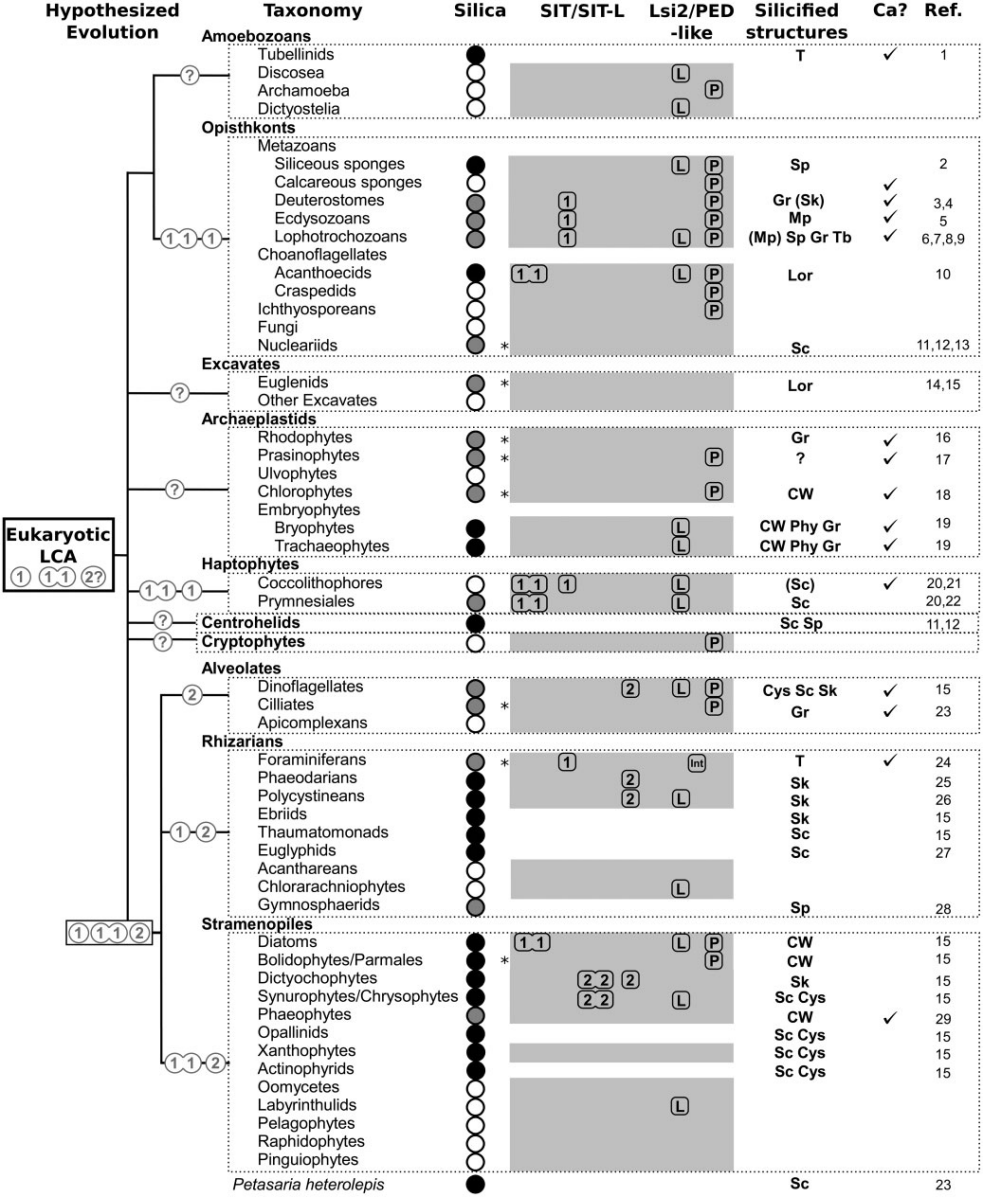
Distribution of biomineralization and transporter genes in eukaryotic groups. Summary table of the presence of silica biomineralization, calcium biomineralization and Si-related transporter genes herein examined in selected eukaryotic groups (taxonomic classification based on Adl et al. 2012, italics signify *incertae sedis*). Black circle = extensive/widespread biosilicification, gray circle= minor/limited biosilicification, white circle = biosilicification absent. Gray shading denotes where genomic or transcriptomic data was available for analysis; asterisks mark where data from that lineage was only available from non-siliceous species. Symbols are placed in taxa where relevant genes were detected; blanks indicate gene not detected and are not a definitive statement of absence. 1 = Group 1, 2 = Group 2; double for SIT, single for SIT-L (see fig. 4). L = Lsi2-like, P = pink-eye dilution-like, Int = intermediate type (see fig. 6). The simplified phylogeny (based on fig. 1) is annotated with our hypothesis for the SIT repertoire (circled) of the eukaryotic LCA, and at the base of each eukaryotic supergroup. This interpretation is based on the ancestral origin hypothesis that assumes vertical inheritance in major groups rather than HGT (see Discussion). Question marks identify supergroups where no SITs or SIT-Ls have been confirmed thus far. Note that Group 2 SIT-Ls are today only found in the SAR supergroup; however, the lack of robust phylogenetic support for the branches separating Group 2 SITs/SIT-Ls from the eukaryotic LCA and the uncertain position of the root (fig. 4) means we cannot rule out a Group 2 origin in the eukaryotic LCA (marked by the 2? symbol) based on our present results. Silicified structures: CW = cell wall, Cys = cysts, Gr = granules, Lor = lorica, Mp = mouthparts, Phy = phytoliths, Sc = scales, Sp = spicules, T = test, Tb = tablets,? = uptake evidence for unknown Si utilization; brackets indicate that is a Si minor component of composite biomineralized structure. The “Ca?” column reports instances of calcification with a tick (Faber and Preisig 1994; Knoll 2003; Knoll and Kotrc 2015). References: 1. Lahr et al. 2013; 2. Sperling et al. 2010; 3. Monniot et al. 1992; 4. Carlisle 1981; 5. Karlson and Bamstedt 1994; 6. Bone et al. 1983; 7. Hua and Li 2007; 8. Williams 1998; 9. Desouky et al. 2002; 10. Leadbeater 2015; 11. Nicholls and Durrschmidt 1985; 12. Patterson and Durrschmidt 1986; 13. Patterson and Durrschmidt 1988; 14. Conforti et al. 1994; 15. Preisig 1994; 16. Chopin et al. 2004; 17. Fuhrman et al. 1978; 18. Millington and Gawlik 1967; 19. Hodson et al. 2005; 20. Durak et al. 2016; 21. Drescher et al. 2012; 22. Yoshida et al. 2006; 23. Foissner et al. 2009; 24. Sen Gupta 2003; 25. Anderson 1986; 26. Ogane et al. 2010; 27. Anderson 1994; 28. Febvre-Chevalier 1973; 29. Mizuta and Yasui 2012.

We have identified SITs and SIT-Ls in a range of eukaryotic groups, including many silicified lineages (fig. 9) and also identified SIT-Ls in bacteria. Phylogenetic analysis points toward an ancient origin deep in the eukaryotic phylogeny, with the inference (on the assumption of vertical inheritance rather than HGT within eukaryotes) that the eukaryotic LCA may have possessed Silicon Transporter genes (fig. 9). Subsequently there have been frequent gene losses independently in not only different lineages but also convergent duplication of 5-TMD SIT-Ls to produce 10-TMD SITs, followed in some lineages by parallel diversification of SITs with different functions. Although alternative evolutionary scenarios involving ancient HGT events may also explain the taxonomic distribution of the SITs and SIT-Ls, these scenarios also require extensive gene loss and multiple independent origins of the SITs. Analysis of another active Si transporter, Lsi2, finds a similar distribution of Lsi2-like genes in siliceous groups compared with nonsiliceous groups (fig. 9) suggesting a conserved role in eukaryotic biosilicification. We hypothesize that Si transporters originally evolved as a detoxification mechanism in high Si oceans. Biosilicification evolved independently in many lineages in the Phanerozoic, with recruitment of different Si transport mechanisms. This introduced new selective pressures, resulting in the complex evolutionary dynamics of loss and convergence observed in Si transporters today. The widespread distribution and deep origins of Si transporters suggests that Si utilization may not be limited to extensively siliceous organisms and points toward a widespread and fundamental role for Si in the evolution and biology of eukaryotes.

## Materials and Methods

### BLAST Searches

*S. diplocostata* SITa (Marron et al. 2013) and *Oryza sativa* Lsi2 (Ma et al. 2007) were used as query sequences for BLASTp and tBLASTn searches using the default parameters (Altschul et al. 1997). Data sets searched were the EMBL/Genbank nucleotide, protein, EST and TSA archives, the MMETSP transcriptome data sets, the Aniseed ascidian genome and transcriptome database (http://www.aniseed.cnrs.fr/; last accessed October 12, 2016), the *Aphrocallistes vastus* transcriptome at the ERA archive of the University of Alberta (https://era.library.ualberta.ca/files/bvd66w001v#.V_1foSRCi7l; last accessed October 11, 2016), the *Equisetum giganteum* transcriptome (Vanneste et al. 2015), and the Compagen database (http://www.compagen.org/; last accessed October 12, 2016). Transcriptomes of 19 choanoflagellate species, 7 rhizarian species, 2 stramenopile species (see supplementary table S1, Supplementary Material online) and *P. neolepis* (Durak et al. 2016) were also searched.

Due to the relatively low similarity of the bacterial SIT-L sequences to SdSITa, the *Synechococcus* sp. KORDI-100 SIT-L was used as a query sequence for prokaryotic database searches, including Cyanobase (http://genome.microbedb.jp/cyanobase; last accessed October 12, 2016), Cyanorak (www.sb-roscoff.fr/cyanorak/; last accessed October 12, 2016), and the *Tara* Oceans metaG Environmental Sequence Datasets (0.22–8-μm fraction samples) (Sunagawa et al. 2015).

A more selective sampling strategy was employed for Lsi2 because of the wider taxonomic distribution, with an emphasis on sponges and nonangiosperm land plants. Hits to Lsi2 were also selected from a taxonomically representative array of prokaryotes and eukaryotes where full genomes were available. The MMETSP transcriptomes and unpublished data sets (see above) were also searched. Top sequence hits from representative species were selected to ensure a broad sampling across MMETSP data, with special reference to transcriptomes which contained SIT or SIT-L sequences. For each species, multiple high-scoring hits were used for further analysis.

### Decontamination Methods

Analysis of SIT sequences is vulnerable to misidentification due to contamination. SITs are poorly characterized and irregularly distributed taxonomically, making a priori phylogenetic assessments difficult. SITs may be highly expressed, so even a minor degree of contamination could result in erroneous SIT contigs in a de novo assembly. Furthermore, most of the data sets being investigated were from previously unsequenced species and taxonomic groups and often from nonaxenic cultures. These problems are compounded through misreading of multiplexed indexes in Illumina Transcriptome Sequencing leading to cross-contamination (Kircher et al. 2012).

We employed a bioinformatic procedure to solve the problem of Illumina Sequencing cross-contamination in the 19 choanoflagellate transcriptomes and the MMETSP data sets (Keeling et al. 2014). First, simple repeats in the contig files were soft-masked using Dustmasker v1.0.0 (Morgulis et al. 2006) using the default parameters to eliminate spurious hits at a high percentage identity. Then an “all versus all” sequence comparison at the nucleotide level with LAST v418 (Kiełbasa et al. 2011) with the option “−e64,” retaining only the top-scoring hit between any pair of contigs.

Instances of cross-contamination between sequencing projects are identified only by comparison of projects involving different species names, or by comparison of different “unknown” strains. In the case of the MMETSP projects, seven pairs of differently named species were apparently identical or near-identical (*Alveolata* sp. CCMP3155 and *Vitrella brassicaformis*; *Glenodinium foliaceum* and *Kryptoperidinium foliaceum*; *Gloeochaete witrockiana* and *Gloeochaete wittrockiana*; *Isochrysis galbana* and *Isochrysis* sp. CCMP1324; *Symbiodinium* sp. CCMP2430 and *Symbiodinium* sp. D1a; Undescribed NY07348D and Unknown NY0313808BC1; Unidentified sp. CCMP1205 and Unidentified sp. CCMP2175). Cross-matches between these pairs were therefore discarded.

Separate percentage identity distributions for hits between each pair of projects were built. Matching contig pairs were only considered to be hits where hit length was ≥150 nt and/or where the length of the alignment was at least 50% of the length of the shorter of the two contigs. Cross-contaminated hits should represent a peak at 100% identity or slightly lower (depending on sequencing and assembly error), and “true” hits between species should be distributed around a lower percentage identity. Thus, if there are any cross-contaminated hits, there should be a local minimum in the distribution between 100% and the “true” average percentage identity between the two species. For true cross-contamination, the number of hits at 100% identity must be greater than the number at 99% identity. Percentage identity bins should then descend from 99% in increments of 1%, until three consecutive bins are found the lower two of which contain a number of hits greater than or equal to the previous bin. This should represent the threshold between cross-contaminated hits and “true” hits. A threshold was designated as representative of cross-contamination between projects for each percentage identity distribution.

Application of the percentage identity threshold to each pair of projects allowed cross-contaminant contigs within a sequencing project to be marked. An exception was made when the reads per kilobase per million mapped reads (RPKM) in one project was ≥10× the RPKM in the other project of the pair. In this case, the contig in the first project is retained, whereas the contig in the second contig is discarded. Alternatively, if the RPKM in the first project is ≥10,000 then this contig is retained. This is because very highly conserved and highly expressed genes are expected to have high percentage identities between species, and in such cases, a 10× RPKM ratio between projects may not be achieved.

Contigs above the percentage identity threshold and marked as cross-contaminants are removed from the nucleotide data and also from the predicted protein data. The contigs and predicted proteins purged as putative cross-contaminants should be retained in separate files if required for later verification. In the case of the MMETSP data sets, statistics on the number of contigs removed as cross-contaminants are given in supplementary table S5, Supplementary Material online.

To detect contamination from foreign eukaryotes present in sequenced cultures, we investigated the phylogeny of Hsp90 and EF-1, highly expressed housekeeping genes ubiquitous in eukaryotes that should be detectable from contaminating species. Hsp90 and EF-1 sequences are available for all of the eukaryotic supergroups and for most of the species where SIT or SIT-L sequences were detected in the MMETSP data sets. For *Dictyocha speculum*, neither Hsp90 nor EF-1 provided sufficient taxonomic resolution, so psaA was used as an alternative.

Representative reference framework sequences (see supplementary table S3, Supplementary Material online) were selected according to BLAST similarity scores to *P. tricornutum* Hsp90, *T. pseudonana* EF-1, or *D. speculum* psaA. These reference sequences were then used as BLAST search queries for each MMETSP data set with a hit to SdSITa. High-scoring contig hits were selected, with a cut-off depending on the number of hits and the E-value compared with that of the best match.

Framework and the selected top-scoring hits were aligned using ClustalW under the default settings (Thompson et al. 1994). Maximum Likelihood analysis of each of the alignments generated was carried out using PhyML v3.0 (Guindon et al. 2010) with the LG + G4 model, the proportion of invariant sites and equilibrium frequencies of amino acids estimated from the data fixed according to the LG model. Starting trees were generated by BioNJ, with tree searching using NNI heuristic methods. Topology and branch lengths were optimized in ML calculations. One hundred bootstrap replicates were analyzed using the same model and method for the PhyML analysis. Phylogenetic trees generated were viewed using FigTree v.1.3.1 (A. Rambaut, Institute of Evolutionary Biology, University of Edinburgh 2006-2009). These trees are given in supplementary figure S2 and S3, Supplementary Material online.

Transcriptomes were deemed to be at risk of contamination if contigs branched, with >70% bootstrap support, distant from the relevant framework housekeeping gene sequence. Where a contig branched within a well-supported stramenopile or choanoflagellate clade, and away from the relevant framework reference sequences, the SIT hits from this species were discarded as potentially being contaminant-derived. Possible contaminant-derived sequences are listed in supplementary table S2, Supplementary Material online. An exception was made where contigs branched with prokaryotic sequences as bacterial contamination was common in the MMETSP cultures; post hoc phylogenetic analysis found no evidence for SIT-Ls being due to bacterial contamination.

Choanoflagellate SIT sequences were confirmed by RT-PCR amplification and cloning. RNA was isolated using a TRIzol-based method (Invitrogen), dTcDNA synthesized using Superscript III Reverse Transcriptase (Thermo-Fisher), and ∼1 μg of cDNA used as template. The primers used are given in supplementary table S6, Supplementary Material online. RT-PCR was conducted using Phusion High-Fidelity Polymerase (New England Biolabs) as per the manufacturer's instructions. RT-PCR conditions were as follows: a hot-start denaturing step at 98 °C for 30s; 35 cycles of 98 °C (10s), 65 °C (30s), 72 °C (60s) and final elongation step of 72 °C for 5 min. Amplified sequences were cloned into the PGEM-T Easy Vector System (Promega) or TOPO TA Vector System (Thermo-Fisher) and Subcloning Efficiency DH5α Competent Cells (Invitrogen). Plasmids were extracted using a Qiaprep Spin Miniprep Kit (Qiagen) and sequenced by Geneservice (Cambridge, United Kingdom) or the UC Berkeley Sequencing Facility.

Redundant sequences (e.g., short contigs or those with only very minor variation) were removed, and overlapping contigs were combined to make a single, longer sequence. The previously identified SdSITb and SdSITc sequences (Marron et al. 2013) were redesignated as SdSITαBC as a result. Silicon Transporter sequences used for further phylogenetic analysis, and those excluded as likely contaminants, are detailed in supplementary table S2, Supplementary Material online.

As with SITs, preliminary alignments were used to filter Lsi2 BLAST hits to remove redundant sequences (data not shown). supplementary table S4, Supplementary Material online lists the Lsi2-matching sequences used for phylogenetic analysis. Possible contaminant-derived sequences are noted.

### Bioinformatic and Phylogenetic Analysis

Alignments of SIT and SIT-L sequences were created using MAFFT L-INS-i v7 (Katoh et al. 2005; Kuraku et al. 2013) with a gap opening penalty of 3 and offset value of 1. The short SIT-L sequences from *Collozoum* sp., *S. streptacantha*, and *Halocynthia roretzi*, and the very long *Florenciella* sp. SIT sequence (which though having firm evidence for being classified as an SIT shows indications of a frameshift assembly error after the second GRQ motif) were used for preliminary analyses but excluded from the final phylogenies due to the possibility of distorting the tree topologies by introducing phylogenetic artefacts. From the final alignment a sub-alignment containing only choanoflagellate SIT sequences was extracted. TMPred (Hoffman and Stoffel 1993) was used to predict potential TMDs and the N-terminal location. TMD predictions were fitted to the alignment, with reference to previous TMD predictions for other SITs (Marron et al. 2013). This overlay was used to split the SIT sequences into N-terminal and C-terminal halves at a conserved point between TMD5 and 6 (at the point aligned with *P. tricornutum* SIT1 residues 219 and 220). These “half-SIT” sequences realigned with SIT-L sequences using MAFFT L-INS-i v7 under the default settings. Sequences similar to Lsi2 (supplementary table S4, Supplementary Material online) were aligned using MAFFT L-INS-i v7 under the default settings.

Alignments were trimmed automatically using TrimAl v1.2 (Capella-Gutierrez et al. 2009) using the gappyout method (for SIT-L + full-SIT and choanoflagellate-only alignments) or heuristic automated1 method (for SIT-L + half-SIT alignment). The SIT-L + full-SIT alignment of 62 sequences contained a total of 485 positions, the choanoflagellate-only SIT alignment contained 13 sequences with a total of 519 positions, the SIT-L + half-SIT alignment contained 98 sequences with a total of 166 positions and the Lsi2 alignment contained 186 sequences of 247 positions. These were used for maximum likelihood analyses using IQ-TREE v1.4.2 (Minh et al. 2013; Nguyen et al. 2015), PhyML v3.0 and RaxML v8.2.6 (Stamatakis 2014). Homo v1.2 (Rouse et al. 2013) confirmed that all alignments were consistent with the assumption of evolution under time-reversible conditions. Optimal evolutionary models were selected under the corrected Akaike Information Criterion using IQ-TREE Model Selection; for the choanoflagellate-only SIT alignment the LG model with a four-category gamma distribution of rate variation and empirical frequencies estimated from the data (LG + G4 + F) was optimal, for the SIT-L + half-SIT alignment the LG model with a four-category gamma distribution of rate variation (LG + G4) provided the best fit (Le and Gascuel 2008). For the SIT-L + full-SIT alignment, the optimal model was LG with five-category FreeRate model and empirical frequencies estimated from the data (LG + R5 + F); however, this model is only implemented in IQ-TREE and therefore for other phylogenetic methods the LG + G4 + F model was used because it was the best-fitting alternative. For the Lsi2 alignment, IQ-TREE selected the mtZOA model. As this model is derived from animal mitochondrial protein sequences (Rota-Stabelli et al. 2009) and because it is not implemented in MrBayes, we instead used the second-best fitting model (LG + G4).

PhyML starting trees were generated by BioNJ, with tree searching using SPR heuristic methods. Topology and branch lengths were optimized in ML calculations. 100 bootstrap replicates or 1000 ultrafast bootstrap replicates (IQ-TREE only) were analyzed using the same model and method for the ML analyses. For PhyML and IQ-TREE SH supported, approximate Likelihood Ratio Tests were also carried out using the default settings. Bayesian tree inference was performed using MrBayes version 3.2.6 (Huelsenbeck and Ronquist 2001) using the default settings of two independent runs with four chains each, running until the average standard deviation of split frequencies between the two runs reached 0.01. All other parameters were left at their default values. Trees were summarized with the default burn-in of 25% of samples. The runs on the Lsi2 Bayesian analysis did not converge and instead stabilized at a standard deviation of split frequencies above 0.01. A consensus tree at the point the runs were stopped in included as supplementary text S5, Supplementary Material online; this tree agrees with the results of the ML analyses.

Further phylogenetic analyses were conducted without bacterial SIT-Ls to test whether the inclusion of bacterial sequences in the SIT-L + full-SIT analysis resulted in phylogenetic artifacts such as long-branch attraction. Alignments were generated using MAFFT L-INS-i v7 with a gap opening penalty of 3 and offset value of 1, and trimmed with the heuristic automated one method in TrimAl v1.2 to produce a data set of 56 sequences and a total of 371 positions. The optimal model was selected (LG + G4 + F) and RaxML, PhyML, IQ-TREE, and MrBayes analyses were carried out as before. The results of these phylogenetic analyses are given in supplementary figure 8A–D, Supplementary Material online.

We also analyzed alignments for SIT-L + full-SIT sequences (both with and without bacterial sequences), and for the SIT-L + half-SIT alignment, using the CAT model in PhyloBayes (Lartillot et al. 2009, 2013) in order to test for possible phylogenetic artefacts caused by the presence of fast-evolving sequences (supplementary figure S8E–G, Supplementary Material online). We performed tree inference using PhyloBayes MPI version 1.6 with four independent chains for each data set using the default GTR + CAT model of sequence evolution and excluding invariant sites (-dc). Runs were stopped when the largest discrepancy across tree bipartitions from different runs (after discarding burn-in) reached approximately 0.1, as recommended by the authors of the software. We also checked convergence of the continuous parameters of the model, and all had an effective sample size of greater than 400 and a maximum difference (after discarding burn-in) of roughly 0.1 or less. All data sets were run for 40,000 generations or more, so we discarded a consistent burn-in level of 10,000 samples when calculating convergence or producing consensus trees.

The phylogenies generated were manipulated using Archaeopteryx (Han and Zmasek 2009), and nodes collapsed where appropriate according to statistical support values (uncollapsed Lsi2 trees are given in supplementary texts S1–S5, Supplementary Material online). Resulting trees were viewed using FigTree.

### Choanoflagellate Si Treatment and SIT RT-qPCR

Control-Si cultures of *D. grandis* and *S. diplocostata* were grown in artificial seawater (ASW) made from 36.5 g/l Marin Salts (Dr. Biener Aquarientechnik, Wartenberg Germany in ddH_2_O. ASW was vacuum-filtered through a 0.22-µm Steriop GP Express Plus filter (Millipore, MA) into a sterile 1-l screw-top glass bottle (Schott Duran) and sterilized by autoclaving. Thirty-five milliliters of ASW was added to 10 ml of concentrated choanoflagellate culture in 50-ml polypropylene conical tubes (Fisher Scientific). High-Si treatment was created by adding 3.75 ml of a 2 mM stock solution of Na_2_SiO_3_.9H_2_O (Aldrich) in ultrapure ddH_2_O. Low-Si treatment was created by replacing ASW with a custom seawater recipe (see supplementary table S7, Supplementary Material online). Si concentrations of each treatment were measured by silicomolybdate assay (Strickland and Parsons 1968; Thamatrakoln and Hildebrand 2008) (see supplementary table S8, Supplementary Material online). Organic enrichment (4 g/l Proteose Peptone [Sigma], 0.8 g/L Yeast Extract [Fluka Biochemika] in ddH_2_O, autoclaved sterile) was added all treatments at a concentration of 15-µl/ml ASW to provide nutrition for prey bacteria. Inoculants were taken from the same batch of concentrated choanoflagellate culture to ensure that the initial gene expression levels were equivalent across all treatments.

All Si treatment cultures were grown at 13.5 °C for 7 days, sufficient for completion of at least three cell cycles (Leadbeater 2015) and thus SIT expression during lorica production. After 48 hours, 100 ml of culture from each treatment was harvested for RNA extraction using TRIzol (Invitrogen). cDNA was then synthesized using Superscript III Reverse Transcriptase (Thermo-Fisher) according to the manufacturer’s instructions.

RT-qPCR was conducted on the Lightcycler 480 II Real-Time PCR System (Roche) and with the SYBR Green Master Mix (Roche) system according to the manufacturer’s instructions. The qPCR primers used are given in supplementary table S6, Supplementary Material online. Primer pairs were tested using pilot studies and their melting curves verified. In accordance with the MIQE guidelines (Bustin and Nolan 2004), a standard curve was created for each SIT primer set based on a dilution series of *D. grandis* or *S. diplocostata* cDNA and carrier tRNA (Sigma). RT-qPCR measurements for each gene from each treatment were conducted in triplicate, together with a ddH_2_O blank control. PCR efficiencies, quantification cycles, and compensation points for each gene were detected using the Lightcycler 480 Software v1.5 (Roche). RT-qPCR analysis was conducted for three independent replicates of each Si treatment, from three independent batches of starting choanoflagellate culture for each species. The results of the RT-qPCR measurements were statistically analyzed using REST 2009 software, RG mode using the pair-wise fixed randomization test with 10,000 permutations (Pfaffl et al. 2002).

## Supplementary Material

Supplementary methods, supplementary tables S1–S8 and figures S1-S8 are available at *Molecular Biology and Evolution* online (http://www.mbe.oxfordjournals.org/).

Supplementary Data
