# Supplementary material for: The Evolution of Silicon Transport in Eukaryotes
Source: Mol Biol Evol. 2016 Oct 11;33(12):3226–48. doi: 10.1093/molbev/msw209 (PMC5100055; doi:10.1093/molbev/msw209)
Supplement: Supplementary Data [file supp_33_12_3226__index.html]

The Evolution of Silicon Transport in Eukaryotes — The Evolution of Silicon Transport in Eukaryotes — The Evolution of Silicon Transport in Eukaryotes — Supplementary Data 

# The Evolution of Silicon Transport in Eukaryotes

## Supplementary Data

files

- Supplementary Data - zip file
