# Supplementary material for: The Evolution of Silicon Transport in Eukaryotes
Source: Mol Biol Evol. 2016 Oct 11;33(12):3226–48. doi: 10.1093/molbev/msw209 (PMC5100055; doi:10.1093/molbev/msw209)
Supplement: Supplementary Data [file supp_msw209_suppl_data.zip › Supplementary_Figure_3.pdf]

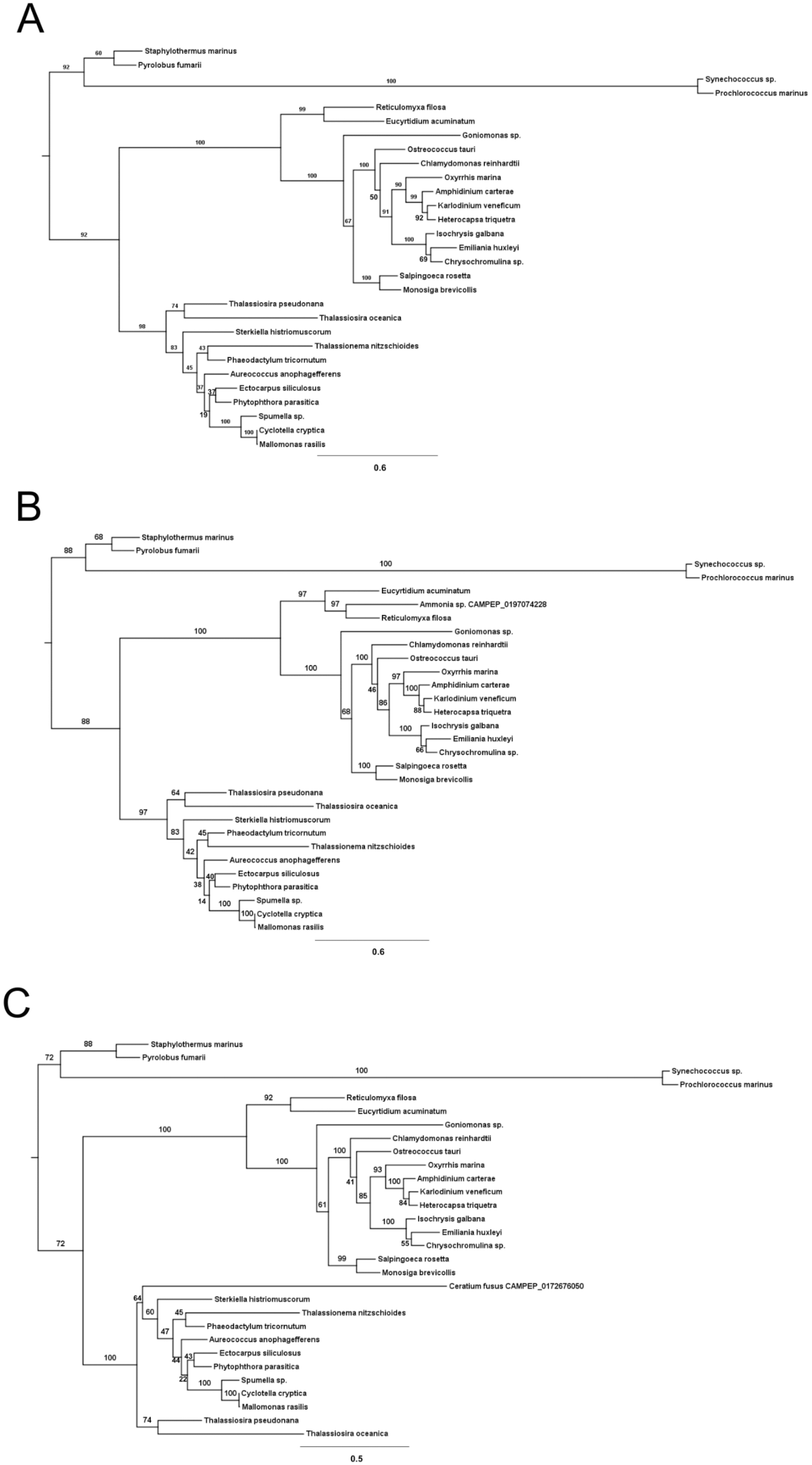
A
Staphylothermus marinus
Pyrolobus fumarii
Synechococcus sp.
Prochlorococcus marinus
Reticulomyxa filosa
Eucyrtidium acuminatum
Goniomonas sp.
Ostreococcus tauri
Chlamydomonas reinhardtii
Oxyrrhis marina
Amphidinium carterae
Karlodinium veneficum
Heterocapsa triquetra
Isochrysis galbana
Emiliania huxleyi
Chrysochromulina sp.
Salpingoeca rosetta
Monosiga brevicollis
Thalassiosira pseudonana
Thalassiosira oceanica
Sterkiella histriomuscorum
Thalassionema nitzschioides
Phaeodactylum tricornutum
Aureococcus anophagefferens
Ectocarpus siliculosus
Phytophthora parasitica
Spumella sp.
Cyclotella cryptica
Mallomonas rasilis
0.6
B
Staphylothermus marinus
Pyrolobus fumarii
Synechococcus sp.
Prochlorococcus marinus
Eucyrtidium acuminatum
Ammonia sp. CAMPEP_0197074228
Reticulomyxa filosa
Goniomonas sp.
Chlamydomonas reinhardtii
Ostreococcus tauri
Oxyrrhis marina
Amphidinium carterae
Karlodinium veneficum
Heterocapsa triquetra
Isochrysis galbana
Emiliania huxleyi
Chrysochromulina sp.
Salpingoeca rosetta
Monosiga brevicollis
Thalassiosira pseudonana
Thalassiosira oceanica
Sterkiella histriomuscorum
Phaeodactylum tricornutum
Thalassionema nitzschioides
Aureococcus anophagefferens
Ectocarpus siliculosus
Phytophthora parasitica
Spumella sp.
Cyclotella cryptica
Mallomonas rasilis
0.6
C
Staphylothermus marinus
Pyrolobus fumarii
Synechococcus sp.
Prochlorococcus marinus
Reticulomyxa filosa
Eucyrtidium acuminatum
Goniomonas sp.
Chlamydomonas reinhardtii
Ostreococcus tauri
Oxyrrhis marina
Amphidinium carterae
Karlodinium veneficum
Heterocapsa triquetra
Isochrysis galbana
Emiliania huxleyi
Chrysochromulina sp.
Salpingoeca rosetta
Monosiga brevicollis
Ceratium fusus CAMPEP_0172676050
Sterkiella histriomuscorum
Thalassionema nitzschioides
Phaeodactylum tricornutum
Aureococcus anophagefferens
Ectocarpus siliculosus
Phytophthora parasitica
Spumella sp.
Cyclotella cryptica
Mallomonas rasilis
Thalassiosira pseudonana
Thalassiosira oceanica
0.5

D

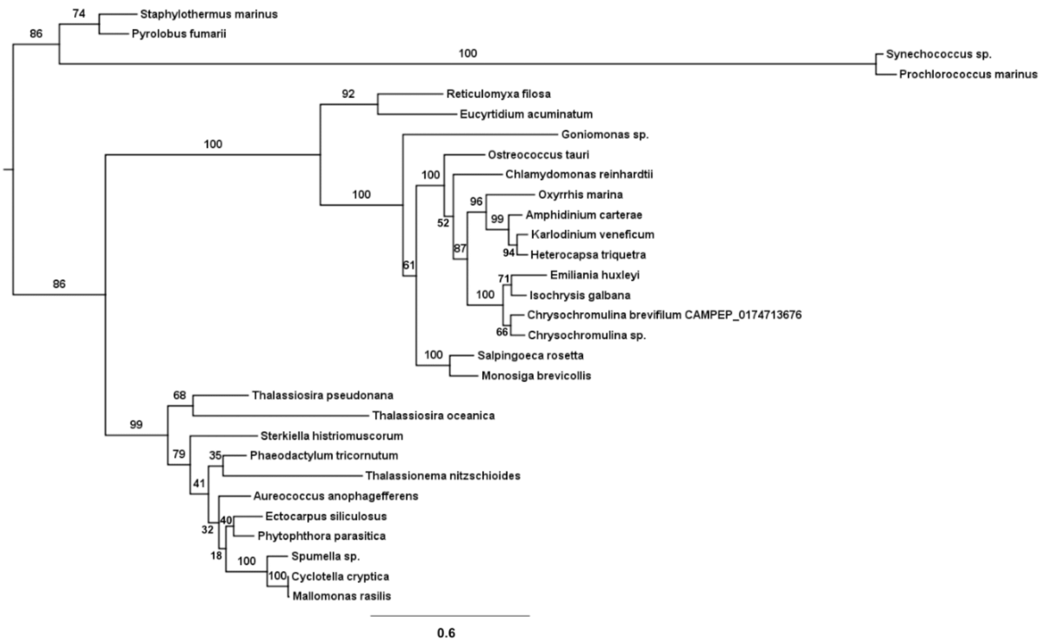
Staphylothermus marinus
Pyrolobus fumarii
Synechococcus sp.
Prochlorococcus marinus
Reticulomyxa filosa
Eucyrtidium acuminatum
Goniomonas sp.
Ostreococcus tauri
Chlamydomonas reinhardtii
Oxyrrhis marina
Amphidinium carterae
Karlodinium veneficum
Heterocapsa triquetra
Emiliania huxleyi
Isochrysis galbana
Chrysochromulina brevifilum CAMPEP_0174713676
Chrysochromulina sp.
Salpingoeca rosetta
Monosiga brevicollis
Thalassiosira pseudonana
Thalassiosira oceanica
Sterkiella histriomuscorum
Phaeodactylum tricornutum
Thalassionema nitzschioides
Aureococcus anophagefferens
Ectocarpus siliculosus
Phytophthora parasitica
Spumella sp.
Cyclotella cryptica
Mallomonas rasilis
0.6

E

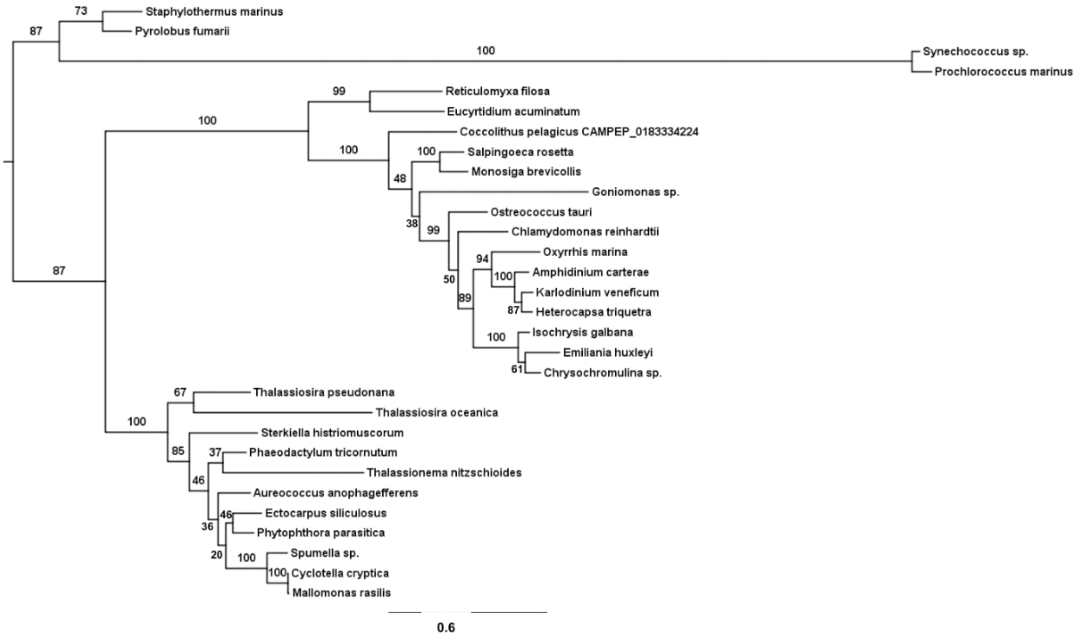
Staphylothermus marinus
Pyrolobus fumarii
Synechococcus sp.
Prochlorococcus marinus
Reticulomyxa filosa
Eucyrtidium acuminatum
Coccolithus pelagicus CAMPEP_0183334224
Salpingoeca rosetta
Monosiga brevicollis
Goniomonas sp.
Ostreococcus tauri
Chlamydomonas reinhardtii
Oxyrrhis marina
Amphidinium carterae
Karlodinium veneficum
Heterocapsa triquetra
Isochrysis galbana
Emiliania huxleyi
Chrysochromulina sp.
Thalassiosira pseudonana
Thalassiosira oceanica
Sterkiella histriomuscorum
Phaeodactylum tricornutum
Thalassionema nitzschioides
Aureococcus anophagefferens
Ectocarpus siliculosus
Phytophthora parasitica
Spumella sp.
Cyclotella cryptica
Mallomonas rasilis
0.6

F

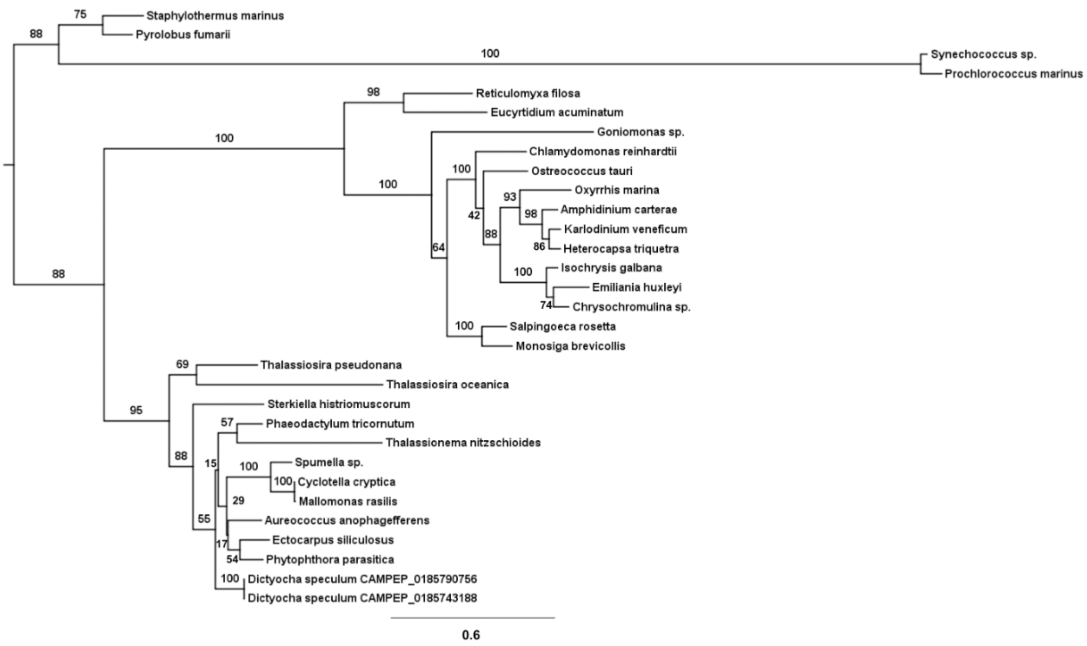
Staphylothermus marinus
Pyrolobus fumarii
Synechococcus sp.
Prochlorococcus marinus
Reticulomyxa filosa
Eucyrtidium acuminatum
Goniomonas sp.
Chlamydomonas reinhardtii
Ostreococcus tauri
Oxyrrhis marina
Amphidinium carterae
Karlodinium veneficum
Heterocapsa triquetra
Isochrysis galbana
Emiliania huxleyi
Chrysochromulina sp.
Salpingoeca rosetta
Monosiga brevicollis
Thalassiosira pseudonana
Thalassiosira oceanica
Sterkiella histriomuscorum
Phaeodactylum tricornutum
Thalassionema nitzschioides
Spumella sp.
Cyclotella cryptica
Mallomonas rasilis
Aureococcus anophagefferens
Ectocarpus siliculosus
Phytophthora parasitica
Dictyocha speculum CAMPEP_0185790756
Dictyocha speculum CAMPEP_0185743188
0.6

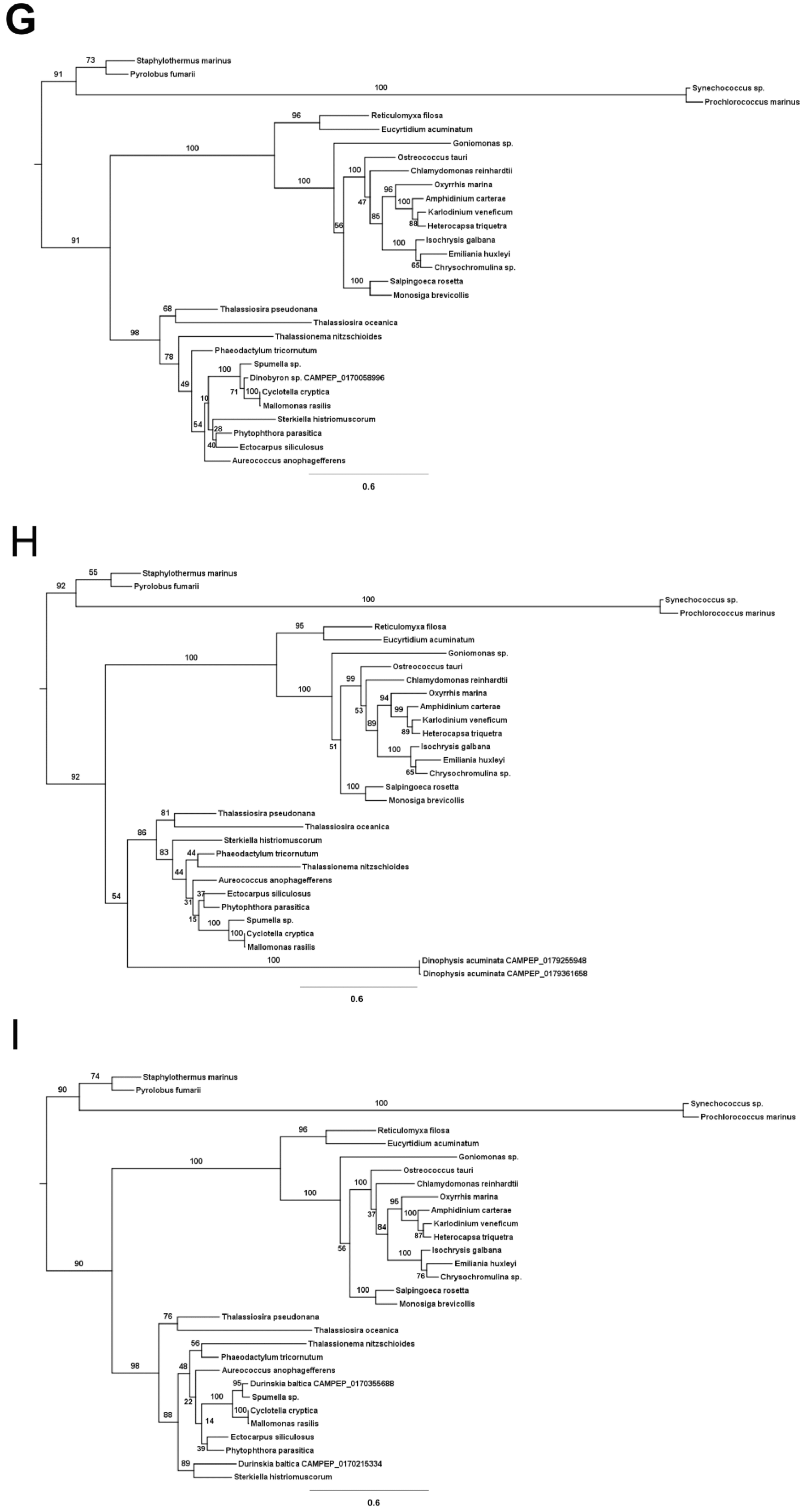
G
Staphylothermus marinus
Pyrolobus fumarii
Synechococcus sp.
Prochlorococcus marinus
Reticulomyxa filosa
Eucyrtidium acuminatum
Goniomonas sp.
Ostreococcus tauri
Chlamydomonas reinhardtii
Oxyrrhis marina
Amphidinium carterae
Karlodinium veneficum
Heterocapsa triquetra
Isochrysis galbana
Emiliania huxleyi
Chrysochromulina sp.
Salpingoeca rosetta
Monosiga brevicollis
Thalassiosira pseudonana
Thalassiosira oceanica
Thalassionema nitzschioides
Phaeodactylum tricornutum
Spumella sp.
Dinobyron sp. CAMPEP_0170058996
Cyclotella cryptica
Mallomonas rasilis
Sterkiella histriomuscorum
Phytophthora parasitica
Ectocarpus siliculosus
Aureococcus anophagefferens
0.6
H
Staphylothermus marinus
Pyrolobus fumarii
Synechococcus sp.
Prochlorococcus marinus
Reticulomyxa filosa
Eucyrtidium acuminatum
Goniomonas sp.
Ostreococcus tauri
Chlamydomonas reinhardtii
Oxyrrhis marina
Amphidinium carterae
Karlodinium veneficum
Heterocapsa triquetra
Isochrysis galbana
Emiliania huxleyi
Chrysochromulina sp.
Salpingoeca rosetta
Monosiga brevicollis
Thalassiosira pseudonana
Thalassiosira oceanica
Sterkiella histriomuscorum
Phaeodactylum tricornutum
Thalassionema nitzschioides
Aureococcus anophagefferens
Ectocarpus siliculosus
Phytophthora parasitica
Spumella sp.
Cyclotella cryptica
Mallomonas rasilis
Dinophysis acuminata CAMPEP_0179255948
Dinophysis acuminata CAMPEP_0179361658
0.6
I
Staphylothermus marinus
Pyrolobus fumarii
Synechococcus sp.
Prochlorococcus marinus
Reticulomyxa filosa
Eucyrtidium acuminatum
Goniomonas sp.
Ostreococcus tauri
Chlamydomonas reinhardtii
Oxyrrhis marina
Amphidinium carterae
Karlodinium veneficum
Heterocapsa triquetra
Isochrysis galbana
Emiliania huxleyi
Chrysochromulina sp.
Salpingoeca rosetta
Monosiga brevicollis
Thalassiosira pseudonana
Thalassiosira oceanica
Thalassionema nitzschioides
Phaeodactylum tricornutum
Aureococcus anophagefferens
Durinskia baltica CAMPEP_0170355688
Spumella sp.
Cyclotella cryptica
Mallomonas rasilis
Ectocarpus siliculosus
Phytophthora parasitica
Durinskia baltica CAMPEP_0170215334
Sterkiella histriomuscorum
0.6

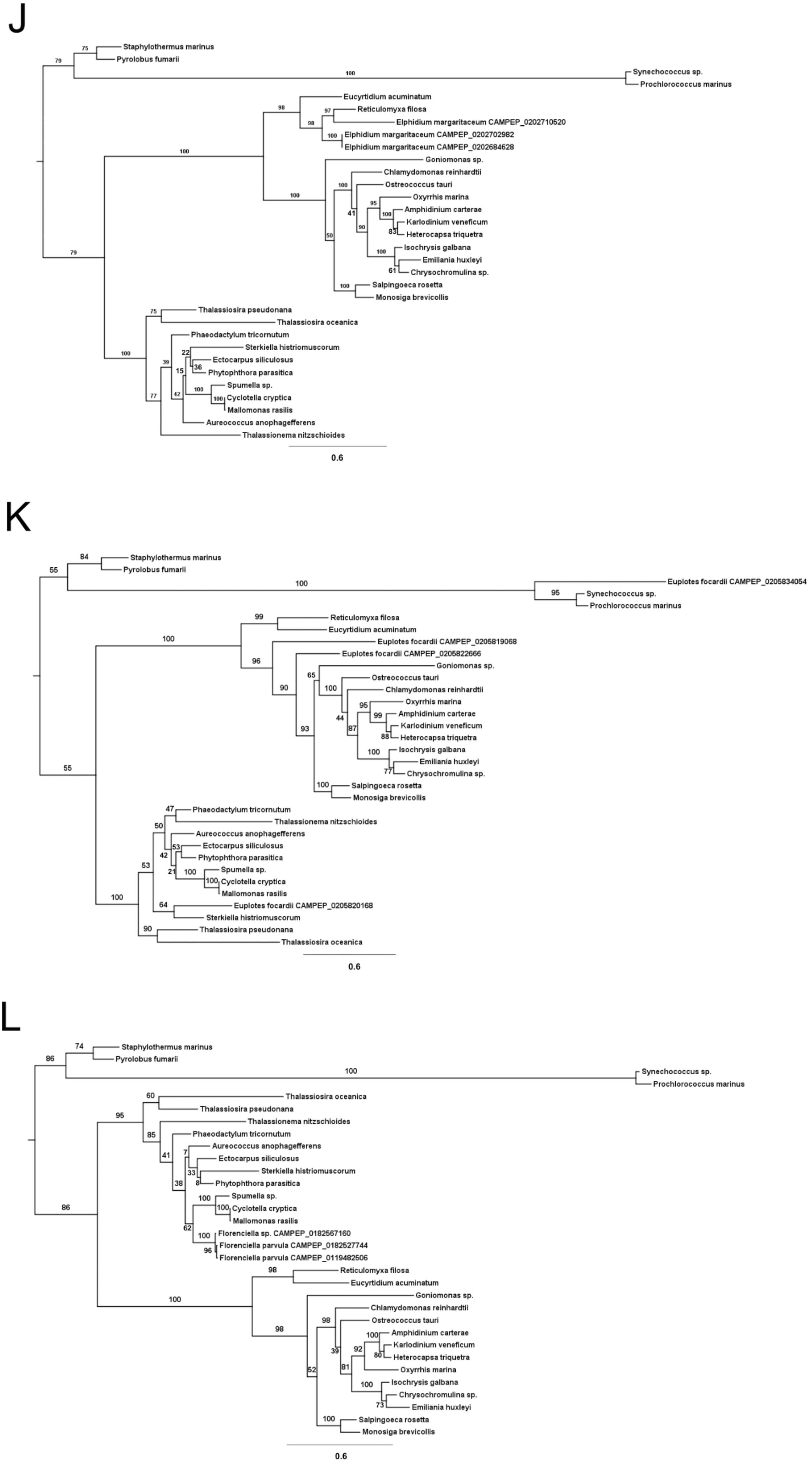
J
Staphylothermus marinus
Pyrolobus fumarii
Synechococcus sp.
Prochlorococcus marinus
Eucyrtidium acuminatum
Reticulomyxa filosa
Elphidium margaritaceum CAMPEP_0202710520
Elphidium margaritaceum CAMPEP_0202702982
Elphidium margaritaceum CAMPEP_0202684628
Goniomonas sp.
Chlamydomonas reinhardtii
Ostreococcus tauri
Oxyrrhis marina
Amphidinium carterae
Karlodinium veneficum
Heterocapsa triquetra
Isochrysis galbana
Emiliania huxleyi
Chrysochromulina sp.
Salpingoeca rosetta
Monosiga brevicollis
Thalassiosira pseudonana
Thalassiosira oceanica
Phaeodactylum tricornutum
Sterkiella histriomuscorum
Ectocarpus siliculosus
Phytophthora parasitica
Spumella sp.
Cyclotella cryptica
Mallomonas rasilis
Aureococcus anophagefferens
Thalassionema nitzschioides
0.6
K
Staphylothermus marinus
Pyrolobus fumarii
Euplotes focardii CAMPEP_0205834054
Synechococcus sp.
Prochlorococcus marinus
Reticulomyxa filosa
Eucyrtidium acuminatum
Euplotes focardii CAMPEP_0205819068
Euplotes focardii CAMPEP_0205822666
Goniomonas sp.
Ostreococcus tauri
Chlamydomonas reinhardtii
Oxyrrhis marina
Amphidinium carterae
Karlodinium veneficum
Heterocapsa triquetra
Isochrysis galbana
Emiliania huxleyi
Chrysochromulina sp.
Salpingoeca rosetta
Monosiga brevicollis
Phaeodactylum tricornutum
Thalassionema nitzschioides
Aureococcus anophagefferens
Ectocarpus siliculosus
Phytophthora parasitica
Spumella sp.
Cyclotella cryptica
Mallomonas rasilis
Euplotes focardii CAMPEP_0205820168
Sterkiella histriomuscorum
Thalassiosira pseudonana
Thalassiosira oceanica
0.6
L
Staphylothermus marinus
Pyrolobus fumarii
Synechococcus sp.
Prochlorococcus marinus
Thalassiosira oceanica
Thalassiosira pseudonana
Thalassionema nitzschioides
Phaeodactylum tricornutum
Aureococcus anophagefferens
Ectocarpus siliculosus
Sterkiella histriomuscorum
Phytophthora parasitica
Spumella sp.
Cyclotella cryptica
Mallomonas rasilis
Florenciella sp. CAMPEP_0182567160
Florenciella parvula CAMPEP_0182527744
Florenciella parvula CAMPEP_0119482506
Reticulomyxa filosa
Eucyrtidium acuminatum
Goniomonas sp.
Chlamydomonas reinhardtii
Ostreococcus tauri
Amphidinium carterae
Karlodinium veneficum
Heterocapsa triquetra
Oxyrrhis marina
Isochrysis galbana
Chrysochromulina sp.
Emiliania huxleyi
Salpingoeca rosetta
Monosiga brevicollis
0.6

M

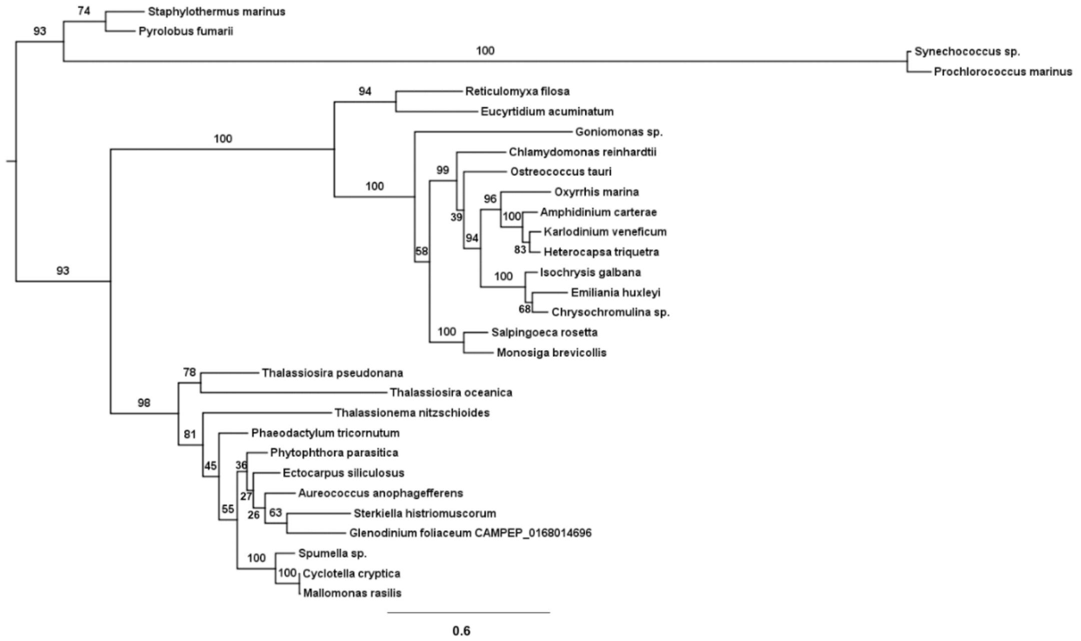
Staphylothermus marinus
Pyrolobus fumarii
Synechococcus sp.
Prochlorococcus marinus
Reticulomyxa filosa
Eucyrtidium acuminatum
Goniomonas sp.
Chlamydomonas reinhardtii
Ostreococcus tauri
Oxyrrhis marina
Amphidinium carterae
Karlodinium veneficum
Heterocapsa triquetra
Isochrysis galbana
Emiliania huxleyi
Chrysochromulina sp.
Salpingoeca rosetta
Monosiga brevicollis
Thalassiosira pseudonana
Thalassiosira oceanica
Thalassionema nitzschioides
Phaeodactylum tricornutum
Phytophthora parasitica
Ectocarpus siliculosus
Aureococcus anophagefferens
Sterkiella histriomuscorum
Glenodinium foliaceum CAMPEP_0168014696
Spumella sp.
Cyclotella cryptica
Mallomonas rasilis
0.6

N

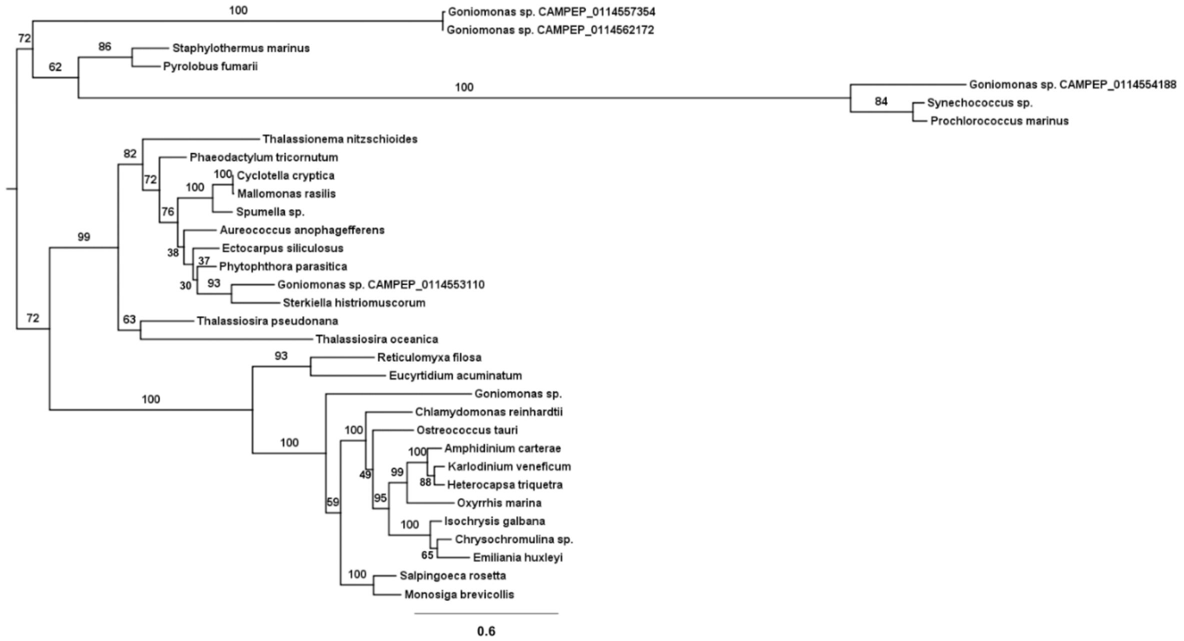
Goniomonas sp. CAMPEP_0114557354
Goniomonas sp. CAMPEP_0114562172
Staphylothermus marinus
Pyrolobus fumarii
Goniomonas sp. CAMPEP_0114554188
Synechococcus sp.
Prochlorococcus marinus
Thalassionema nitzschioides
Phaeodactylum tricornutum
Cyclotella cryptica
Mallomonas rasilis
Spumella sp.
Aureococcus anophagefferens
Ectocarpus siliculosus
Phytophthora parasitica
Goniomonas sp. CAMPEP_0114553110
Sterkiella histriomuscorum
Thalassiosira pseudonana
Thalassiosira oceanica
Reticulomyxa filosa
Eucyrtidium acuminatum
Goniomonas sp.
Chlamydomonas reinhardtii
Ostreococcus tauri
Amphidinium carterae
Karlodinium veneficum
Heterocapsa triquetra
Oxyrrhis marina
Isochrysis galbana
Chrysochromulina sp.
Emiliania huxleyi
Salpingoeca rosetta
Monosiga brevicollis
0.6

O

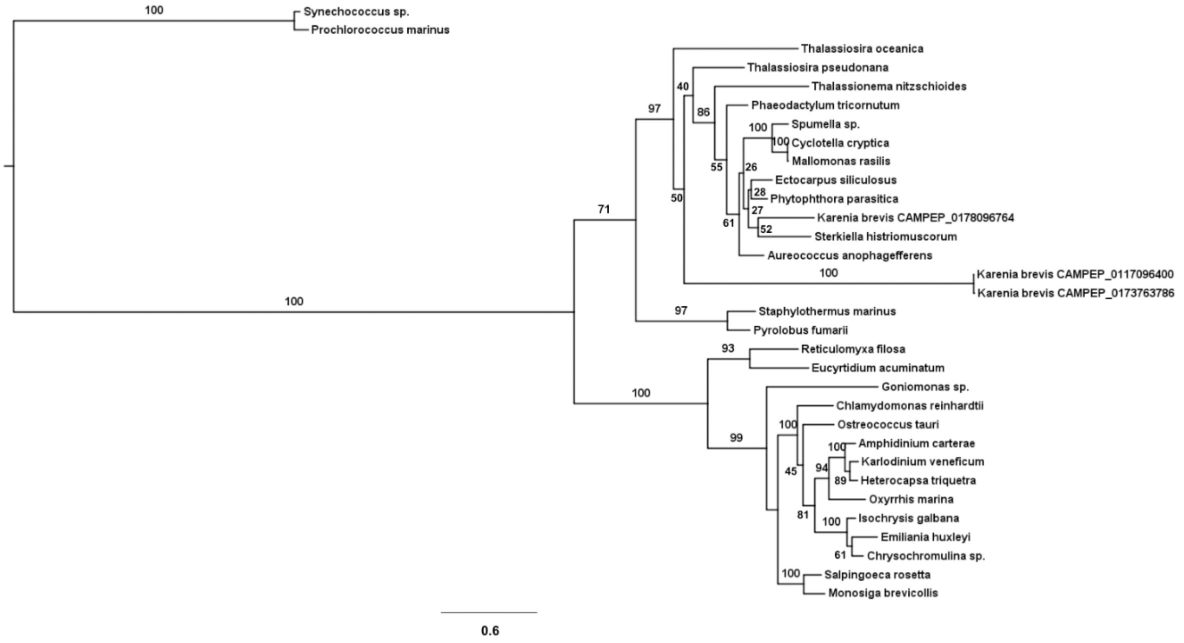
Synechococcus sp.
Prochlorococcus marinus
Thalassiosira oceanica
Thalassiosira pseudonana
Thalassionema nitzschioides
Phaeodactylum tricornutum
Spumella sp.
Cyclotella cryptica
Mallomonas rasilis
Ectocarpus siliculosus
Phytophthora parasitica
Karenia brevis CAMPEP_0178096764
Sterkiella histriomuscorum
Aureococcus anophagefferens
Karenia brevis CAMPEP_0117096400
Karenia brevis CAMPEP_0173763786
Staphylothermus marinus
Pyrolobus fumarii
Reticulomyxa filosa
Eucyrtidium acuminatum
Goniomonas sp.
Chlamydomonas reinhardtii
Ostreococcus tauri
Amphidinium carterae
Karlodinium veneficum
Heterocapsa triquetra
Oxyrrhis marina
Isochrysis galbana
Emiliania huxleyi
Chrysochromulina sp.
Salpingoeca rosetta
Monosiga brevicollis
0.6

Q

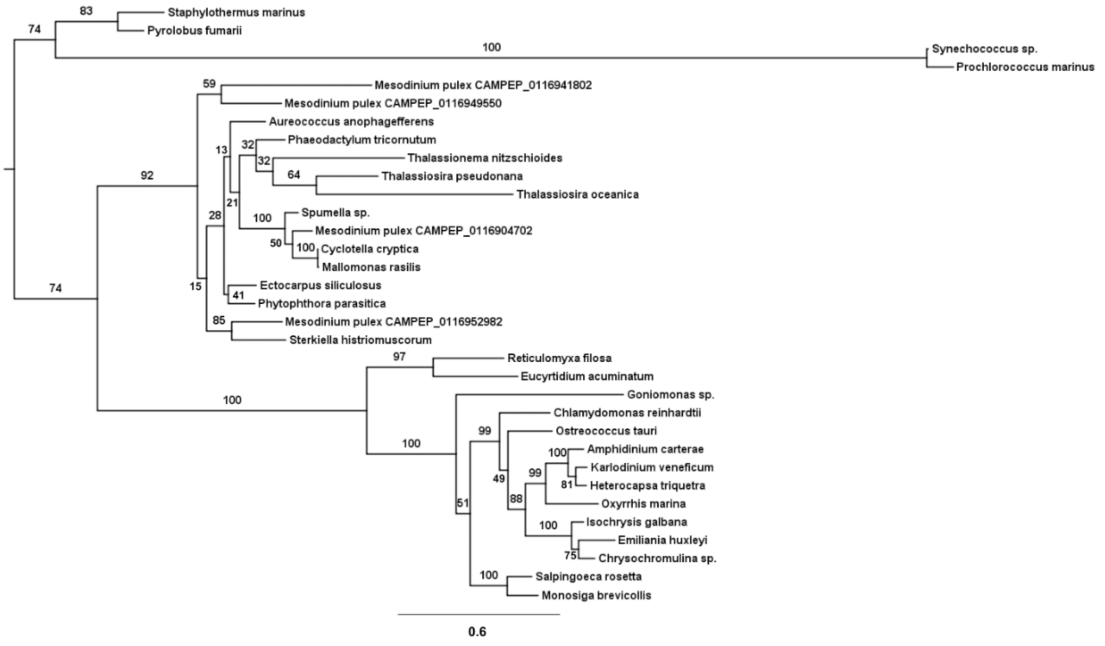
Staphylothermus marinus
Pyrolobus fumarii
Synechococcus sp.
Prochlorococcus marinus
Mesodinium pulex CAMPEP_0116941802
Mesodinium pulex CAMPEP_0116949550
Aureococcus anophagefferens
Phaeodactylum tricornutum
Thalassionema nitzschioides
Thalassiosira pseudonana
Thalassiosira oceanica
Spumella sp.
Mesodinium pulex CAMPEP_0116904702
Cyclotella cryptica
Mallomonas rasilis
Ectocarpus siliculosus
Phytophthora parasitica
Mesodinium pulex CAMPEP_0116952982
Sterkiella histriomuscorum
Reticulomyxa filosa
Eucyrtidium acuminatum
Goniomonas sp.
Chlamydomonas reinhardtii
Ostreococcus tauri
Amphidinium carterae
Karlodinium veneficum
Heterocapsa triquetra
Oxyrrhis marina
Isochrysis galbana
Emiliania huxleyi
Chrysochromulina sp.
Salpingoeca rosetta
Monosiga brevicollis
0.6

R

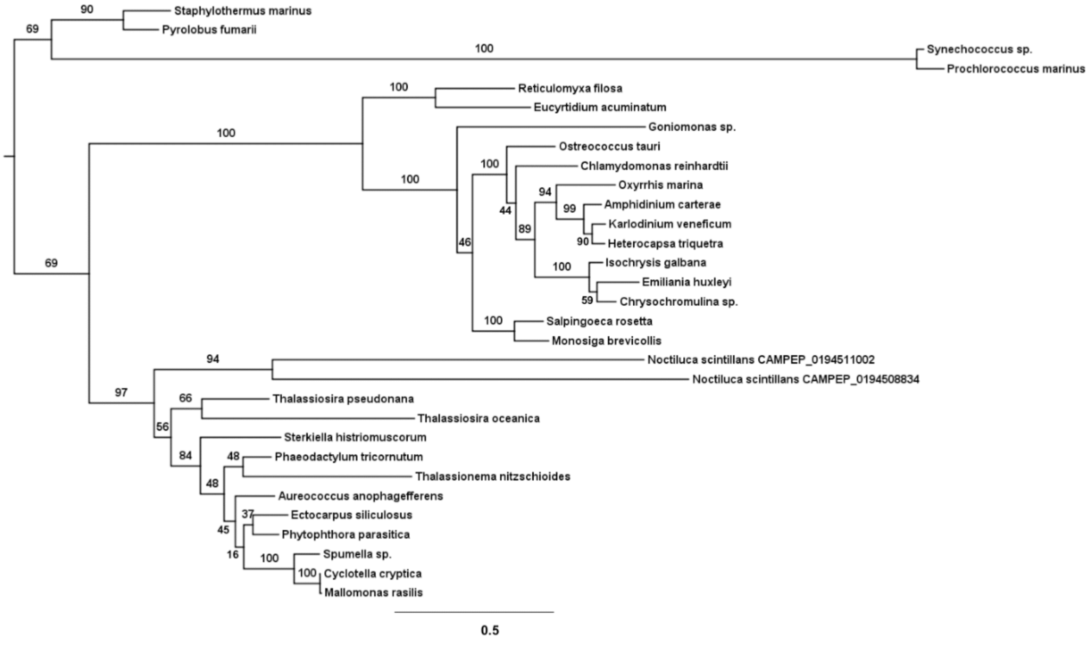
Staphylothermus marinus
Pyrolobus fumarii
Synechococcus sp.
Prochlorococcus marinus
Reticulomyxa filosa
Eucyrtidium acuminatum
Goniomonas sp.
Ostreococcus tauri
Chlamydomonas reinhardtii
Oxyrrhis marina
Amphidinium carterae
Karlodinium veneficum
Heterocapsa triquetra
Isochrysis galbana
Emiliania huxleyi
Chrysochromulina sp.
Salpingoeca rosetta
Monosiga brevicollis
Noctiluca scintillans CAMPEP_0194511002
Noctiluca scintillans CAMPEP_0194508834
Thalassiosira pseudonana
Thalassiosira oceanica
Sterkiella histriomuscorum
Phaeodactylum tricornutum
Thalassionema nitzschioides
Aureococcus anophagefferens
Ectocarpus siliculosus
Phytophthora parasitica
Spumella sp.
Cyclotella cryptica
Mallomonas rasilis
0.5

S

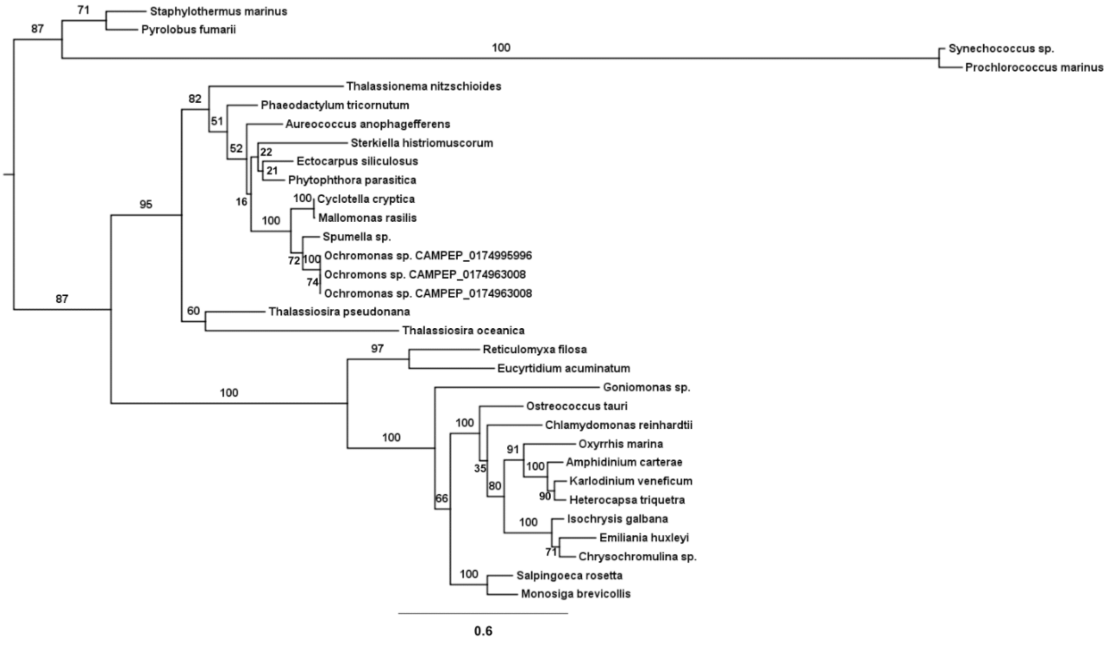
Staphylothermus marinus
Pyrolobus fumarii
Synechococcus sp.
Prochlorococcus marinus
Thalassionema nitzschioides
Phaeodactylum tricornutum
Aureococcus anophagefferens
Sterkiella histriomuscorum
Ectocarpus siliculosus
Phytophthora parasitica
Cyclotella cryptica
Mallomonas rasilis
Spumella sp.
Ochromonas sp. CAMPEP_0174995996
Ochromons sp. CAMPEP_0174963008
Ochromonas sp. CAMPEP_0174963008
Thalassiosira pseudonana
Thalassiosira oceanica
Reticulomyxa filosa
Eucyrtidium acuminatum
Goniomonas sp.
Ostreococcus tauri
Chlamydomonas reinhardtii
Oxyrrhis marina
Amphidinium carterae
Karlodinium veneficum
Heterocapsa triquetra
Isochrysis galbana
Emiliania huxleyi
Chrysochromulina sp.
Salpingoeca rosetta
Monosiga brevicollis
0.6

T

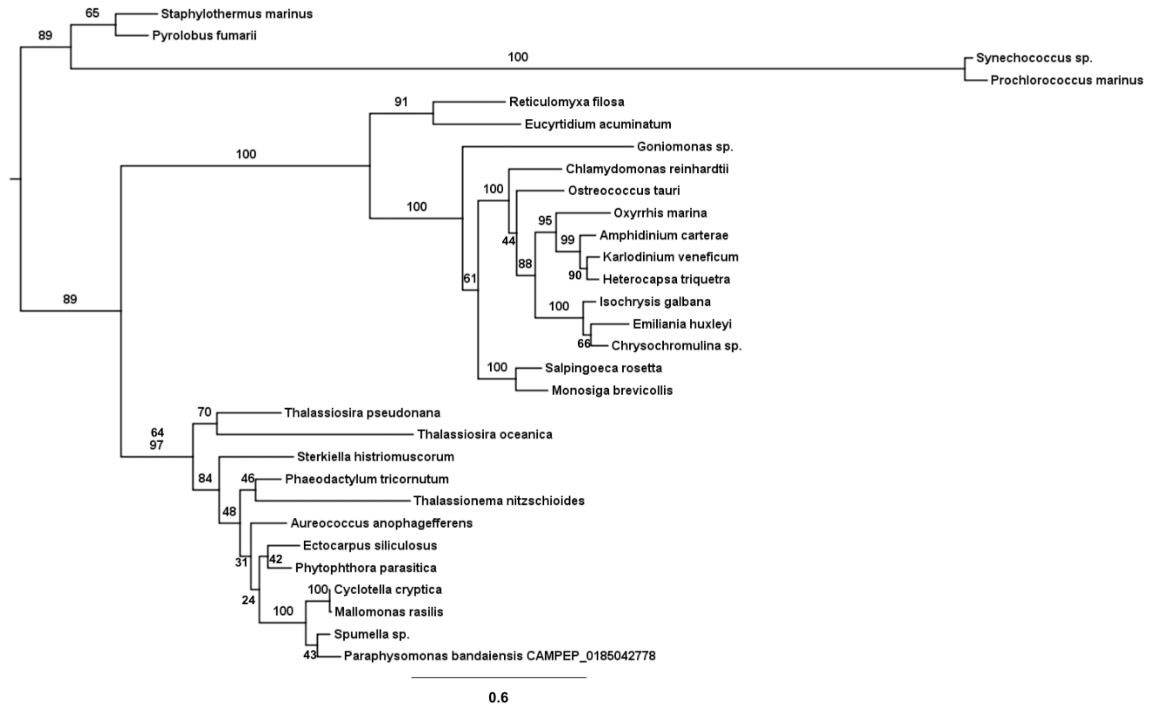

Staphylothermus marinus
Pyrolobus fumarii
Synechococcus sp.
Prochlorococcus marinus
Reticulomyxa filosa
Eucyrtidium acuminatum
Goniomonas sp.
Chlamydomonas reinhardtii
Ostreococcus tauri
Oxyrrhis marina
Amphidinium carterae
Karlodinium veneficum
Heterocapsa triquetra
Isochrysis galbana
Emiliania huxleyi
Chrysochromulina sp.
Salpingoeca rosetta
Monosiga brevicollis
Thalassiosira pseudonana
Thalassiosira oceanica
Sterkiella histriomuscorum
Phaeodactylum tricornutum
Thalassionema nitzschioides
Aureococcus anophagefferens
Ectocarpus siliculosus
Phytophthora parasitica
Cyclotella cryptica
Mallomonas rasilis
Spumella sp.
Paraphysomonas bandaiensis CAMPEP_0185042778
0.6

U

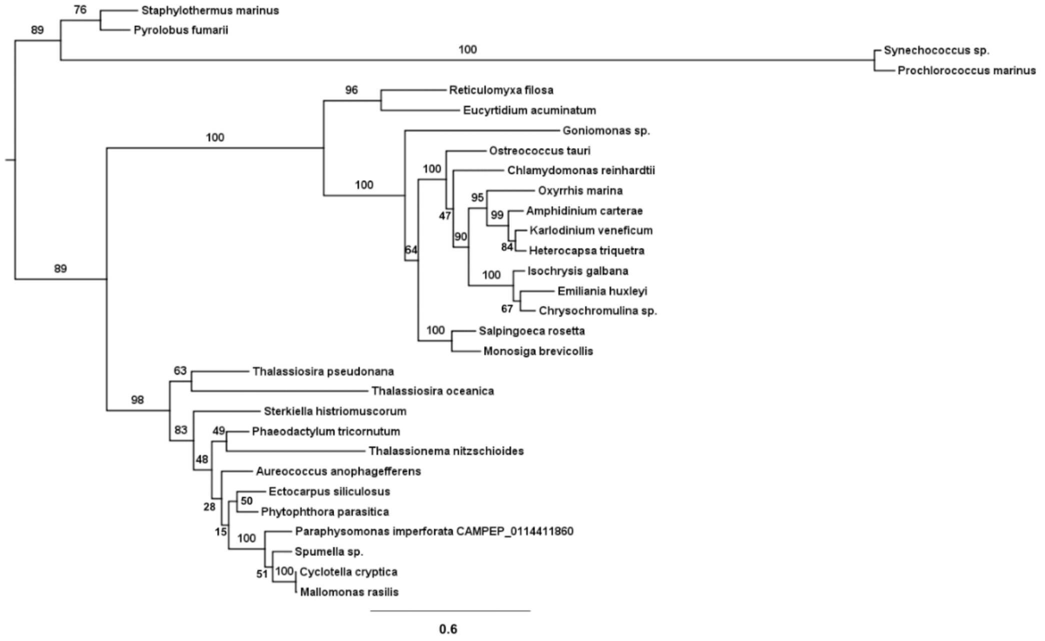

Staphylothermus marinus
Pyrolobus fumarii
Synechococcus sp.
Prochlorococcus marinus
Reticulomyxa filosa
Eucyrtidium acuminatum
Goniomonas sp.
Ostreococcus tauri
Chlamydomonas reinhardtii
Oxyrrhis marina
Amphidinium carterae
Karlodinium veneficum
Heterocapsa triquetra
Isochrysis galbana
Emiliania huxleyi
Chrysochromulina sp.
Salpingoeca rosetta
Monosiga brevicollis
Thalassiosira pseudonana
Thalassiosira oceanica
Sterkiella histriomuscorum
Phaeodactylum tricornutum
Thalassionema nitzschioides
Aureococcus anophagefferens
Ectocarpus siliculosus
Phytophthora parasitica
Paraphysomonas imperforata CAMPEP_0114411860
Spumella sp.
Cyclotella cryptica
Mallomonas rasilis
0.6

V

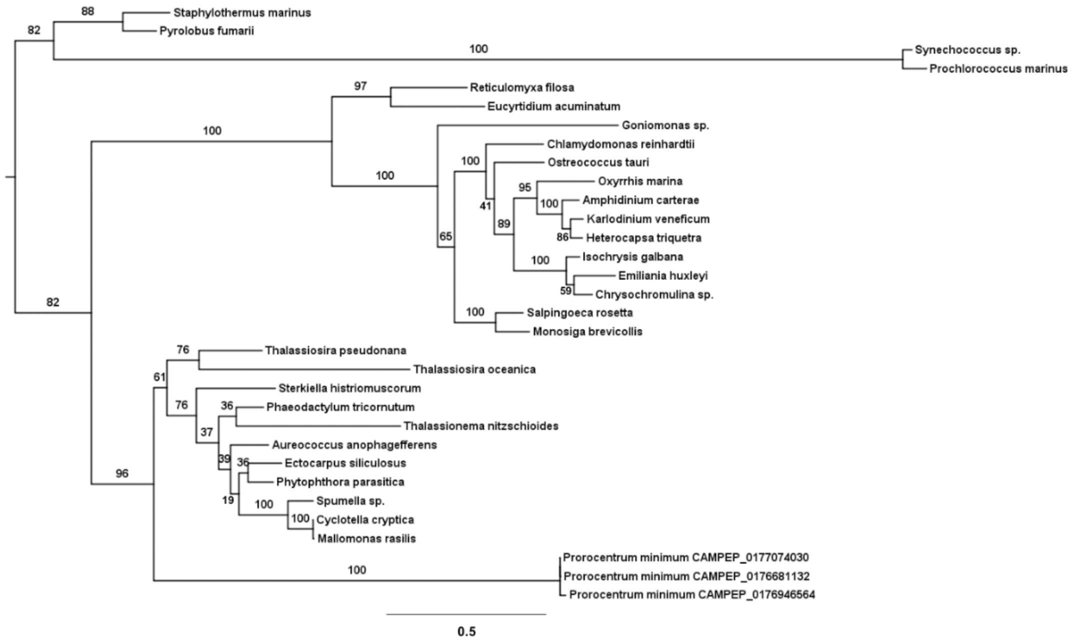

Staphylothermus marinus
Pyrolobus fumarii
Synechococcus sp.
Prochlorococcus marinus
Reticulomyxa filosa
Eucyrtidium acuminatum
Goniomonas sp.
Chlamydomonas reinhardtii
Ostreococcus tauri
Oxyrrhis marina
Amphidinium carterae
Karlodinium veneficum
Heterocapsa triquetra
Isochrysis galbana
Emiliania huxleyi
Chrysochromulina sp.
Salpingoeca rosetta
Monosiga brevicollis
Thalassiosira pseudonana
Thalassiosira oceanica
Sterkiella histriomuscorum
Phaeodactylum tricornutum
Thalassionema nitzschioides
Aureococcus anophagefferens
Ectocarpus siliculosus
Phytophthora parasitica
Spumella sp.
Cyclotella cryptica
Mallomonas rasilis
Prorocentrum minimum CAMPEP_0177074030
Prorocentrum minimum CAMPEP_0176681132
Prorocentrum minimum CAMPEP_0176946564
0.5

W

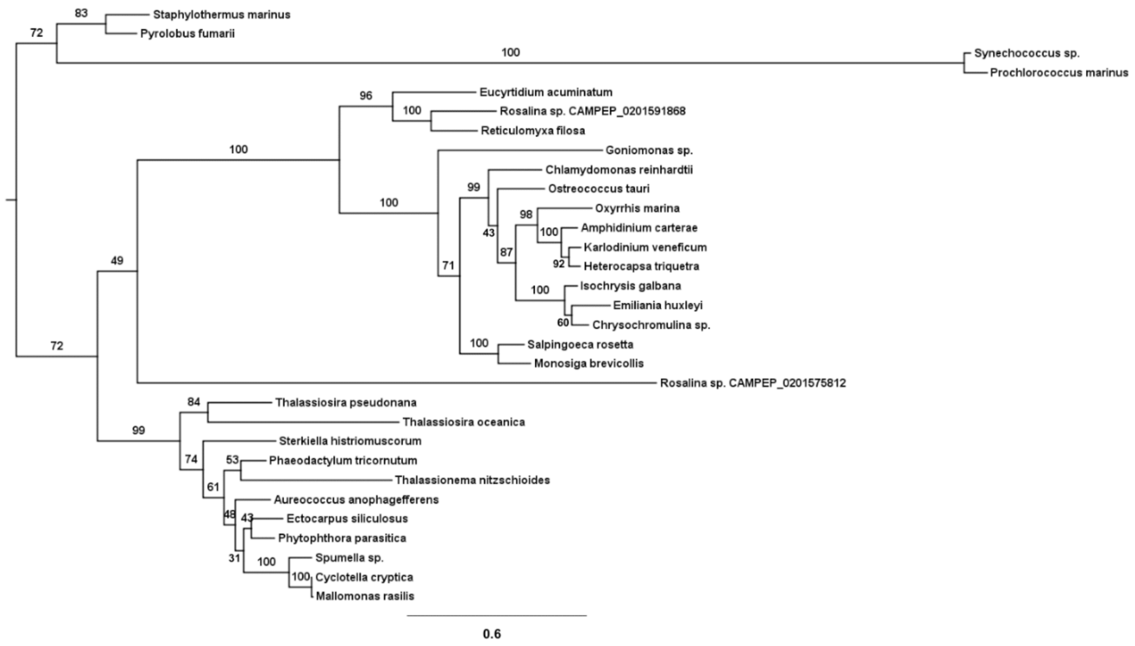

Staphylothermus marinus
Pyrolobus fumarii
Synechococcus sp.
Prochlorococcus marinus
Eucyrtidium acuminatum
Rosalina sp. CAMPEP_0201591868
Reticulomyxa filosa
Goniomonas sp.
Chlamydomonas reinhardtii
Ostreococcus tauri
Oxyrrhis marina
Amphidinium carterae
Karlodinium veneficum
Heterocapsa triquetra
Isochrysis galbana
Emiliania huxleyi
Chrysochromulina sp.
Salpingoeca rosetta
Monosiga brevicollis
Rosalina sp. CAMPEP_0201575812
Thalassiosira pseudonana
Thalassiosira oceanica
Sterkiella histriomuscorum
Phaeodactylum tricornutum
Thalassionema nitzschioides
Aureococcus anophagefferens
Ectocarpus siliculosus
Phytophthora parasitica
Spumella sp.
Cyclotella cryptica
Mallomonas rasilis
0.6

X

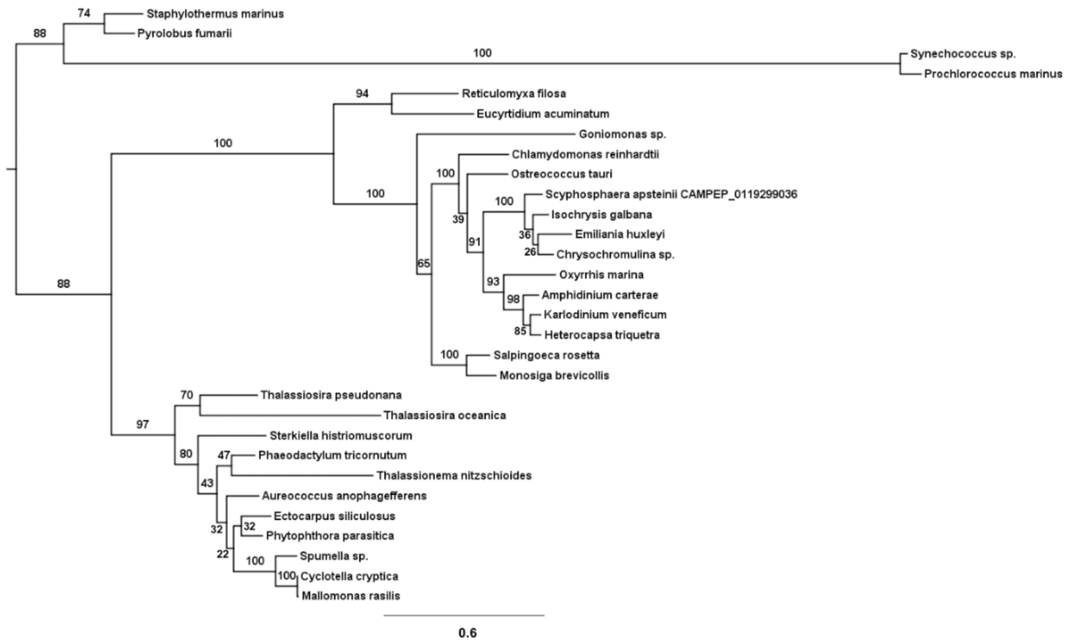

Staphylothermus marinus
Pyrolobus fumarii
Synechococcus sp.
Prochlorococcus marinus
Reticulomyxa filosa
Eucyrtidium acuminatum
Goniomonas sp.
Chlamydomonas reinhardtii
Ostreococcus tauri
Scyphosphaera apsteinii CAMPEP_0119299036
Isochrysis galbana
Emiliania huxleyi
Chrysochromulina sp.
Oxyrrhis marina
Amphidinium carterae
Karlodinium veneficum
Heterocapsa triquetra
Salpingoeca rosetta
Monosiga brevicollis
Thalassiosira pseudonana
Thalassiosira oceanica
Sterkiella histriomuscorum
Phaeodactylum tricornutum
Thalassionema nitzschioides
Aureococcus anophagefferens
Ectocarpus siliculosus
Phytophthora parasitica
Spumella sp.
Cyclotella cryptica
Mallomonas rasilis
0.6

Y

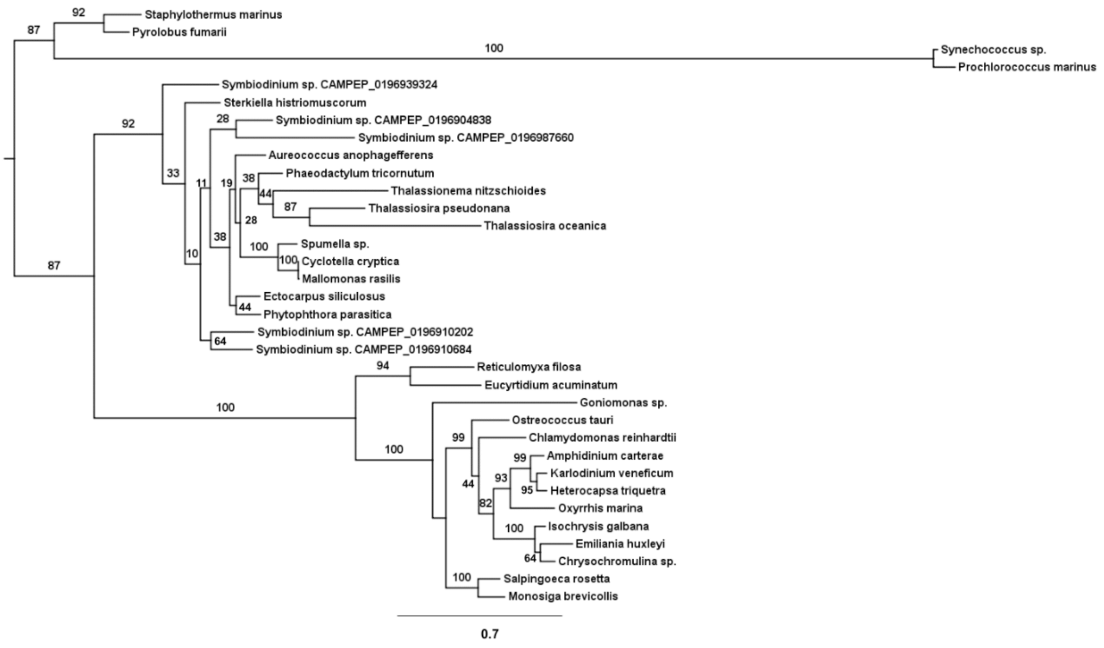

Staphylothermus marinus
Pyrolobus fumarii
Synechococcus sp.
Prochlorococcus marinus
Symbiodinium sp. CAMPEP_0196939324
Sterkiella histriomuscorum
Symbiodinium sp. CAMPEP_0196904838
Symbiodinium sp. CAMPEP_0196987660
Aureococcus anophagefferens
Phaeodactylum tricornutum
Thalassionema nitzschioides
Thalassiosira pseudonana
Thalassiosira oceanica
Spumella sp.
Cyclotella cryptica
Mallomonas rasilis
Ectocarpus siliculosus
Phytophthora parasitica
Symbiodinium sp. CAMPEP_0196910202
Symbiodinium sp. CAMPEP_0196910684
Reticulomyxa filosa
Eucyrtidium acuminatum
Goniomonas sp.
Ostreococcus tauri
Chlamydomonas reinhardtii
Amphidinium carterae
Karlodinium veneficum
Heterocapsa triquetra
Oxyrrhis marina
Isochrysis galbana
Emiliania huxleyi
Chrysochromulina sp.
Salpingoeca rosetta
Monosiga brevicollis
0.7

Z

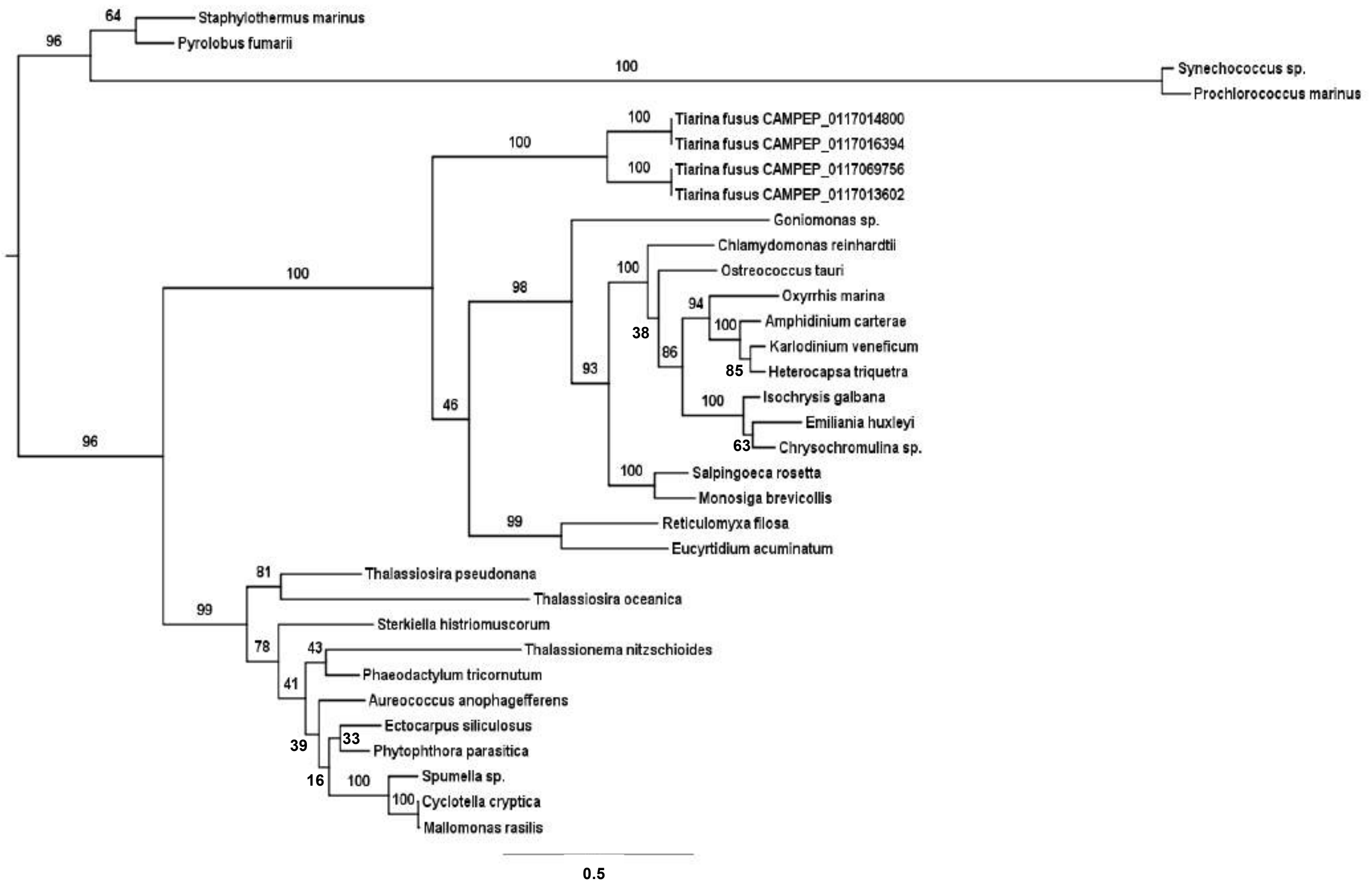

SI Figure 3. Contamination testing using EF-1 phylogenies. Sequences used are listed in Supplementary Table 3. Contamination was presumed if MMETSP sequences branch as with eukaryotes but distant to taxonomically relevant sequences, and as sister to stramenopiles. Trees were generated by maximum likelihood analysis using PhyML, using the LG+G model. Numbers at nodes are a percentage of 100 bootstrap replicates. Scale bar indicates average number of amino acid substitutions per site. (A) Reference Dataset tree, (B) *Ammonia* sp. (C) *Ceratium fusus* (D) *Chrysochromulina* sp. (E) *Coccolithus pelagicus* (F) *Dictyocha speculum* (G) *Dinobryon* sp. (H) *Dinophysis acuminata* (I) *Durinskia baltica* (J) *Elphidium margaritaceum* (K) *Euplotes focardii* (L) *Florenciella parvula* (M) *Glenodinium foliaceum* (N) *Karenia brevis* (O) *Kryptoperidinium foliaceum* (P) *Mallomonas* sp. (Q) *Mesodinium pulex* (R) *Noctiluca scintillans* (S) *Ochromonas* sp. (T) *Paraphysomonas bandaiensis* (U) *Paraphysomonas imperforata* (V) *Prorocentrum minimum* (W) *Rosalina* sp. (X) *Scyphosphaera apsteinii* (Y) *Symbiodinium* sp. (Z) *Tiarina fusus*
