# Supplementary material for: The Evolution of Silicon Transport in Eukaryotes
Source: Mol Biol Evol. 2016 Oct 11;33(12):3226–48. doi: 10.1093/molbev/msw209 (PMC5100055; doi:10.1093/molbev/msw209)
Supplement: Supplementary Data [file supp_msw209_suppl_data.zip › Supplementary_Figures_1-2.pdf]

```
                                            TM1                                     TM2
Rhodococcus opacus B4 SIT-L                  FRREIVSLSFTRLVAVTGNNPVTAAA------------------------------AAVR
Rhodococcus opacus SIT-L                     FRREIVSLSFTRLVAVTGNNPVTAAA------------------------------AAVR
Synechococcus KORDI                          ------------------------------------------------------------
Synechococcus CC9616 SIT-L                   ------------------------------------------------------------
TARA_004 SRF 0.22-1.6                        ------------------------------------------------------------
TARA_142 SRF 0.22-3                          ------------------------------------------------------------
Florenciella parvula SIT-L                   LGGSNKQAEVSGAVVAKAGSPTPAVK----------------------------------
Phaeodaria OSH121 SIT-L1                     ------------------------------------------------------------
Phaeodaria OSH121 SIT-L2                     ------------------------------------------------------------
Ceratium fusus SIT-L                         ------------------------------------------------------------
Dinophysis acuminata SIT-L                   ------------------------------------------------------------
Calanus finmarchicus SIT-L                   ------------------------------------------------------------
Platynereis dumerilii SIT-L                  ----------------------SRRR----------------------------------
Capitella teleta SIT-L                       HYAPPVEIYSSGFINNKEGYAAWVSQ----------------------------------
Ciona intestinalis SIT-L                     ------------------------------------------------------------
Molgula tectiformis SIT-L                    -------------------------R----------------------------------
Phallusia mammillata SIT-L                   ------------------------------------------------------------
Scyphosphaera apsteinii SIT-L                ------------------------------------------------------------
Coccolithus pelagicus SIT-L                  ---------GLLLMINTTNTQMPSND----------------------------------
Calcidiscus leptoporus SIT-L                 ------------------------------------------------------------
Rosalina sp. SIT-L                           FVESMSEFEAGLIKTHGANGSNPKQR---------------------------------L
Elphidium margaritaceum SIT-L                ------------------------------------------------------------
Ammonia sp. SIT-L1                           -MSSARSPRSPGEPSMTDFSASHLDV----------------------------------
Ammonia sp. SIT-L2                           KRKLLGNSSPGVSLADAGYGASDASN------------------------------LPIK
Savillea parva SITBeta                       YVWSTGLLIFCFAIILYGIGAEWNHP--------PWDQGSVHPAFEVILFLILLFWSAIL
Acanthoeca spectabilis SITBeta               YVWSTAVLVFSLVIVYYGIGAEWNNP--------PWDQGSTHPVFESCLFSILLFWVCIL
Helgoeca nana SITBeta                        YFWSTALLIFSLIVVFYGIGSKWNNP--------PWGQGVIHPAIEAVIFVVLITWIALL
Stephanoeca diplocostata SITAlphaBC          YYWSMVLLIFATVIMIYTIAKEWTNP--------PWN--YTHPVSEIFIFLLLLSWIALL
Stephanoeca diplocostata SITAlphaA           YVWSMVLLIFATVIMIYTIAKEWTNP--------PWN--YTNPAGEIIIFLLLLTWIALL
Acanthoeca spectabilis SITAlpha              YWWGSALTLFSFIIMIYTISKQWNNP--------PWSQTKSHPVLELICFFFMLGWIALL
Helgoeca nana SITAlpha                       YVWSTLLLIFAMTVTLYGIGKQWNNP--------PWSQTNSHPVLEIGFFFFMCTWIGLL
Savillea parva SITAlpha                      YVWSTLLLIFAMVVHIYGIAKQWNNP--------PWSQTYSHPVLEILFFLFMCSWIGLL
Diaphanoeca grandis SITAlpha                 YWWSSALLIFALFVVFYGMGKQWNNP--------PWDPSDSHPALDICLFIVMLTWIALL
Diplotheca costata SITAlpha                  YVWSMALLGFALFVVVYGMSRQWTNP--------PWHPRDGHPALEITLFFVMLFWIALL
Stephanoeca diplocostata SITBeta             YVWATILLAFALVVVYYQMGQDWTNP--------PWGPDKSPVWINIVIFTLLLFWIMLL
Diaphanoeca grandis SITBeta                  YCWSLFLLVFALFVTYYDLAMGWTNDFDD----LPHRSEPYMVFINIFLFTIFLFWVALL
Diplotheca costata SITBeta                   YFWSTGLLIFAIFVMYYDIAKDWANDFDDNSEPGEGAGSRTPVAINIIFVTILLFWVAIL
Prymnesium neolepis SIT                      YFMSAGAVGFFLAILFDIACGPGRKL------------EALHPFPIILIWWFMLFYLSCL
Scyphosphaera apsteinii SIT                  YWYSAAAVAFCFAILFVIVGKEESKI-------------GIHGVPAVLIWWFMLFYLSCL
Phaeodactylum tricornutum SIT3               CVYSMILLVFSIIVVLAAIFSEQTVA--------TG-ENNVNPVLAVFLVCFLITWLAMM
Synedra acus SIT                             FIYSSALLVLCLIIVHAAMFTTQTTS--------GA-DFGLHPAVSFLIFWFLIIWLAMM
Phaeodactylum tricornutum SIT2               CAYSVGLLIFSTIIIMGLIFNEETKL--------S---SDVHSAVAFIAIWVGVLWLTMV
Phaeodactylum tricornutum SIT1               CAYSVGLLVFSTIIIMGLIFNEETKL--------S---SDVNSAVAFVAIWGGVLWLTMV
Cylindrotheca fusiformis SIT3                QFYSMALVIFSVVIVTALMFTDNTKM--------A---KDAHPVAALVIMWLGILWMSMV
Cylindrotheca fusiformis SIT1                QFYSMALVIFSVVIVTALMFTDNTKL--------A---KDAHPVAALVIMWLGILWMSMV
Cylindrotheca fusiformis SIT5                QFYSMALVIFSVVIVTALMFTDNTKM--------A---QDAHPVAALVIMWLGILWMSMV
Cylindrotheca fusiformis SIT4                QAYSMCLVIFSVVIVTALMFTDNTKL--------A---KDAHPVAALVIMWLGILWMSMV
Cylindrotheca fusiformis SIT2                QAYSMCLVIFSVVIVTALMFTDNTKL--------A---KDAHPVAALVIMWLGILWMSMV
Nitzschia alba SIT                           MAYSMGLVIFSVIIVTALMFEKDTKI--------A---NDVHPAAALVIMWAGILWMSMV
Thalassiosira pseudonana SIT2                ------------------MFTGNTRV--------A---KDANPWVALIVCILAIVWLSMI
Thalassiosira pseudonana SIT1                YIGSIGLLIFSIIIVGALMFTGNTRV--------A---KDANPWVSLIVCILAIVWLSMI
Skeletonema costatum SIT                     YTYSVILLIFSIVLVVSLMFTGNTKL--------A---ADASPWAALFVCIAAVVWLSMI
Thalassiosira pseudonana SIT3                YTYSLALLAFSIILIVSVIFNQGTKL------------STVNPWLALCVMVGTIVWLGMM
Dinobryon sp. SIT                            LVLSTLVVTILLVVIFVGVSLTYCVL-------------KIFPLVNFLLMIGCLTLLAYC
Mallomonas sp. SIT*                          ------------------------------------------------------------
Ochromonas distigma SIT                      YILSSALTLTYVIYILWGIFDGQAVL-------------PVHPVVNFIIFCFCMTLVAYL
Ochromonas sp. SIT                           --------MTYVIYILWGIWSGQAVL-------------PVPPVVSFIIFCFCMTLVAYL
Paraphysomonas bandaiensis SIT1              YIVSSGLTLTYVVYILWGIWTNQAVL-------------PGPPIAHFLIFLFCMTLVAYL
Paraphysomonas imperforata SIT1              YAFSSAMTVTYLIYIMWGIWTHQAVL-------------PGHFVFHFIVFLFCLTLVAYL
Paraphysomonas imperforata SIT2              YIISTLVTVFYCSVVFHGIAKDLNVA------------DFGGPIPEFLIFTLCIILLAVN
Paraphysomonas bandaiensis SIT2              YFVSTVGIVLSCIIVFTGIGADYNVA------------DFGGPVLEFITFFLALLLLAMN
Noctiluca scintillans SIT-L                  ----------TAMEHPWPDRSERKIA----------------------------------
                                            TM1                                     TM2
```

TM3

```
Rhodococcus opacus B4 SIT-L                   EAVAAKGIDEDT-LNAML---RTVPARKTDADAIHY------CFNTAAPVPTRA------
Rhodococcus opacus SIT-L                      EAVAAKGIDEDT-LNAML---RTVPARKTDADAIHY------CFNTAAPVPTRA------
Synechococcus KORDI                           ------------------------------------------------------------
Synechococcus CC9616 SIT-L                    ------------------------------------------------------------
TARA_004 SRF 0.22-1.6                         ------------------------------------------------------------
TARA_142 SRF 0.22-3                           ------------------------------------------------------------
Florenciella parvula SIT-L                    ------------------------------------------------------------
Phaeodaria OSH121 SIT-L1                      ------------------------------------------------------------
Phaeodaria OSH121 SIT-L2                      ------------------------------------------------------------
Ceratium fusus SIT-L                          ------------------------------------------------------------
Dinophysis acuminata SIT-L                    ------------------------------------------------------------
Calanus finmarchicus SIT-L                    ------------------------------------------------------------
Platynereis dumerilii SIT-L                   ------------------------------------------------------------
Capitella teleta SIT-L                        ------------------------------------------------------------
Ciona intestinalis SIT-L                      ------------------------------------------------------------
Molgula tectiformis SIT-L                     ------------------------------------------------------------
Phallusia mammillata SIT-L                    ------------------------------------------------------------
Scyphosphaera apsteinii SIT-L                 ------------------------------------------------------------
Coccolithus pelagicus SIT-L                   ------------------------------------------------------------
Calcidiscus leptoporus SIT-L                  ------------------------------------------------------------
Rosalina sp. SIT-L                            LRGSKSVDDNKY---------NGYSNNNSSMGLAGN------TNTMASNATDRTSMQKIS
Elphidium margaritaceum SIT-L                 ------------------------------------------------------------
Ammonia sp. SIT-L1                            EGTGVDGSGAVA------------------------------------------------
Ammonia sp. SIT-L2                            YGELNGTAGDQL---------GTFPLAPPQQPRPRR------------------------
Savillea parva SITBeta                        EGAQVSVVGLQS-IPMESFR-YTHPRAYKCCALAHK------GPNVERFIVGRQFLLLFI
Acanthoeca spectabilis SITBeta                EGAQVSIVGLQS-VPIETFR-LTHPQSYRCCKLVHA------GPNVERFIVGRQFLLLFI
Helgoeca nana SITBeta                         EGAQVSIVGLQS-VPIESFR-NTHPRAFKCCELVHK------GPNVERFIVGRQFLLLFI
Stephanoeca diplocostata SITAlphaBC           EGCQISIVGLQA-IDIELYK-QTHPRAYQVLKLAHK------GPNVERFLVGRQFLLLFN
Stephanoeca diplocostata SITAlphaA            EGCQISIVGLQA-INIELYK-KTHPRAYQVLKLAHK------GPNVERFLVGRQFLLLFN
Acanthoeca spectabilis SITAlpha               EGAQISIVGLQG-IDFEQYR-HSHPRAYAALKVVHA------GPNVERFLVGRQFLLLFN
Helgoeca nana SITAlpha                        EGAQISLVGLQG-INLELYK-DSHPRAYAALKLCHH------GPNVERFLVGRQFLLLFN
Savillea parva SITAlpha                       EGCQISLVGLQG-IDLEKYK-HSHPRAYAALKVCHS------GPNVERFLVGRQFLLLFN
Diaphanoeca grandis SITAlpha                  EGCQISIVGLQN-VNVEAYK-LTHPRAYAVCNLAHK------GPNVERFLVGRQFLLLFN
Diplotheca costata SITAlpha                   EGCQISIVGLQN-VNVEAYK-DTYPRGYAVCKLAHR------GPNVERFLVGRQFLLLFN
Stephanoeca diplocostata SITBeta              EGAHVSIVGLSS-VDIETFK-DTHPQSYTVCKWAHK------GPNLERFIVGRQFLLLFI
Diaphanoeca grandis SITBeta                   EGAQVSIVGLSS-VNIESFK-MTHPRSYAVCKLCHR------GCNLERFIVGRQFLLLFI
Diplotheca costata SITBeta                    EGAQVSIVGLSA-VDIEKFR-YSHPQSYATCKLLHE------GANLEKFIVGRQFLLLFV
Prymnesium neolepis SIT                       EGVQAALVALTP-IDPKIYE-KSHPITYMCTTLCFK------GNTLDKFIIGRQFLVIFV
Scyphosphaera apsteinii SIT                   EGTQAALVALQH-IDPEVYR-TSHPHAYKCATLGFK------GNNLDKYIIGRQFSVVFV
Phaeodactylum tricornutum SIT3                EGGQGALVGLQP-IDKELYA-VSHPRTYACTKLAHQ------GNNMERVIVGRQFLVVLV
Synedra acus SIT                              EGGQGALVGLQP-VDKALYA-ESHPRALLRAPLAHN------GDNMERFIVGRQYLVVLV
Phaeodactylum tricornutum SIT2                EGGQGSLVGLAP-VNGELYK-DSHPIAYKCTSIAHK------GDNLDRYLLGRQFMVVLT
Phaeodactylum tricornutum SIT1                EGGQGSLVGLAP-VNRELYK-DSHPIAYKCTAIAHK------GDNLDRYLLGRQFMVVLT
Cylindrotheca fusiformis SIT3                 EGGQCSMVGLPP-IDRELYK-ESHPITYKICSLGHK------GNNLDRYLMGRQFLVIFI
Cylindrotheca fusiformis SIT1                 EGGQCSMVGLPP-IDRDLYK-ESHPITYKICSLGHK------GNNLDRYLMGRQFMVIFI
Cylindrotheca fusiformis SIT5                 EGGQCSMVGLPP-IDRELYK-ESHPTTYKICSLGHK------GNNLDRYLMGRQFMVIFI
Cylindrotheca fusiformis SIT4                 EGGQCSMVGLPP-IDRDLYK-ESHPTTYKICSLGHK------GNNLDRYLMGRQFMVIFI
Cylindrotheca fusiformis SIT2                 EGGQCSMVGLPP-IDRDLYK-ESHPTTYKICSLGHK------GNNLDRYLMGRQFMVIFI
Nitzschia alba SIT                            EGGQCSMVGLPP-VNRDLYK-ESHPITHQICSLGHK------GDNLDRYLMGRQFMVIFI
Thalassiosira pseudonana SIT2                 EGQQASLVGLPP-VDPTLYK-DSHPLTYKNAALAFK------GDNLDRYLMGRQFMVLLV
Thalassiosira pseudonana SIT1                 EGQQASLVGLPP-VDPDLYK-DSHPLTYKNAALAFK------GDNLDRYLMGRQFMVLLV
Skeletonema costatum SIT                      EGQQASLVGLPP-VDPELYK-ETHPVTYLNAATAFL------GDNLDRYLMGRQFMVLLV
Thalassiosira pseudonana SIT3                 EGQQGALVGLTGVVDHLVYK-ESHSLAFRNTQLAYR------GDNLDRYVTGRQFMVLMC
Dinobryon sp. SIT                             EALHYAVVAVEK-WDMQQFA-EKFPRAVKCHSFVDT------PQKVKKFLVGRQFFVIFV
Mallomonas sp. SIT*                           ------------------------------------------------------------
Ochromonas distigma SIT                       EGLQVAILAVEK-YSPDNMQ-DTHPRAYKLIKSVRE------GNNVERFLVGRQFFTIFV
Ochromonas sp. SIT                            EGLQVAILAVEK-YXPDKMQ-ETHPRAYKLIKSVRE------GNNVERFLIGRQFFTIFV
Paraphysomonas bandaiensis SIT1               EGLQVAILAVEH-TDPEKYR-ETHPRAYELITSVKR------GNNVEKFLVGRQFFTVFV
Paraphysomonas imperforata SIT1               EGLQVAILNCEH-VDPEKRK-ESHPRAYKLMTSVRQ------GNNVERFLVGRQFFTIFV
Paraphysomonas imperforata SIT2               EGFQVGVLNSQS-MSAIFIRDEGYERAARVHEVMFGVPGNWVTGNLKRLLVGQSFMVVFL
Paraphysomonas bandaiensis SIT2               EAFQVGVLNTQH-LDAQYIRDEGYPRAAKVHELMFGTKAAPVESNVKRLLIGQSFMVVLC
Noctiluca scintillans SIT-L                   ------------------------------------------------------------
```

TM3

TM3 TM4

```
Rhodococcus opacus B4 SIT-L            ------------------------------------------------------------
Rhodococcus opacus SIT-L               ------------------------------------------------------------
Synechococcus KORDI                    ------------------------------------------------------------
Synechococcus CC9616 SIT-L             ------------------------------------------------------------
TARA_004 SRF 0.22-1.6                  ------------------------------------------------------------
TARA_142 SRF 0.22-3                    ------------------------------------------------------------
Florenciella parvula SIT-L             ------------------------------------------------------------
Phaeodaria OSH121 SIT-L1               ------------------------------------------------------------
Phaeodaria OSH121 SIT-L2               ------------------------------------------------------------
Ceratium fusus SIT-L                   ------------------------------------------------------------
Dinophysis acuminata SIT-L             ------------------------------------------------------------
Calanus finmarchicus SIT-L             ------------------------------------------------------------
Platynereis dumerilii SIT-L            ------------------------------------------------------------
Capitella teleta SIT-L                 ------------------------------------------------------------
Ciona intestinalis SIT-L               ------------------------------------------------------------
Molgula tectiformis SIT-L              ------------------------------------------------------------
Phallusia mammillata SIT-L             ------------------------------------------------------------
Scyphosphaera apsteinii SIT-L          ------------------------------------------------------------
Coccolithus pelagicus SIT-L            ------------------------------------------------------------
Calcidiscus leptoporus SIT-L           ------------------------------------------------------------
Rosalina sp. SIT-L                     ------------------------------------------------------------
Elphidium margaritaceum SIT-L          ------------------------------------------------------------
Ammonia sp. SIT-L1                     ------------------------------------------------------------
Ammonia sp. SIT-L2                     ------------------------------------------------------------
Savillea parva SITBeta                 VFMISRIGGAGDMPH----------------KNFYIGDWEWSSEATQIFWTNSVLLIVLV
Acanthoeca spectabilis SITBeta         VFIISRIGGAGVLPE----------------ENFYIGDWRWNSEATQFFWVNSVLLLIVI
Helgoeca nana SITBeta                  VFIVSRIGGAGTLPT----------------ENFYIGDWQWSSEATQLFWVNSVFLIIII
Stephanoeca diplocostata SITAlphaBC    GFLVTKVSGADGD-------------------EFYIGDWHWTREAANFFWKNSVLLMIVI
Stephanoeca diplocostata SITAlphaA     GFLVTKVSGADGD-------------------EFYIGDWHWTREAANFFWKNSVLLMIVI
Acanthoeca spectabilis SITAlpha        GFLMSKVSGAADQG-----------------HNFYIGDWEWSAEATQFFWLNSVLLMVVI
Helgoeca nana SITAlpha                 GFLLSKVAGAATE-------------------DFYIGDWKWSSEASQFFWVNSVLLMIVV
Savillea parva SITAlpha                GFLLSKVAGAGTS-------------------EFYIGDWHWSSEANDFFWSNSALLMVLI
Diaphanoeca grandis SITAlpha           GFLVSRIGGGKQE-------------------DFYIGDWHWNVEATQFFWLNATLLMVVI
Diplotheca costata SITAlpha            GFLVSRIGGGKHE-------------------DFYIGDWDWNDSATDFFWLNSTLLMIVI
Stephanoeca diplocostata SITBeta       VFMVSRLGGSDGSDL-----------ENTGGRYFFIGEWEWSATADVIFIQNNVLLMIVI
Diaphanoeca grandis SITBeta            VFIISRLGSHNQLKESFKGFNESATVNNTYDKDFYIGDWEWVRIAELVFLQNSILLMIVI
Diplotheca costata SITBeta             VFIISRLGGHAPGRVD----------EKTNNGDFYIGDWHWHTNADLAFMQNNILLMIVI
Prymnesium neolepis SIT                VFMIGFCTGYDGD-------------------RLVDNPLGVPDGFRDSLIKLGFVGALTT
Scyphosphaera apsteinii SIT            VFMIGYCTSYDAD-------------------KMENNPFDLSDGFRDSMIKLGFVSALTT
Phaeodactylum tricornutum SIT3         VFVTNLMVSSIA----------------------NASVLSLPDAINETFLATGLAVTLTV
Synedra acus SIT                       ITVINLMGSAVK----------------------GATVLGLPQVMNDIFLGNGLAMILTT
Phaeodactylum tricornutum SIT2         VFTINISGGPLK----------------------DAELWGFPSVLTNMFLGSGLAMILFT
Phaeodactylum tricornutum SIT1         VFVVNMSGGPLK----------------------DAELWGFPYVLTNMFLGSGLAMILFT
Cylindrotheca fusiformis SIT3          NFTINLCGAPLD----------------------GAEVLGLPKVLTDIFLGSGIAMVLIV
Cylindrotheca fusiformis SIT1          NFTINLCGAPLE----------------------GAEVLGLPEILTDIFLGSGIAMVLTV
Cylindrotheca fusiformis SIT5          NFTINLCGAPLE----------------------GAEVLGLPEILTDIFLGSGIAMVLTV
Cylindrotheca fusiformis SIT4          NFTINLCGAPLE----------------------GAEVLGLPEILTDIFLGSGIAMVLTV
Cylindrotheca fusiformis SIT2          NFTINLCGAPLE----------------------GAEVLGLPEILTDIFLGSGIAMVLTV
Nitzschia alba SIT                     NFTINLCGAPLE----------------------DAEVLGLPSVIQSIFLGSGIAMILTV
Thalassiosira pseudonana SIT2          VFVINQCSSPLDP---------------------KVDVLGLPDGVKFIFLDIGLAMIIFT
Thalassiosira pseudonana SIT1          VFVINQCSSPLDP---------------------TVDVLGLPDGVKFIFLDIGLAMIIFT
Skeletonema costatum SIT               VFIINLCGAPSSG---------------------DADVLGMPGWLKTIFLDVGLGMIIFT
Thalassiosira pseudonana SIT3          VFVINLCGSPLPG--------------------MSESSLNLPQIIEEIFLKTGIAMILMT
Dinobryon sp. SIT                      VFLLAQITSFPDI---------------------PANFAGMPHVLVLILVQTGLPGVALT
Mallomonas sp. SIT*                    ------------------------------------------------------------
Ochromonas distigma SIT                MTLIAQVTSFPE-----------------------ISNLGINPIVWFIFVQTGLPAALVV
Ochromonas sp. SIT                     MTLIAQVTSFPN-----------------------ISSLGIPPVIWFIFVQTGLPAALVV
Paraphysomonas bandaiensis SIT1        MTLMAQVTSFPN-----------------------ISSLGIPPVIWFIFIQTGLPGAMVV
Paraphysomonas imperforata SIT1        MTLMAQVTSFPD-----------------------ISHLGVPDVVWFIFISTGLPGAMVV
Paraphysomonas imperforata SIT2        SFTLAQLTTFPD----------------------YPNIFNMNSHLFNMLFCSGLPGVVIT
Paraphysomonas bandaiensis SIT2        SFTLAQLTTFKY----------------------YPLFVPMPEALFSGLVRSGLPGVVLV
Noctiluca scintillans SIT-L             ------------------------------------------------------------
```

TM3 TM4

```
                                              TM4                  TM5
Rhodococcus opacus B4 SIT-L                  --------------------------AMRRVVEAVEELDLGT------------------
Rhodococcus opacus SIT-L                     --------------------------AMRRVVEAVEELDLGT------------------
Synechococcus KORDI                          ------------------------------------------------------------
Synechococcus CC9616 SIT-L                   ------------------------------------------------------------
TARA_004 SRF 0.22-1.6                        ------------------------------------------------------------
TARA_142 SRF 0.22-3                          ------------------------------------------------------------
Florenciella parvula SIT-L                   ---------------------------------------LGD------------------
Phaeodaria OSH121 SIT-L1                     ------------------------------------------------------------
Phaeodaria OSH121 SIT-L2                     ------------------------------------------------------------
Ceratium fusus SIT-L                         ------------------------------------------------------------
Dinophysis acuminata SIT-L                   -------------------------------------------QH---------------
Calanus finmarchicus SIT-L                   ------------------------------------------------------------
Platynereis dumerilii SIT-L                  ------------------------------------------------------------
Capitella teleta SIT-L                       ------------------------------------------------------------
Ciona intestinalis SIT-L                     ------------------------------------------------------------
Molgula tectiformis SIT-L                    ------------------------------------------------------------
Phallusia mammillata SIT-L                   ------------------------------------------------------------
Scyphosphaera apsteinii SIT-L                ------------------------------------------------------------
Coccolithus pelagicus SIT-L                  ------------------------------------------------------------
Calcidiscus leptoporus SIT-L                 ------------------------------------------------------------
Rosalina sp. SIT-L                           ------LSSPNAAELLRRIEEIDE--NQAVHKKSIDLATHGE--------------D---
Elphidium margaritaceum SIT-L                ------------------------------------------------------------
Ammonia sp. SIT-L1                           ------------------------------------------------------------
Ammonia sp. SIT-L2                           ----------------------------DTVDTIETHGDIAI------------------
Savillea parva SITBeta                       IVPGNLIAQLMASSNMLAFLDLPYGAYYTVVLPSLAIESLGI-THTSYILKDLFVY----
Acanthoeca spectabilis SITBeta               IVP-ILVAQLLAADKMLAFLELSFAPYYTVVLPSLALEAVGI-THAAYVLKELFVW----
Helgoeca nana SITBeta                        TVPGSLVTQLMAANKMLAFLELPYAPYYTVVLPSLALESIGI-THTSYVLKDVFVR----
Stephanoeca diplocostata SITAlphaBC          IVPGQLVSQLMAAEKMLGFLNLPFYGYYTVLLPCLIMESTGL-VHSSYMLKDVLCK----
Stephanoeca diplocostata SITAlphaA           IVPGQLVSQLMAAEKMLGFLNLPFFGYYTVLLPCLIMESTGL-VHSSYMLKDVLCR----
Acanthoeca spectabilis SITAlpha              ISLGQLPTQLLAADKMLGFFDLHYYALYTIVYPCLFVESIGL-THSSYLLKDLCCI----
Helgoeca nana SITAlpha                       AAFAQLVTQLLAAQKMLGFLNLPFFALYTIVYPCLIVESVGL-THSSYLLKDFLVH----
Savillea parva SITAlpha                      AAFAQLPTQLLAAQKMLGFLNLPFFSLYTIVYPCLIVESVGL-THSTYLLKDFLVR----
Diaphanoeca grandis SITAlpha                 VVPGQLVTQLIAMDKMLGFLNLKFYAYYTVLMPCMFVESIGL-THSAYLLKDVLVK----
Diplotheca costata SITAlpha                  IALGQLPTQLMAADKMLGFLDLRFYGYYTVLLPCLFVESIGL-THSTYLLKDILVS----
Stephanoeca diplocostata SITBeta             IVPGHLVSQLIAAGKMLDFLELPFAPIHTVMYPSMAFETLGL-THCCYALRDLFLR----
Diaphanoeca grandis SITBeta                  IVPG-LVSQLIAADKMLDFLELPFAPMLTVALPSLGLEALGI-THTAYVLRDLFAR----
Diplotheca costata SITBeta                   IVPGQLVSQLLAAGKMLAFLELPFFPMYTVAYPSLLLEFLGI-THCAYVIKDITAK----
Prymnesium neolepis SIT                      IVIGQLISQVVASKCNIDYLNYRF-QYYAVIGVCLFMEFTGL-MHAVYMVQKFVVA----
Scyphosphaera apsteinii SIT                  IVVGQLISQIVASRCNIDYLNYRF-QYYAVISVCLFMEFIGL-MHFVYLIQKVVVQ----
Phaeodactylum tricornutum SIT3               IIVGQLTAQVNAANCMLDFINNYF--MLFTIYTSLVIEASGL-LHSVYLVQTIFSK----
Synedra acus SIT                             IMVGQLTAQVNAANCMLDFINNYF--MLFTSYVSYGIEFSGL-LHCVYLVQMIFSK----
Phaeodactylum tricornutum SIT2               AMIGQLNSQVNASLCMLDYINNYF--ALFTFWVAMAIEFSGL-LHASYLVQMLVAA----
Phaeodactylum tricornutum SIT1               AMVGQLNSQVNASLCMLDYINNYF--ALFTLWVAMAIEFSGL-LHASYLVQMLVAA----
Cylindrotheca fusiformis SIT3                VPLGQLTAQVNSSHCMLDYINTHF--MTFTLYITLIIEATGV-MHVCYLIRDMFYH----
Cylindrotheca fusiformis SIT1                VTIGQLTAQVNASHCMLDYINTHF--MTFTLYVTLVIEATGV-MHSCYLIRDMFYH----
Cylindrotheca fusiformis SIT5                TTIGQLTAQVNASHCMLDYINTHF--MTFTLYVTLIIEATGV-MHSCYFIRDLFYH----
Cylindrotheca fusiformis SIT4                VTIGQLTAQVNASHCMLDYINTHF--MTFTLYVTLIIEVTGV-MHSCYLIRDMFYF----
Cylindrotheca fusiformis SIT2                VTIGQLTAQVNASHCMLDYINTHF--MTFTLYVTLIIEVTGV-MHSCYLIRDMFYF----
Nitzschia alba SIT                           VTIGQLTAQVNASHCMLDYVNTHF--MTFTLYVALAIEKTGV-KHTSYLIQYFFYW----
Thalassiosira pseudonana SIT2                CILGQLTTQVNASYAMIDFINNYF--ALFTLYTTMVVEFSGI-MHASYLIQNILAA----
Thalassiosira pseudonana SIT1                CILGQLTTQVNASYAMIDFINNYF--ALFTLYTTMAVEFSGI-MHSSYLIQNILSA----
Skeletonema costatum SIT                     CQLGQLTTQVNASHCMLDFINNYF--ALFTLYTAMCIEFSGV-MHSSYLIQNVLSF----
Thalassiosira pseudonana SIT3                AMISQLPPQVNASHCMIDFINNYF--ALFTLYTALIIEFSGV-MHASYLIQNIISL----
Dinobryon sp. SIT                            LTFGQLVSQIYVEEFTLQFLNLYG--CEFVIRLGLGAEWIGI-CNFSWLLYGTASRVFCG
Mallomonas sp. SIT*                          MTFGQLIPQLFVEQYTLPFMNLYG--NVFVMDLCNTAEFLGV-CHFSWLLYFTVSGVVCR
Ochromonas distigma SIT                      TTIGSLQPQLLAARDPWKFLDLYG--CNAVLNLCYGMELTGICTHFAWMLVIILRHTLF-
Ochromonas sp. SIT                           TTIGSLQPQLLAAKDPWXFLNLWG--CNAVLNLCYGLELSGIATHFAWMLIGILRKTLF-
Paraphysomonas bandaiensis SIT1              TTIGSLQPQLLAAKDPWKFMNLYG--SCATLYLCYGLEFTGICTHFAWMLITILRNTVF-
Paraphysomonas imperforata SIT1              TTIGSLQPQLLAAKDPWNFLDLYG--SNSVLNLCYGLEWTGICTHFAWMLIGILRKTVF-
Paraphysomonas imperforata SIT2              VVIGQLTPSLLAKEYPIGFLNLPG--IYHIILVALYVEKSGI-SHFVYVLYGICNTLFFK
Paraphysomonas bandaiensis SIT2              ICFAQLTPSLMAKEYPLRILNIPG--IYSTIYLALLIEQCGL-VHFVYVFYGIVNRVCFA
Noctiluca scintillans SIT-L                  ------------------------------------------------------------
                                              TM4                  TM5
```

```
Rhodococcus opacus B4 SIT-L         ------------------------------------------------------------
Rhodococcus opacus SIT-L            ------------------------------------------------------------
Synechococcus KORDI                 ------------------------------------------------------------
Synechococcus CC9616 SIT-L          ------------------------------------------------------------
TARA_004 SRF 0.22-1.6               ------------------------------------------------------------
TARA_142 SRF 0.22-3                 ------------------------------------------------------------
Florenciella parvula SIT-L          ------------------------------------------------------------
Phaeodaria OSH121 SIT-L1            ------------------------------------------------------------
Phaeodaria OSH121 SIT-L2            ------------------------------------------------------------
Ceratium fusus SIT-L                ------------------------------------------------------------
Dinophysis acuminata SIT-L          ------------------------------------------------------------
Calanus finmarchicus SIT-L          ------------------------------------------------------------
Platynereis dumerilii SIT-L         ------------------------------------------------------------
Capitella teleta SIT-L              ------------------------------------------------------------
Ciona intestinalis SIT-L            ------------------------------------------------------------
Molgula tectiformis SIT-L           ------------------------------------------------------------
Phallusia mammillata SIT-L          ------------------------------------------------------------
Scyphosphaera apsteinii SIT-L       ------------------------------------------------------------
Coccolithus pelagicus SIT-L         ------------------------------------------------------------
Calcidiscus leptoporus SIT-L        ------------------------------------------------------------
Rosalina sp. SIT-L                  ------------------------------------------------------------
Elphidium margaritaceum SIT-L       ------------------------------------------------------------
Ammonia sp. SIT-L1                  ------------------------------------------------------------
Ammonia sp. SIT-L2                  ------------------------------------------------------------
Savillea parva SITBeta              ------------------------------------------------------------
Acanthoeca spectabilis SITBeta      ------------------------------------------------------------
Helgoeca nana SITBeta               ------------------------------------------------------------
Stephanoeca diplocostata SITAlphaBC ------------------------------------------------------------
Stephanoeca diplocostata SITAlphaA  ------------------------------------------------------------
Acanthoeca spectabilis SITAlpha     ------------------------------------------------------------
Helgoeca nana SITAlpha              ------------------------------------------------------------
Savillea parva SITAlpha             ------------------------------------------------------------
Diaphanoeca grandis SITAlpha        ------------------------------------------------------------
Diplotheca costata SITAlpha         ------------------------------------------------------------
Stephanoeca diplocostata SITBeta    ------------------------------------------------------------
Diaphanoeca grandis SITBeta         ------------------------------------------------------------
Diplotheca costata SITBeta          ------------------------------------------------------------
Prymnesium neolepis SIT             ------------------------------------------------------------
Scyphosphaera apsteinii SIT         ------------------------------------------------------------
Phaeodactylum tricornutum SIT3      ------------------------------------------------------------
Synedra acus SIT                    ------------------------------------------------------------
Phaeodactylum tricornutum SIT2      ------------------------------------------------------------
Phaeodactylum tricornutum SIT1      ------------------------------------------------------------
Cylindrotheca fusiformis SIT3       ------------------------------------------------------------
Cylindrotheca fusiformis SIT1       ------------------------------------------------------------
Cylindrotheca fusiformis SIT5       ------------------------------------------------------------
Cylindrotheca fusiformis SIT4       ------------------------------------------------------------
Cylindrotheca fusiformis SIT2       ------------------------------------------------------------
Nitzschia alba SIT                  ------------------------------------------------------------
Thalassiosira pseudonana SIT2       ------------------------------------------------------------
Thalassiosira pseudonana SIT1       ------------------------------------------------------------
Skeletonema costatum SIT            ------------------------------------------------------------
Thalassiosira pseudonana SIT3       ------------------------------------------------------------
Dinobryon sp. SIT                   KVRRI-------------------------------------------------------
Mallomonas sp. SIT*                 SLRQQ-------------------------------------------------------
Ochromonas distigma SIT             ------------------------------------------------------------
Ochromonas sp. SIT                  ------------------------------------------------------------
Paraphysomonas bandaiensis SIT1     ------------------------------------------------------------
Paraphysomonas imperforata SIT1     ------------------------------------------------------------
Paraphysomonas imperforata SIT2     EKEKDEEGEGMEEGGAIAKTKSTDTFVVPSLSDMEAYRGMNVGITMSANGSVDLNSVSDG
Paraphysomonas bandaiensis SIT2     GTSRNPT-----------------------------------------------------
Noctiluca scintillans SIT-L         ------------------------------------------------------------
```

```
Rhodococcus opacus B4 SIT-L          ---------FESIDLISPVTRLV-------------------------------------
Rhodococcus opacus SIT-L             ---------FESIDLISPVTRLV-------------------------------------
Synechococcus KORDI                  ------------------------------------------------------------
Synechococcus CC9616 SIT-L           ---------------------MK-------------------------------------
TARA_004 SRF 0.22-1.6                ------------------------------------------------------------
TARA_142 SRF 0.22-3                  --------------LLLENLNVN-------------------------------------
Florenciella parvula SIT-L           ---------GTAAEATSEYPPVI-------------------------------------
Phaeodaria OSH121 SIT-L1             ----------------KMEASLF-------------------------------------
Phaeodaria OSH121 SIT-L2             ------------------------------------------------------------
Ceratium fusus SIT-L                 --------------------VAL-------------------------------------
Dinophysis acuminata SIT-L           ---------MAAMSTASIVKAVR-------------------------------------
Calanus finmarchicus SIT-L           ----------LSRMVNVKEATIP-------------------------------------
Platynereis dumerilii SIT-L          ---------PFSKMVELKKANPF-------------------------------------
Capitella teleta SIT-L               ---------EIYKMVELRKANPC-------------------------------------
Ciona intestinalis SIT-L             -------------MVNFSESSPF-------------------------------------
Molgula tectiformis SIT-L            ---------KGTIMVKLNNLSWL-------------------------------------
Phallusia mammillata SIT-L           ------------------------------------------------------------
Scyphosphaera apsteinii SIT-L        ------------------------------------------------------------
Coccolithus pelagicus SIT-L          ---------LQQPSMWRWGAAFA-------------------------------------
Calcidiscus leptoporus SIT-L         --------------------AVA-------------------------------------
Rosalina sp. SIT-L                   ---------TITLCHGTTFQNVV-------------------------------------
Elphidium margaritaceum SIT-L        ------------------------------------------------------------
Ammonia sp. SIT-L1                   ---------IKMIHTDSTWTIIA-------------------------------------
Ammonia sp. SIT-L2                   ---------ELCLDANSLGAKIW-------------------------------------
Savillea parva SITBeta               --LSGTDTLEEEDPARRMKKDAA-------------------------------------
Acanthoeca spectabilis SITBeta       --LSGVEV-DENDP-KRMHKNKW-------------------------------------
Helgoeca nana SITBeta                --LSGAD--RKRDDPKQMKKNKL-------------------------------------
Stephanoeca diplocostata SITAlphaBC  --IGGIDV-SKGDPKKRMSKDFL-------------------------------------
Stephanoeca diplocostata SITAlphaA   --IGGIDV-SKGGPKKRMSKDFL-------------------------------------
Acanthoeca spectabilis SITAlpha      --IGRIDR-STEDPAKKMNKNFL-------------------------------------
Helgoeca nana SITAlpha               --ITGIPL-SEADPAKAQNKNIV-------------------------------------
Savillea parva SITAlpha              --ITGIDR-SQADPAKQMNKNFL-------------------------------------
Diaphanoeca grandis SITAlpha         --VTGVDP-QKGDPSKKMDKNFL-------------------------------------
Diplotheca costata SITAlpha          --VAGIDR-SQADPEKEIPRDFL-------------------------------------
Stephanoeca diplocostata SITBeta     --WAKL---PPISGAKAHRKNWL-------------------------------------
Diaphanoeca grandis SITBeta          --ASG----TEIDAKAKMPKTIF-------------------------------------
Diplotheca costata SITBeta           --LAGV---QSQDPDKEHNKNWF-------------------------------------
Prymnesium neolepis SIT              --LRGTDE-AKRKQVENQGNPIF-------------------------------------
Scyphosphaera apsteinii SIT          --IKGTDE-AKKKQMKNQGNPFF-------------------------------------
Phaeodactylum tricornutum SIT3       --MSGTPI-ESNEPSRSVFQSLL-------------------------------------
Synedra acus SIT                     --ITGKPI-ESNEPPRNAAQNLF-------------------------------------
Phaeodactylum tricornutum SIT2       --LSGKKI-ESNEEPRNGLQNLF-------------------------------------
Phaeodactylum tricornutum SIT1       --LSGKKI-ESNEEPRNGLQNLF-------------------------------------
Cylindrotheca fusiformis SIT3        --AAGKPV-ESNEPPRSAVQNVF-------------------------------------
Cylindrotheca fusiformis SIT1        --AAGKPV-ETNEPPRSAVQNLF-------------------------------------
Cylindrotheca fusiformis SIT5        --AAGKPV-ETNEPPRTAVQNLF-------------------------------------
Cylindrotheca fusiformis SIT4        --AAGKPV-ETNEPPRNAVQNLF-------------------------------------
Cylindrotheca fusiformis SIT2        --AAGKPV-ETNEPPRNAVQNLF-------------------------------------
Nitzschia alba SIT                   --LAGKPV-VTNEPPRSAPQAIF-------------------------------------
Thalassiosira pseudonana SIT2        --VSGKPI-STNEEPKTGMTFAF-------------------------------------
Thalassiosira pseudonana SIT1        --VSGKPI-QTNEEPKTGMTFAF-------------------------------------
Skeletonema costatum SIT             --ASGKPI-HSNEEPKRGFTLLF-------------------------------------
Thalassiosira pseudonana SIT3        --ISGKPV-KTREEPRTRLQASF-------------------------------------
Dinobryon sp. SIT                    -QKSLDSA-KSSDDMMEYQQRQVENGTNE------PMSPTELNRGPNYVSGVEP------
Mallomonas sp. SIT*                  -QLKGEST-EEIGIIVEEQVEKV-------------------------------------
Ochromonas distigma SIT              --ITEEKP-RTHNPEMSPAYYAL-------------------------------------
Ochromonas sp. SIT                   --ITDEKP-RKHDVEKTPAYYAL-------------------------------------
Paraphysomonas bandaiensis SIT1      --ITDQKP-KKHQREVTLQSQLI-------------------------------------
Paraphysomonas imperforata SIT1      --RSDIPT-KDKKSEMNTQERAI-------------------------------------
Paraphysomonas imperforata SIT2      KSESTGNS-EGVSSVTLTSASSVVKVPSDSKLGWHNDEPDSAPNTPKKSEFVTAMPSLNR
Paraphysomonas bandaiensis SIT2      -PLSGKEL-SDTESVSHKSTEEVCEYSEE------EDTEDSACEVAIEGAVQTA------
Noctiluca scintillans SIT-L          ---------GRLWNFMSFFTLWL-------------------------------------
```

TM6 (1) **TM7 (2)**

```
Rhodococcus opacus B4 SIT-L              ------------RHVRDVAAGALFAFCLYLVLGAVLTGQNAMA-----NHTSTAFVLGAL
Rhodococcus opacus SIT-L                 ------------RHVRDVAAGALFAFCLYLVLGAVLTGQNAMA-----NHTSTAFVLGAL
Synechococcus KORDI                      ----------------------------------------------------MWHTFLLL
Synechococcus CC9616 SIT-L               ------------NFFSFAFSLFLLVATVYIVLFGILSGESSLT---LVFGSPIWLTFLLL
TARA_004 SRF 0.22-1.6                    ------------------------------------------------------------
TARA_142 SRF 0.22-3                      ------------KLFSIVFSLVLLLAAFYVVLFGILAGQSSLT---LMFGSPVWLTVLLL
Florenciella parvula SIT-L               ------------DIVRHVFSAIITLGAIGLIFYGIGAGYAALP-------GHPTLLYIIF
Phaeodaria OSH121 SIT-L1                 ------------DYARYIFATILTIISLFLIGYAIAGGHAALP-------GHPIMQYALL
Phaeodaria OSH121 SIT-L2                 -------------LPSLCFCDNFNDSINWVNRLCDCRRPCSSS-------WSSCYAISLL
Ceratium fusus SIT-L                     ------------DVFKKIVSSVLTLGGLALICYGIFTGQAVLT-------SNTVVPFILF
Dinophysis acuminata SIT-L               ------------EGVKKTVSTILTLGGFAFICYGIATGHAALT-----------------
Calanus finmarchicus SIT-L               ------------DYLRFVFSFCLLIFSGVVTCYAIWAQKTSMW-----KAVPGWASLIIF
Platynereis dumerilii SIT-L              ------------DYIRFIISALLLLFSLIVTSYAIWQKNTMFW-----DAVPGYAALILF
Capitella teleta SIT-L                   ------------DYIRFIISSLLLLFAIVCTSYAIIMQHTHFW-----KEVPGWAGLIIL
Ciona intestinalis SIT-L                 ------------DYIRYVISFTLLSFSIAVTSYAIFEGRTGFW-----PQVPGPAAFALF
Molgula tectiformis SIT-L                ------------DYPRLALSFTLLSFSALITFYAIFTGITTFY-----HAVPPVASFCIF
Phallusia mammillata SIT-L               -----------------------------------------------------------M
Scyphosphaera apsteinii SIT-L            ------------------------------------------------------------
Coccolithus pelagicus SIT-L              ------------HHGRVAFSFIFLVFCCGVTGTAIFTGKTDFF------GVPAWAQLLLL
Calcidiscus leptoporus SIT-L             ------------YHGRVVFSSLLLIFCCGVVGSGIFMGKTDFF------GVPAWAQLLLL
Rosalina sp. SIT-L                       ------------DTIRYIFSSCLLCLSLFSIVYAITEHETGMP-----TTIPNWTLYVML
Elphidium margaritaceum SIT-L            -----------------------------------------MP-----DVFPPWAQFTIL
Ammonia sp. SIT-L1                       ------------DVIRLVFSICLLIFSTIMTLYAIATSTTTMP-----GTIPPWAQYLLL
Ammonia sp. SIT-L2                       ------------DTIRLIFSSVLLCLSIFSIAYAITEGGTEFP-----DGFPHWTLYLIL
Savillea parva SITBeta                   ------------YYIKAVVSACLVVFSAVFVFKGLFAGQTGATNGVGWDKLPGWAAFLLT
Acanthoeca spectabilis SITBeta           ------------YYVRCFFSVSLVIFSGVFVFKGLFAGQTGATNGVGWDKLPGWAAFLLT
Helgoeca nana SITBeta                    ------------YYARCIMSVGLVIFSTIFVLKGLFSGQTGATNGVGWNNLPGWAAFIIT
Stephanoeca diplocostata SITAlphaBC      ------------YYSRVLISIAAVIFSGVFIIKGLSKKQTNATDGPGWNKLPGWAAIIMT
Stephanoeca diplocostata SITAlphaA       ------------YYSRVLISISAVIFSGLFIIKGLANKQTNATDGPGWNKLPGWAAIIMT
Acanthoeca spectabilis SITAlpha          ------------YYLRCFISVCAVIFSFTFIVKGLALRQTAATDGVGWYKLPGWAAVILT
Helgoeca nana SITAlpha                   ------------YYIKCGISVAAVIFSFVFIVKGLAMSQTNATEGVGWNKLPGWAAVLLS
Savillea parva SITAlpha                  ------------YYARCALSVAAVIFSFVFIVKGLAMKQTNATEGVGWSKLPGWAAVLCS
Diaphanoeca grandis SITAlpha             ------------YYFRVLISVSAVIFSGTFIVKGLYMSQTNATQGPGWRKLPGYGAVIVG
Diplotheca costata SITAlpha              ------------HNIRVFISVSAVIFSGVFIVKGWALGQTGATTGPGWENLPGGAAVAVG
Stephanoeca diplocostata SITBeta         ------------YHARVLMSVCVVIFASVFIVKGLFAEQTGATNGVGWNKLPGVGAFFVC
Diaphanoeca grandis SITBeta              ------------YYLKVFMSITLVVFGLVCVLVGLFTKQTNATNGKGWELLPGWAAFLVT
Diplotheca costata SITBeta               ------------YYLRCTYSVLLVMFSAVVIVKGLLAGQTNV-----WDTLPGYGALLLS
Prymnesium neolepis SIT                  ------------FWVRVLFSTAVLGLCVAVVFDSLFRGATGFA--DVFSSLPNWLGVIFF
Scyphosphaera apsteinii SIT              ------------FWGRVVLSTGVLALCLAIVFDSLFRGDTGYA--ATFPDMPNYSGLIFF
Phaeodactylum tricornutum SIT3           ------------FWGRVAMSLVLLGFSFAVLLTALFNGKTNM-----WDGIPAIASVIIF
Synedra acus SIT                         ------------FWARVIMSVAILCFCLAVTLVALFDGKTM------WDGVPPYVSVIIL
Phaeodactylum tricornutum SIT2           ------------FWSRCLVSLAILAYCFAVTLAALFDGKTTM-----WEGVPSAVAVIVF
Phaeodactylum tricornutum SIT1           ------------FWSRCLGSLAILGYCFAVTLAALFAGKTTM-----WEGVPPSVAVIVF
Cylindrotheca fusiformis SIT3            ------------HWGRVAFSLGVLCFALAVTIEALFNGETTM-----WQSIPNGVAIVLF
Cylindrotheca fusiformis SIT1            ------------HWGRVVFSLGVLCFALAVTIEALFNGKTTM-----WEFIPNGVAIVLF
Cylindrotheca fusiformis SIT5            ------------HWGRVVFSLGVLCFALAVTLEALFNGKTTM-----WEFIPNGVAVLLF
Cylindrotheca fusiformis SIT4            ------------HWGRVVFSLGVLCFALAVTLEALFKGQTTM-----WEFIPNGVAVVLF
Cylindrotheca fusiformis SIT2            ------------HWGRVVFSLGVLCFALAVTLEALFKGQTTM-----WEFIPNGVAVVLF
Nitzschia alba SIT                       ------------FWGRCLFSVGVLFFALAVTLKALFDGNTTM-----WSFIPNTVAVILF
Thalassiosira pseudonana SIT2            ------------FWGRVLMSLAILGFCLAVTLSALLNGQTSVA--VKYPSISPGLAVFLL
Thalassiosira pseudonana SIT1            ------------FWGRVLMSLAILGFCLAVTLVALFNGQTSVS--VKYPSISPGLSVFLL
Skeletonema costatum SIT                 ------------FWGRVLMSLAILGFSLAVVISALFQGRTMMA--VKYPSVSNGASVFLF
Thalassiosira pseudonana SIT3            ------------FWLRVLMSLSTLCFSMAVTVVALFQGKTTM-----WQGVPEWVSLVLF
Dinobryon sp. SIT                        -----KEHLSWFDYLKYAWSTAATIGAMIVVCYGISLQVYVLP-------VPPVGAYILA
Mallomonas sp. SIT*                      -----EPYGAW-DLFRFTWSTFATFSSVFLILLGISRGHAILP-------APIGACYVVL
Ochromonas distigma SIT                  ------------EGLKFTVSFVVLMTYCSYIMWGIWTGQAILP-------VPFIVVFIIY
Ochromonas sp. SIT                       ------------EGFKYVLSAGVXLTYCSYIMWGIWTGQAILP-------VPFVVVFIIY
Paraphysomonas bandaiensis SIT1          ------------EAAKYVVSTCVILTYMTYLMWGIWTGQAVLP-------VPHIAVFIVF
Paraphysomonas imperforata SIT1          ------------DYLKYFVSFCVLGMYFTYLMYGIWTGEALLS----QSGVPGVVVFIVF
Paraphysomonas imperforata SIT2          KNEVLSCCNTTILYLKYVLSTIFTLVCFAFLIYGLAMRYSLIN-------LTLPLQLLLL
Paraphysomonas bandaiensis SIT2          -----RMIDTILLYVRYVLSTGLTVLCIVFVLGCIGSGHSMLE-------INVGVQFFLG
Noctiluca scintillans SIT-L              ------------SLVKYITSTMAYMLSIAFVVWCISEGQTDFS-------VPVAWNMCLM
```

TM6 (1) **TM7 (2)**

TM7 (2)

Rhodococcus opacus B4 SIT-L	AVCLGLLALLEAAHIAAVALSTADVSQ---LRESHSRVFKLHPFVA--TSERLEHYLAGR
Rhodococcus opacus SIT-L	AVCLGLLALLEAAHIAAVALSTADVSQ---LRESHSRVFKLHPFVA--TSERLEHYLAGR
Synechococcus KORDI	VLTLLLLSSLEGSQIAIISLSDRNTDQLIGVKNESPKACSRMRLIC--SKIRSQQYLAGR
Synechococcus CC9616 SIT-L	VFTLLLLSSLEGSQIAIVSLSDRNPDELTGVKSEYPKAYSVMRLIC--SKIRSQQYLAGR
TARA_004 SRF 0.22-1.6	------------------SLSDRDHDQLVEVRSDYPTAYSVMRLIC--SKLRSQQYLAGR
TARA_142 SRF 0.22-3	SFTLLLLASLEGSQIAIVSLSDRSVEQLSEVKGKYPSAFSTLQLLG--SKMRSQQYLAGR
Florenciella parvula SIT-L	FSVLILLAYLEGLQVAILALEHTNIDG---LRERYPRAHVTHALSTAYDGLNVQRFLVGR
Phaeodaria OSH121 SIT-L1	IFDLTLLAYLEGLQVAILALERVRYST---FPEHMKRAIASHKLATGGRGINVQRFLVGR
Phaeodaria OSH121 SIT-L2	VFDLTLLAYLEGLQVAILALERVRYST---FPEDMKRAIASHKLAIGNRGHNVQRFLVGR
Ceratium fusus SIT-L	LAAVTLLGYLEGLQVAILALEGQDGEP---FRKSHPRAYWLHTLVN--KPMNVQRFLVGR
Dinophysis acuminata SIT-L	---------PNPVVPAILAMEGQDAAP---FKESHPRAYSLHRWVN--QPLNVQRFLVGR
Calanus finmarchicus SIT-L	ILVLFLLGVMEGLQIALVELKRQEPES---YKNSHPKAYRLGQYAM--KADNVERFLMGR
Platynereis dumerilii SIT-L	FVDLFCLGIVEGLQIALVELKRQHPDS---YKNSHPRAHRLGQIAA--RGDNVERFLMGR
Capitella teleta SIT-L	VVDLFILGVVEGLQIALVELKRQHPEA---YKHSHPAAYRLGLIAL--KGDNVERFLMGR
Ciona intestinalis SIT-L	LVCLFLLGVVEGLQISLVELKRMHPGT---YKTSYPRAYKLSCIAG--KGDNVERFLMGR
Molgula tectiformis SIT-L	IVNLILLGIME----ALVELKRQHPDS---YKNSHPKAFELGKQAN--HKDNVERFLMGR
Phallusia mammillata SIT-L	ESGRRVILIIVGLQISLVELKRQHPDS---YKNTHKRSYQLAQIAN--SKDNVERFLMGR
Scyphosphaera apsteinii SIT-L	----TGLAVIEGLQIALVELKKHPTES---YRTSHARAFRTGSLAF--RGDNCERFLIGR
Coccolithus pelagicus SIT-L	LVSLFLLGVIEGLQIALVELKKQDPAS---YRASHPRAYTTGCLAS--CGENCERFLIGR
Calcidiscus leptoporus SIT-L	LVCLFLLGVVEGLQIALVELKKQDPEL---YRASHPRAYATGCFAS--RGENCERFLIGR
Rosalina sp. SIT-L	FLGLFCIGVLEGVQIAVVELAHEDPRE---YQKFYPRAAKMIKWEN--QGRNVERFLIGR
Elphidium margaritaceum SIT-L	IVALFILGVMEGVQIGVVELAHKDPNK---YRKQYPRAANLLALEN--KGRNVERFLLGR
Ammonia sp. SIT-L1	GIALMVLGVLEGLQIGVVELAHKDPNR---YRKLYPRAARLLAFEN--KGRNVERFLMGR
Ammonia sp. SIT-L2	LACLFCIGVLEGLQIGVVELAHEDPKQ---YERHYPRAAKLLKWEN--EGRNVERFLIGR
Savillea parva SITBeta	LFFLCVIGCCEGFQIAAVSLAKMPASA---YKTKAPLAYRTTQVLY--KGRNMQAFLVGR
Acanthoeca spectabilis SITBeta	LFFLFVIGCCEGFQIAAVSLAKLPSSD---FKTKAPIAYQTTQILF--AGRNMQAFLVGR
Helgoeca nana SITBeta	IFFLFVIGCCEGFQIAAVSLSQVPSDD---FKTKAPTAYKTTQLLY--AGRNMQAFLVGR
Stephanoeca diplocostata SITAlphaBC	LFFLFIMACAEGLQVSALALAKTHTAS---FKDKSPLAYRTTQLLY--AGRNMQAFLVGR
Stephanoeca diplocostata SITAlphaA	LFFLFIMACAEGLQVSALALAKTHTAS---FKDKSPLAYRTTQLLY--AGRNMQAFLVGR
Acanthoeca spectabilis SITAlpha	IFFLFIMACAEGLQVSALALAQVPSHE---YKNKAPLAYRLCQIMF--AGRNMQAFLVGR
Helgoeca nana SITAlpha	LFFIYLIACAEGMQVSLLAMAKIPSHE---YKQRAPLAYRTTQLAY--SGRNMQAFLVGR
Savillea parva SITAlpha	LFFLYLIACSEGMQVSALALAKVPSHE---YKQKSPLAYRTTQLFY--AGRNMQAFLVGR
Diaphanoeca grandis SITAlpha	IFFLFLMACAEGLQVSALALANTHTAA---FKKTSPLAYRTCQLLY--AGRNMNAFLVGR
Diplotheca costata SITAlpha	CLFLFIMACAEGLQVSALALAGTHTAE---FKQRAPLAYRTCQLCF--AGRNMQAFLVGR
Stephanoeca diplocostata SITBeta	IFFLFFIGCCEGFQIAAVTLAKVPSSE---LRTKYPVAYRTALMLY--TGRNLQAFLVGR
Diaphanoeca grandis SITBeta	LFFMFVIGCCEGFQIAAMKLASKSTSE---LKVNYKQAYRTTQTLF--AGRNLQAFLVGR
Diplotheca costata SITBeta	IFFLFLIGCCEGFQIAAVALSKIPAEE---LKEHYPMAYTISQTLY--SGRNLQAFLVGR
Prymnesium neolepis SIT	ILLCTVVHCTEGLQVALFAMQKMDPEE----FKVHAGAEANGAVAF--AGNNLQCFLIGR
Scyphosphaera apsteinii SIT	MLLCGLVHCTEGLQVALFAMQKMDPEE----IKAHAGAQANAAVAF--KGNNLQAFLIGR
Phaeodactylum tricornutum SIT3	FILMAVVGIMEGMQIALFAVVNLPKEE----LRKHPIAYANCGLTF--SGQNLQAFLIGR
Synedra acus SIT	FILMAVVGMMEGMQIALFAVVNMPEEE----LRNSPIAYANCQLTF--AGQNLQAFLIGR
Phaeodactylum tricornutum SIT2	FLLMSVVGLLEGMQIAFFAVAKIPKSE----RGDSVFAKKTCELLFKGEGNNLPGFMIGR
Phaeodactylum tricornutum SIT1	FVLMSVVGLLEGMQIAFFAVAKIPKAE----RGDSVFAKKTCDLLFKGDGNNLPGFMIGR
Cylindrotheca fusiformis SIT3	ILLMAVVGLLEGMQIAFFAVAKIPKAE----RGDHPFARKTCELVFRGNGRNLPGFMVGR
Cylindrotheca fusiformis SIT1	ILLMSVVGLLEGMQIAFFAVAKIPKAE----RGDHPFARKTCELLFKGKGRNLPGFMVGR
Cylindrotheca fusiformis SIT5	VLLMSVVGLLEGMQIAFFAVAKIPKAE----RGDHPFARKTCELLFRGKGRNLPGFMVGR
Cylindrotheca fusiformis SIT4	VLLMSLVGLLEGMQIAFFAVAKIPKAD----RGDHPFARKTCELLFKGNGRNLPGFMVGR
Cylindrotheca fusiformis SIT2	VLLMSLVGLLEGMQIAFFAVAKIPKAD----RGDHPFARKTCEVLFKGNGRNLPGFMVGR
Nitzschia alba SIT	FLLMSVVGLLEGMQIAFFAVAKLRKEE----RGEHPMAMRTCELLFRGEGKNLPGFMVGR
Thalassiosira pseudonana SIT2	FFFMSIVGMLEGMQIAFFAVAKLPASE----RGTSFFGKKTCELLFRGNGENLPGFMIGR
Thalassiosira pseudonana SIT1	FFFMAVVGMLEGMQIAFFAVAKLPANE----RGTSFFGRKTCEILFKGNGENLPGFMIGR
Skeletonema costatum SIT	FFLMCIVGMLEGMQIAFFAVAKLPASE----RGTTFFGRKTCDLLFKGNGQNLPGFMIGR
Thalassiosira pseudonana SIT3	FVLLTIVGMLEGMQIAFLATSKMRREQ----RGTSFFGKKTVEVISKKNGQNLPAFFIGR
Dinobryon sp. SIT	ILALTILFYLEGLMIAIVGTQYWDPET---FRDIYPRAYRMHKFIN--KPDNVKRFIIGR
Mallomonas sp. SIT*	IIFLGLLFYLEGLMICIVATQFWDKET---FKELYPRAYMLHELVN--RPENLKRFIIGR
Ochromonas distigma SIT	CCCICFLSYLEGSQVAILVAEEYDLKK---YEQSHPRAYALMVRAK--TEKNVRRYLIGR
Ochromonas sp. SIT	CCCILFLAQLEGLQVAMLVKEEEDLTP---YAXTHPRAYALMQKAT--FEKNVRRFLIGR
Paraphysomonas bandaiensis SIT1	CCCILFLAHLEGLQVAILVAEAKDPEP---YAQSYPRAYSLMKRAT--YEKNVRRFLIGR
Paraphysomonas imperforata SIT1	SCCILFLALLEGLQVGILVRENKDPEE-NNYPETHPRATALMKRAT--HEKNVRRFLIGR
Paraphysomonas imperforata SIT2	FLSMVVIVYCEGMKVSIVSTTHIDSEE---MKETHKTAYHVHKLLNVDTSEGVKKFLLGR
Paraphysomonas bandaiensis SIT2	FLALVIVTYCEGLKVAVVSTTHLDSED----MKAYPTAYKIHRLLNSSVSEGVKKFLLGR
Noctiluca scintillans SIT-L	FASITFLFYLEGLQVGLLALFRQDLRG---RERQFPGTERVFALAS--KGDNLQRFLLGR

TM7 (2)

TM8 (3)

```
Rhodococcus opacus B4 SIT-L           QAGVVLVVFGIAEVTRTA-----------------GMTSL-----------PF-TSIGIP
Rhodococcus opacus SIT-L              QAGVVLVVFGIAEVTRTA-----------------GMTSL-----------PF-TSIGIP
Synechococcus KORDI                   QFFVILTVFVIAQMTSFP-----------------RMEALPF---------TNYPVSQLP
Synechococcus CC9616 SIT-L            QFFVILTVFVIAQITSFP-----------------GMEFLPF---------TSYPVMQLP
TARA_004 SRF 0.22-1.6                 QFFVILTVFVIAQITSFP-----------------RMEFLPF---------TDYPVAQLP
TARA_142 SRF 0.22-3                   QFFVIVTVFVIAQITSFP-----------------QLSYLPF---------SNVPVSDLP
Florenciella parvula SIT-L            QFFVVFVVFLCAQLTTYP---------------------------------EL-PKGGVP
Phaeodaria OSH121 SIT-L1              QFFVVFVVFLCAQLTTYA---------------------------------TL-SIPWMP
Phaeodaria OSH121 SIT-L2              QFFVVFVVFLCAQLTTYA---------------------------------TL-SIPWMP
Ceratium fusus SIT-L                  QFFVIFNVMLISQGSTFP---------------------------------DLERPSWFP
Dinophysis acuminata SIT-L            QFGVIFVVMMISQVSTFP---------------------------------DLERPSWVP
Calanus finmarchicus SIT-L            QVFVVCLVFFAAKLTTIH-----------------GNS-E-----------SG-FLFYVP
Platynereis dumerilii SIT-L           QVCVVVLVFFAAKLTTIH-----------------LQD-G-----------DT-FLFPVP
Capitella teleta SIT-L                QVCVVVLVFLAAKLTTLE-----------------LQD-G-----------KD-FLFPVP
Ciona intestinalis SIT-L              QA-----VFFIAKLTTIV-----------------LEDLN-----------SD-FFFPVP
Molgula tectiformis SIT-L             QVFVIFIVFFISKITTVY-----------------LED-L-----------SD-FLFPVP
Phallusia mammillata SIT-L            QVLVVFLVFFIAKLTTIS-----------------LQNLE-----------TD-FFFHVP
Scyphosphaera apsteinii SIT-L         QMSVIFLVFVVAHITSSE------------------------------------NLDFLP
Coccolithus pelagicus SIT-L           QMGVIFLVFFIANITSSE----------------------------------N-LDVFEP
Calcidiscus leptoporus SIT-L          QMSVIFLVFFIARITSSD----------------------------------N-LEVFEP
Rosalina sp. SIT-L                    QVLLVGLVFLCARITTFE----------------------------------T-FPTDLP
Elphidium margaritaceum SIT-L         QVLVVMTVFVAARITTFE---------------------------------------GFW
Ammonia sp. SIT-L1                    QVLVVGTVFIAARITTF---------------------------------------DGVW
Ammonia sp. SIT-L2                    QVLLVYIVFLVGRITTFS----------------------------------V-FIADIP
Savillea parva SITBeta                QVFVAMMMVLLAKVTTYA-----------------GSDGELV---------RG-DDWGMG
Acanthoeca spectabilis SITBeta        QVFVAMMMVLLAKVTGYA-----------------GRDGELV---------EG-DDWGMG
Helgoeca nana SITBeta                 QVFVAMMMVLLGKVTSYA-----------------GSEGVLV---------EG-DDWGMG
Stephanoeca diplocostata SITAlphaBC   QTIVAMMMVLLARVTSYA-----------------GSDGELL---------EG-GDWGMG
Stephanoeca diplocostata SITAlphaA    QTIVAMMTVLLARVTSYA-----------------GSDGELL---------EG-GDWGMG
Acanthoeca spectabilis SITAlpha       QAMVAMMMILLARVTSYA-----------------GSDGILV---------SG-TDWGMG
Helgoeca nana SITAlpha                QFTVAMMIIILAKVTSYA-----------------GSEGILI--------GDG-EDWGMG
Savillea parva SITAlpha               QFTVAMMIVLLSRVTSYA-----------------GSEGKLI--------GTG-EDWGMG
Diaphanoeca grandis SITAlpha          QFLTAMCMVLLGRVTSYT-----------------GSEGVI----------SG-DDWGMG
Diplotheca costata SITAlpha           QFFVAMMMVLLARVTGYA-----------------GSDGEI----------DG-NDWGMG
Stephanoeca diplocostata SITBeta      QVFVAMLMVLLAKATGYA-----------------GTDGDLV---------TG-SDWGMG
Diaphanoeca grandis SITBeta           HVFVAMMMVLLGRVTGFARPCPDAPIDASTGFPIDPTDGNYD---------SE-CIFNFP
Diplotheca costata SITBeta            QVLSAIMMVLLARVTTFS-----------------DADD---------------DVWGFA
Prymnesium neolepis SIT               QALTAMSMFVLSTICGVQ-------ANLTKEVEFTNSTGT-------FTKEEGYTILGLG
Scyphosphaera apsteinii SIT           QALTALSMFILSLITSVQ-------ADLTKTSTSTLINGTEISSGTLNTVEEGYTILGLG
Phaeodactylum tricornutum SIT3        QICVTVCTFVIARITSVS-----------------VNTDI-----------GENNVFGVS
Synedra acus SIT                      QIFVATCMFIVARIASPT-----------------YGKD------------DA-NIFGIS
Phaeodactylum tricornutum SIT2        QLCVVSCMFFIARVTSVE-----------------IAEG------------EE-NIFGVS
Phaeodactylum tricornutum SIT1        QLCVVSCMFFIARVTSVE-----------------IAEG------------EE-NIFGVS
Cylindrotheca fusiformis SIT3         QITVTLCFFIIARVTTLD-----------------VDVGV-----------DD-NIFGVS
Cylindrotheca fusiformis SIT1         QMTVTLCFFIIARVTTLD-----------------IEVGV-----------DD-NIFGVS
Cylindrotheca fusiformis SIT5         QMTVTLCFFIIARVTTLD-----------------IEVGV-----------DD-NIFGVS
Cylindrotheca fusiformis SIT4         QMTVTLCFFIIARVTTLD-----------------IEIGV-----------DD-NIFGVS
Cylindrotheca fusiformis SIT2         QMTVTLCFFIIARVTTLD-----------------IEIGV-----------DD-NIFGVS
Nitzschia alba SIT                    QMTVTLCFFVIARVTTLD-----------------IEVGV-----------DE-NVFGVS
Thalassiosira pseudonana SIT2         QLTVVCSFFLVGSFTSLT-----------------IEPGM-----------GE-NIFGVS
Thalassiosira pseudonana SIT1         QLTVVCSFFLVGSFTSLI-----------------IEPGQ-----------GE-NIFGVS
Skeletonema costatum SIT              QLTVVASFFIVASITSMN-----------------IQPGN-----------EDGNIFGVS
Thalassiosira pseudonana SIT3         QLMVVGCFFILARVTTPD-----------------VEVGT-----------GN-NIFGVS
Dinobryon sp. SIT                     QFFTVLTNFLLAQVFTFN----------------------------------------TW
Mallomonas sp. SIT*                   QFFTVLSNIMLAEVTAFP---------------------------------SW-RHQTWN
Ochromonas distigma SIT               QFLVIFVVFLINQCTIFP---------------------------------EI-SQLGID
Ochromonas sp. SIT                    QFLVIFVVFLINQCTIFP---------------------------------DI-SQLGID
Paraphysomonas bandaiensis SIT1       QFFVIFVVFLINQCTIFP---------------------------------EI-SKLGIN
Paraphysomonas imperforata SIT1       QFFVIFVVFLINQCTIFP---------------------------------DI-QQFGVN
Paraphysomonas imperforata SIT2       QMLVVSLGFFIASLTHFA-----------------------------------GLKEHVP
Paraphysomonas bandaiensis SIT2       QLTVVPLGFLIAALTHFE---------------------------------NL-NRDNFP
Noctiluca scintillans SIT-L           QFLVVFIVFLCSQVCTLH---------------------------------VR-RPVGIP
```

TM8 (3)

```
                                              TM9 (4)                    TM10 (5)

Rhodococcus opacus B4 SIT-L                   HTAE-ILLGIGVPGALIVLCIGQVAPQLVAARKPAGMMNTLPMAGAFTVTRWIANLGLAT
Rhodococcus opacus SIT-L                      HTAE-ILLGIGVPGALIVLCIGQVAPQLVAARKPAGMMNTLPMAGAFTVTRWIANLGLAT
Synechococcus KORDI                           DWINLICFQFGFLGALPVLWTAQLIPQYFANRHPDMFLNFPGNFLVVRLCLLIESIGPTK
Synechococcus CC9616 SIT-L                    DWINLICFKFGFLGALLVLWTAQLIPQYFANRHPDMFLNFPGNFLVVRLCLLIESIGPTK
TARA_004 SRF 0.22-1.6                         DWINLICFKFGFLGALLVLWTAQLIPQYFANRHPDMFLNFPGNFMVVRLCLLIESIGPTK
TARA_142 SRF 0.22-3                           DWINLICFKLGFLGALIVLWTAQLIPQYFANRYPDLFLSFPGNSQIVRLCLLIESIGPTK
Florenciella parvula SIT-L                    GWLFTAIIETGLPGALVVLAFGQLMPQLLAATHPVVFMNLHGTWLVVQIALGFESLGVTH
Phaeodaria OSH121 SIT-L1                      KTLFIVLIETGLPGALIVLAFGQLMPQLVAATHPITFMNLPGTWSVIQLCLCFEAVGVTH
Phaeodaria OSH121 SIT-L2                      QTLFIVLIETGLPGALIVLAFGQLMPQLVAATHPITFMNLPGTWSVIQLCLCFEAVGVTH
Ceratium fusus SIT-L                          QPLWVAYVDTALYGALCVLAFGQLMPQLIGARHAVAFCNLPGSPSVLCLTLFCEATGIAH
Dinophysis acuminata SIT-L                    EVLWIGYVGTALYGALVVLAFGQLMPQLIGARHAVFFLNLPGSYSVLALTLFMEATGIAH
Calanus finmarchicus SIT-L                    DWVQALFLETGLLACVVVVIIAQLMPQIVASLYPTQFLELLVMRPAYWACIILETSGVTH
Platynereis dumerilii SIT-L                   QWVRSAFFETGLLACVVVVILAQLMPQIVAAKFPVHFLQILIMYLAYYVCVFLEATGITH
Capitella teleta SIT-L                        QWVQSAFFETGFLSCIVVVILAQLMPQIAAAQFPVHFLQLFIMKPAYYFCVFLEMTGLTH
Ciona intestinalis SIT-L                      HWLYVSLLETGFITCVLVVIVAQLMPQIVAAKFPVHFLDMRIMFIAYYACIFVEMSGVTH
Molgula tectiformis SIT-L                     RAVYYIFLETGFLTCVMVVIIGQLMPQILASKCPVHFLELRVMKFAYFACICVESTGVTH
Phallusia mammillata SIT-L                    NWLYSSLLETGFLTCILVVIVSQLMPQIVAAKYPVHFLNLRIMYFFYHVCTFIEATGLTH
Scyphosphaera apsteinii SIT-L                 DPFFAFMLKSGLFGAILVCISAQLVPQVMAAKYPVQFLDIPGMWLALQITFVLEATGISH
Coccolithus pelagicus SIT-L                   SEVSTFVLKAGLLGAVLVCIIAQLTPQIIAAKFPVHFLNLRGMYSALVVCLILETSGICH
Calcidiscus leptoporus SIT-L                  SEVSAFVLKAGLLGAVIVCIVAQLTPQVVAAKYPVHFLNLRGMYSALVVCLILEASGICH
Rosalina sp. SIT-L                            NWVVQSVMATGFLGVILVVIIAQLTPQVLAAAYPVEFLNLPGIPIAFGICLVTEATGLAH
Elphidium margaritaceum SIT-L                 DEMH-ALSYSGFLGVILVVVVAQLTPQVLAAAYPVEFLNMYGMKIAFRACLIVEATGLVH
Ammonia sp. SIT-L1                            DAIH-GLTFSGFLGVVLVVIVAQLTPQVLAAAYPTEFMNLPGMKIAFWACLLVEATGLVH
Ammonia sp. SIT-L2                            DWVVNSFMYSGFLGVILVVIIAQLTPQVLASAYPVEFLNLYGMNVAFGICLLVEWSGIAH
Savillea parva SITBeta                        QGFNK-LLQTGILGAIFVVNVGQLSFRMMASSFPVVFISNPVVYALLRIALMVEATGVVN
Acanthoeca spectabilis SITBeta                SDFNEILLQTGILGAVFVVNVGQLSFRMAASSFPVLFINNFVLRALLRVALVVEATGIVN
Helgoeca nana SITBeta                         TEFNRILLQTGILGAIFVVNVGQLSFRMAASCFPVMFINNFVVNYLLQVVLVVEATGVVN
Stephanoeca diplocostata SITAlphaBC           KGFNQWLLQTGILGAVLVCNVAQLASQVTASIFPVELINNHIMHILLRLMLLIEASGVVN
Stephanoeca diplocostata SITAlphaA            KGFNQWLLQTGILGAVLVCNVAQLASQVTASIFPVELINNHVMHILLRLMLLIEASGVVN
Acanthoeca spectabilis SITAlpha               EGFNKGLLQTGFLGAILVVNVAQLASQVLASLFPIPVINNTLMRVLLNIMLFIEASGVVN
Helgoeca nana SITAlpha                        EGFNAGLLQTGFLGAILVVNVGQLASQVAASVFPVACINNHVMHFLLRLMLFIEFSGIVN
Savillea parva SITAlpha                       EGFNAGLLQTGFLGAILVVNVGQLASQVAASIFPVACINNYVMNFLLRFMLFIEFSGIVN
Diaphanoeca grandis SITAlpha                  KGFNEGLLQTGFLGAILVVNVAQLASQVMASIFPITFINNHFLYLLLQLMLLVETCGVVN
Diplotheca costata SITAlpha                   RAFNEGLLQTGFLGAIFVVNVAQLASQVLASVFPISFINNHFLNWLLRIMLLVEASGVVN
Stephanoeca diplocostata SITBeta              RRFNEVLLQTGILGAIFVVNVGQLSFRMAATAFPILFINNYVMYALLRVALLVEMTGAFN
Diaphanoeca grandis SITBeta                   EWFMKGFMQSGIIGAIVVVNIAQLSFRMFAAGFPVIFINNRLMFFLLKVCLFVEATGIVN
Diplotheca costata SITBeta                    QWAKDGFLTTGILGAVFVVNVGQLSFRMMAINFPTVFINNFIMYGLLSVALVVEATGVVN
Prymnesium neolepis SIT                       TGISTAFLETGLSGAVVLTIMASLSGQVIASAFPLGFCGFPGMWIVLYACLLTEFIGLTH
Scyphosphaera apsteinii SIT                   NTFSSMFLENGLVGAVVLTICASLSGQVIASAFPLGFCGLPGMWIVLDACLLVEFIGLTH
Phaeodactylum tricornutum SIT3                DGIQ-NFFNTGMLGALVTTIVASLAWRIIASSLPVAFMSNPLIYLIIRLCLILEATGLCS
Synedra acus SIT                              DGFQ-SFLNSGLTGAVITTVIGSLAWRIIASSFPLAFLSNPLIYIIIRVCLVLESVGICS
Phaeodactylum tricornutum SIT2                DGVQ-KLFDTGLLGAIITTIVASISWQLVASAFPIAFLSNPFTYIFLRICLLLEAIGICS
Phaeodactylum tricornutum SIT1                DGFQ-KLFDTGLLGAIITTIVASISWQLVASAFPIAFLSNPFTYIFLRICLVLEAIGICS
Cylindrotheca fusiformis SIT3                 DGVQ-EFFNLGFLGAIITTILASIAWQLVASAFPIAFLSNPIVYIMLRIVLLIQSTGICA
Cylindrotheca fusiformis SIT1                 DGIQ-EFFNLGFLGAIITTILASIAWQLVASAFPIAFLSNPIVYIVLRIVLLIEATGICA
Cylindrotheca fusiformis SIT5                 DGIQ-EFFNLGFLGAIITTILASIAWQLVASAFPIAFLSNPIVYIMLRIVLLIEATGICS
Cylindrotheca fusiformis SIT4                 DGIQ-EFFNLGFLGAIITTILASIAWQLVASAFPIAFLSNPVVYIVLRIVLLIEATGICA
Cylindrotheca fusiformis SIT2                 DGIQ-EFFNLGFLGAIITTILASIAWQLVASAFPIAFLSNPVVYIVLRIVLLIEATGICA
Nitzschia alba SIT                            DPIQ-EFFNMGFLGAIITAILGSIAWQLVASAFPLEFLSNPMVYIFLNLALALEATGVVS
Thalassiosira pseudonana SIT2                 DGAQ-AFLNYGFQGAVITTILASITWQLAASAFPIAFLNNPATFILLVIALFLERIGLCA
Thalassiosira pseudonana SIT1                 DGAQ-AFLNYGFQGAVITTILASITWQLAASAFPIAFLNNPVTFILLVVALFLERIGLCA
Skeletonema costatum SIT                      DGAQ-AFLNLGFHAAVITTILASITWQLAASAFPIAFLNNPVTYVLLVFALFLEWTGLCA
Thalassiosira pseudonana SIT3                 DGAQ-AFLNTGLHAALLMTILASNTWKLAASTFPVAFVNLPFTYILLWCGLILEATGICS
Dinobryon sp. SIT                             EPGSVNPXXSGLVGVLIILSCAQLLPELLAAEYPLRFMNMYGSYTIAYASLFFDAIGVGH
Mallomonas sp. SIT*                           PAGFYAIIQSGLIGALTTLAFAQLCPELLAAEFPLRFMNMYGSFMVVRICLFIESIGIGH
Ochromonas distigma SIT                       DAAWFIFIQLGLPTALNVLCFAQLPAQLLGNQDPMMYMNRYGPRATMEFCIYAEMTGIAH
Ochromonas sp. SIT                            SAVWFVFIQLGLPTALNVLCFAQLPA----------------------------------
Paraphysomonas bandaiensis SIT1               NGVWFVFVQLGFPTALNILCFAQLPAQLLGNQDPWLFMNRYGPRFTLEVCLFTEMTGLAH
Paraphysomonas imperforata SIT1               PALWFVVIQLGLPTALNVLCFGQLPAQLLANHDPMLFMNRPGPRFTLEVCLFTEMTGIAH
Paraphysomonas imperforata SIT2               YAIYFMVVTAALPGVMVFLEVAQLTPQLLAEQNNIAFINLPGSYYLAKFLLFVEACGIMN
Paraphysomonas bandaiensis SIT2               GGLYFIVVTAGLPGVLILLQIAQLTPQLLAQQNSILFLNLPGSYLLVLWTLAVESLGIVN
Noctiluca scintillans SIT-L                   SALWWPVVKTGLPGSLVVLAFGQLTPQVLASRCPLQFCNTVGAYIALVVSLAVESAGATD

                                              TM9 (4)                    TM10 (5)
```

TM10 (5)

```
Rhodococcus opacus B4 SIT-L        PSKWLMAGFPGTERIATAPRQRYLSDS---LDAEGFG-VESIAH----------------
Rhodococcus opacus SIT-L           PSKWLMAGFPGTERIATAPRQRYLSDS---LDAEGFG-VESIAH----------------
Synechococcus KORDI                PANWMCDVLLKAVE--------DDNP----------------------------------
Synechococcus CC9616 SIT-L         PANWMCDVLLKAVE--------DDRPK------------QLKI-----------------
TARA_004 SRF 0.22-1.6              PANWMCDVLLKAVE--------DDRPK------------QLKS-----------------
TARA_142 SRF 0.22-3                PANWMSFVILNAVK--------KHQQG---------------------------------
Florenciella parvula SIT-L         FSWLLTSMVKYVFK--------MDAGGDPSMHLHGD--GSGSGS----------------
Phaeodaria OSH121 SIT-L1           FSWVLSWTVKKMFN--------MGEKE---KIKKSN--KSGNVM----------------
Phaeodaria OSH121 SIT-L2           FSWVLSATVKIMFQ--------MGAKE---KIKKSV--KSGNVM----------------
Ceratium fusus SIT-L               FAWVLTDMVLRMAG--------MRKTA---KEVDLE--SGDVLS----------------
Dinophysis acuminata SIT-L         FSWVLTDLCLYLCG--------FRDKE---EDADVE--QGRTA-----------------
Calanus finmarchicus SIT-L         ICWVLAWGLSKMFR--------MKEEV---TAGGVQ--MDKHG-----------------
Platynereis dumerilii SIT-L        ICWVLSHLMSIAM-----------------------------------------------
Capitella teleta SIT-L             SCWLLSGLMTLACG--------MKDDD---TTKNPN--EYEHPE----------------
Ciona intestinalis SIT-L           ACWLLSHLFGKLAG--------MKDKI---EVKVSP--SYPDSD----------------
Molgula tectiformis SIT-L          ACWVLSYV----------------------------------------------------
Phallusia mammillata SIT-L         ACWVLSYLLGKIAG--------MASEE---IYFKKT--LSESD-----------------
Scyphosphaera apsteinii SIT-L      ATWLMVTFLNIPGM--------WLTLQ---ICFLLE--ATGIS-----------------
Coccolithus pelagicus SIT-L        ASWLFARATAWMVG--------WERSS---SPAKML--HLLRL-----------------
Calcidiscus leptoporus SIT-L       ATWLFARATMRLVG--------WGRSS---SPAEML--RSLGL-----------------
Rosalina sp. SIT-L                 AVWLLCYGVRTAFY--------SCLCK---YHTLWL--MDVKR-----------------
Elphidium margaritaceum SIT-L      AVWFLCVVTKSMCGACL----CRQSDEMDDYAFERV--ASRSP-----------------
Ammonia sp. SIT-L1                 AVWFLCVVVKKGCYKCV----CAVSDDDDDVKFDRV--ASKSAMRGLKRHRDDDDDEDDD
Ammonia sp. SIT-L2                 AVWLLCWSIKNAFY--------KCLCQ---YRHLRM--MQKRM-----------------
Savillea parva SITBeta             SCWPLAWGLEALLG--------LRDDP---VVLDNN--GGGATN----------------
Acanthoeca spectabilis SITBeta     ACWPLTWGIDRLLQ--------LKPDP---VSGELA--TVSPQ-----------------
Helgoeca nana SITBeta              SCWPLAWGLDSMLG--------LTPDN---FDGDGDEWVNTAA-----------------
Stephanoeca diplocostata SITAlphaBC ACWPLAWGVDALFG--------LEKDP---FDGDEN--VKTPA-----------------
Stephanoeca diplocostata SITAlphaA ACWPLAWGVDSLFG--------LEHDP---FDGDET--VKTPA-----------------
Acanthoeca spectabilis SITAlpha    ACWPLTWLLDAMTG--------LPKDP---FWGDDA--VNTPA-----------------
Helgoeca nana SITAlpha             SCWVLTWALDAITG--------LPKDP---FDGDEA--VKTPA-----------------
Savillea parva SITAlpha            SCWVLTWCLDAITG--------LPLDP---FDGDET--VDTPA-----------------
Diaphanoeca grandis SITAlpha       ACWPLAALIDNLLK--------IPKDP---FEDDKD--VDTFN-----------------
Diplotheca costata SITAlpha        ACWPLARFFDWAFG--------MVKDP---FEDDED--IKTPA-----------------
Stephanoeca diplocostata SITBeta   SCWVLAWAVDHTLR--------LQHDP---FDGEEN--IKSPA-----------------
Diaphanoeca grandis SITBeta        SCWPLAWGLDGFCK--------LNPDP---FDGDGD--VETPA-----------------
Diplotheca costata SITBeta         SCWPLAWGLDRILK--------LQSDL---NVFEERD-LQTPP-----------------
Prymnesium neolepis SIT            FAWPLADGVQALMGNL------MQNDE---VYLGGK--HAGAI-----------------
Scyphosphaera apsteinii SIT        FSWPLADGVQKLMGKY------MQDDE---VYLHGS--KEFAR-----------------
Phaeodactylum tricornutum SIT3     AAWVLALLQKSLAG--------YQRDD---VYIGTA--GERVV-----------------
Synedra acus SIT                   GAWVFGRFNKLIAG--------YQPDE---VYLEGA--ERHTS-----------------
Phaeodactylum tricornutum SIT2     GAWVLAAIHKKIAG--------FQRDE---VYIGTA--EERAA-----------------
Phaeodactylum tricornutum SIT1     GAWVLAAIHKKVAG--------FQRDE---VYIGTA--EERAA-----------------
Cylindrotheca fusiformis SIT3      SAWFLGMIHKKIAG--------FQEDE---VYVGTA--EERAA-----------------
Cylindrotheca fusiformis SIT1      GAWFLGMIHKKVAG--------FQLDE---VYVGTA--EERAA-----------------
Cylindrotheca fusiformis SIT5      GAWFLGMIHKKVAG--------FQLDE---VYVGTA--EERAA-----------------
Cylindrotheca fusiformis SIT4      GAWFLGMIHKKVAG--------FQLDE---VYVGTA--EERAA-----------------
Cylindrotheca fusiformis SIT2      GAWFLGMIHKKVAG--------FQLDE---VYVGTA--EERAA-----------------
Nitzschia alba SIT                 GAWFLGIIHKNVAG--------FQEDE---VYIGTP--EERAA-----------------
Thalassiosira pseudonana SIT2      GAWVLASVQKKIMK--------FEYDE---VYVGTP--EERIE-----------------
Thalassiosira pseudonana SIT1      GAWVLASAQKKAMK--------FEYDE---VYVGTP--EERIA-----------------
Skeletonema costatum SIT           GAWVLARVMKKALK--------YEYDE---VYVGTP--EERAA-----------------
Thalassiosira pseudonana SIT3      GAWVLARILKRVTK--------LKYDE---EYVGTP--ERPTL-----------------
Dinobryon sp. SIT                  CAWTIYFVTRQLCC--------SKHLD---------------------------------
Mallomonas sp. SIT*                CSWVFYFLTRKLFC--------GAHED---IDEIRP--GVLRVN----------------
Ochromonas distigma SIT            FSWVVSSFSKMTWF--------RISRE---CAASPI--KDPVI-----------------
Ochromonas sp. SIT                 ------------------------------------------------------------
Paraphysomonas bandaiensis SIT1    FSWVATSFSKATWF--------PITVS---AISEIEQYKDLES-----------------
Paraphysomonas imperforata SIT1    FSWVATAISMFTWF--------KVSPT---ALDDLATFKDLEA-----------------
Paraphysomonas imperforata SIT2    VAWLIYYGLDNCLC--------SKPRR---IAEEXDE-LDTTA-----------------
Paraphysomonas bandaiensis SIT2    FTWVLYFAIDKLLC--------RKRKD---GSV-----YSRPT-----------------
Noctiluca scintillans SIT-L        FPKLLMASCWRFVF--------LFRPA---AVHEKS--LDLRS-----------------
```

TM10 (5)

Supplementary Figure 1. Full SIT and SIT-L alignment of transmembrane domains and conserved features highlighted. The alignment was created using MAFFT (see Methods). Transmembrane region annotations are based on matching the 10 predicted SIT transmembrane domains with the five transmembrane domains predicted for SIT-Ls (numbered in brackets). Conserved EGXQ and GRQ motifs highlighted in yellow, conserved xQxxxQ motif in bright green, with variant residues noted in light blue. Conserved individual residues are in bold and underlined; bright red=positive, magenta=negative, dark green=hydroxylated. Asterisk denotes *Mallomonas* sp. sequence with strong evidence for being an incomplete SIT.

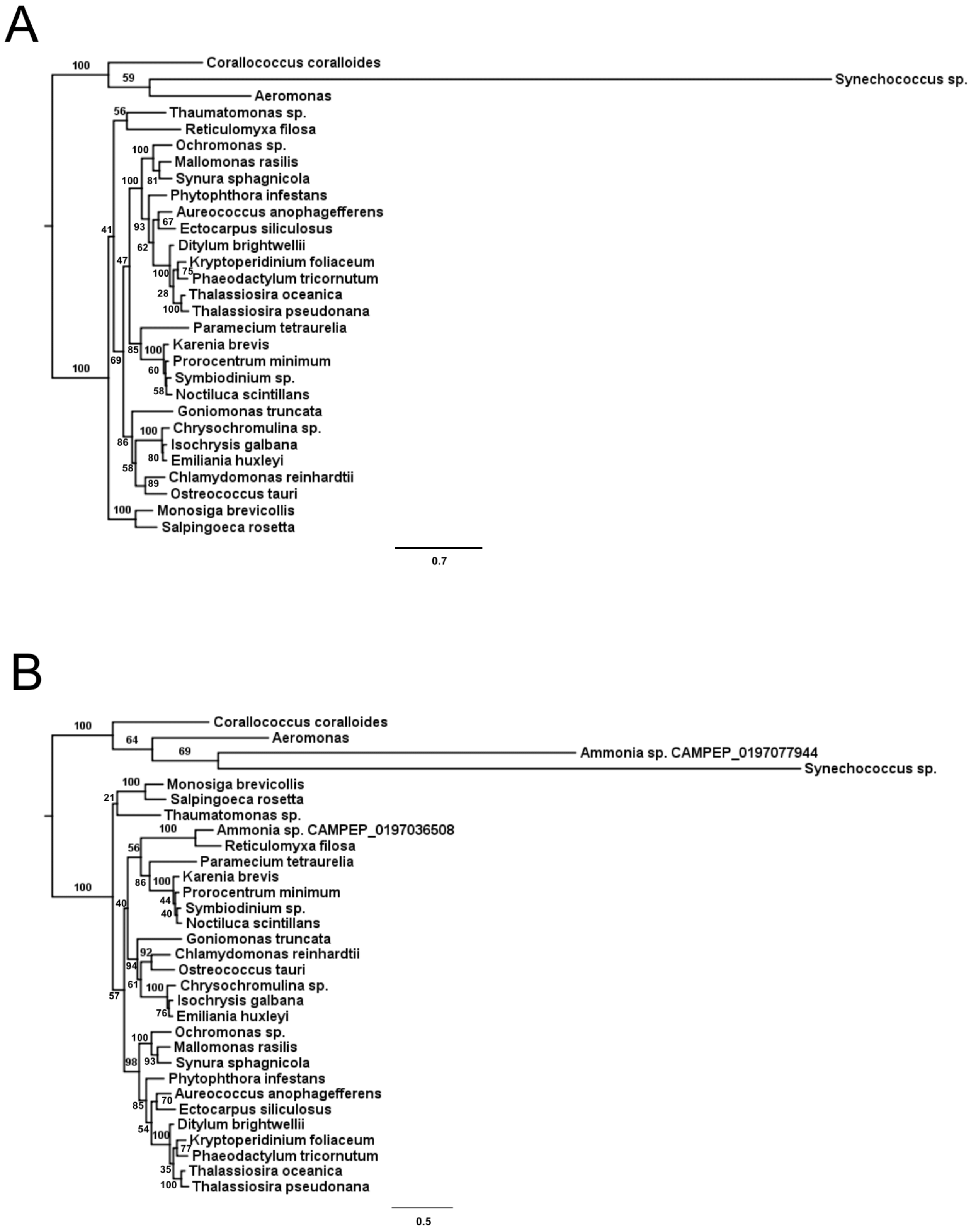

A
Corallococcus coralloides
Synechococcus sp.
Aeromonas
Thaumatomonas sp.
Reticulomyxa filosa
Ochromonas sp.
Mallomonas rasilis
Synura sphagnicola
Phytophthora infestans
Aureococcus anophagefferens
Ectocarpus siliculosus
Ditylum brightwellii
Kryptoperidinium foliaceum
Phaeodactylum tricornutum
Thalassiosira oceanica
Thalassiosira pseudonana
Paramecium tetraurelia
Karenia brevis
Prorocentrum minimum
Symbiodinium sp.
Noctiluca scintillans
Goniomonas truncata
Chrysochromulina sp.
Isochrysis galbana
Emiliania huxleyi
Chlamydomonas reinhardtii
Ostreococcus tauri
Monosiga brevicollis
Salpingoeca rosetta
0.7
B
Corallococcus coralloides
Aeromonas
Ammonia sp. CAMPEP_0197077944
Synechococcus sp.
Monosiga brevicollis
Salpingoeca rosetta
Thaumatomonas sp.
Ammonia sp. CAMPEP_0197036508
Reticulomyxa filosa
Paramecium tetraurelia
Karenia brevis
Prorocentrum minimum
Symbiodinium sp.
Noctiluca scintillans
Goniomonas truncata
Chlamydomonas reinhardtii
Ostreococcus tauri
Chrysochromulina sp.
Isochrysis galbana
Emiliania huxleyi
Ochromonas sp.
Mallomonas rasilis
Synura sphagnicola
Phytophthora infestans
Aureococcus anophagefferens
Ectocarpus siliculosus
Ditylum brightwellii
Kryptoperidinium foliaceum
Phaeodactylum tricornutum
Thalassiosira oceanica
Thalassiosira pseudonana
0.5

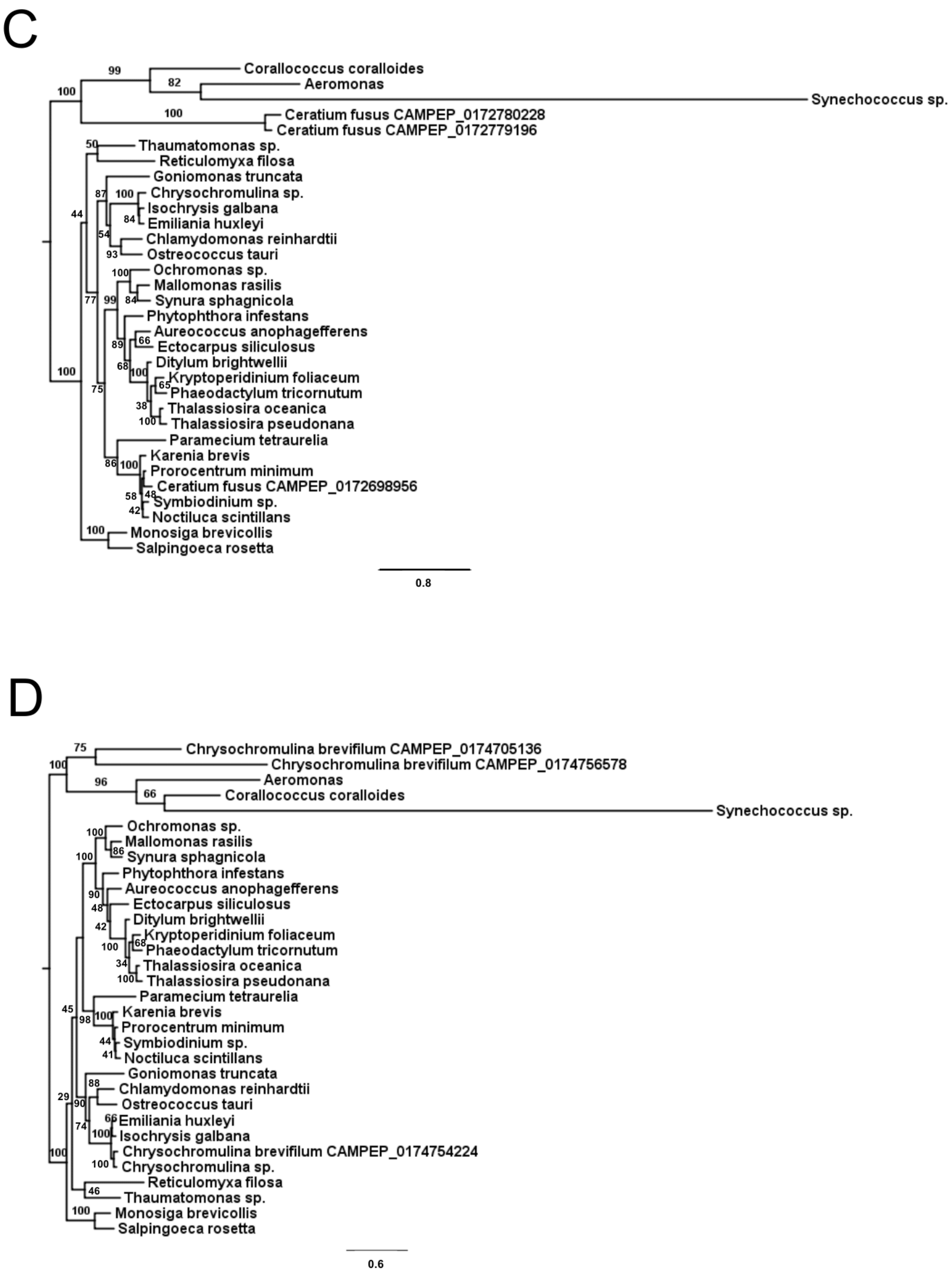
C
Corallococcus coralloides
Aeromonas
Synechococcus sp.
Ceratium fusus CAMPEP_0172780228
Ceratium fusus CAMPEP_0172779196
Thaumatomonas sp.
Reticulomyxa filosa
Goniomonas truncata
Chrysochromulina sp.
Isochrysis galbana
Emiliania huxleyi
Chlamydomonas reinhardtii
Ostreococcus tauri
Ochromonas sp.
Mallomonas rasilis
Synura sphagnicola
Phytophthora infestans
Aureococcus anophagefferens
Ectocarpus siliculosus
Ditylum brightwellii
Kryptoperidinium foliaceum
Phaeodactylum tricornutum
Thalassiosira oceanica
Thalassiosira pseudonana
Paramecium tetraurelia
Karenia brevis
Prorocentrum minimum
Ceratium fusus CAMPEP_0172698956
Symbiodinium sp.
Noctiluca scintillans
Monosiga brevicollis
Salpingoeca rosetta
0.8
D
Chrysochromulina brevifilum CAMPEP_0174705136
Chrysochromulina brevifilum CAMPEP_0174756578
Aeromonas
Corallococcus coralloides
Synechococcus sp.
Ochromonas sp.
Mallomonas rasilis
Synura sphagnicola
Phytophthora infestans
Aureococcus anophagefferens
Ectocarpus siliculosus
Ditylum brightwellii
Kryptoperidinium foliaceum
Phaeodactylum tricornutum
Thalassiosira oceanica
Thalassiosira pseudonana
Paramecium tetraurelia
Karenia brevis
Prorocentrum minimum
Symbiodinium sp.
Noctiluca scintillans
Goniomonas truncata
Chlamydomonas reinhardtii
Ostreococcus tauri
Emiliania huxleyi
Isochrysis galbana
Chrysochromulina brevifilum CAMPEP_0174754224
Chrysochromulina sp.
Reticulomyxa filosa
Thaumatomonas sp.
Monosiga brevicollis
Salpingoeca rosetta
0.6

E

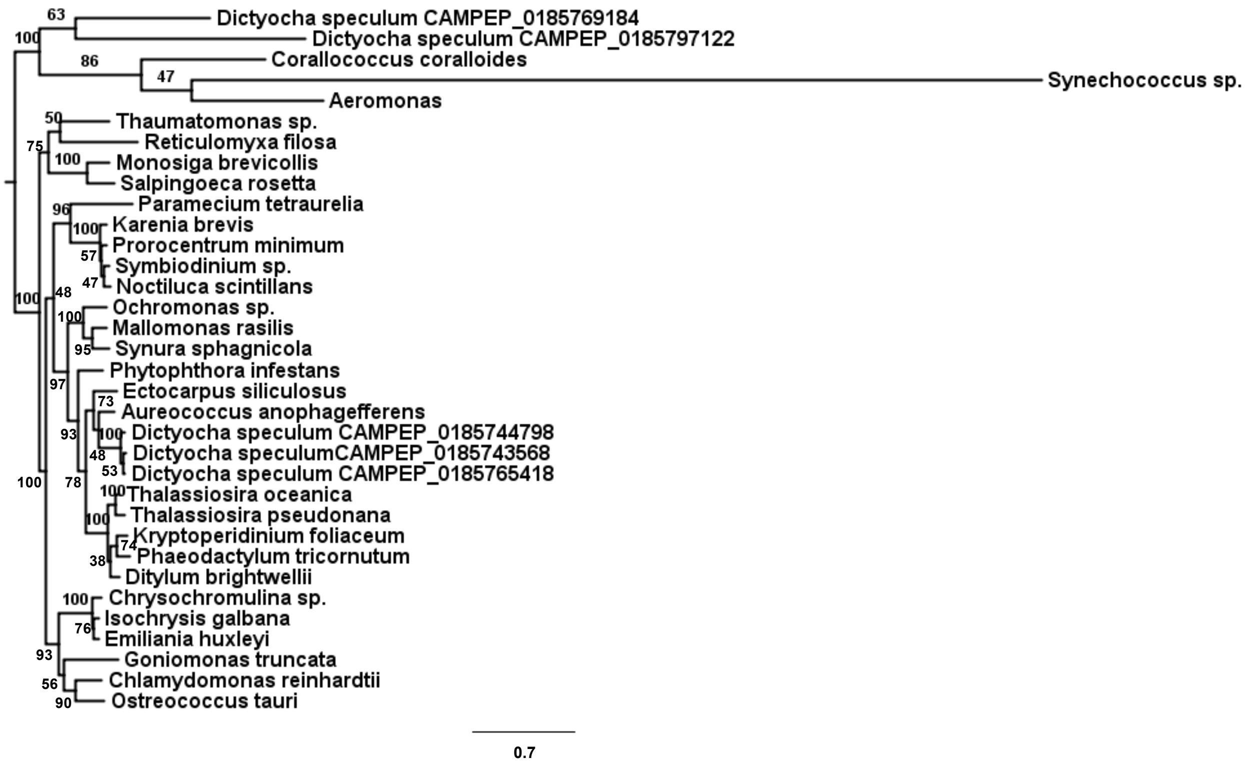
Dictyocha speculum CAMPEP_0185769184
Dictyocha speculum CAMPEP_0185797122
Corallococcus coralloides
Synechococcus sp.
Aeromonas
Thaumatomonas sp.
Reticulomyxa filosa
Monosiga brevicollis
Salpingoeca rosetta
Paramecium tetraurelia
Karenia brevis
Prorocentrum minimum
Symbiodinium sp.
Noctiluca scintillans
Ochromonas sp.
Mallomonas rasilis
Synura sphagnicola
Phytophthora infestans
Ectocarpus siliculosus
Aureococcus anophagefferens
Dictyocha speculum CAMPEP_0185744798
Dictyocha speculumCAMPEP_0185743568
Dictyocha speculum CAMPEP_0185765418
Thalassiosira oceanica
Thalassiosira pseudonana
Kryptoperidinium foliaceum
Phaeodactylum tricornutum
Ditylum brightwellii
Chrysochromulina sp.
Isochrysis galbana
Emiliania huxleyi
Goniomonas truncata
Chlamydomonas reinhardtii
Ostreococcus tauri
0.7

F

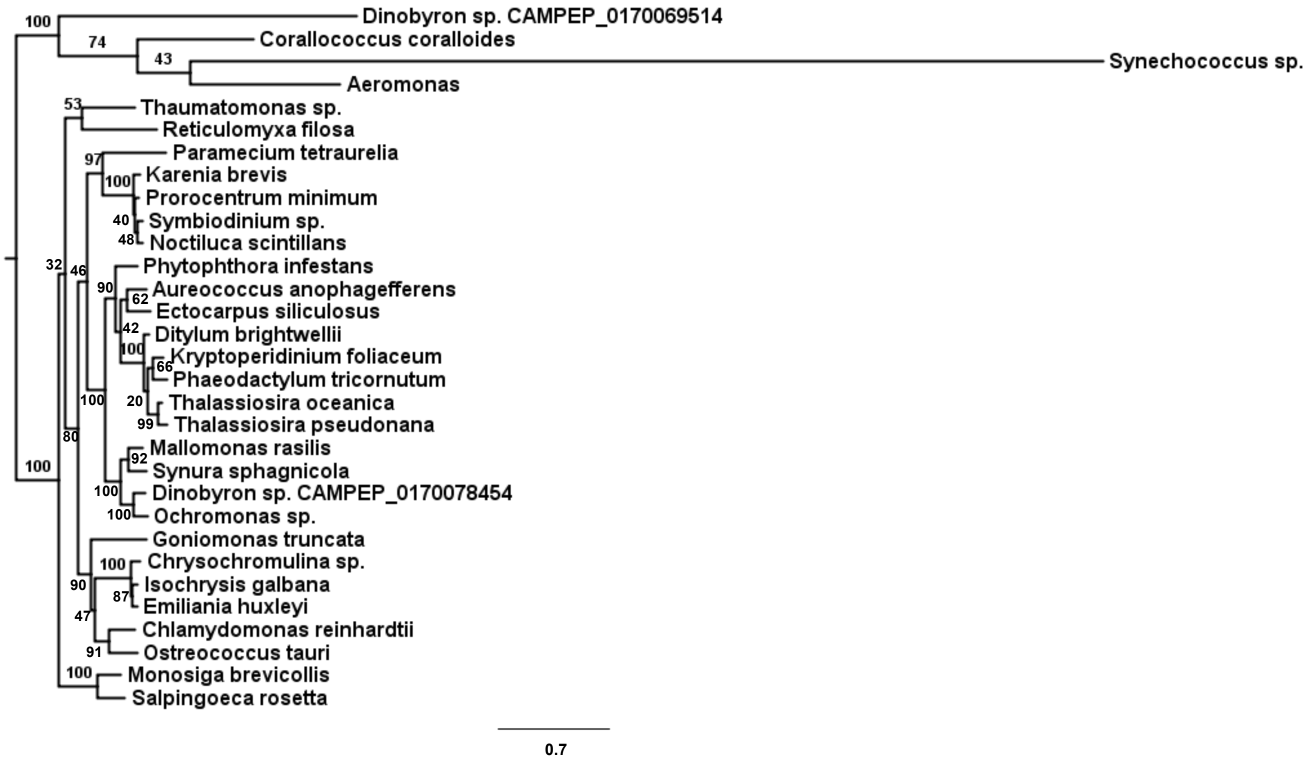
Dinobyron sp. CAMPEP_0170069514
Corallococcus coralloides
Synechococcus sp.
Aeromonas
Thaumatomonas sp.
Reticulomyxa filosa
Paramecium tetraurelia
Karenia brevis
Prorocentrum minimum
Symbiodinium sp.
Noctiluca scintillans
Phytophthora infestans
Aureococcus anophagefferens
Ectocarpus siliculosus
Ditylum brightwellii
Kryptoperidinium foliaceum
Phaeodactylum tricornutum
Thalassiosira oceanica
Thalassiosira pseudonana
Mallomonas rasilis
Synura sphagnicola
Dinobyron sp. CAMPEP_0170078454
Ochromonas sp.
Goniomonas truncata
Chrysochromulina sp.
Isochrysis galbana
Emiliania huxleyi
Chlamydomonas reinhardtii
Ostreococcus tauri
Monosiga brevicollis
Salpingoeca rosetta
0.7

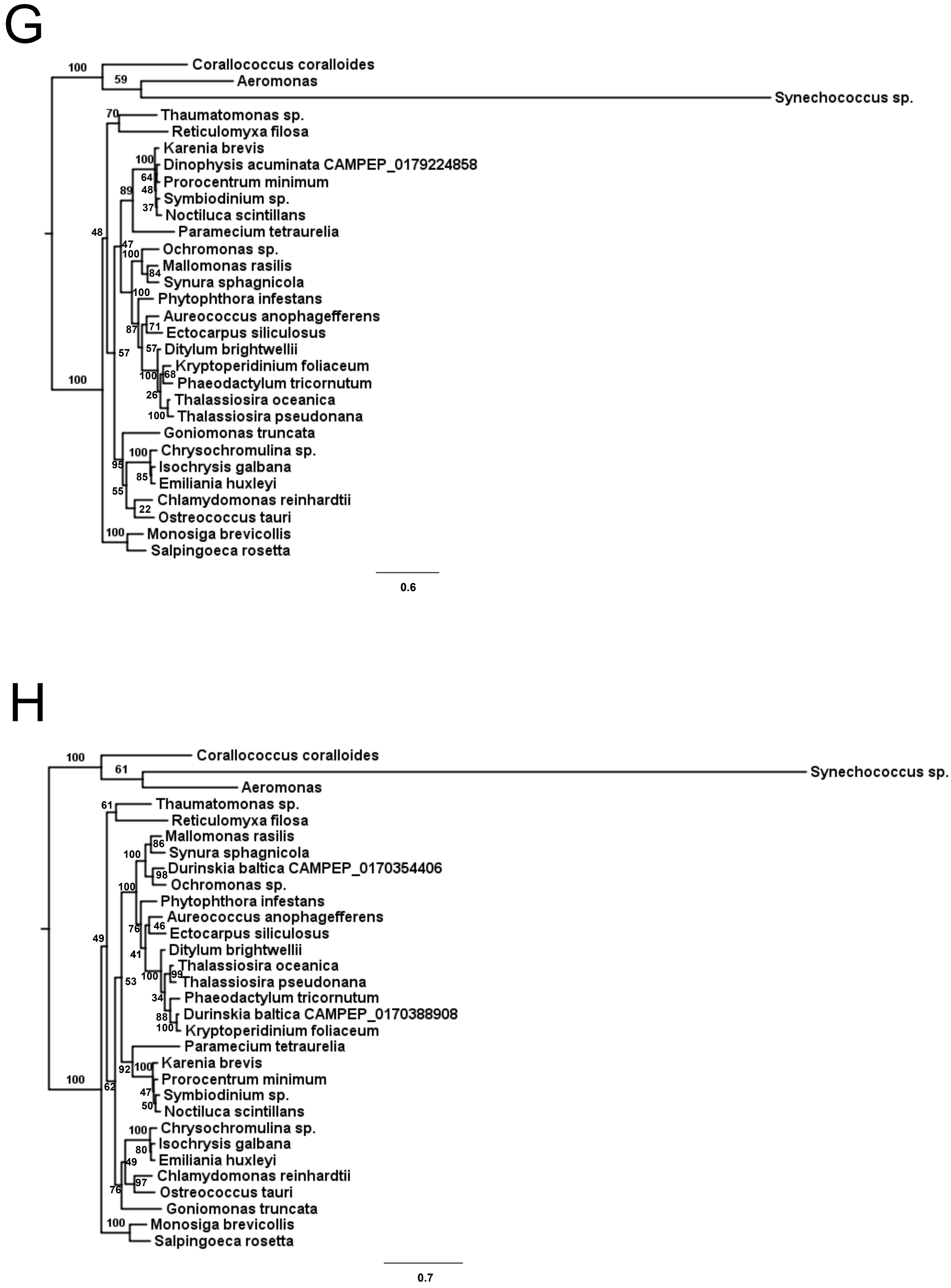
G
Corallococcus coralloides
Aeromonas
Synechococcus sp.
Thaumatomonas sp.
Reticulomyxa filosa
Karenia brevis
Dinophysis acuminata CAMPEP_0179224858
Prorocentrum minimum
Symbiodinium sp.
Noctiluca scintillans
Paramecium tetraurelia
Ochromonas sp.
Mallomonas rasilis
Synura sphagnicola
Phytophthora infestans
Aureococcus anophagefferens
Ectocarpus siliculosus
Ditylum brightwellii
Kryptoperidinium foliaceum
Phaeodactylum tricornutum
Thalassiosira oceanica
Thalassiosira pseudonana
Goniomonas truncata
Chrysochromulina sp.
Isochrysis galbana
Emiliania huxleyi
Chlamydomonas reinhardtii
Ostreococcus tauri
Monosiga brevicollis
Salpingoeca rosetta
0.6
H
Corallococcus coralloides
Synechococcus sp.
Aeromonas
Thaumatomonas sp.
Reticulomyxa filosa
Mallomonas rasilis
Synura sphagnicola
Durinskia baltica CAMPEP_0170354406
Ochromonas sp.
Phytophthora infestans
Aureococcus anophagefferens
Ectocarpus siliculosus
Ditylum brightwellii
Thalassiosira oceanica
Thalassiosira pseudonana
Phaeodactylum tricornutum
Durinskia baltica CAMPEP_0170388908
Kryptoperidinium foliaceum
Paramecium tetraurelia
Karenia brevis
Prorocentrum minimum
Symbiodinium sp.
Noctiluca scintillans
Chrysochromulina sp.
Isochrysis galbana
Emiliania huxleyi
Chlamydomonas reinhardtii
Ostreococcus tauri
Goniomonas truncata
Monosiga brevicollis
Salpingoeca rosetta
0.7

I

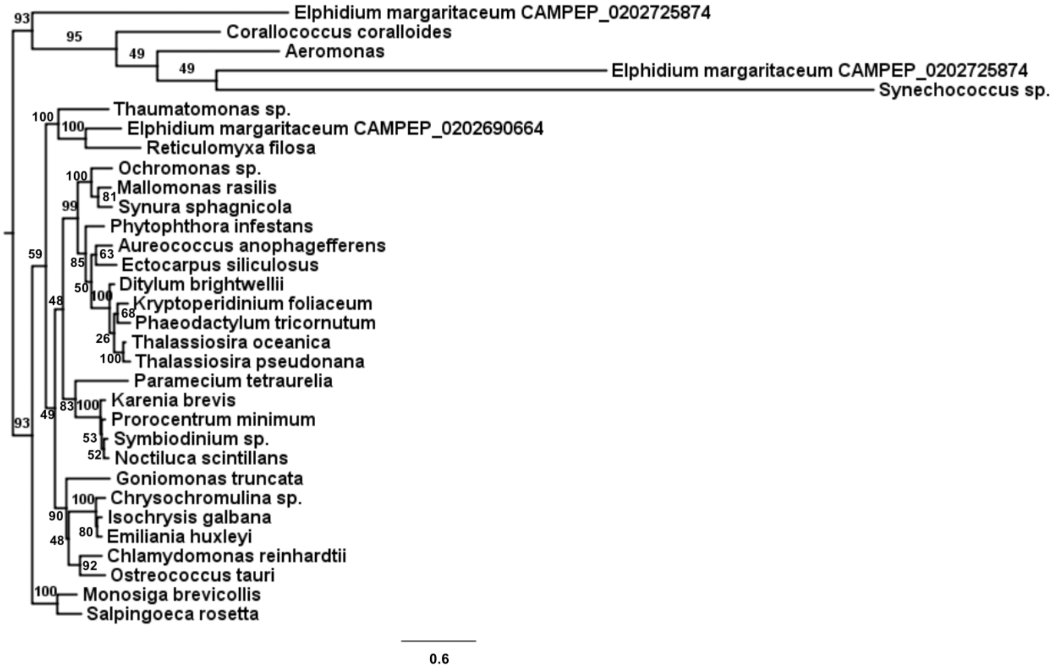
Elphidium margaritaceum CAMPEP_0202725874
Corallococcus coralloides
Aeromonas
Elphidium margaritaceum CAMPEP_0202725874
Synechococcus sp.
Thaumatomonas sp.
Elphidium margaritaceum CAMPEP_0202690664
Reticulomyxa filosa
Ochromonas sp.
Mallomonas rasilis
Synura sphagnicola
Phytophthora infestans
Aureococcus anophagefferens
Ectocarpus siliculosus
Ditylum brightwellii
Kryptoperidinium foliaceum
Phaeodactylum tricornutum
Thalassiosira oceanica
Thalassiosira pseudonana
Paramecium tetraurelia
Karenia brevis
Prorocentrum minimum
Symbiodinium sp.
Noctiluca scintillans
Goniomonas truncata
Chrysochromulina sp.
Isochrysis galbana
Emiliania huxleyi
Chlamydomonas reinhardtii
Ostreococcus tauri
Monosiga brevicollis
Salpingoeca rosetta
0.6

J

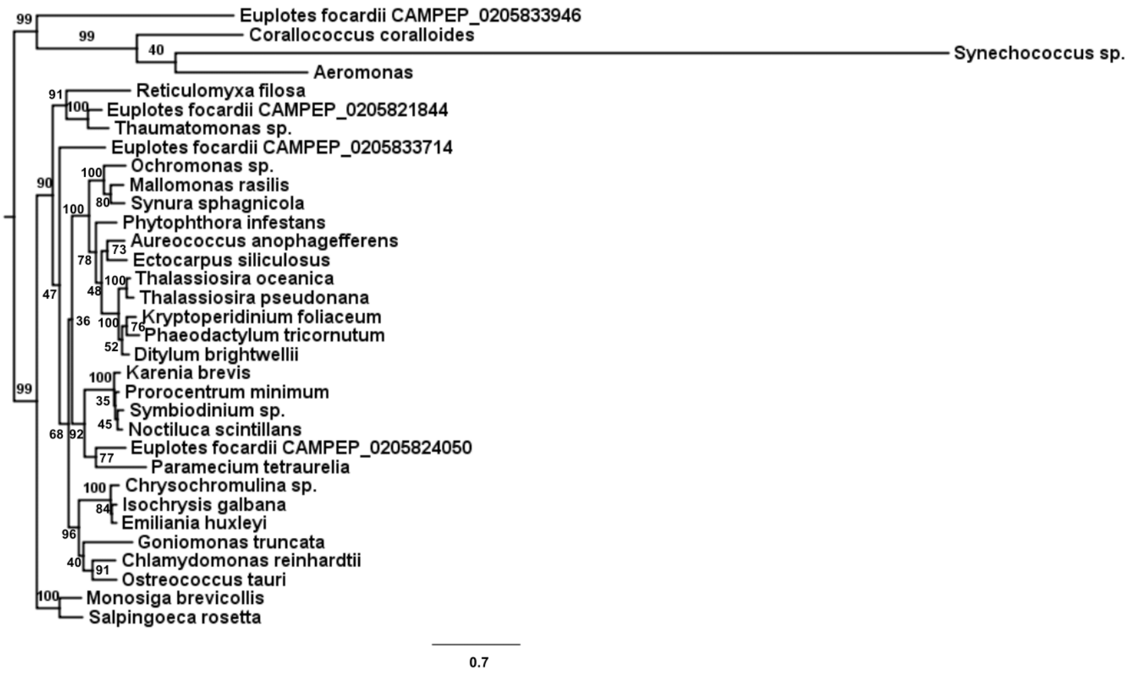
Euplotes focardii CAMPEP_0205833946
Corallococcus coralloides
Synechococcus sp.
Aeromonas
Reticulomyxa filosa
Euplotes focardii CAMPEP_0205821844
Thaumatomonas sp.
Euplotes focardii CAMPEP_0205833714
Ochromonas sp.
Mallomonas rasilis
Synura sphagnicola
Phytophthora infestans
Aureococcus anophagefferens
Ectocarpus siliculosus
Thalassiosira oceanica
Thalassiosira pseudonana
Kryptoperidinium foliaceum
Phaeodactylum tricornutum
Ditylum brightwellii
Karenia brevis
Prorocentrum minimum
Symbiodinium sp.
Noctiluca scintillans
Euplotes focardii CAMPEP_0205824050
Paramecium tetraurelia
Chrysochromulina sp.
Isochrysis galbana
Emiliania huxleyi
Goniomonas truncata
Chlamydomonas reinhardtii
Ostreococcus tauri
Monosiga brevicollis
Salpingoeca rosetta
0.7

K

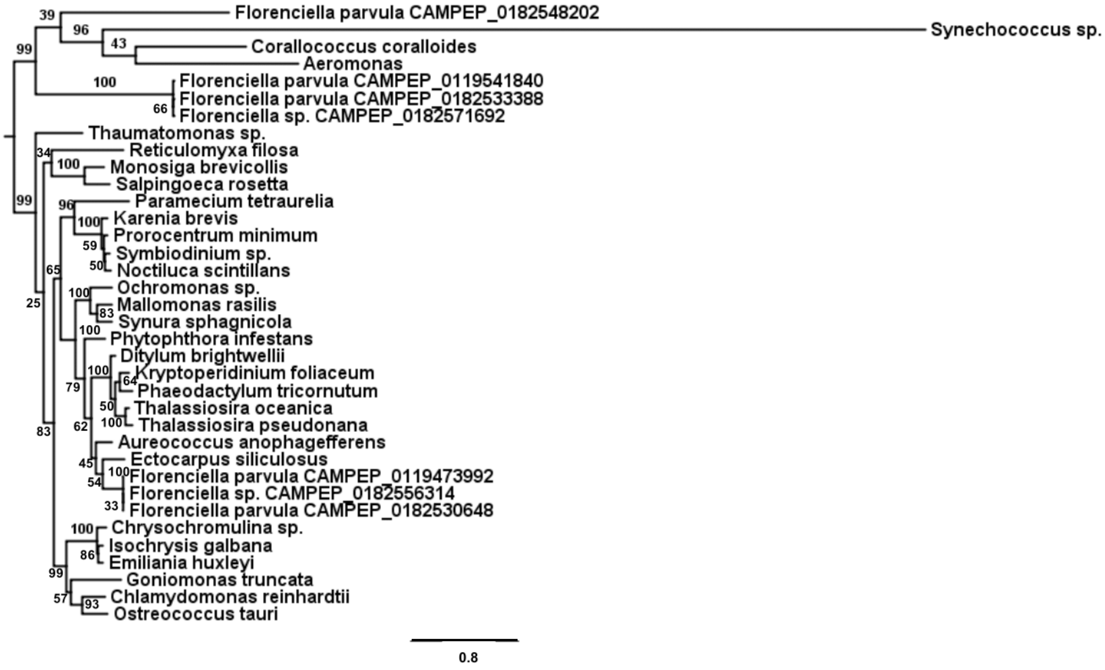
Florenciella parvula CAMPEP_0182548202
Synechococcus sp.
Corallococcus coralloides
Aeromonas
Florenciella parvula CAMPEP_0119541840
Florenciella parvula CAMPEP_0182533388
Florenciella sp. CAMPEP_0182571692
Thaumatomonas sp.
Reticulomyxa filosa
Monosiga brevicollis
Salpingoeca rosetta
Paramecium tetraurelia
Karenia brevis
Prorocentrum minimum
Symbiodinium sp.
Noctiluca scintillans
Ochromonas sp.
Mallomonas rasilis
Synura sphagnicola
Phytophthora infestans
Ditylum brightwellii
Kryptoperidinium foliaceum
Phaeodactylum tricornutum
Thalassiosira oceanica
Thalassiosira pseudonana
Aureococcus anophagefferens
Ectocarpus siliculosus
Florenciella parvula CAMPEP_0119473992
Florenciella sp. CAMPEP_0182556314
Florenciella parvula CAMPEP_0182530648
Chrysochromulina sp.
Isochrysis galbana
Emiliania huxleyi
Goniomonas truncata
Chlamydomonas reinhardtii
Ostreococcus tauri
0.8

L

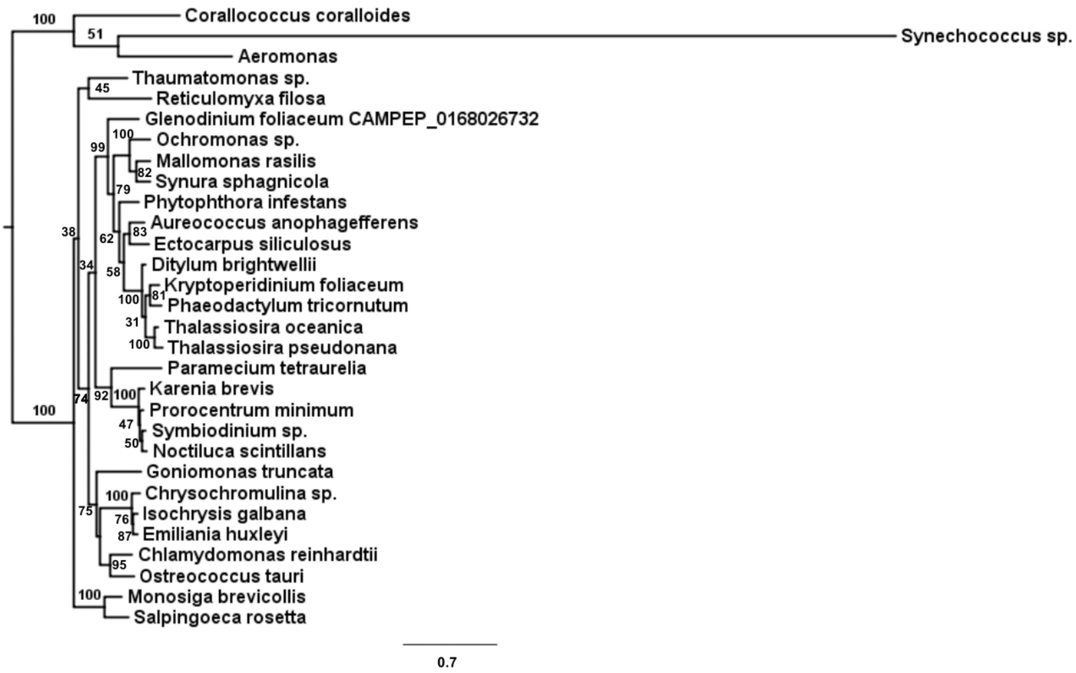
Corallococcus coralloides
Synechococcus sp.
Aeromonas
Thaumatomonas sp.
Reticulomyxa filosa
Glenodinium foliaceum CAMPEP_0168026732
Ochromonas sp.
Mallomonas rasilis
Synura sphagnicola
Phytophthora infestans
Aureococcus anophagefferens
Ectocarpus siliculosus
Ditylum brightwellii
Kryptoperidinium foliaceum
Phaeodactylum tricornutum
Thalassiosira oceanica
Thalassiosira pseudonana
Paramecium tetraurelia
Karenia brevis
Prorocentrum minimum
Symbiodinium sp.
Noctiluca scintillans
Goniomonas truncata
Chrysochromulina sp.
Isochrysis galbana
Emiliania huxleyi
Chlamydomonas reinhardtii
Ostreococcus tauri
Monosiga brevicollis
Salpingoeca rosetta
0.7

M

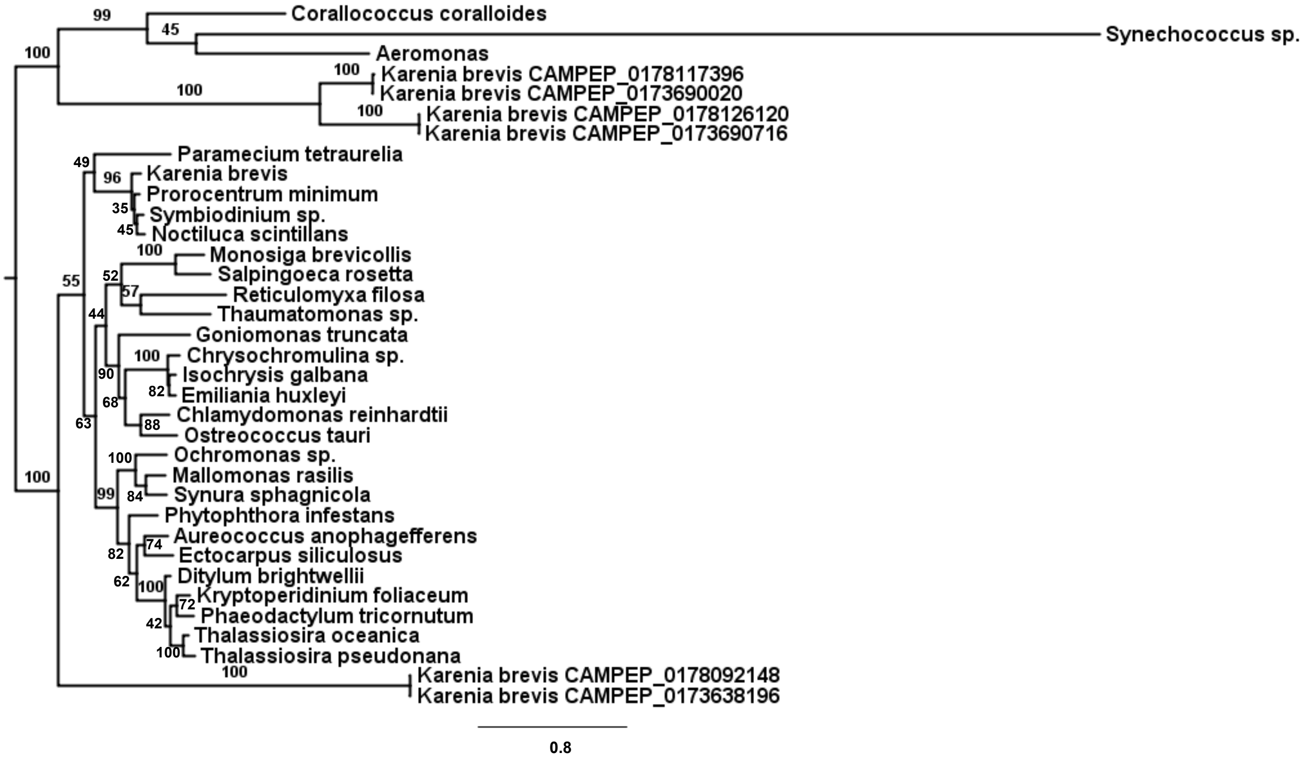
Corallococcus coralloides
Synechococcus sp.
Aeromonas
Karenia brevis CAMPEP_0178117396
Karenia brevis CAMPEP_0173690020
Karenia brevis CAMPEP_0178126120
Karenia brevis CAMPEP_0173690716
Paramecium tetraurelia
Karenia brevis
Prorocentrum minimum
Symbiodinium sp.
Noctiluca scintillans
Monosiga brevicollis
Salpingoeca rosetta
Reticulomyxa filosa
Thaumatomonas sp.
Goniomonas truncata
Chrysochromulina sp.
Isochrysis galbana
Emiliania huxleyi
Chlamydomonas reinhardtii
Ostreococcus tauri
Ochromonas sp.
Mallomonas rasilis
Synura sphagnicola
Phytophthora infestans
Aureococcus anophagefferens
Ectocarpus siliculosus
Ditylum brightwellii
Kryptoperidinium foliaceum
Phaeodactylum tricornutum
Thalassiosira oceanica
Thalassiosira pseudonana
Karenia brevis CAMPEP_0178092148
Karenia brevis CAMPEP_0173638196
0.8

N

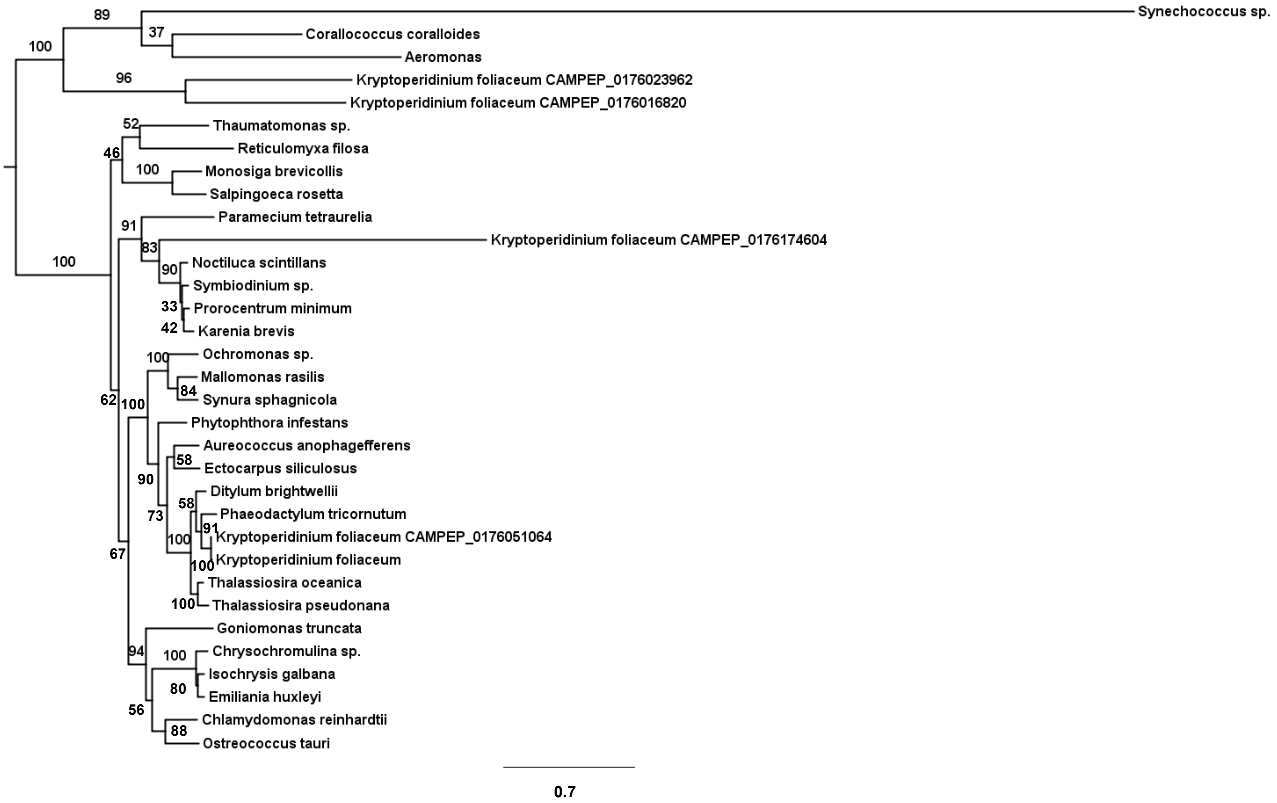
Synechococcus sp.
Corallococcus coralloides
Aeromonas
Kryptoperidinium foliaceum CAMPEP_0176023962
Kryptoperidinium foliaceum CAMPEP_0176016820
Thaumatomonas sp.
Reticulomyxa filosa
Monosiga brevicollis
Salpingoeca rosetta
Paramecium tetraurelia
Kryptoperidinium foliaceum CAMPEP_0176174604
Noctiluca scintillans
Symbiodinium sp.
Prorocentrum minimum
Karenia brevis
Ochromonas sp.
Mallomonas rasilis
Synura sphagnicola
Phytophthora infestans
Aureococcus anophagefferens
Ectocarpus siliculosus
Ditylum brightwellii
Phaeodactylum tricornutum
Kryptoperidinium foliaceum CAMPEP_0176051064
Kryptoperidinium foliaceum
Thalassiosira oceanica
Thalassiosira pseudonana
Goniomonas truncata
Chrysochromulina sp.
Isochrysis galbana
Emiliania huxleyi
Chlamydomonas reinhardtii
Ostreococcus tauri
0.7

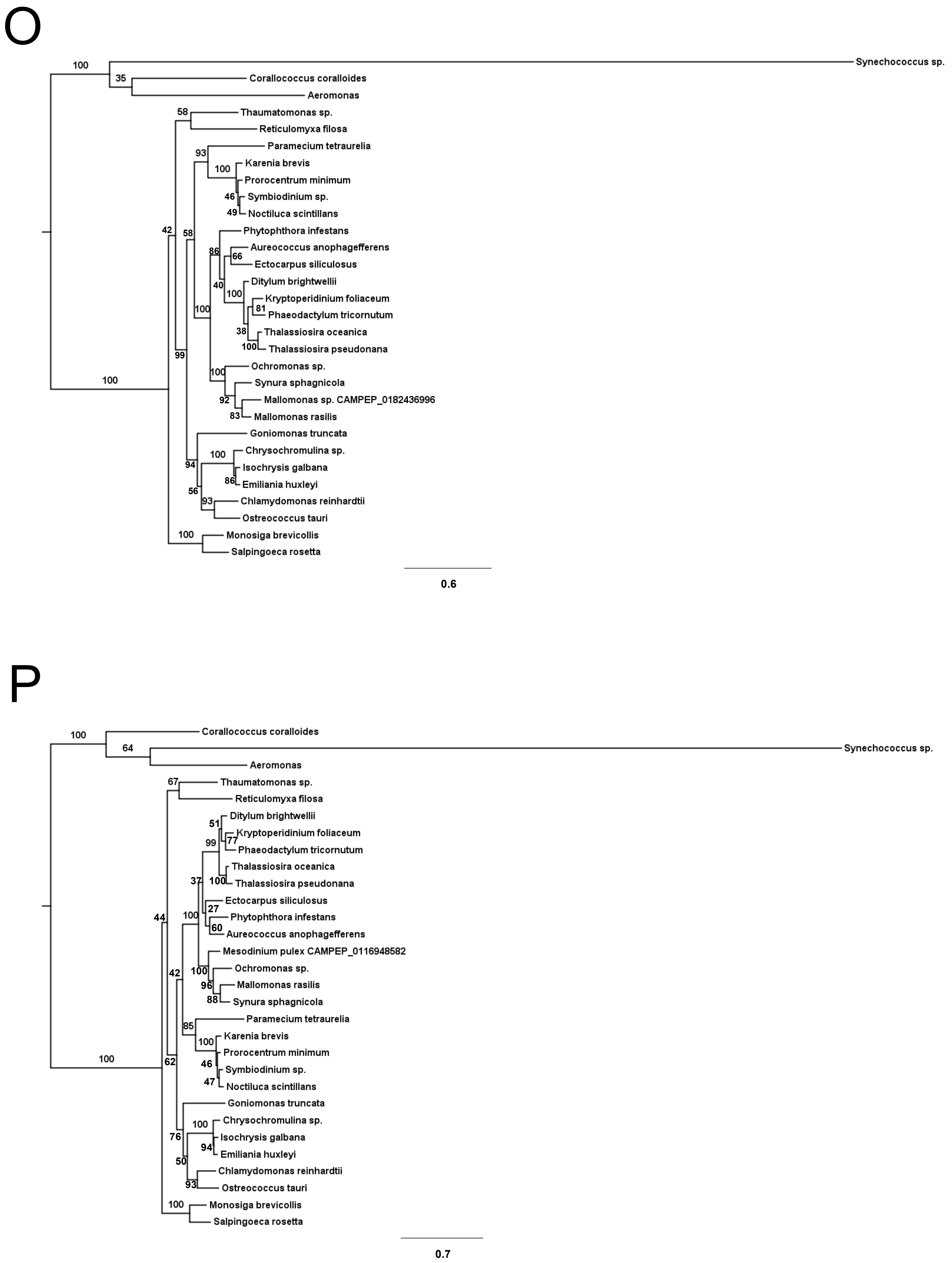
O
Synechococcus sp.
Corallococcus coralloides
Aeromonas
Thaumatomonas sp.
Reticulomyxa filosa
Paramecium tetraurelia
Karenia brevis
Prorocentrum minimum
Symbiodinium sp.
Noctiluca scintillans
Phytophthora infestans
Aureococcus anophagefferens
Ectocarpus siliculosus
Ditylum brightwellii
Kryptoperidinium foliaceum
Phaeodactylum tricornutum
Thalassiosira oceanica
Thalassiosira pseudonana
Ochromonas sp.
Synura sphagnicola
Mallomonas sp. CAMPEP_0182436996
Mallomonas rasilis
Goniomonas truncata
Chrysochromulina sp.
Isochrysis galbana
Emiliania huxleyi
Chlamydomonas reinhardtii
Ostreococcus tauri
Monosiga brevicollis
Salpingoeca rosetta
0.6
P
Corallococcus coralloides
Synechococcus sp.
Aeromonas
Thaumatomonas sp.
Reticulomyxa filosa
Ditylum brightwellii
Kryptoperidinium foliaceum
Phaeodactylum tricornutum
Thalassiosira oceanica
Thalassiosira pseudonana
Ectocarpus siliculosus
Phytophthora infestans
Aureococcus anophagefferens
Mesodinium pulex CAMPEP_0116948582
Ochromonas sp.
Mallomonas rasilis
Synura sphagnicola
Paramecium tetraurelia
Karenia brevis
Prorocentrum minimum
Symbiodinium sp.
Noctiluca scintillans
Goniomonas truncata
Chrysochromulina sp.
Isochrysis galbana
Emiliania huxleyi
Chlamydomonas reinhardtii
Ostreococcus tauri
Monosiga brevicollis
Salpingoeca rosetta
0.7

Q

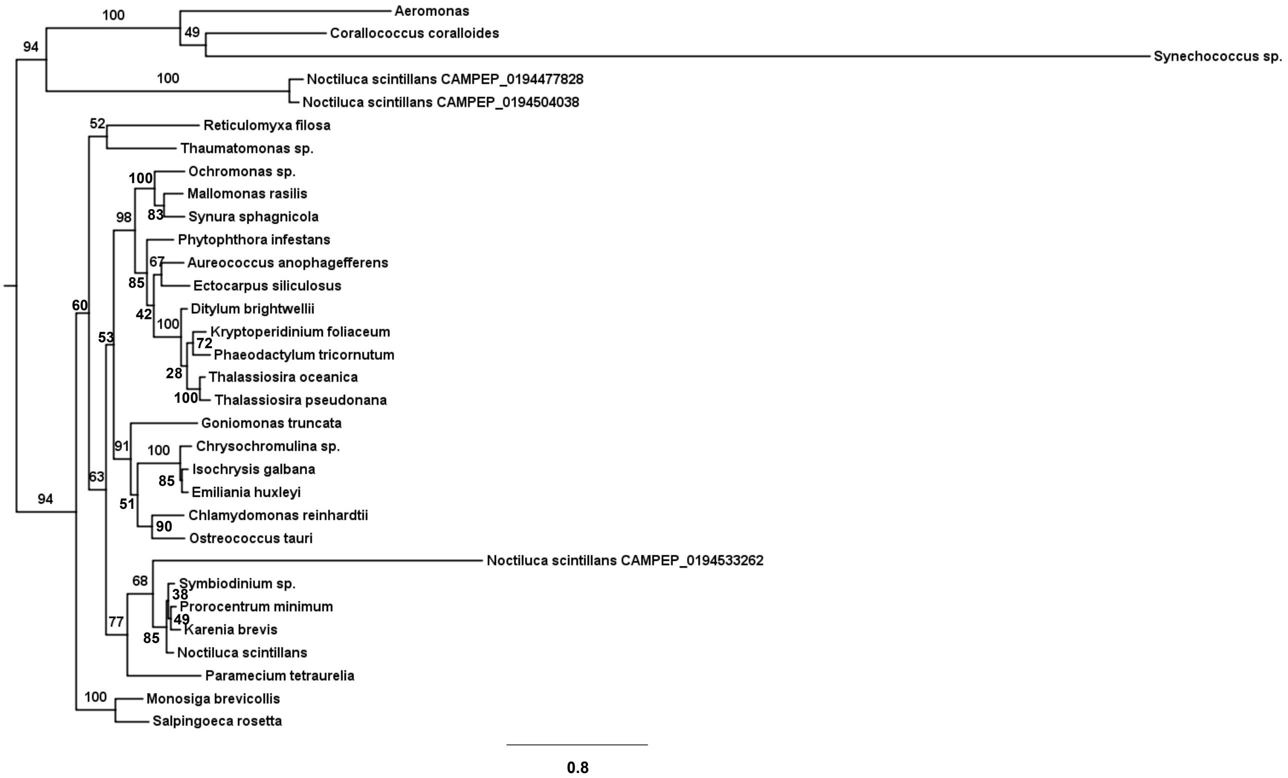
Aeromonas
Corallococcus coralloides
Synechococcus sp.
Noctiluca scintillans CAMPEP_0194477828
Noctiluca scintillans CAMPEP_0194504038
Reticulomyxa filosa
Thaumatomonas sp.
Ochromonas sp.
Mallomonas rasilis
Synura sphagnicola
Phytophthora infestans
Aureococcus anophagefferens
Ectocarpus siliculosus
Ditylum brightwellii
Kryptoperidinium foliaceum
Phaeodactylum tricornutum
Thalassiosira oceanica
Thalassiosira pseudonana
Goniomonas truncata
Chrysochromulina sp.
Isochrysis galbana
Emiliania huxleyi
Chlamydomonas reinhardtii
Ostreococcus tauri
Noctiluca scintillans CAMPEP_0194533262
Symbiodinium sp.
Prorocentrum minimum
Karenia brevis
Noctiluca scintillans
Paramecium tetraurelia
Monosiga brevicollis
Salpingoeca rosetta
0.8

R

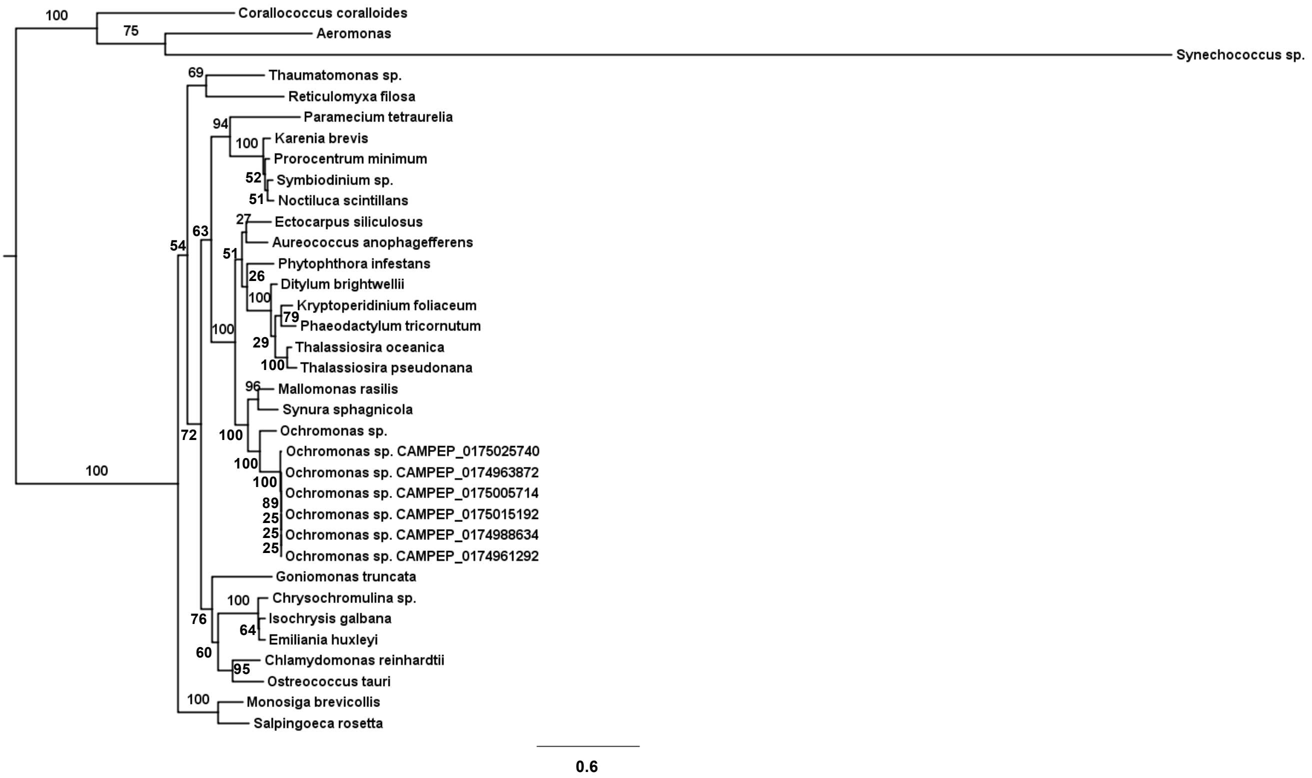
Corallococcus coralloides
Aeromonas
Synechococcus sp.
Thaumatomonas sp.
Reticulomyxa filosa
Paramecium tetraurelia
Karenia brevis
Prorocentrum minimum
Symbiodinium sp.
Noctiluca scintillans
Ectocarpus siliculosus
Aureococcus anophagefferens
Phytophthora infestans
Ditylum brightwellii
Kryptoperidinium foliaceum
Phaeodactylum tricornutum
Thalassiosira oceanica
Thalassiosira pseudonana
Mallomonas rasilis
Synura sphagnicola
Ochromonas sp.
Ochromonas sp. CAMPEP_0175025740
Ochromonas sp. CAMPEP_0174963872
Ochromonas sp. CAMPEP_0175005714
Ochromonas sp. CAMPEP_0175015192
Ochromonas sp. CAMPEP_0174988634
Ochromonas sp. CAMPEP_0174961292
Goniomonas truncata
Chrysochromulina sp.
Isochrysis galbana
Emiliania huxleyi
Chlamydomonas reinhardtii
Ostreococcus tauri
Monosiga brevicollis
Salpingoeca rosetta
0.6

S

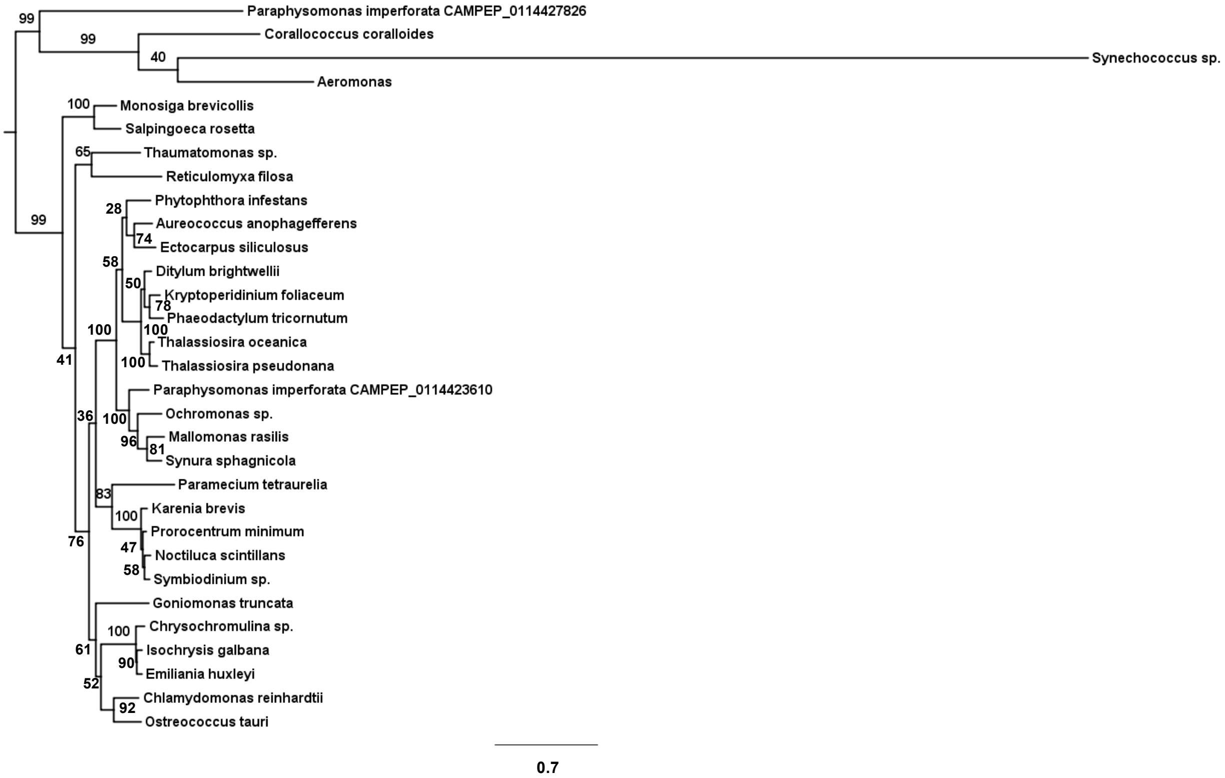
Paraphysomonas imperforata CAMPEP_0114427826
Corallococcus coralloides
Synechococcus sp.
Aeromonas
Monosiga brevicollis
Salpingoeca rosetta
Thaumatomonas sp.
Reticulomyxa filosa
Phytophthora infestans
Aureococcus anophagefferens
Ectocarpus siliculosus
Ditylum brightwellii
Kryptoperidinium foliaceum
Phaeodactylum tricornutum
Thalassiosira oceanica
Thalassiosira pseudonana
Paraphysomonas imperforata CAMPEP_0114423610
Ochromonas sp.
Mallomonas rasilis
Synura sphagnicola
Paramecium tetraurelia
Karenia brevis
Prorocentrum minimum
Noctiluca scintillans
Symbiodinium sp.
Goniomonas truncata
Chrysochromulina sp.
Isochrysis galbana
Emiliania huxleyi
Chlamydomonas reinhardtii
Ostreococcus tauri
0.7

T

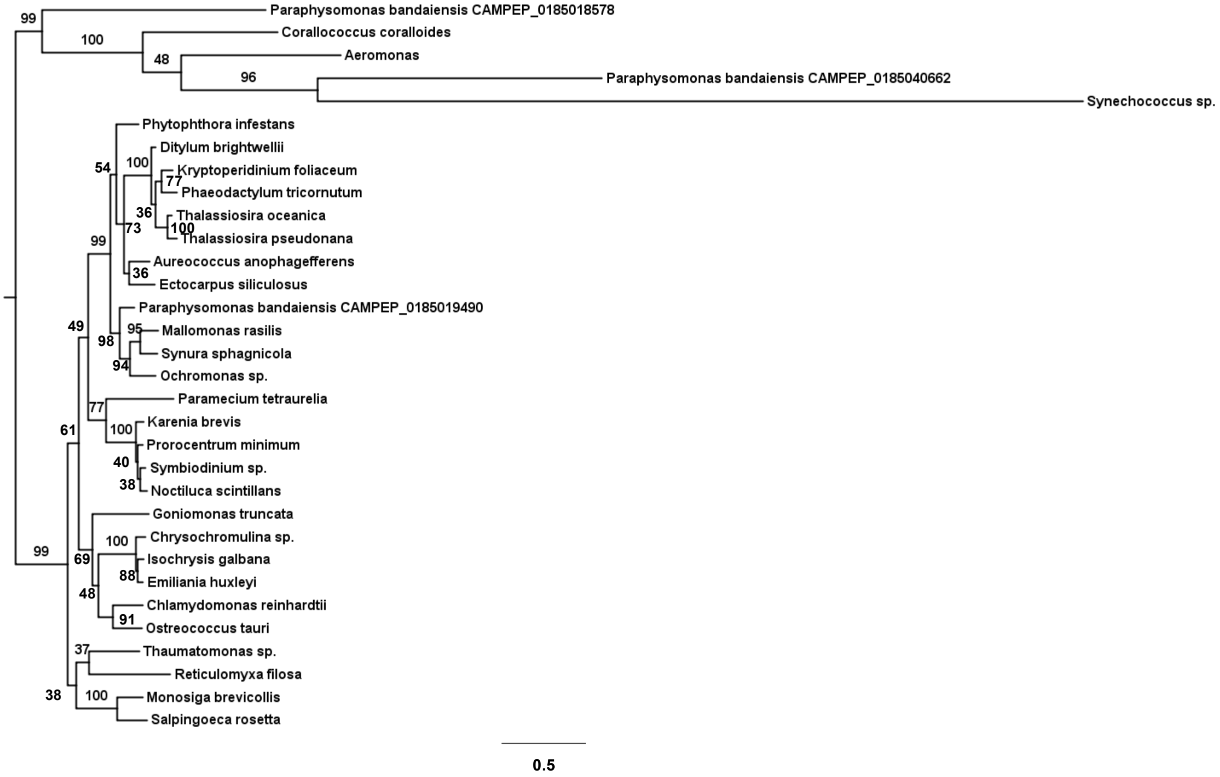
Paraphysomonas bandaiensis CAMPEP_0185018578
Corallococcus coralloides
Aeromonas
Paraphysomonas bandaiensis CAMPEP_0185040662
Synechococcus sp.
Phytophthora infestans
Ditylum brightwellii
Kryptoperidinium foliaceum
Phaeodactylum tricornutum
Thalassiosira oceanica
Thalassiosira pseudonana
Aureococcus anophagefferens
Ectocarpus siliculosus
Paraphysomonas bandaiensis CAMPEP_0185019490
Mallomonas rasilis
Synura sphagnicola
Ochromonas sp.
Paramecium tetraurelia
Karenia brevis
Prorocentrum minimum
Symbiodinium sp.
Noctiluca scintillans
Goniomonas truncata
Chrysochromulina sp.
Isochrysis galbana
Emiliania huxleyi
Chlamydomonas reinhardtii
Ostreococcus tauri
Thaumatomonas sp.
Reticulomyxa filosa
Monosiga brevicollis
Salpingoeca rosetta
0.5

U

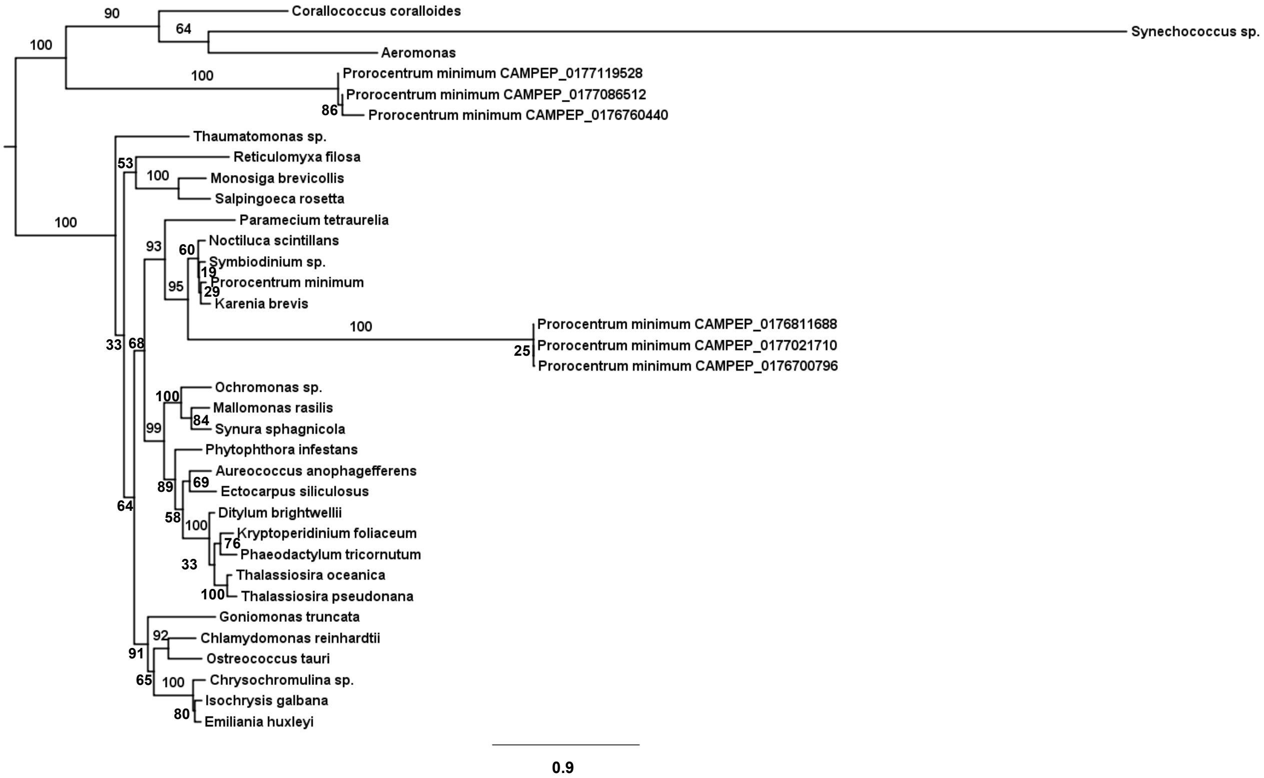
Corallococcus coralloides
Synechococcus sp.
Aeromonas
Prorocentrum minimum CAMPEP_0177119528
Prorocentrum minimum CAMPEP_0177086512
Prorocentrum minimum CAMPEP_0176760440
Thaumatomonas sp.
Reticulomyxa filosa
Monosiga brevicollis
Salpingoeca rosetta
Paramecium tetraurelia
Noctiluca scintillans
Symbiodinium sp.
Prorocentrum minimum
Karenia brevis
Prorocentrum minimum CAMPEP_0176811688
Prorocentrum minimum CAMPEP_0177021710
Prorocentrum minimum CAMPEP_0176700796
Ochromonas sp.
Mallomonas rasilis
Synura sphagnicola
Phytophthora infestans
Aureococcus anophagefferens
Ectocarpus siliculosus
Ditylum brightwellii
Kryptoperidinium foliaceum
Phaeodactylum tricornutum
Thalassiosira oceanica
Thalassiosira pseudonana
Goniomonas truncata
Chlamydomonas reinhardtii
Ostreococcus tauri
Chrysochromulina sp.
Isochrysis galbana
Emiliania huxleyi
0.9

V

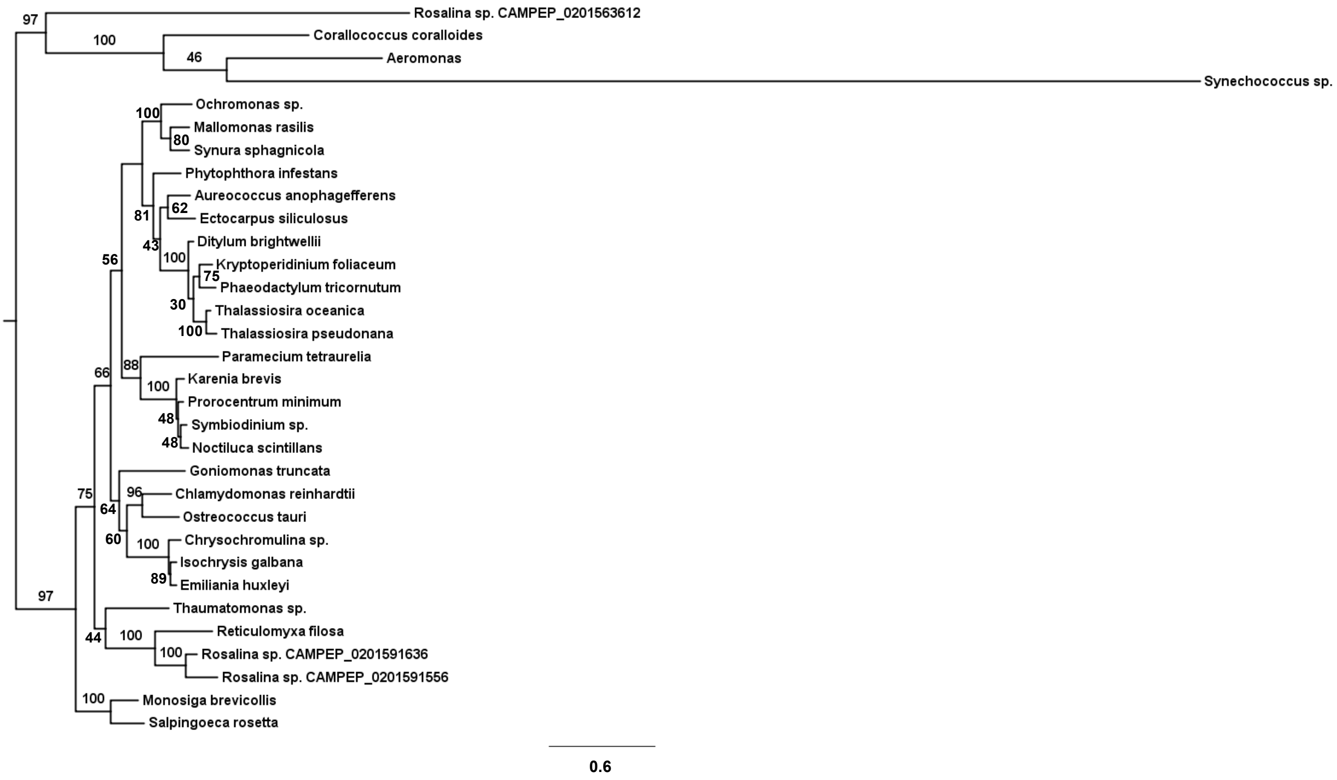
Rosalina sp. CAMPEP_0201563612
Corallococcus coralloides
Aeromonas
Synechococcus sp.
Ochromonas sp.
Mallomonas rasilis
Synura sphagnicola
Phytophthora infestans
Aureococcus anophagefferens
Ectocarpus siliculosus
Ditylum brightwellii
Kryptoperidinium foliaceum
Phaeodactylum tricornutum
Thalassiosira oceanica
Thalassiosira pseudonana
Paramecium tetraurelia
Karenia brevis
Prorocentrum minimum
Symbiodinium sp.
Noctiluca scintillans
Goniomonas truncata
Chlamydomonas reinhardtii
Ostreococcus tauri
Chrysochromulina sp.
Isochrysis galbana
Emiliania huxleyi
Thaumatomonas sp.
Reticulomyxa filosa
Rosalina sp. CAMPEP_0201591636
Rosalina sp. CAMPEP_0201591556
Monosiga brevicollis
Salpingoeca rosetta
0.6

W

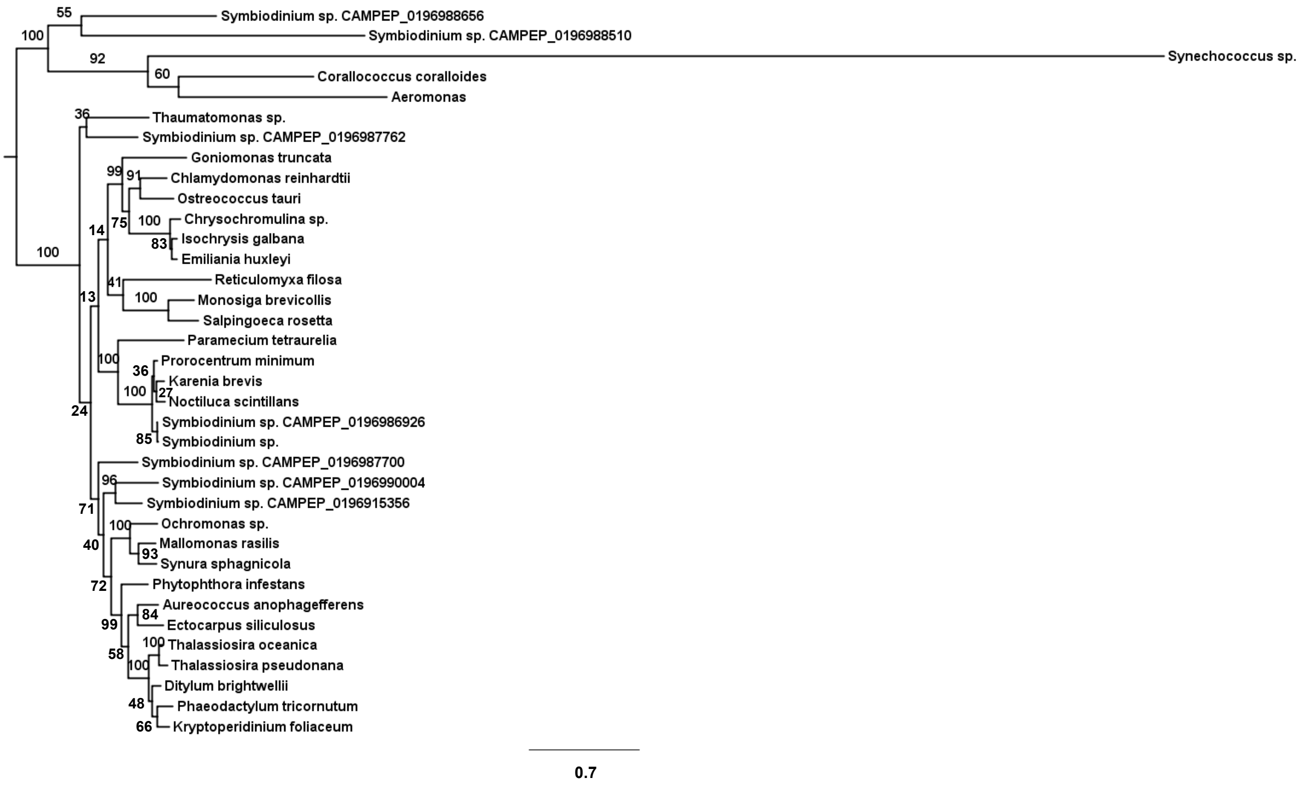
Symbiodinium sp. CAMPEP_0196988656
Symbiodinium sp. CAMPEP_0196988510
Synechococcus sp.
Corallococcus coralloides
Aeromonas
Thaumatomonas sp.
Symbiodinium sp. CAMPEP_0196987762
Goniomonas truncata
Chlamydomonas reinhardtii
Ostreococcus tauri
Chrysochromulina sp.
Isochrysis galbana
Emiliania huxleyi
Reticulomyxa filosa
Monosiga brevicollis
Salpingoeca rosetta
Paramecium tetraurelia
Prorocentrum minimum
Karenia brevis
Noctiluca scintillans
Symbiodinium sp. CAMPEP_0196986926
Symbiodinium sp.
Symbiodinium sp. CAMPEP_0196987700
Symbiodinium sp. CAMPEP_0196990004
Symbiodinium sp. CAMPEP_0196915356
Ochromonas sp.
Mallomonas rasilis
Synura sphagnicola
Phytophthora infestans
Aureococcus anophagefferens
Ectocarpus siliculosus
Thalassiosira oceanica
Thalassiosira pseudonana
Ditylum brightwellii
Phaeodactylum tricornutum
Kryptoperidinium foliaceum
0.7

X

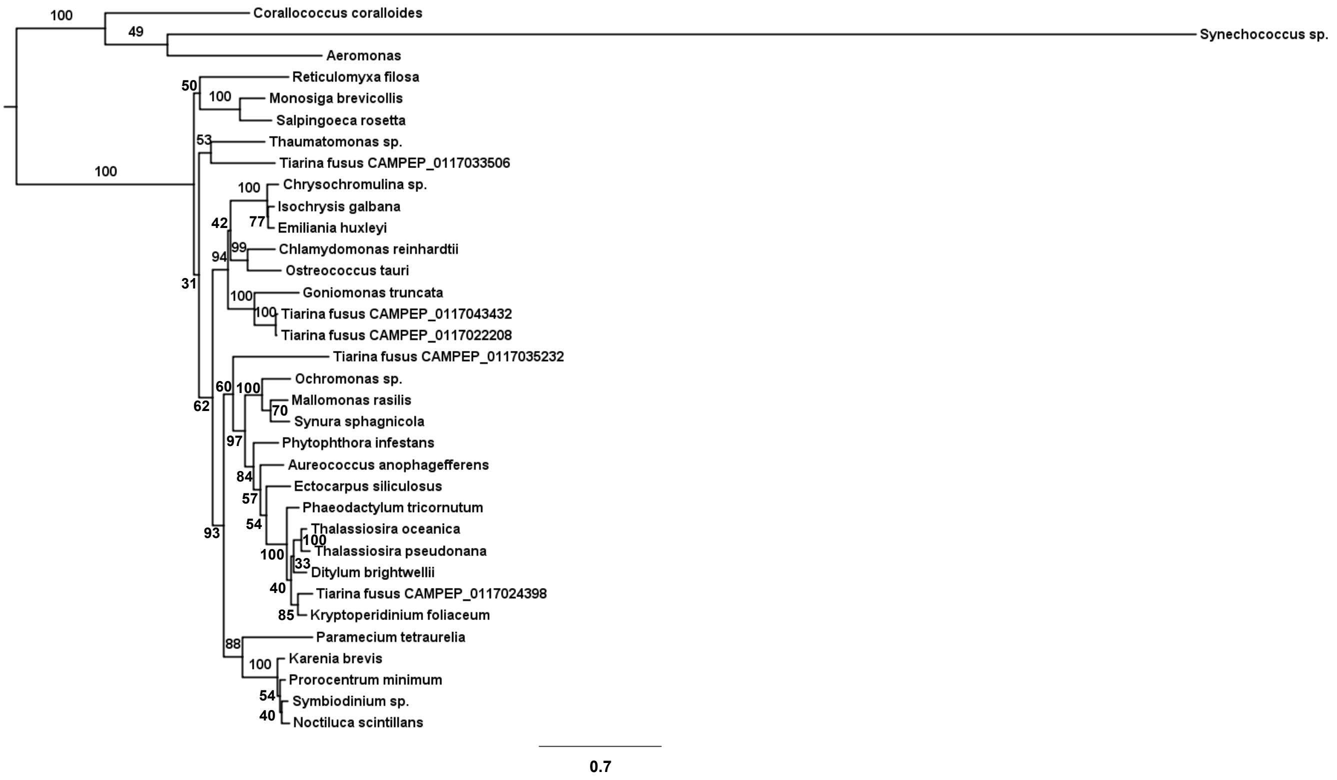
Corallococcus coralloides
Synechococcus sp.
Aeromonas
Reticulomyxa filosa
Monosiga brevicollis
Salpingoeca rosetta
Thaumatomonas sp.
Tiarina fusus CAMPEP_0117033506
Chrysochromulina sp.
Isochrysis galbana
Emiliania huxleyi
Chlamydomonas reinhardtii
Ostreococcus tauri
Goniomonas truncata
Tiarina fusus CAMPEP_0117043432
Tiarina fusus CAMPEP_0117022208
Tiarina fusus CAMPEP_0117035232
Ochromonas sp.
Mallomonas rasilis
Synura sphagnicola
Phytophthora infestans
Aureococcus anophagefferens
Ectocarpus siliculosus
Phaeodactylum tricornutum
Thalassiosira oceanica
Thalassiosira pseudonana
Ditylum brightwellii
Tiarina fusus CAMPEP_0117024398
Kryptoperidinium foliaceum
Paramecium tetraurelia
Karenia brevis
Prorocentrum minimum
Symbiodinium sp.
Noctiluca scintillans
0.7

Y

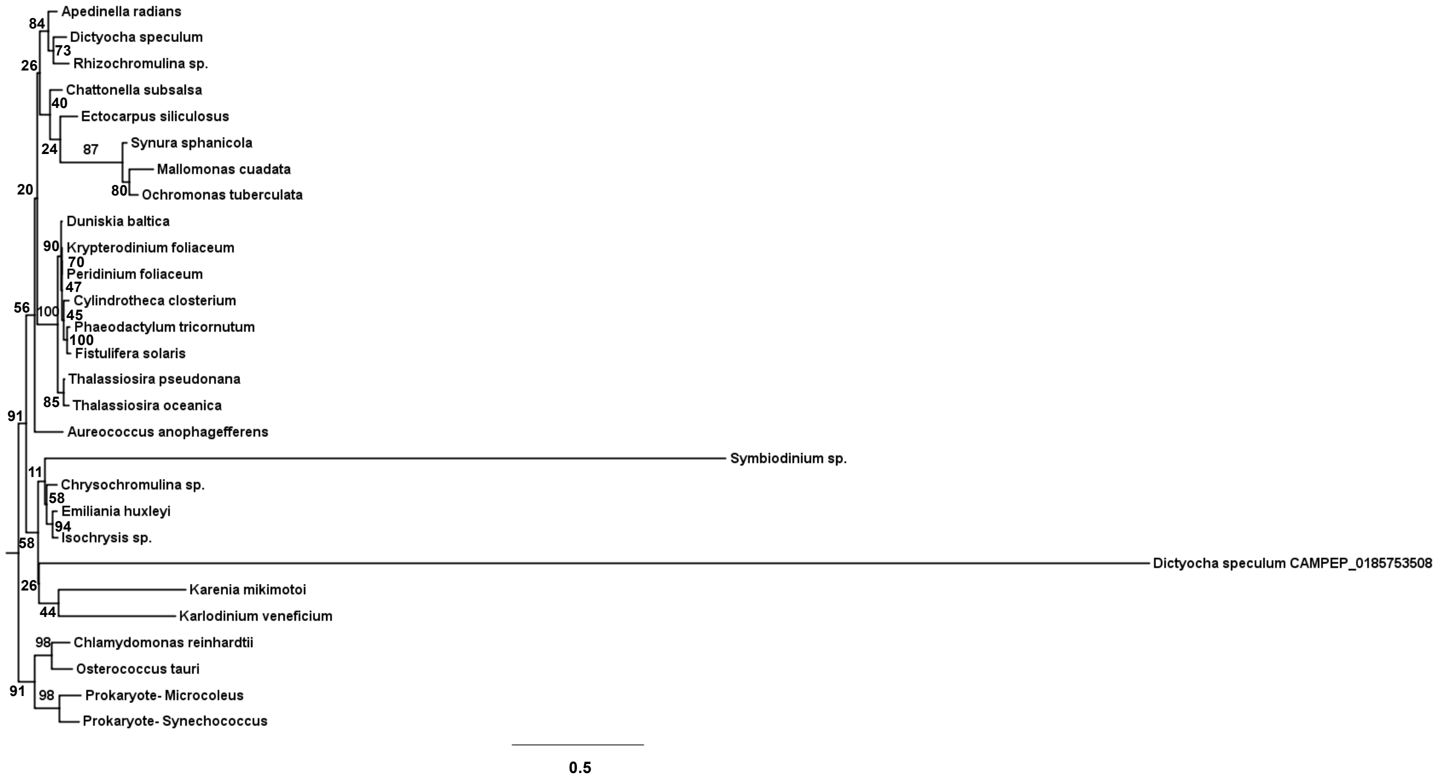

Supplementary Figure 2. Contamination testing using Hsp90 and psaA phylogenies. Sequences used are listed in Supplementary Table 3. Contamination was presumed if MMETSP sequences branch as with eukaryotes but distant to taxonomically relevant sequences, and as sister to stramenopiles. Trees were generated by PhyML maximum likelihood analysis, using the LG+G model. Numbers at nodes are a percentage of 100 bootstrap replicates. Scale bar indicates average number of amino acid substitutions per site. (A) Hsp90 Reference Framework tree, (B) Hsp90 *Ammonia* sp. (C) Hsp90 *Ceratium fusus* (D) Hsp90 *Chrysochromulina* sp. (E) Hsp90 *Dictyocha speculum* (F) Hsp90 *Dinobryon* sp. (G) Hsp90 *Dinophysis acuminata* (H) Hsp90 *Durinskia baltica* (I) Hsp90 *Elphidium margaritaceum* (J) Hsp90 *Euplotes focardii* (K) Hsp90 *Florenciella parvula* (L) Hsp90 *Glenodinium foliaceum* (M) Hsp90 *Karenia brevis* (N) Hsp90 *Kryptoperidinium foliaceum* (O) Hsp90 *Mallomonas* sp. (P) Hsp90 *Mesodinium pulex* (Q) Hsp90 *Noctiluca scintillans* (R) Hsp90 *Ochromonas* sp. (S) *Paraphysomonas bandaiensis* (T) Hsp90 *Paraphysomonas imperforata* (U) Hsp90 *Prorocentrum minimum* (V) Hsp90 *Rosalina* sp. (W) Hsp90 *Symbiodinium* sp. (X) Hsp90 *Tiarina fusus* (Y) psaA *Dictyocha speculum*
