# Supplementary material for: The Evolution of Silicon Transport in Eukaryotes
Source: Mol Biol Evol. 2016 Oct 11;33(12):3226–48. doi: 10.1093/molbev/msw209 (PMC5100055; doi:10.1093/molbev/msw209)
Supplement: Supplementary Data [file supp_msw209_suppl_data.zip › Supplementary_Figures_4-5-6.pdf]

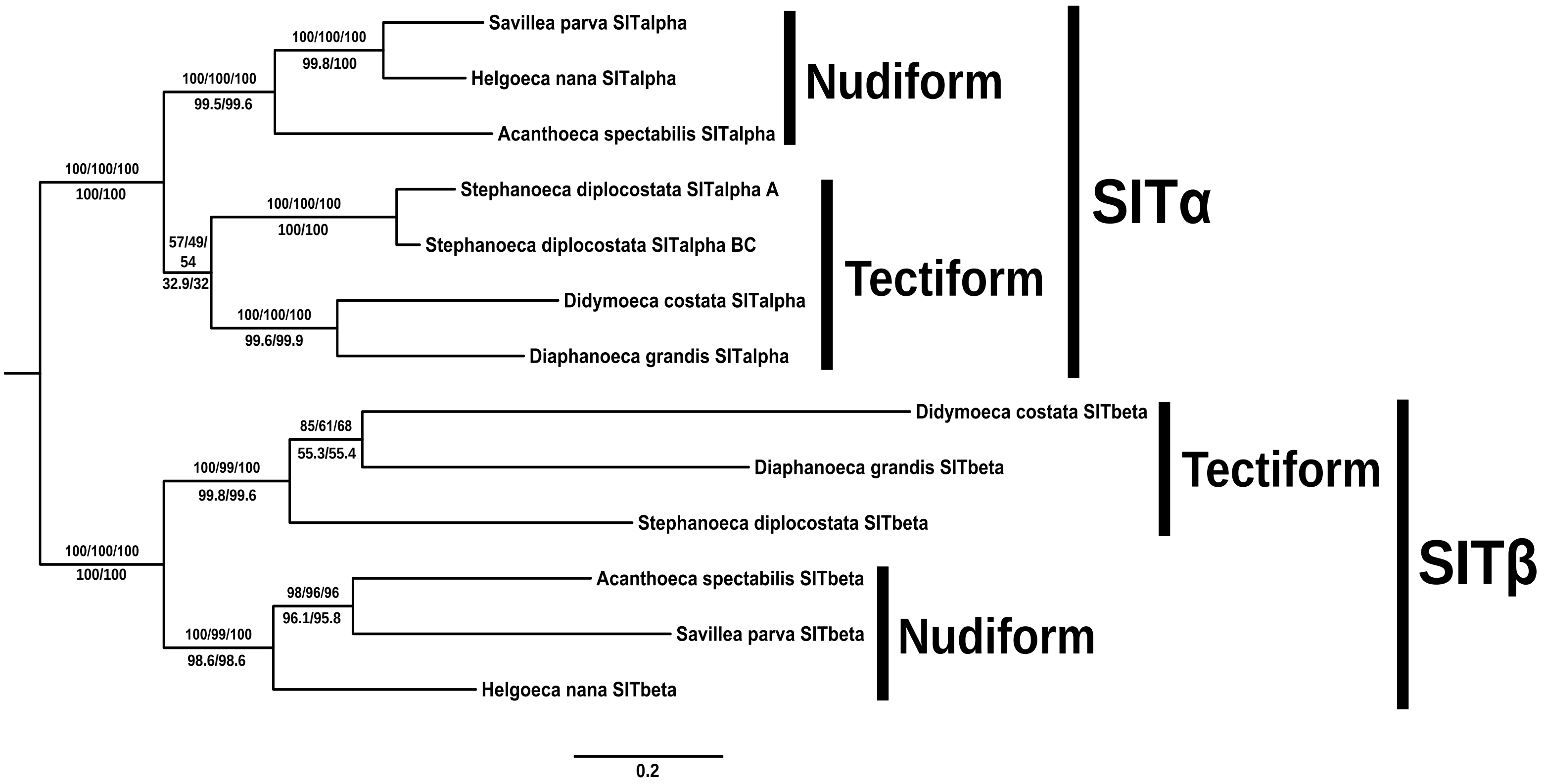

Supplementary Figure 4. The choanoflagellate SIT phylogeny. Phylogenetic analysis demonstrates that loricate choanoflagellate SITs are divided into two distinct clades, SITα and SITβ, and that the branching order reflects the species level subdivision into nudiform and tectiform families . The tree was produced usingPhyML, RaxML and IQ-TREE maximum likelihood analysis with the LG+G+F model from an alignment of 519 amino acid residues. Numbers above nodes are a percentage of 100 standard bootstrap or 1000 ultrafast bootstrap replicates in the format RaxML/PhyML/IQ-TREE value. Numbers below nodes indicate percentage SH-aLRT support in the format PhyML/IQ-Tree value. Scale bar indicates average number of amino acid substitutions per site. Bayesian MCMC analysis with MrBayes produced a tree with identical topology and all posterior probabilities values=1.

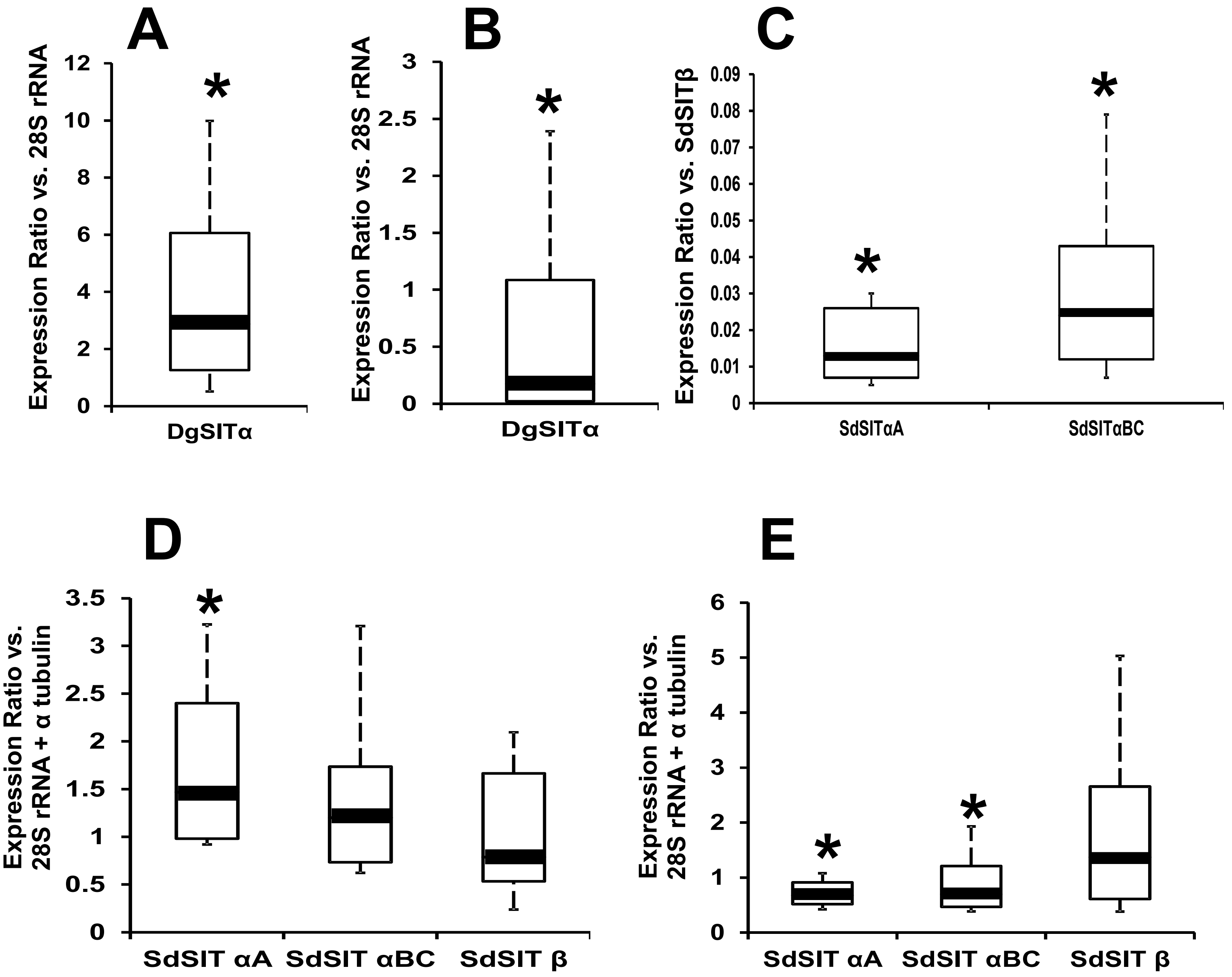

Supplementary Figure 5. Relative quantification of choanoflagellate SIT expression by RT-qPCR. (A) DgSITα expression is significantly upregulated in low Si versus control Si. (B) DgSITα expression is significantly downregulated in high Si versus control Si. (C) SdSITαA and SdSITαBC have significantly greater expression than SdSITβ. (D) Only SdSITαA expression is upregulated in low Si treatment versus control Si. SdSITαBC and SdSITβ show no significant changes in expression. (E) Both SdSITαA and SdSITαBC expression are significantly downregulated in high Si versus control Si. SdSITβ shows no significant change in expression. For (A)-(B) and (D)-(E) expression ratios are compared to 28S rRNA and α tubulin reference genes for that species, for (C) expression ratios are compared to SdSITβ expression. For (A)-(B), DgSITβ was expressed at too low a level to be reliably measured. Boxes represent the interquartile range, black line represents the median gene expression and whiskers represent the minimum and maximum observations. Asterisks denote a significant difference in relative expression as calculated from a pair-wise fixed randomization test with 10,000 permutations. In all cases n=3 from three independent replicates.

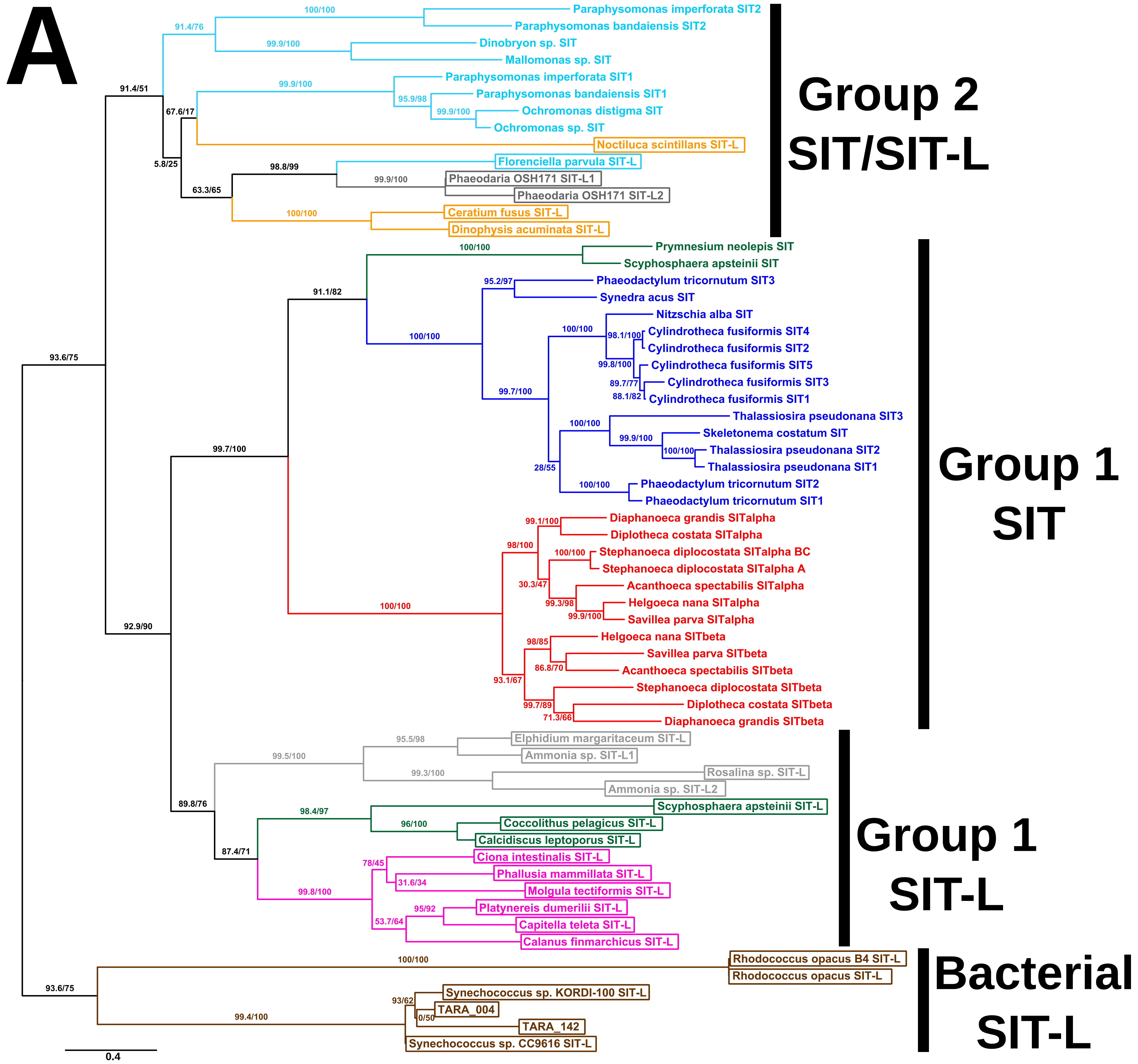
A
Group 2
SIT/SIT-L
Group 1
SIT
Group 1
SIT-L
Bacterial
SIT-L
Paraphysomonas imperforata SIT2
Paraphysomonas bandaiensis SIT2
Dinobryon sp. SIT
Mallomonas sp. SIT
Paraphysomonas imperforata SIT1
Paraphysomonas bandaiensis SIT1
Ochromonas distigma SIT
Ochromonas sp. SIT
Noctiluca scintillans SIT-L
Florenciella parvula SIT-L
Phaeodaria OSH171 SIT-L1
Phaeodaria OSH171 SIT-L2
Ceratium fusus SIT-L
Dinophysis acuminata SIT-L
Prymnesium neolepis SIT
Scyphosphaera apsteinii SIT
Phaeodactylum tricornutum SIT3
Synedra acus SIT
Nitzschia alba SIT
Cylindrotheca fusiformis SIT4
Cylindrotheca fusiformis SIT2
Cylindrotheca fusiformis SIT5
Cylindrotheca fusiformis SIT3
Cylindrotheca fusiformis SIT1
Thalassiosira pseudonana SIT3
Skeletonema costatum SIT
Thalassiosira pseudonana SIT2
Thalassiosira pseudonana SIT1
Phaeodactylum tricornutum SIT2
Phaeodactylum tricornutum SIT1
Diaphanoeca grandis SITalpha
Diplotheca costata SITalpha
Stephanoeca diplocostata SITalpha BC
Stephanoeca diplocostata SITalpha A
Acanthoeca spectabilis SITalpha
Helgoeca nana SITalpha
Savillea parva SITalpha
Helgoeca nana SITbeta
Savillea parva SITbeta
Acanthoeca spectabilis SITbeta
Stephanoeca diplocostata SITbeta
Diplotheca costata SITbeta
Diaphanoeca grandis SITbeta
Elphidium margaritaceum SIT-L
Ammonia sp. SIT-L1
Rosalina sp. SIT-L
Ammonia sp. SIT-L2
Scyphosphaera apsteinii SIT-L
Coccolithus pelagicus SIT-L
Calcidiscus leptoporus SIT-L
Ciona intestinalis SIT-L
Phallusia mammillata SIT-L
Molgula tectiformis SIT-L
Platynereis dumerilii SIT-L
Capitella teleta SIT-L
Calanus finmarchicus SIT-L
Rhodococcus opacus B4 SIT-L
Rhodococcus opacus SIT-L
Synechococcus sp. KORDI-100 SIT-L
TARA_004
TARA_142
Synechococcus sp. CC9616 SIT-L
0.4

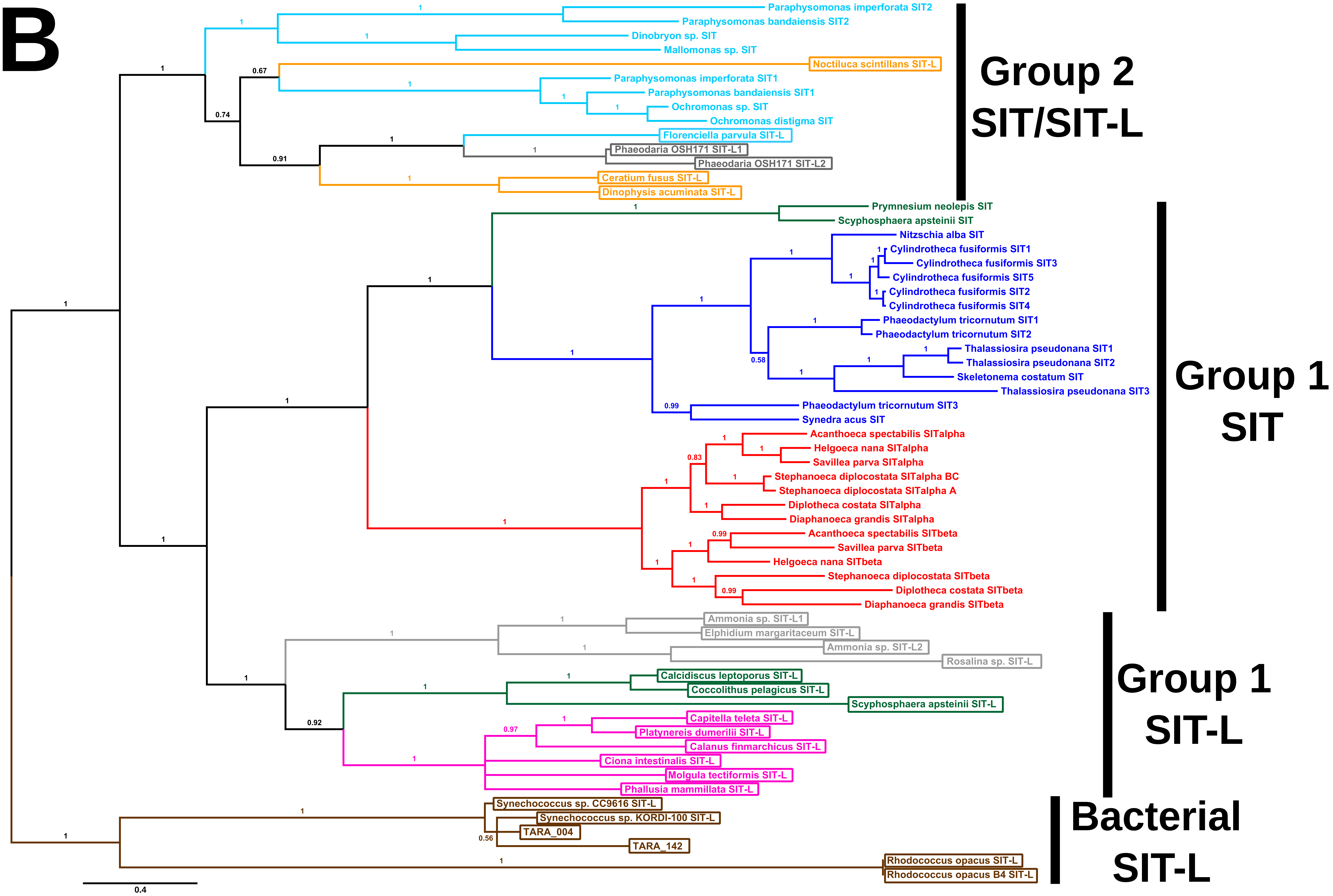
B
Paraphysomonas imperforata SIT2
Paraphysomonas bandaiensis SIT2
Dinobryon sp. SIT
Mallomonas sp. SIT
Noctiluca scintillans SIT-L
Paraphysomonas imperforata SIT1
Paraphysomonas bandaiensis SIT1
Ochromonas sp. SIT
Ochromonas distigma SIT
Florenciella parvula SIT-L
Phaeodaria OSH171 SIT-L1
Phaeodaria OSH171 SIT-L2
Ceratium fusus SIT-L
Dinophysis acuminata SIT-L
Group 2
SIT/SIT-L
Prymnesium neolepis SIT
Scyphosphaera apsteinii SIT
Nitzschia alba SIT
Cylindrotheca fusiformis SIT1
Cylindrotheca fusiformis SIT3
Cylindrotheca fusiformis SIT5
Cylindrotheca fusiformis SIT2
Cylindrotheca fusiformis SIT4
Phaeodactylum tricornutum SIT1
Phaeodactylum tricornutum SIT2
Thalassiosira pseudonana SIT1
Thalassiosira pseudonana SIT2
Skeletonema costatum SIT
Thalassiosira pseudonana SIT3
Phaeodactylum tricornutum SIT3
Synedra acus SIT
Acanthoeca spectabilis SITalpha
Helgoeca nana SITalpha
Savillea parva SITalpha
Stephanoeca diplocostata SITalpha BC
Stephanoeca diplocostata SITalpha A
Diplotheca costata SITalpha
Diaphanoeca grandis SITalpha
Acanthoeca spectabilis SITbeta
Savillea parva SITbeta
Helgoeca nana SITbeta
Stephanoeca diplocostata SITbeta
Diplotheca costata SITbeta
Diaphanoeca grandis SITbeta
Group 1
SIT
Ammonia sp. SIT-L1
Elphidium margaritaceum SIT-L
Ammonia sp. SIT-L2
Rosalina sp. SIT-L
Calcidiscus leptoporus SIT-L
Coccolithus pelagicus SIT-L
Scyphosphaera apsteinii SIT-L
Capitella teleta SIT-L
Platynereis dumerilii SIT-L
Calanus finmarchicus SIT-L
Ciona intestinalis SIT-L
Molgula tectiformis SIT-L
Phallusia mammillata SIT-L
Group 1
SIT-L
Synechococcus sp. CC9616 SIT-L
Synechococcus sp. KORDI-100 SIT-L
TARA_004
TARA_142
Rhodococcus opacus SIT-L
Rhodococcus opacus B4 SIT-L
Bacterial
SIT-L
0.4

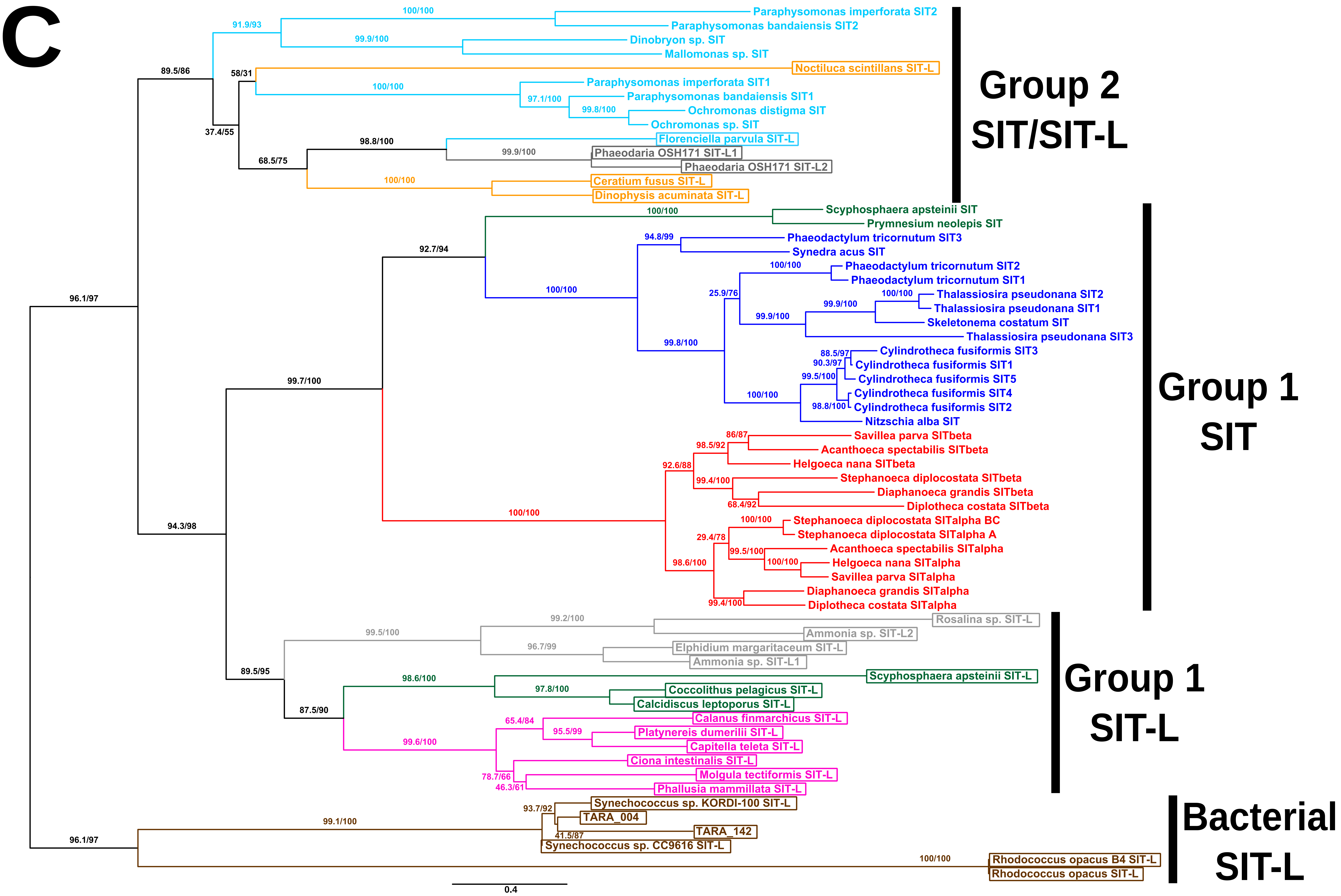
C
Paraphysomonas imperforata SIT2
Paraphysomonas bandaiensis SIT2
Dinobryon sp. SIT
Mallomonas sp. SIT
Noctiluca scintillans SIT-L
Paraphysomonas imperforata SIT1
Paraphysomonas bandaiensis SIT1
Ochromonas distigma SIT
Ochromonas sp. SIT
Florenciella parvula SIT-L
Phaeodaria OSH171 SIT-L1
Phaeodaria OSH171 SIT-L2
Ceratium fusus SIT-L
Dinophysis acuminata SIT-L
Group 2
SIT/SIT-L
Scyphosphaera apsteinii SIT
Prymnesium neolepis SIT
Phaeodactylum tricornutum SIT3
Synedra acus SIT
Phaeodactylum tricornutum SIT2
Phaeodactylum tricornutum SIT1
Thalassiosira pseudonana SIT2
Thalassiosira pseudonana SIT1
Skeletonema costatum SIT
Thalassiosira pseudonana SIT3
Cylindrotheca fusiformis SIT3
Cylindrotheca fusiformis SIT1
Cylindrotheca fusiformis SIT5
Cylindrotheca fusiformis SIT4
Cylindrotheca fusiformis SIT2
Nitzschia alba SIT
Savillea parva SITbeta
Acanthoeca spectabilis SITbeta
Helgoeca nana SITbeta
Stephanoeca diplocostata SITbeta
Diaphanoeca grandis SITbeta
Diplotheca costata SITbeta
Stephanoeca diplocostata SITalpha BC
Stephanoeca diplocostata SITalpha A
Acanthoeca spectabilis SITalpha
Helgoeca nana SITalpha
Savillea parva SITalpha
Diaphanoeca grandis SITalpha
Diplotheca costata SITalpha
Group 1
SIT
Rosalina sp. SIT-L
Ammonia sp. SIT-L2
Elphidium margaritaceum SIT-L
Ammonia sp. SIT-L1
Scyphosphaera apsteinii SIT-L
Coccolithus pelagicus SIT-L
Calcidiscus leptoporus SIT-L
Calanus finmarchicus SIT-L
Platynereis dumerilii SIT-L
Capitella teleta SIT-L
Ciona intestinalis SIT-L
Molgula tectiformis SIT-L
Phallusia mammillata SIT-L
Group 1
SIT-L
Synechococcus sp. KORDI-100 SIT-L
TARA_004
TARA_142
Synechococcus sp. CC9616 SIT-L
Rhodococcus opacus B4 SIT-L
Rhodococcus opacus SIT-L
Bacterial
SIT-L
0.4

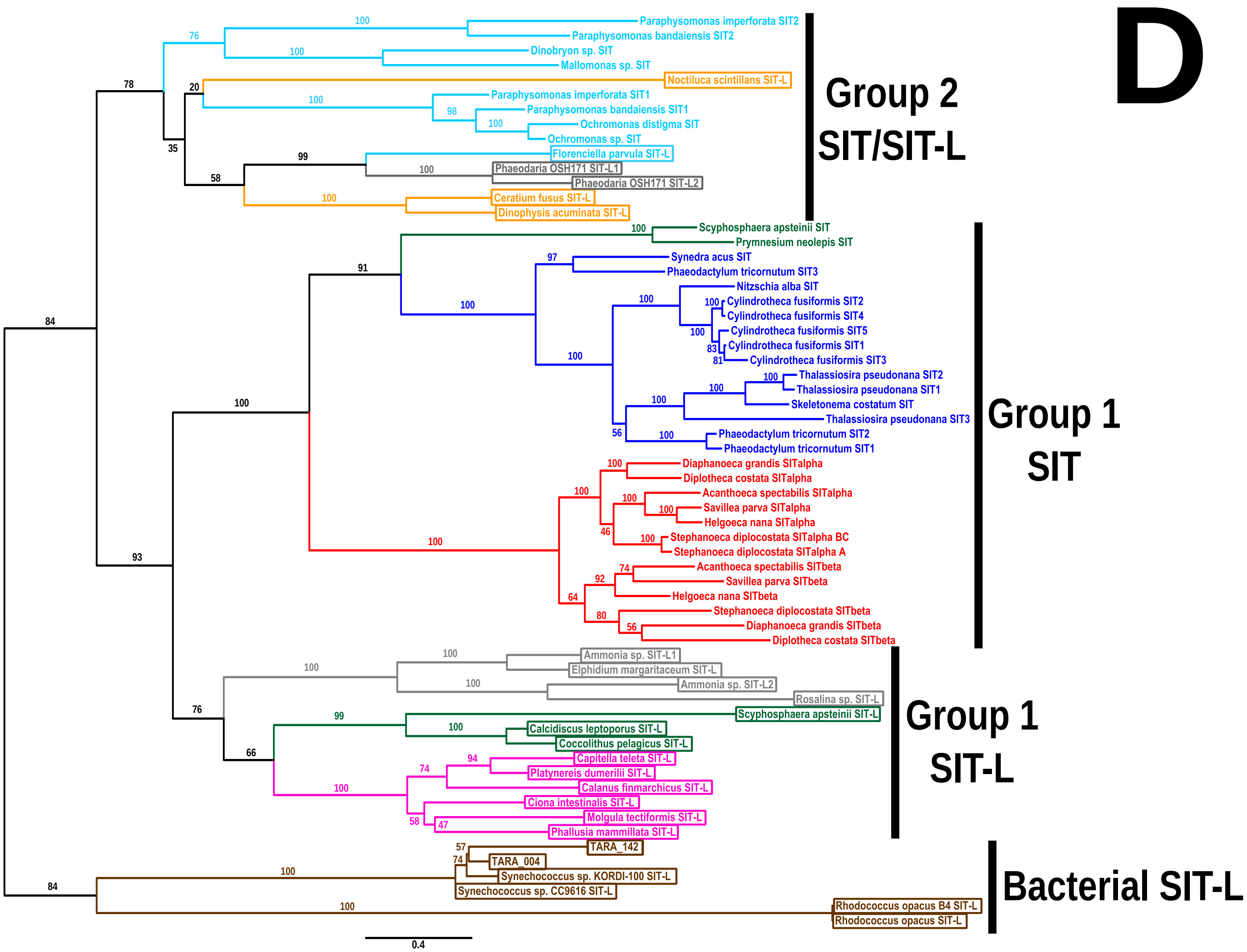
D
Paraphysomonas imperforata SIT2
Paraphysomonas bandaiensis SIT2
Dinobryon sp. SIT
Mallomonas sp. SIT
Noctiluca scintillans SIT-L
Paraphysomonas imperforata SIT1
Paraphysomonas bandaiensis SIT1
Ochromonas distigma SIT
Ochromonas sp. SIT
Florenciella parvula SIT-L
Phaeodaria OSH171 SIT-L1
Phaeodaria OSH171 SIT-L2
Ceratium fusus SIT-L
Dinophysis acuminata SIT-L
Group 2
SIT/SIT-L
Scyphosphaera apsteinii SIT
Prymnesium neolepis SIT
Synedra acus SIT
Phaeodactylum tricornutum SIT3
Nitzschia alba SIT
Cylindrotheca fusiformis SIT2
Cylindrotheca fusiformis SIT4
Cylindrotheca fusiformis SIT5
Cylindrotheca fusiformis SIT1
Cylindrotheca fusiformis SIT3
Thalassiosira pseudonana SIT2
Thalassiosira pseudonana SIT1
Skeletonema costatum SIT
Thalassiosira pseudonana SIT3
Phaeodactylum tricornutum SIT2
Phaeodactylum tricornutum SIT1
Diaphanoeca grandis SITalpha
Diplotheca costata SITalpha
Acanthoeca spectabilis SITalpha
Savillea parva SITalpha
Helgoeca nana SITalpha
Stephanoeca diplocostata SITalpha BC
Stephanoeca diplocostata SITalpha A
Acanthoeca spectabilis SITbeta
Savillea parva SITbeta
Helgoeca nana SITbeta
Stephanoeca diplocostata SITbeta
Diaphanoeca grandis SITbeta
Diplotheca costata SITbeta
Group 1
SIT
Ammonia sp. SIT-L1
Elphidium margaritaceum SIT-L
Ammonia sp. SIT-L2
Rosalina sp. SIT-L
Scyphosphaera apsteinii SIT-L
Calcidiscus leptoporus SIT-L
Coccolithus pelagicus SIT-L
Capitella teleta SIT-L
Platynereis dumerilii SIT-L
Calanus finmarchicus SIT-L
Ciona intestinalis SIT-L
Molgula tectiformis SIT-L
Phallusia mammillata SIT-L
Group 1
SIT-L
TARA_142
TARA_004
Synechococcus sp. KORDI-100 SIT-L
Synechococcus sp. CC9616 SIT-L
Rhodococcus opacus B4 SIT-L
Rhodococcus opacus SIT-L
Bacterial SIT-L
0.4

Supplementary Figure 6. Phylogenetic trees of Silicon Transporters. Trees generated using alternative phylogenetic analysis methods are congruous with Fig 4. Note that all the rectangular trees presented here are unrooted and have identical topologies to the radial tree in Fig. 4. The bacterial SIT-L clade has been arbitrarily designated as an outgroup to the eukaryotic SIT and SIT-L clades for presentation purposes. (A) Tree produced using PhyML maximum likelihood analysis. Numbers at nodes indicate statistical support as a percentage of 1000 SH-aLRT replicates/ 100 bootstrap replicates. (B) Majority rule consensus tree produced using MrBayes Bayesian MCMC analysis. Numbers at nodes indicate posterior probability values. (C) Tree produced using IQ-TREE maximum likelihood analysis. Numbers at nodes indicate statistical support as a percentage of 1000 SH-aLRT replicates/1000 ultrafast bootstrap replicates. (D) Tree produced using RaxML maximum likelihood analysis. Numbers at nodes indicate statistical support as a percentage of 100 bootstrap replicates. Brown= Bacteria, Dark Green= Haptophyte, Grey= Rhizarian (Light Grey= Foraminifera), Bright Red= Choanoflagellate, Magenta= Metazoan, Orange= Dinoflagellate, Dark Blue= Diatoms, Light Blue= Other Stramenopiles. SIT-L sequences are in boxes. All trees produced from the same alignment of 485 amino acid residues using the LG+G4+F model (A, B and D) or the LG+R5+F model (C). Scale bars indicate average number of amino acid substitutions per site.
