# Supplementary material for: The Evolution of Silicon Transport in Eukaryotes
Source: Mol Biol Evol. 2016 Oct 11;33(12):3226–48. doi: 10.1093/molbev/msw209 (PMC5100055; doi:10.1093/molbev/msw209)
Supplement: Supplementary Data [file supp_msw209_suppl_data.zip › Supplementary_Figures_7-8.pdf]

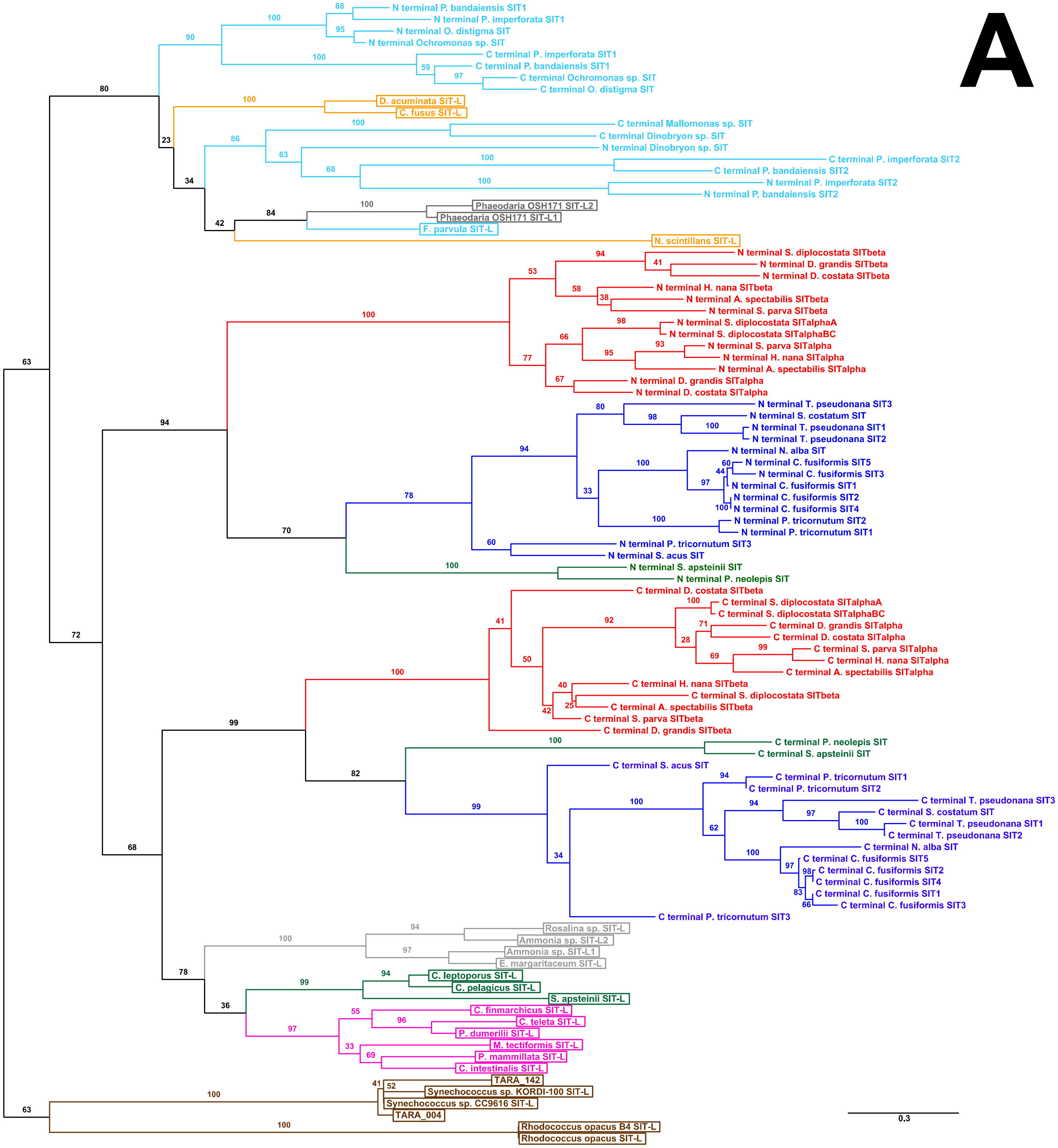
A
N terminal P. bandaiensis SIT1
N terminal P. imperforata SIT1
N terminal O. distigma SIT
N terminal Ochromonas sp. SIT
C terminal P. imperforata SIT1
C terminal P. bandaiensis SIT1
C terminal Ochromonas sp. SIT
C terminal O. distigma SIT
D. acuminata SIT-L
C. fusus SIT-L
C terminal Mallomonas sp. SIT
C terminal Dinobryon sp. SIT
N terminal Dinobryon sp. SIT
C terminal P. imperforata SIT2
C terminal P. bandaiensis SIT2
N terminal P. imperforata SIT2
N terminal P. bandaiensis SIT2
Phaeodaria OSH171 SIT-L2
Phaeodaria OSH171 SIT-L1
F. parvula SIT-L
N. scintillans SIT-L
N terminal S. diplocostata SITbeta
N terminal D. grandis SITbeta
N terminal D. costata SITbeta
N terminal H. nana SITbeta
N terminal A. spectabilis SITbeta
N terminal S. parva SITbeta
N terminal S. diplocostata SITalphaA
N terminal S. diplocostata SITalphaBC
N terminal S. parva SITalpha
N terminal H. nana SITalpha
N terminal A. spectabilis SITalpha
N terminal D. grandis SITalpha
N terminal D. costata SITalpha
N terminal T. pseudonana SIT3
N terminal S. costatum SIT
N terminal T. pseudonana SIT1
N terminal T. pseudonana SIT2
N terminal N. alba SIT
N terminal C. fusiformis SIT5
N terminal C. fusiformis SIT3
N terminal C. fusiformis SIT1
N terminal C. fusiformis SIT2
N terminal C. fusiformis SIT4
N terminal P. tricornutum SIT2
N terminal P. tricornutum SIT1
N terminal P. tricornutum SIT3
N terminal S. acus SIT
N terminal S. apsteinii SIT
N terminal P. neolepis SIT
C terminal D. costata SITbeta
C terminal S. diplocostata SITalphaA
C terminal S. diplocostata SITalphaBC
C terminal D. grandis SITalpha
C terminal D. costata SITalpha
C terminal S. parva SITalpha
C terminal H. nana SITalpha
C terminal A. spectabilis SITalpha
C terminal H. nana SITbeta
C terminal S. diplocostata SITbeta
C terminal A. spectabilis SITbeta
C terminal S. parva SITbeta
C terminal D. grandis SITbeta
C terminal P. neolepis SIT
C terminal S. apsteinii SIT
C terminal S. acus SIT
C terminal P. tricornutum SIT1
C terminal P. tricornutum SIT2
C terminal T. pseudonana SIT3
C terminal S. costatum SIT
C terminal T. pseudonana SIT1
C terminal T. pseudonana SIT2
C terminal N. alba SIT
C terminal C. fusiformis SIT5
C terminal C. fusiformis SIT2
C terminal C. fusiformis SIT4
C terminal C. fusiformis SIT1
C terminal C. fusiformis SIT3
C terminal P. tricornutum SIT3
Rosalina sp. SIT-L
Ammonia sp. SIT-L2
Ammonia sp. SIT-L1
E. margaritaceum SIT-L
C. leptoporus SIT-L
C. pelagicus SIT-L
S. apsteinii SIT-L
C. finmarchicus SIT-L
C. teleta SIT-L
P. dumerilii SIT-L
M. tectiformis SIT-L
P. mammillata SIT-L
C. intestinalis SIT-L
TARA_142
Synechococcus sp. KORDI-100 SIT-L
Synechococcus sp. CC9616 SIT-L
TARA_004
Rhodococcus opacus B4 SIT-L
Rhodococcus opacus SIT-L
0.3

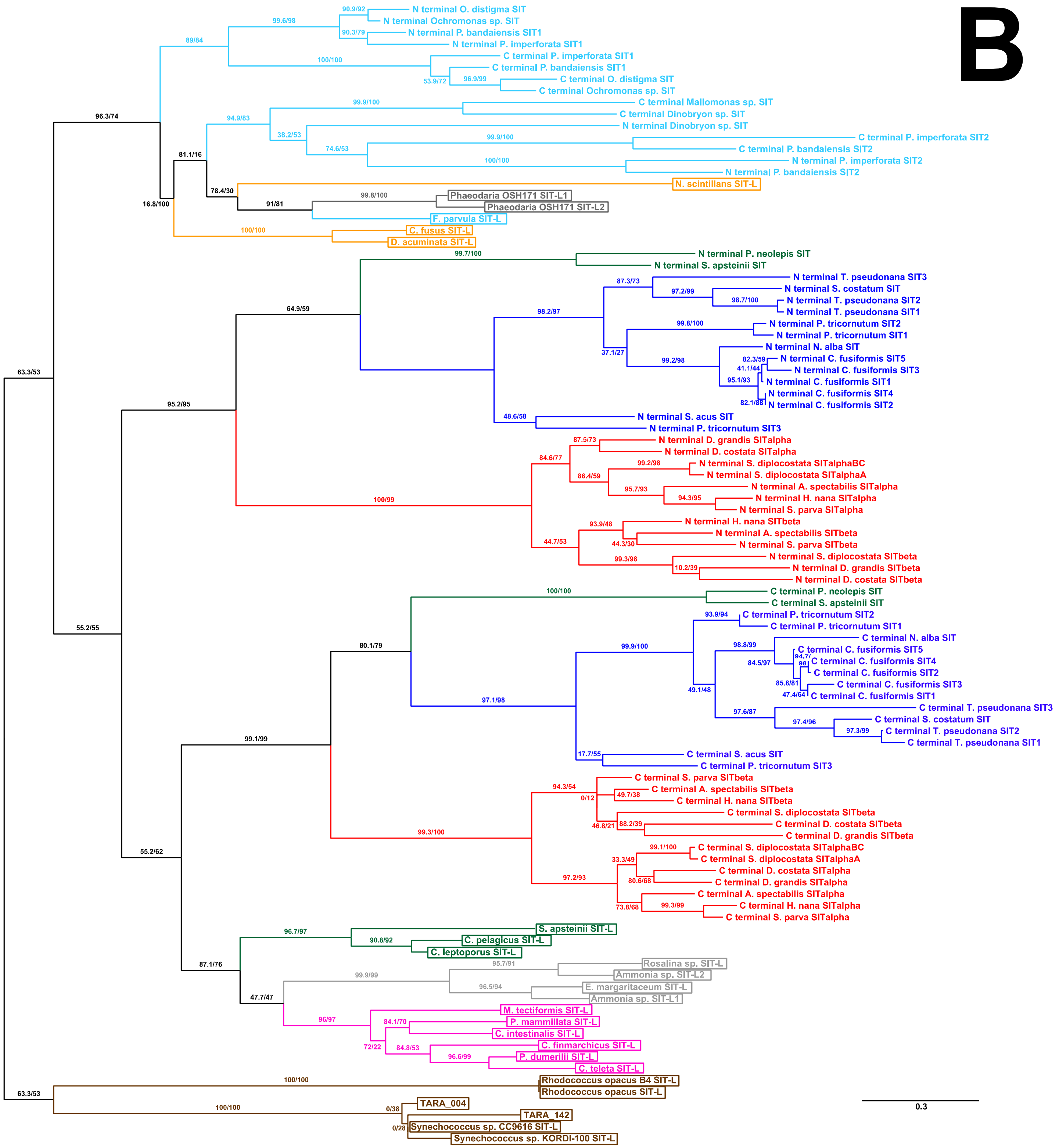
B
N terminal O. distigma SIT
N terminal Ochromonas sp. SIT
N terminal P. bandaiensis SIT1
N terminal P. imperforata SIT1
C terminal P. imperforata SIT1
C terminal P. bandaiensis SIT1
C terminal O. distigma SIT
C terminal Ochromonas sp. SIT
C terminal Mallomonas sp. SIT
C terminal Dinobryon sp. SIT
N terminal Dinobryon sp. SIT
C terminal P. imperforata SIT2
C terminal P. bandaiensis SIT2
N terminal P. imperforata SIT2
N terminal P. bandaiensis SIT2
N. scintillans SIT-L
Phaeodaria OSH171 SIT-L1
Phaeodaria OSH171 SIT-L2
F. parvula SIT-L
C. fusus SIT-L
D. acuminata SIT-L
N terminal P. neolepis SIT
N terminal S. apsteinii SIT
N terminal T. pseudonana SIT3
N terminal S. costatum SIT
N terminal T. pseudonana SIT2
N terminal T. pseudonana SIT1
N terminal P. tricornutum SIT2
N terminal P. tricornutum SIT1
N terminal N. alba SIT
N terminal C. fusiformis SIT5
N terminal C. fusiformis SIT3
N terminal C. fusiformis SIT1
N terminal C. fusiformis SIT4
N terminal C. fusiformis SIT2
N terminal S. acus SIT
N terminal P. tricornutum SIT3
N terminal D. grandis SITalpha
N terminal D. costata SITalpha
N terminal S. diplocostata SITalphaBC
N terminal S. diplocostata SITalphaA
N terminal A. spectabilis SITalpha
N terminal H. nana SITalpha
N terminal S. parva SITalpha
N terminal H. nana SITbeta
N terminal A. spectabilis SITbeta
N terminal S. parva SITbeta
N terminal S. diplocostata SITbeta
N terminal D. grandis SITbeta
N terminal D. costata SITbeta
C terminal P. neolepis SIT
C terminal S. apsteinii SIT
C terminal P. tricornutum SIT2
C terminal P. tricornutum SIT1
C terminal N. alba SIT
C terminal C. fusiformis SIT5
C terminal C. fusiformis SIT4
C terminal C. fusiformis SIT2
C terminal C. fusiformis SIT3
C terminal C. fusiformis SIT1
C terminal T. pseudonana SIT3
C terminal S. costatum SIT
C terminal T. pseudonana SIT2
C terminal T. pseudonana SIT1
C terminal S. acus SIT
C terminal P. tricornutum SIT3
C terminal S. parva SITbeta
C terminal A. spectabilis SITbeta
C terminal H. nana SITbeta
C terminal S. diplocostata SITbeta
C terminal D. costata SITbeta
C terminal D. grandis SITbeta
C terminal S. diplocostata SITalphaBC
C terminal S. diplocostata SITalphaA
C terminal D. costata SITalpha
C terminal D. grandis SITalpha
C terminal A. spectabilis SITalpha
C terminal H. nana SITalpha
C terminal S. parva SITalpha
S. apsteinii SIT-L
C. pelagicus SIT-L
C. leptoporus SIT-L
Rosalina sp. SIT-L
Ammonia sp. SIT-L2
E. margaritaceum SIT-L
Ammonia sp. SIT-L1
M. tectiformis SIT-L
P. mammillata SIT-L
C. intestinalis SIT-L
C. finmarchicus SIT-L
P. dumerilii SIT-L
C. teleta SIT-L
Rhodococcus opacus B4 SIT-L
Rhodococcus opacus SIT-L
TARA_004
TARA_142
Synechococcus sp. CC9616 SIT-L
Synechococcus sp. KORDI-100 SIT-L
0.3

C

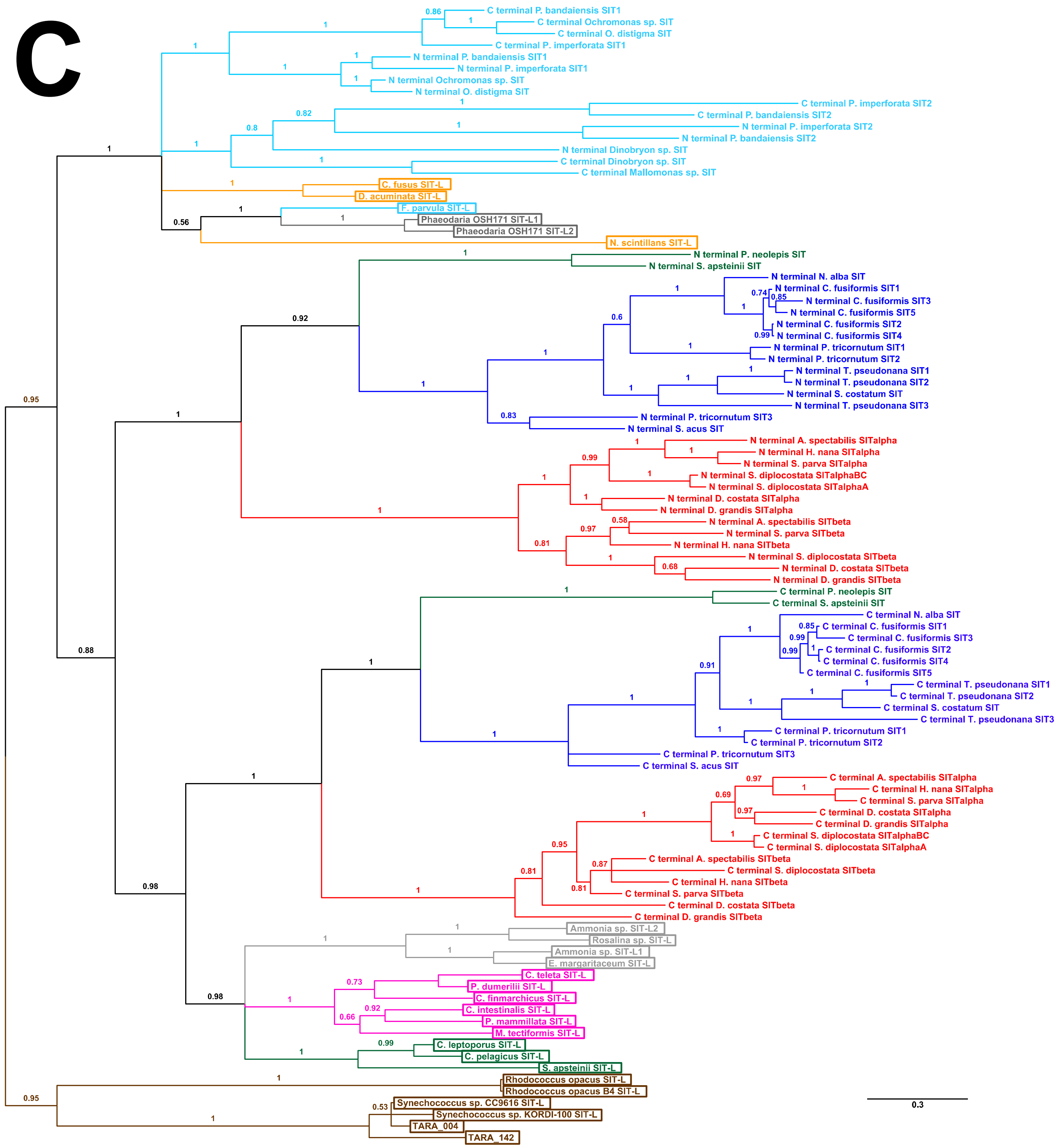
C terminal P. bandaiensis SIT1
C terminal Ochromonas sp. SIT
C terminal O. distigma SIT
C terminal P. imperforata SIT1
N terminal P. bandaiensis SIT1
N terminal P. imperforata SIT1
N terminal Ochromonas sp. SIT
N terminal O. distigma SIT
C terminal P. imperforata SIT2
C terminal P. bandaiensis SIT2
N terminal P. imperforata SIT2
N terminal P. bandaiensis SIT2
N terminal Dinobryon sp. SIT
C terminal Dinobryon sp. SIT
C terminal Mallomonas sp. SIT
C. fusus SIT-L
D. acuminata SIT-L
F. parvula SIT-L
Phaeodaria OSH171 SIT-L1
Phaeodaria OSH171 SIT-L2
N. scintillans SIT-L
N terminal P. neolepis SIT
N terminal S. apsteinii SIT
N terminal N. alba SIT
N terminal C. fusiformis SIT1
N terminal C. fusiformis SIT3
N terminal C. fusiformis SIT5
N terminal C. fusiformis SIT2
N terminal C. fusiformis SIT4
N terminal P. tricornutum SIT1
N terminal P. tricornutum SIT2
N terminal T. pseudonana SIT1
N terminal T. pseudonana SIT2
N terminal S. costatum SIT
N terminal T. pseudonana SIT3
N terminal P. tricornutum SIT3
N terminal S. acus SIT
N terminal A. spectabilis SITalpha
N terminal H. nana SITalpha
N terminal S. parva SITalpha
N terminal S. diplocostata SITalphaBC
N terminal S. diplocostata SITalphaA
N terminal D. costata SITalpha
N terminal D. grandis SITalpha
N terminal A. spectabilis SITbeta
N terminal S. parva SITbeta
N terminal H. nana SITbeta
N terminal S. diplocostata SITbeta
N terminal D. costata SITbeta
N terminal D. grandis SITbeta
C terminal P. neolepis SIT
C terminal S. apsteinii SIT
C terminal N. alba SIT
C terminal C. fusiformis SIT1
C terminal C. fusiformis SIT3
C terminal C. fusiformis SIT2
C terminal C. fusiformis SIT4
C terminal C. fusiformis SIT5
C terminal T. pseudonana SIT1
C terminal T. pseudonana SIT2
C terminal S. costatum SIT
C terminal T. pseudonana SIT3
C terminal P. tricornutum SIT1
C terminal P. tricornutum SIT2
C terminal P. tricornutum SIT3
C terminal S. acus SIT
C terminal A. spectabilis SITalpha
C terminal H. nana SITalpha
C terminal S. parva SITalpha
C terminal D. costata SITalpha
C terminal D. grandis SITalpha
C terminal S. diplocostata SITalphaBC
C terminal S. diplocostata SITalphaA
C terminal A. spectabilis SITbeta
C terminal S. diplocostata SITbeta
C terminal H. nana SITbeta
C terminal S. parva SITbeta
C terminal D. costata SITbeta
C terminal D. grandis SITbeta
Ammonia sp. SIT-L2
Rosalina sp. SIT-L
Ammonia sp. SIT-L1
E. margaritaceum SIT-L
C. teleta SIT-L
P. dumerilii SIT-L
C. finmarchicus SIT-L
C. intestinalis SIT-L
P. mammillata SIT-L
M. tectiformis SIT-L
C. leptoporus SIT-L
C. pelagicus SIT-L
S. apsteinii SIT-L
Rhodococcus opacus SIT-L
Rhodococcus opacus B4 SIT-L
Synechococcus sp. CC9616 SIT-L
Synechococcus sp. KORDI-100 SIT-L
TARA_004
TARA_142
0.3

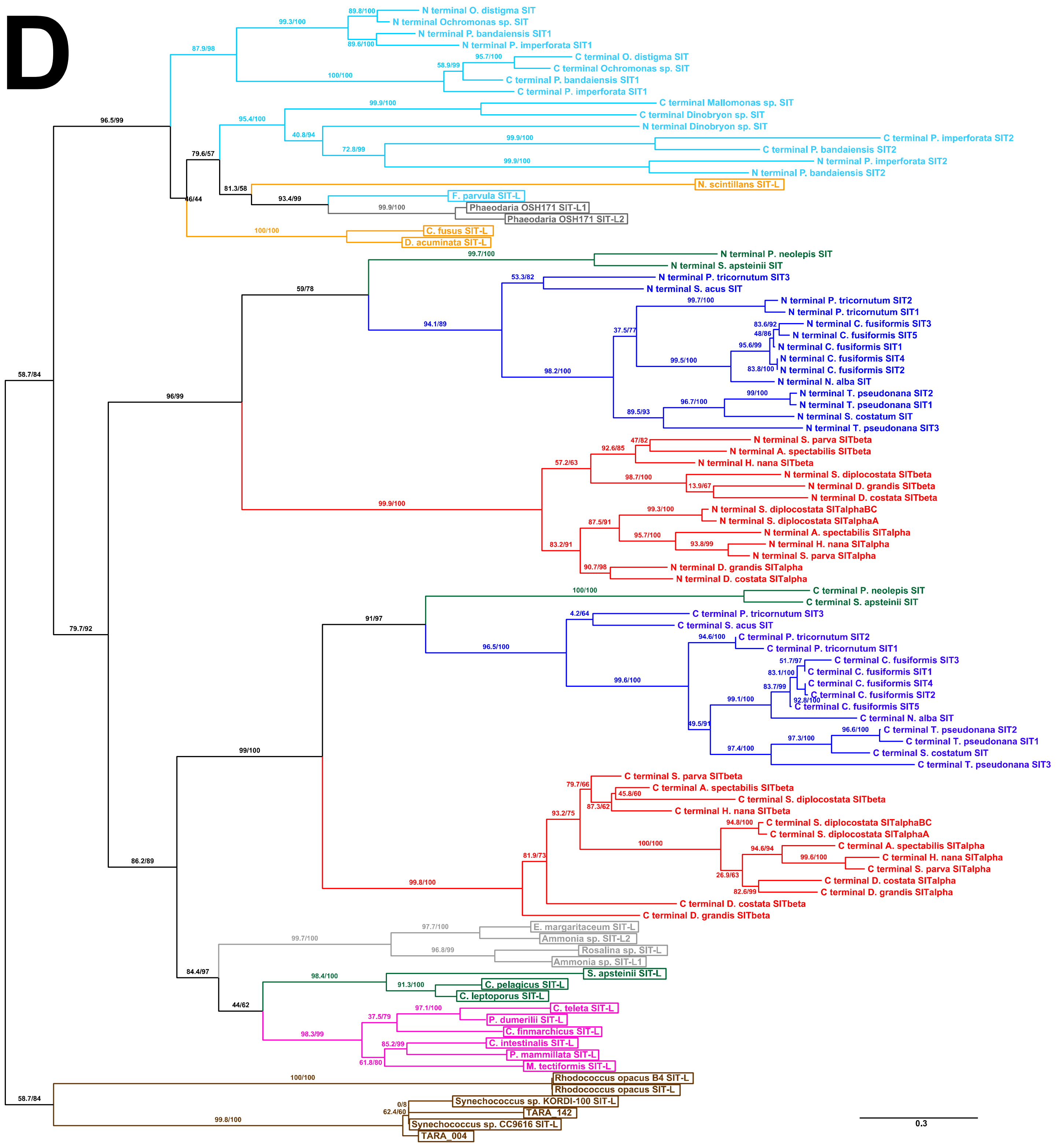
D
N terminal O. distigma SIT
N terminal Ochromonas sp. SIT
N terminal P. bandaiensis SIT1
N terminal P. imperforata SIT1
C terminal O. distigma SIT
C terminal Ochromonas sp. SIT
C terminal P. bandaiensis SIT1
C terminal P. imperforata SIT1
C terminal Mallomonas sp. SIT
C terminal Dinobryon sp. SIT
N terminal Dinobryon sp. SIT
C terminal P. imperforata SIT2
C terminal P. bandaiensis SIT2
N terminal P. imperforata SIT2
N terminal P. bandaiensis SIT2
N. scintillans SIT-L
F. parvula SIT-L
Phaeodaria OSH171 SIT-L1
Phaeodaria OSH171 SIT-L2
C. fusus SIT-L
D. acuminata SIT-L
N terminal P. neolepis SIT
N terminal S. apsteinii SIT
N terminal P. tricornutum SIT3
N terminal S. acus SIT
N terminal P. tricornutum SIT2
N terminal P. tricornutum SIT1
N terminal C. fusiformis SIT3
N terminal C. fusiformis SIT5
N terminal C. fusiformis SIT1
N terminal C. fusiformis SIT4
N terminal C. fusiformis SIT2
N terminal N. alba SIT
N terminal T. pseudonana SIT2
N terminal T. pseudonana SIT1
N terminal S. costatum SIT
N terminal T. pseudonana SIT3
N terminal S. parva SITbeta
N terminal A. spectabilis SITbeta
N terminal H. nana SITbeta
N terminal S. diplocostata SITbeta
N terminal D. grandis SITbeta
N terminal D. costata SITbeta
N terminal S. diplocostata SITalphaBC
N terminal S. diplocostata SITalphaA
N terminal A. spectabilis SITalpha
N terminal H. nana SITalpha
N terminal S. parva SITalpha
N terminal D. grandis SITalpha
N terminal D. costata SITalpha
C terminal P. neolepis SIT
C terminal S. apsteinii SIT
C terminal P. tricornutum SIT3
C terminal S. acus SIT
C terminal P. tricornutum SIT2
C terminal P. tricornutum SIT1
C terminal C. fusiformis SIT3
C terminal C. fusiformis SIT1
C terminal C. fusiformis SIT4
C terminal C. fusiformis SIT2
C terminal C. fusiformis SIT5
C terminal N. alba SIT
C terminal T. pseudonana SIT2
C terminal T. pseudonana SIT1
C terminal S. costatum SIT
C terminal T. pseudonana SIT3
C terminal S. parva SITbeta
C terminal A. spectabilis SITbeta
C terminal S. diplocostata SITbeta
C terminal H. nana SITbeta
C terminal S. diplocostata SITalphaBC
C terminal S. diplocostata SITalphaA
C terminal A. spectabilis SITalpha
C terminal H. nana SITalpha
C terminal S. parva SITalpha
C terminal D. costata SITalpha
C terminal D. grandis SITalpha
C terminal D. costata SITbeta
C terminal D. grandis SITbeta
E. margaritaceum SIT-L
Ammonia sp. SIT-L2
Rosalina sp. SIT-L
Ammonia sp. SIT-L1
S. apsteinii SIT-L
C. pelagicus SIT-L
C. leptoporus SIT-L
C. teleta SIT-L
P. dumerilii SIT-L
C. finmarchicus SIT-L
C. intestinalis SIT-L
P. mammillata SIT-L
M. tectiformis SIT-L
Rhodococcus opacus B4 SIT-L
Rhodococcus opacus SIT-L
Synechococcus sp. KORDI-100 SIT-L
TARA_142
Synechococcus sp. CC9616 SIT-L
TARA_004
0.3

Supplementary Figure 7. Complete phylogenetic trees of SIT N-terminal halves, SIT C-terminal halves and SIT-Ls. (A) Tree produced using RaxML maximum likelihood analysis. Numbers at nodes indicate statistical support as a percentage of 100 bootstrap replicates. (B) Tree produced using PhyML maximum likelihood analysis. Numbers at nodes indicate statistical support as a percentage of 1000 SH-aLRT replicates/ 100 bootstrap replicates. (C) Majority rule consensus tree produced using MrBayes Bayesian MCMC analysis. Numbers at nodes indicate posterior probability values. (D) Tree produced using IQ-TREE maximum likelihood analysis. Numbers at nodes indicate statistical support as a percentage of 1000 SH-aLRT replicates/1000 ultrafast bootstrap replicates. Note that in all trees the topology is unrooted; the bacterial SIT-L clade was arbitrarily designated as an outgroup for presentation purposes. Brown= Bacteria, Green= Haptophyte, Grey= Rhizarian (Light Grey= Foraminifera), Red= Choanoflagellate, Magenta= Metazoan, Orange= Dinoflagellate, Dark Blue= Diatom, Light Blue= Other Stramenopiles. SIT-L sequences are in boxes. All trees produced from the same alignment of 166 amino acid residues using the LG+G4 model. Scale bars indicate average number of amino acid substitutions per site.

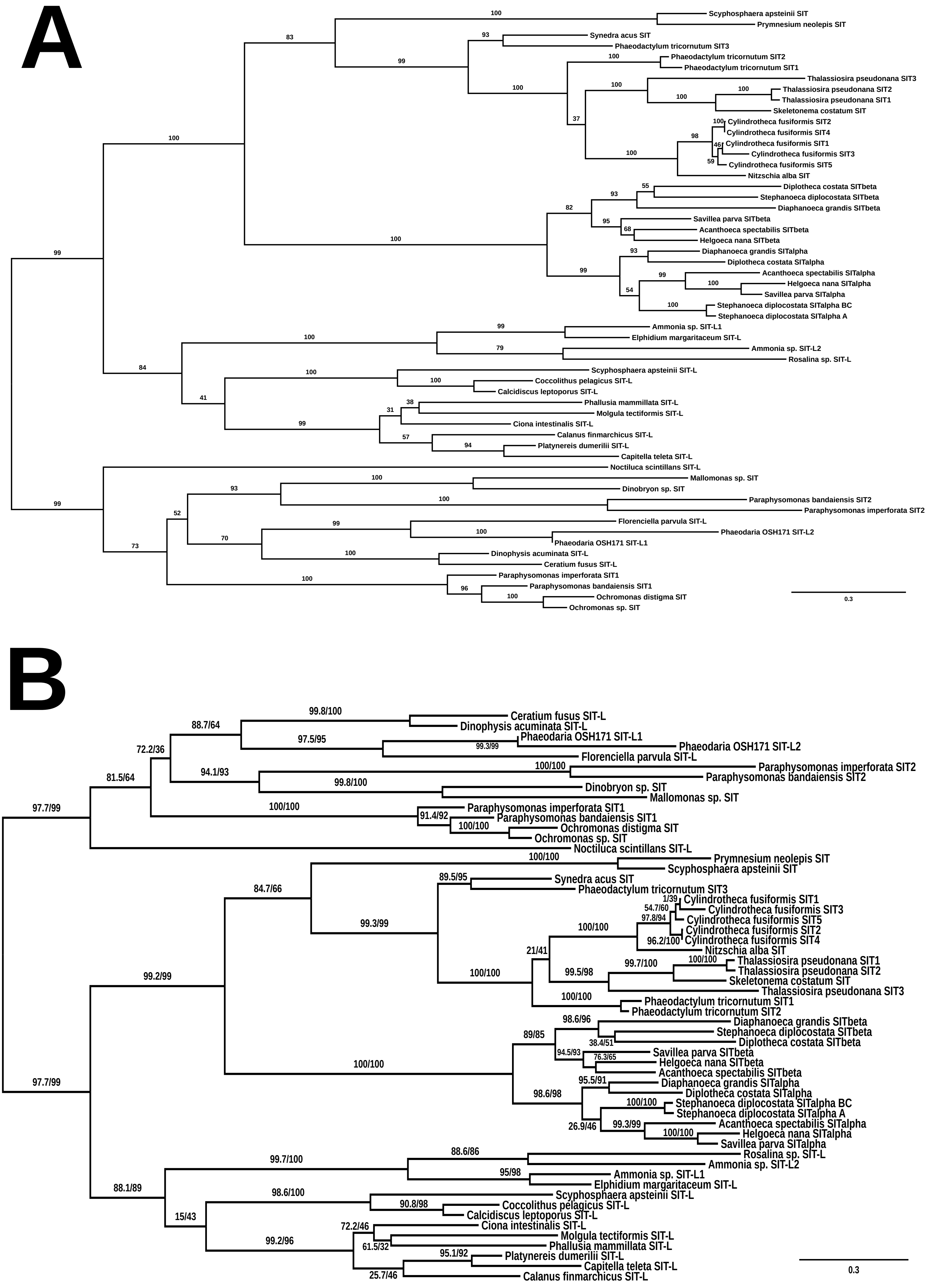
A
Scyphosphaera apsteinii SIT
Prymnesium neolepis SIT
Synedra acus SIT
Phaeodactylum tricornutum SIT3
Phaeodactylum tricornutum SIT2
Phaeodactylum tricornutum SIT1
Thalassiosira pseudonana SIT3
Thalassiosira pseudonana SIT2
Thalassiosira pseudonana SIT1
Skeletonema costatum SIT
Cylindrotheca fusiformis SIT2
Cylindrotheca fusiformis SIT4
Cylindrotheca fusiformis SIT1
Cylindrotheca fusiformis SIT3
Cylindrotheca fusiformis SIT5
Nitzschia alba SIT
Diplotheca costata SITbeta
Stephanoeca diplocostata SITbeta
Diaphanoeca grandis SITbeta
Savillea parva SITbeta
Acanthoeca spectabilis SITbeta
Helgoeca nana SITbeta
Diaphanoeca grandis SITalpha
Diplotheca costata SITalpha
Acanthoeca spectabilis SITalpha
Helgoeca nana SITalpha
Savillea parva SITalpha
Stephanoeca diplocostata SITalpha BC
Stephanoeca diplocostata SITalpha A
Ammonia sp. SIT-L1
Elphidium margaritaceum SIT-L
Ammonia sp. SIT-L2
Rosalina sp. SIT-L
Scyphosphaera apsteinii SIT-L
Coccolithus pelagicus SIT-L
Calcidiscus leptoporus SIT-L
Phallusia mammillata SIT-L
Molgula tectiformis SIT-L
Ciona intestinalis SIT-L
Calanus finmarchicus SIT-L
Platynereis dumerilii SIT-L
Capitella teleta SIT-L
Noctiluca scintillans SIT-L
Mallomonas sp. SIT
Dinobryon sp. SIT
Paraphysomonas bandaiensis SIT2
Paraphysomonas imperforata SIT2
Florenciella parvula SIT-L
Phaeodaria OSH171 SIT-L2
Phaeodaria OSH171 SIT-L1
Dinophysis acuminata SIT-L
Ceratium fusus SIT-L
Paraphysomonas imperforata SIT1
Paraphysomonas bandaiensis SIT1
Ochromonas distigma SIT
Ochromonas sp. SIT
0.3
B
Ceratium fusus SIT-L
Dinophysis acuminata SIT-L
Phaeodaria OSH171 SIT-L1
Phaeodaria OSH171 SIT-L2
Florenciella parvula SIT-L
Paraphysomonas imperforata SIT2
Paraphysomonas bandaiensis SIT2
Dinobryon sp. SIT
Mallomonas sp. SIT
Paraphysomonas imperforata SIT1
Paraphysomonas bandaiensis SIT1
Ochromonas distigma SIT
Ochromonas sp. SIT
Noctiluca scintillans SIT-L
Prymnesium neolepis SIT
Scyphosphaera apsteinii SIT
Synedra acus SIT
Phaeodactylum tricornutum SIT3
Cylindrotheca fusiformis SIT1
Cylindrotheca fusiformis SIT3
Cylindrotheca fusiformis SIT5
Cylindrotheca fusiformis SIT2
Cylindrotheca fusiformis SIT4
Nitzschia alba SIT
Thalassiosira pseudonana SIT1
Thalassiosira pseudonana SIT2
Skeletonema costatum SIT
Thalassiosira pseudonana SIT3
Phaeodactylum tricornutum SIT1
Phaeodactylum tricornutum SIT2
Diaphanoeca grandis SITbeta
Stephanoeca diplocostata SITbeta
Diplotheca costata SITbeta
Savillea parva SITbeta
Helgoeca nana SITbeta
Acanthoeca spectabilis SITbeta
Diaphanoeca grandis SITalpha
Diplotheca costata SITalpha
Stephanoeca diplocostata SITalpha BC
Stephanoeca diplocostata SITalpha A
Acanthoeca spectabilis SITalpha
Helgoeca nana SITalpha
Savillea parva SITalpha
Rosalina sp. SIT-L
Ammonia sp. SIT-L2
Ammonia sp. SIT-L1
Elphidium margaritaceum SIT-L
Scyphosphaera apsteinii SIT-L
Coccolithus pelagicus SIT-L
Calcidiscus leptoporus SIT-L
Ciona intestinalis SIT-L
Molgula tectiformis SIT-L
Phallusia mammillata SIT-L
Platynereis dumerilii SIT-L
Capitella teleta SIT-L
Calanus finmarchicus SIT-L
0.3

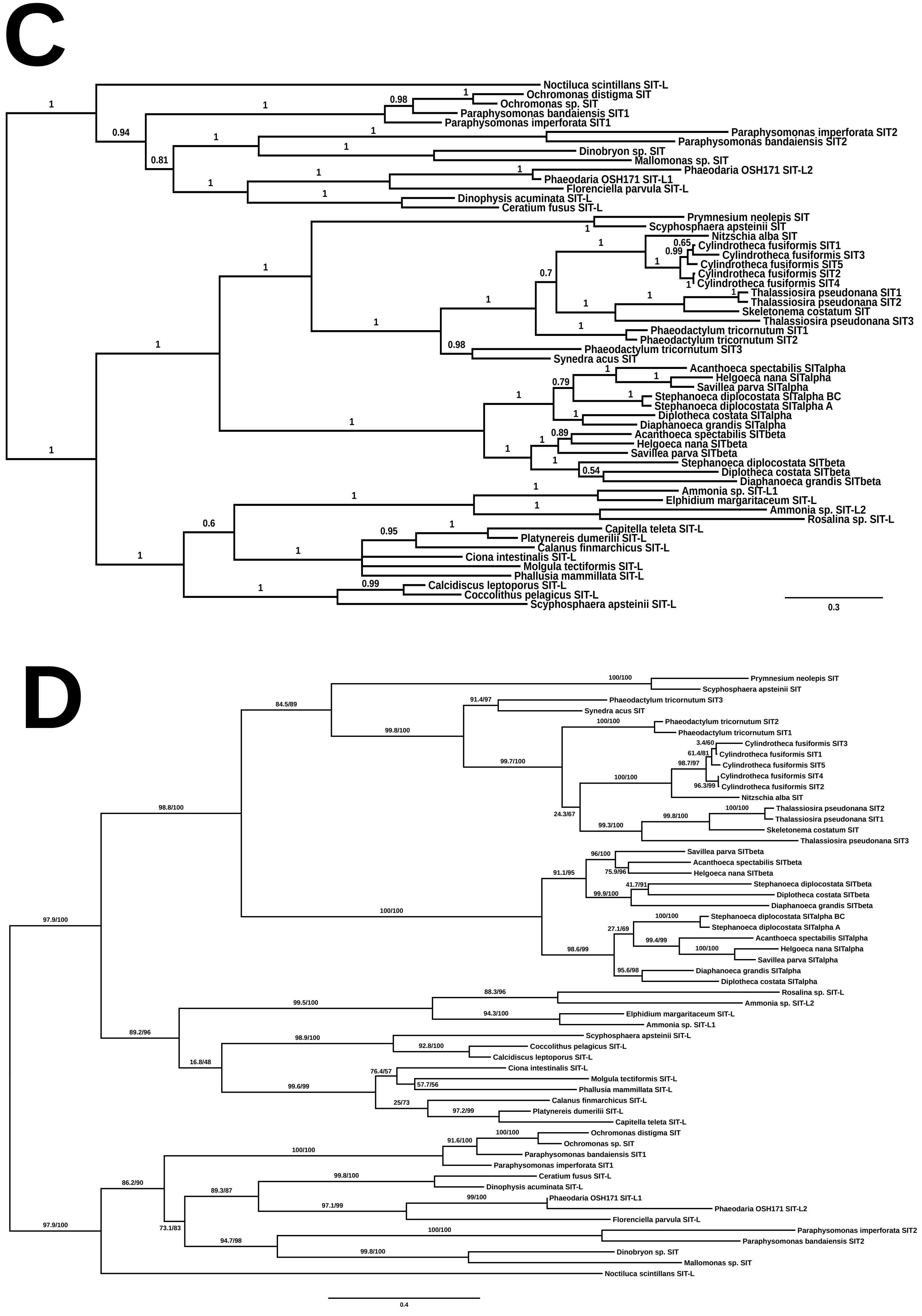
C
Noctiluca scintillans SIT-L
Ochromonas distigma SIT
Ochromonas sp. SIT
Paraphysomonas bandaiensis SIT1
Paraphysomonas imperforata SIT1
Paraphysomonas imperforata SIT2
Paraphysomonas bandaiensis SIT2
Dinobryon sp. SIT
Mallomonas sp. SIT
Phaeodaria OSH171 SIT-L2
Phaeodaria OSH171 SIT-L1
Florenciella parvula SIT-L
Dinophysis acuminata SIT-L
Ceratium fusus SIT-L
Prymnesium neolepis SIT
Scyphosphaera apsteinii SIT
Nitzschia alba SIT
Cylindrotheca fusiformis SIT1
Cylindrotheca fusiformis SIT3
Cylindrotheca fusiformis SIT5
Cylindrotheca fusiformis SIT2
Cylindrotheca fusiformis SIT4
Thalassiosira pseudonana SIT1
Thalassiosira pseudonana SIT2
Skeletonema costatum SIT
Thalassiosira pseudonana SIT3
Phaeodactylum tricornutum SIT1
Phaeodactylum tricornutum SIT2
Phaeodactylum tricornutum SIT3
Synedra acus SIT
Acanthoeca spectabilis SITalpha
Helgoeca nana SITalpha
Savillea parva SITalpha
Stephanoeca diplocostata SITalpha BC
Stephanoeca diplocostata SITalpha A
Diplotheca costata SITalpha
Diaphanoeca grandis SITalpha
Acanthoeca spectabilis SITbeta
Helgoeca nana SITbeta
Savillea parva SITbeta
Stephanoeca diplocostata SITbeta
Diplotheca costata SITbeta
Diaphanoeca grandis SITbeta
Ammonia sp. SIT-L1
Elphidium margaritaceum SIT-L
Ammonia sp. SIT-L2
Rosalina sp. SIT-L
Capitella teleta SIT-L
Platynereis dumerilii SIT-L
Calanus finmarchicus SIT-L
Ciona intestinalis SIT-L
Molgula tectiformis SIT-L
Phallusia mammillata SIT-L
Calcidiscus leptoporus SIT-L
Coccolithus pelagicus SIT-L
Scyphosphaera apsteinii SIT-L
0.3
D
Prymnesium neolepis SIT
Scyphosphaera apsteinii SIT
Phaeodactylum tricornutum SIT3
Synedra acus SIT
Phaeodactylum tricornutum SIT2
Phaeodactylum tricornutum SIT1
Cylindrotheca fusiformis SIT3
Cylindrotheca fusiformis SIT1
Cylindrotheca fusiformis SIT5
Cylindrotheca fusiformis SIT4
Cylindrotheca fusiformis SIT2
Nitzschia alba SIT
Thalassiosira pseudonana SIT2
Thalassiosira pseudonana SIT1
Skeletonema costatum SIT
Thalassiosira pseudonana SIT3
Savillea parva SITbeta
Acanthoeca spectabilis SITbeta
Helgoeca nana SITbeta
Stephanoeca diplocostata SITbeta
Diplotheca costata SITbeta
Diaphanoeca grandis SITbeta
Stephanoeca diplocostata SITalpha BC
Stephanoeca diplocostata SITalpha A
Acanthoeca spectabilis SITalpha
Helgoeca nana SITalpha
Savillea parva SITalpha
Diaphanoeca grandis SITalpha
Diplotheca costata SITalpha
Rosalina sp. SIT-L
Ammonia sp. SIT-L2
Elphidium margaritaceum SIT-L
Ammonia sp. SIT-L1
Scyphosphaera apsteinii SIT-L
Coccolithus pelagicus SIT-L
Calcidiscus leptoporus SIT-L
Ciona intestinalis SIT-L
Molgula tectiformis SIT-L
Phallusia mammillata SIT-L
Calanus finmarchicus SIT-L
Platynereis dumerilii SIT-L
Capitella teleta SIT-L
Ochromonas distigma SIT
Ochromonas sp. SIT
Paraphysomonas bandaiensis SIT1
Paraphysomonas imperforata SIT1
Ceratium fusus SIT-L
Dinophysis acuminata SIT-L
Phaeodaria OSH171 SIT-L1
Phaeodaria OSH171 SIT-L2
Florenciella parvula SIT-L
Paraphysomonas imperforata SIT2
Paraphysomonas bandaiensis SIT2
Dinobryon sp. SIT
Mallomonas sp. SIT
Noctiluca scintillans SIT-L
0.4

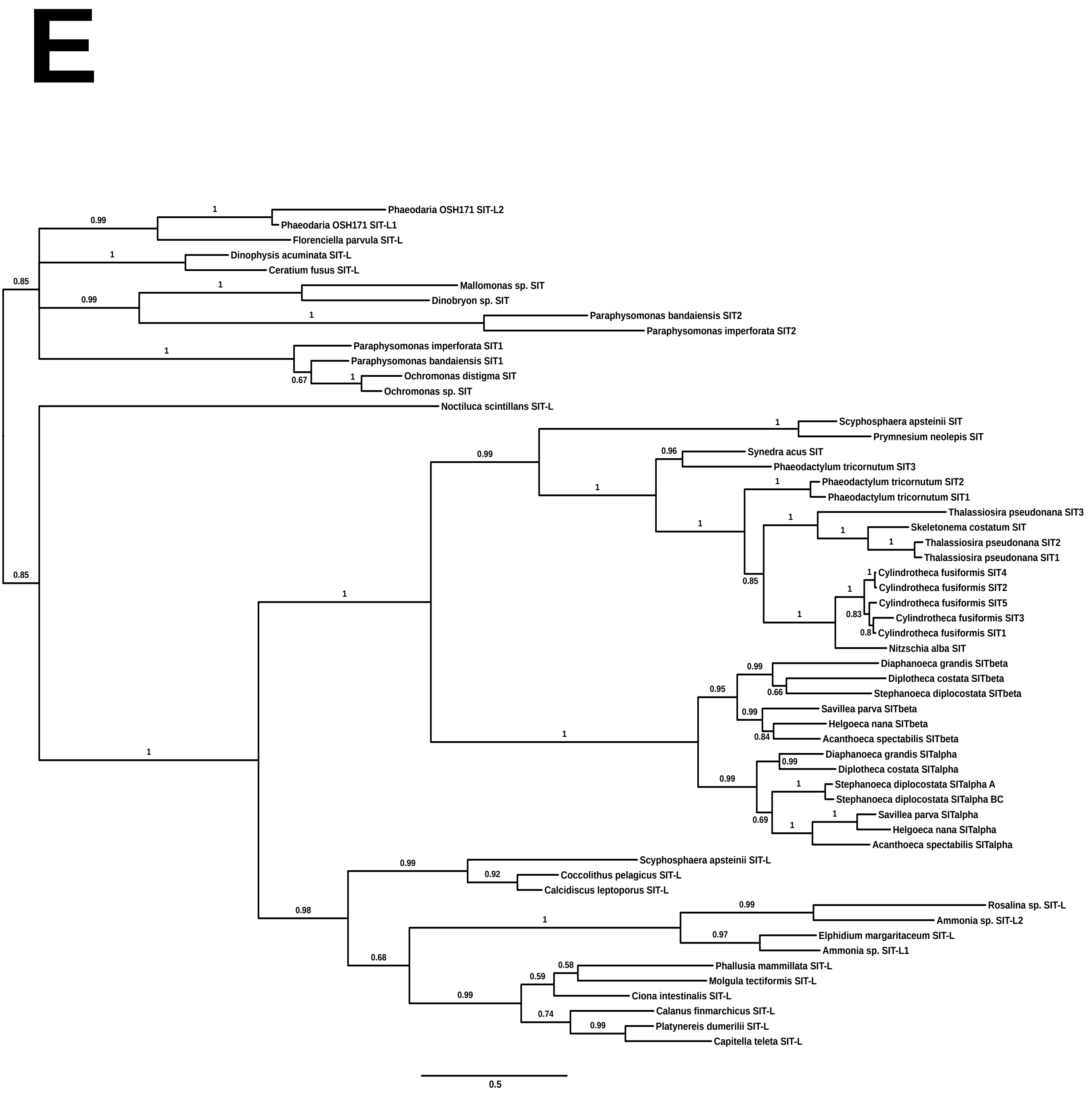
E
Phaeodaria OSH171 SIT-L2
Phaeodaria OSH171 SIT-L1
Florenciella parvula SIT-L
Dinophysis acuminata SIT-L
Ceratium fusus SIT-L
Mallomonas sp. SIT
Dinobryon sp. SIT
Paraphysomonas bandaiensis SIT2
Paraphysomonas imperforata SIT2
Paraphysomonas imperforata SIT1
Paraphysomonas bandaiensis SIT1
Ochromonas distigma SIT
Ochromonas sp. SIT
Noctiluca scintillans SIT-L
Scyphosphaera apsteinii SIT
Prymnesium neolepis SIT
Synedra acus SIT
Phaeodactylum tricornutum SIT3
Phaeodactylum tricornutum SIT2
Phaeodactylum tricornutum SIT1
Thalassiosira pseudonana SIT3
Skeletonema costatum SIT
Thalassiosira pseudonana SIT2
Thalassiosira pseudonana SIT1
Cylindrotheca fusiformis SIT4
Cylindrotheca fusiformis SIT2
Cylindrotheca fusiformis SIT5
Cylindrotheca fusiformis SIT3
Cylindrotheca fusiformis SIT1
Nitzschia alba SIT
Diaphanoeca grandis SITbeta
Diplotheca costata SITbeta
Stephanoeca diplocostata SITbeta
Savillea parva SITbeta
Helgoeca nana SITbeta
Acanthoeca spectabilis SITbeta
Diaphanoeca grandis SITalpha
Diplotheca costata SITalpha
Stephanoeca diplocostata SITalpha A
Stephanoeca diplocostata SITalpha BC
Savillea parva SITalpha
Helgoeca nana SITalpha
Acanthoeca spectabilis SITalpha
Scyphosphaera apsteinii SIT-L
Coccolithus pelagicus SIT-L
Calcidiscus leptoporus SIT-L
Rosalina sp. SIT-L
Ammonia sp. SIT-L2
Elphidium margaritaceum SIT-L
Ammonia sp. SIT-L1
Phallusia mammillata SIT-L
Molgula tectiformis SIT-L
Ciona intestinalis SIT-L
Calanus finmarchicus SIT-L
Platynereis dumerilii SIT-L
Capitella teleta SIT-L
0.5

F

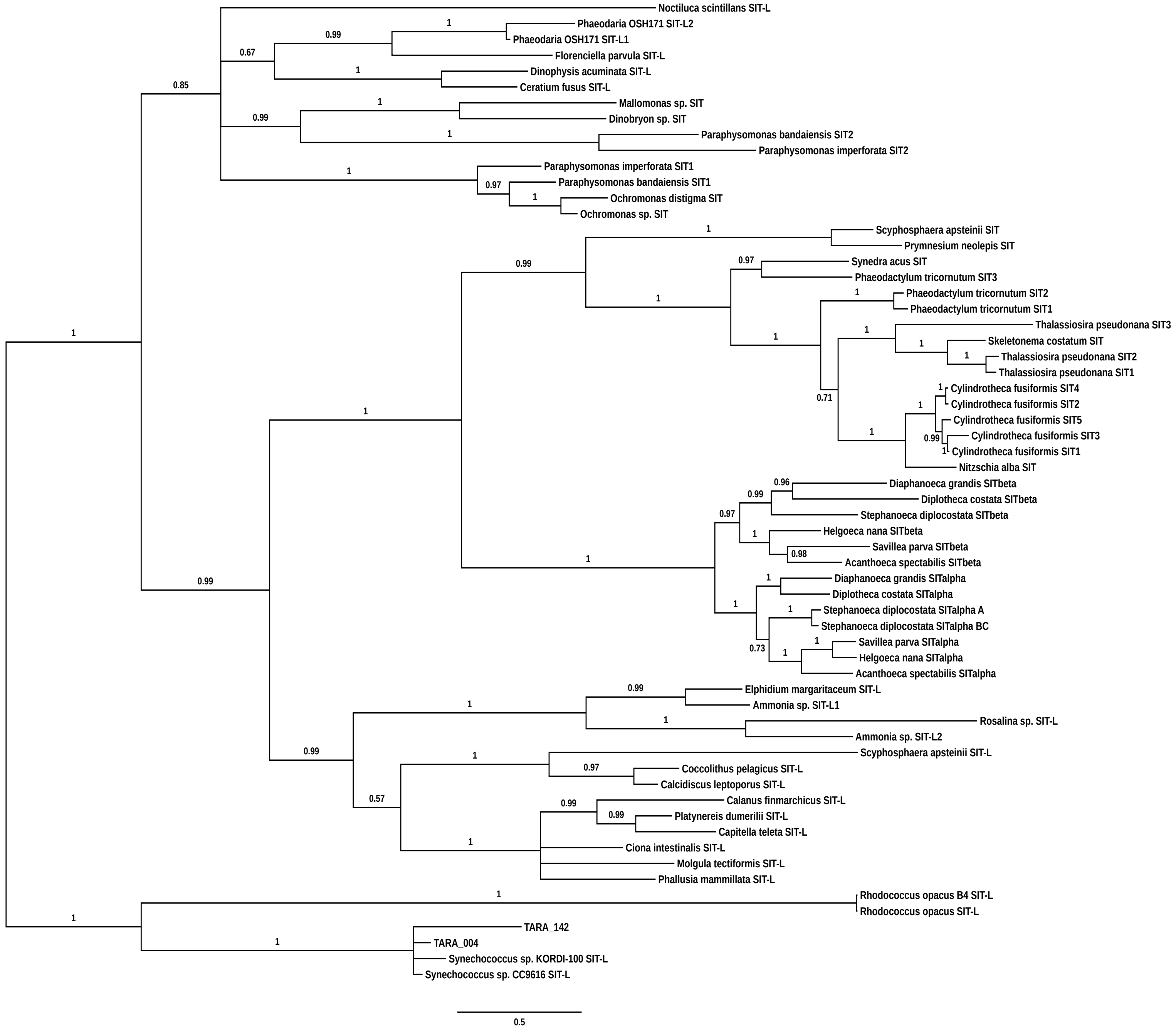

Noctiluca scintillans SIT-L
Phaeodaria OSH171 SIT-L2
Phaeodaria OSH171 SIT-L1
Florenciella parvula SIT-L
Dinophysis acuminata SIT-L
Ceratium fusus SIT-L
Mallomonas sp. SIT
Dinobryon sp. SIT
Paraphysomonas bandaiensis SIT2
Paraphysomonas imperforata SIT2
Paraphysomonas imperforata SIT1
Paraphysomonas bandaiensis SIT1
Ochromonas distigma SIT
Ochromonas sp. SIT
Scyphosphaera apsteinii SIT
Prymnesium neolepis SIT
Synedra acus SIT
Phaeodactylum tricornutum SIT3
Phaeodactylum tricornutum SIT2
Phaeodactylum tricornutum SIT1
Thalassiosira pseudonana SIT3
Skeletonema costatum SIT
Thalassiosira pseudonana SIT2
Thalassiosira pseudonana SIT1
Cylindrotheca fusiformis SIT4
Cylindrotheca fusiformis SIT2
Cylindrotheca fusiformis SIT5
Cylindrotheca fusiformis SIT3
Cylindrotheca fusiformis SIT1
Nitzschia alba SIT
Diaphanoeca grandis SITbeta
Diplotheca costata SITbeta
Stephanoeca diplocostata SITbeta
Helgoeca nana SITbeta
Savillea parva SITbeta
Acanthoeca spectabilis SITbeta
Diaphanoeca grandis SITalpha
Diplotheca costata SITalpha
Stephanoeca diplocostata SITalpha A
Stephanoeca diplocostata SITalpha BC
Savillea parva SITalpha
Helgoeca nana SITalpha
Acanthoeca spectabilis SITalpha
Elphidium margaritaceum SIT-L
Ammonia sp. SIT-L1
Rosalina sp. SIT-L
Ammonia sp. SIT-L2
Scyphosphaera apsteinii SIT-L
Coccolithus pelagicus SIT-L
Calcidiscus leptoporus SIT-L
Calanus finmarchicus SIT-L
Platynereis dumerilii SIT-L
Capitella teleta SIT-L
Ciona intestinalis SIT-L
Molgula tectiformis SIT-L
Phallusia mammillata SIT-L
Rhodococcus opacus B4 SIT-L
Rhodococcus opacus SIT-L
TARA_142
TARA_004
Synechococcus sp. KORDI-100 SIT-L
Synechococcus sp. CC9616 SIT-L
0.85
0.67
0.99
0.99
0.97
0.99
0.97
0.71
0.99
0.96
0.99
0.97
0.98
0.73
0.99
0.99
0.99
0.57
0.97
0.99
0.99
0.5

G

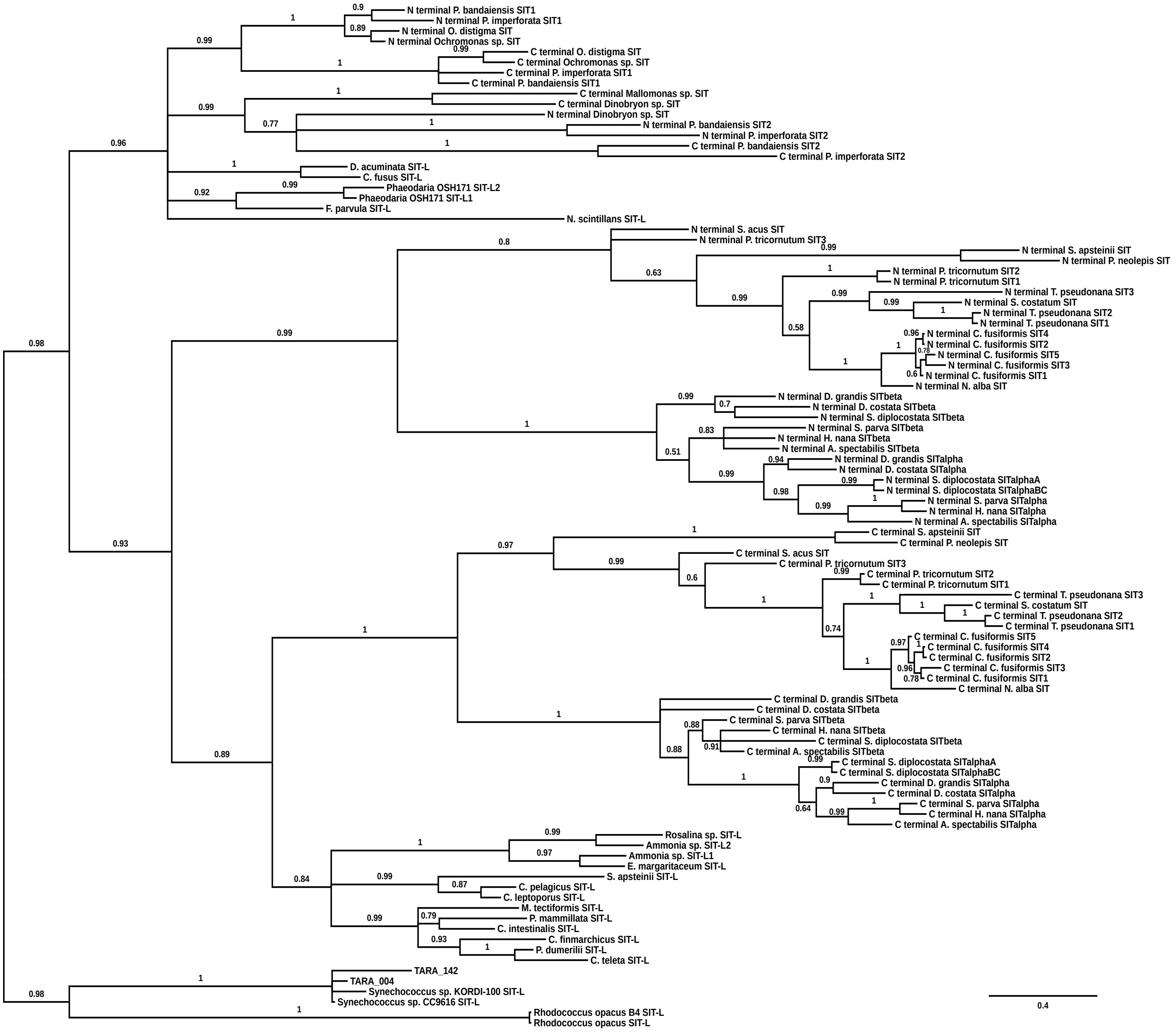

N terminal P. bandaiensis SIT1
N terminal P. imperforata SIT1
N terminal O. distigma SIT
N terminal Ochromonas sp. SIT
C terminal O. distigma SIT
C terminal Ochromonas sp. SIT
C terminal P. imperforata SIT1
C terminal P. bandaiensis SIT1
C terminal Mallomonas sp. SIT
C terminal Dinobryon sp. SIT
N terminal Dinobryon sp. SIT
N terminal P. bandaiensis SIT2
N terminal P. imperforata SIT2
C terminal P. bandaiensis SIT2
C terminal P. imperforata SIT2
D. acuminata SIT-L
C. fusus SIT-L
Phaeodaria OSH171 SIT-L2
Phaeodaria OSH171 SIT-L1
F. parvula SIT-L
N. scintillans SIT-L
N terminal S. acus SIT
N terminal P. tricornutum SIT3
N terminal S. apsteinii SIT
N terminal P. neolepis SIT
N terminal P. tricornutum SIT2
N terminal P. tricornutum SIT1
N terminal T. pseudonana SIT3
N terminal S. costatum SIT
N terminal T. pseudonana SIT2
N terminal T. pseudonana SIT1
N terminal C. fusiformis SIT4
N terminal C. fusiformis SIT2
N terminal C. fusiformis SIT5
N terminal C. fusiformis SIT3
N terminal C. fusiformis SIT1
N terminal N. alba SIT
N terminal D. grandis SITbeta
N terminal D. costata SITbeta
N terminal S. diplocostata SITbeta
N terminal S. parva SITbeta
N terminal H. nana SITbeta
N terminal A. spectabilis SITbeta
N terminal D. grandis SITalpha
N terminal D. costata SITalpha
N terminal S. diplocostata SITalphaA
N terminal S. diplocostata SITalphaBC
N terminal S. parva SITalpha
N terminal H. nana SITalpha
N terminal A. spectabilis SITalpha
C terminal S. apsteinii SIT
C terminal P. neolepis SIT
C terminal S. acus SIT
C terminal P. tricornutum SIT3
C terminal P. tricornutum SIT2
C terminal P. tricornutum SIT1
C terminal T. pseudonana SIT3
C terminal S. costatum SIT
C terminal T. pseudonana SIT2
C terminal T. pseudonana SIT1
C terminal C. fusiformis SIT5
C terminal C. fusiformis SIT4
C terminal C. fusiformis SIT2
C terminal C. fusiformis SIT3
C terminal C. fusiformis SIT1
C terminal N. alba SIT
C terminal D. grandis SITbeta
C terminal D. costata SITbeta
C terminal S. parva SITbeta
C terminal H. nana SITbeta
C terminal S. diplocostata SITbeta
C terminal A. spectabilis SITbeta
C terminal S. diplocostata SITalphaA
C terminal S. diplocostata SITalphaBC
C terminal D. grandis SITalpha
C terminal D. costata SITalpha
C terminal S. parva SITalpha
C terminal H. nana SITalpha
C terminal A. spectabilis SITalpha
Rosalina sp. SIT-L
Ammonia sp. SIT-L2
Ammonia sp. SIT-L1
E. margaritaceum SIT-L
S. apsteinii SIT-L
C. pelagicus SIT-L
C. leptoporus SIT-L
M. tectiformis SIT-L
P. mammillata SIT-L
C. intestinalis SIT-L
C. finmarchicus SIT-L
P. dumerilii SIT-L
C. teleta SIT-L
TARA_142
TARA_004
Synechococcus sp. KORDI-100 SIT-L
Synechococcus sp. CC9616 SIT-L
Rhodococcus opacus B4 SIT-L
Rhodococcus opacus SIT-L
0.4

Supplementary Figure 8. Phylogenetic trees of Silicon Transporters with bacterial sequences omitted and using CAT rate variation to test for phylogenetic artefacts. Removing bacterial SIT-L sequences and phylogenetically analysing only eukaryotic SITs and SIT-Ls results in the same overall tree topology and statistical supports as for the previous analyses (fig. 4, fig. 5, Supplementary Figures 6 and 7). By implementing the CAT model of rate variation in PhyloBayes it is possible to correct for the effects of long-branch attraction caused by fast evolving sequences. Such reanalyses did not produce any significant changes in tree topology; isolated disagreements had low statistical support. These results indicate that including bacterial SIT-Ls does not result in significant phylogenetic artefacts or distorted tree topologies, nor is long-branch attraction caused by fast-evolving sequences a major influence on the topology of the phylogenetic analyses. (A) Tree produced using RaxML maximum likelihood analysis. Numbers at nodes indicate statistical support as a percentage of 100 bootstrap replicates. (B) Tree produced using PhyML maximum likelihood analysis. Numbers at nodes indicate statistical support as a percentage of 1000 SH-aLRT replicates/ 100 bootstrap replicates. (C) Majority rule consensus tree produced using MrBayes Bayesian MCMC analysis. Numbers at nodes indicate posterior probability values. (D) Tree produced using IQ-TREE maximum likelihood analysis. Numbers at nodes indicate statistical support as a percentage of 1000 SH-aLRT replicates/1000 ultrafast bootstrap replicates. (E) Majority rule consensus tree produced using PhyloBayes Bayesian MCMC analysis. All trees produced from the same alignment of 371 amino acid residues using the LG+G4+F model, except for (E) which used the GTR+CAT model. (F) Majority rule consensus tree of SIT-L+Full-SIT sequences produced using PhyloBayes Bayesian MCMC analysis from an alignment of 485 amino acid residues using the GTR+CAT model. (G) Majority rule consensus tree of SIT-L+Half-SIT sequences produced using PhyloBayes Bayesian MCMC analysis from an alignment of 166 amino acid residues using the GTR+CAT model. Note that in all trees the topology is unrooted; Group 2 SIT/SIT-Ls (A-E) or the bacterial SIT-L group (F and G) was arbitrarily designated as an outgroup for presentation purposes. In all trees scale bars indicate average number of amino acid substitutions per site.
